# In trans paired nicking triggers seamless genome editing without double-stranded DNA cutting

**DOI:** 10.1038/s41467-017-00687-1

**Published:** 2017-09-22

**Authors:** Xiaoyu Chen, Josephine M. Janssen, Jin Liu, Ignazio Maggio, Anke E. J. ‘t Jong, Harald M.M. Mikkers, Manuel A. F. V. Gonçalves

**Affiliations:** Department of Molecular Cell Biology, Leiden University Medical Center, Einthovenweg 20, 2333 ZC Leiden, The Netherlands

## Abstract

Precise genome editing involves homologous recombination between donor DNA and chromosomal sequences subjected to double-stranded DNA breaks made by programmable nucleases. Ideally, genome editing should be efficient, specific, and accurate. However, besides constituting potential translocation-initiating lesions, double-stranded DNA breaks (targeted or otherwise) are mostly repaired through unpredictable and mutagenic non-homologous recombination processes. Here, we report that the coordinated formation of paired single-stranded DNA breaks, or nicks, at donor plasmids and chromosomal target sites by RNA-guided nucleases based on CRISPR-Cas9 components, triggers seamless homology-directed gene targeting of large genetic payloads in human cells, including pluripotent stem cells. Importantly, in addition to significantly reducing the mutagenicity of the genome modification procedure, this in trans paired nicking strategy achieves multiplexed, single-step, gene targeting, and yields higher frequencies of accurately edited cells when compared to the standard double-stranded DNA break-dependent approach.

## Abstract (web summary)

CRISPR-Cas9-based gene editing involves double-strand breaks at target sequences, which are often repaired by mutagenic non-homologous end-joining. Here the authors use Cas9 nickases to generate coordinated single-strand breaks in donor and target DNA for precise homology-directed gene editing.

## Introduction

Programmable nucleases, and in particular RNA-guided nucleases (RGNs), are rendering genome editing applicable to numerous basic and applied research settings^1–3^. RGNs are ribonucleoprotein complexes formed by a guide RNA (gRNA) and a Cas9 protein with two nuclease domains, i.e., HNH and RuvC. RGNs cleave DNA complementary to the 5′ end of the gRNA when a contiguous protospacer adjacent motif (PAM) is present^3^. The fact that target DNA cutting is ultimately dictated by simple RNA-DNA hybridization rules confers versatility to RGN technologies^1–3^. A major drawback of conventional DNA editing stems, however, from the fact that double-stranded DNA break (DSB) repair in mammalian cells often takes place via mutagenic non-homologous end joining (NHEJ) instead of accurate homologous recombination (HR)^4^. As a result, allelic and non-allelic mutations, loss-of-heterozygosity, translocations, and other unwarranted genetic changes caused by on-target and off-target DSBs, are frequent^5^. Moreover, NHEJ also contributes to random and imprecise chromosomal insertion of the donor DNA^1, 6^. As a whole, these unpredictable genome-modifying events complicate the interpretation of experimental results and reduce the safety profile of candidate genetic therapies. Despite this, in certain experimental settings, such as those amenable to cell isolation and screening, homology-independent chromosomal DNA insertion is a valuable genetic modification strategy owing to its efficiency and applicability to non-dividing target cells^7–9^.

Following from the above, developing new genome-editing principles that favor not only efficient but also precise homology-directed gene targeting in detriment of mutagenic NHEJ are in demand. Indeed, emergent genome-editing research lines involve testing small RNAs, drugs, or viral proteins that steer DSB repair towards the HR pathway by inhibiting the competing NHEJ^10–12^. Parallel research lines exploit sequence-specific and strand-specific programmable nucleases (“nickases”)^13–17^ for generating single-stranded DNA breaks (SSBs), or nicks, which are non-canonical NHEJ substrates^4^. Besides bypassing DSB formation, “nickases” do not alter the regular cellular metabolism as small RNAs, drugs and viral proteins do. However, genome editing based on “nickases” is inefficient^13, 15–17^. In fact, the investigation of site-specific SSBs as triggers for homology-directed targeting of large DNA segments (e.g., entire transcriptional units) has not been explored.

Here, we investigate the feasibility of exploiting nicking RGNs containing the RuvC Cas9 mutant Asp10Ala (Cas9^D10A^) or the HNH Cas9 mutant His840Ala (Cas9^H840A^) to trigger genome editing via the simultaneous formation of SSBs at endogenous and exogenous DNA. We report that this strategy based on coordinated in trans paired nicking can improve the three main parameters of DNA editing, i.e., efficiency, specificity, and fidelity^1, 2^ and achieves multiplexing homology-directed DNA addition of large genetic payloads.

## Results

### Mutagenesis caused by cleaving Cas9 vs. nicking Cas9

We started by confirming that unwarranted, potentially adverse, genome-modifying events (i.e., target allele mutagenesis and chromosomal translocations)^1^ do occur more frequently in cells exposed to cleaving Cas9 than in those subjected to nicking Cas9 proteins. Firstly, we assessed the mutation rates resulting from RGN complexes consisting of cleaving (i.e., Cas9:gRNA^X^) or nicking Cas9 nucleases (i.e., Cas9^D10A^:gRNA^X^ or Cas9^H840A^:gRNA^X^), where “X” symbolizes the target locus. The Cas9^D10A^ and Cas9^H840A^ proteins differ from wild-type Cas9 in that they have amino-acid substitutions disrupting the catalytic centers of their RuvC and HNH nuclease domains, respectively. As a result, RGN complexes with Cas9^D10A^ and Cas9^H840A^ induce sequence-specific and strand-specific breaks on opposite DNA chains, namely, on the chain complementary and non-complementary to the gRNA, respectively. The *AAVS1* locus at 19q13.42 was selected for these experiments owing to its frequent use as a “safe harbor” for the targeted chromosomal insertion of exogenous DNA^18^. This assessment is based on a series of studies showing that *AAVS1* integrants are neither disturbed by, nor disturb the surrounding genomic environment, providing for long-term and stable transgene expression in different cell types^18^. A target site genotyping assay in human embryonic kidney 293 T cells showed that Cas9:gRNA^S1^ complexes targeting the *AAVS1* locus readily yielded substantially higher levels of DSBs than their Cas9^D10A^:gRNA^S1^ counterparts (Supplementary Fig. 1a). To augment the stringency of the genotyping assay, we next carried out dose–response experiments in human cervix carcinoma HeLa cells using increasing amounts of adenoviral vectors encoding either Cas9 or Cas9^D10A^, each mixed with a fixed amount of an adenoviral vector expressing a gRNA addressing each Cas9 protein to *AAVS1*. A direct relationship between the detection of small insertions and deletions (indels) and nuclease concentrations could be readily established after Cas9:gRNA^S1^ delivery, whereas this was much less so upon Cas9^D10A^:gRNA^S1^ transfer (Supplementary Fig. 1b). These data directly correlated with the much higher frequencies of indel-derived *EGFP* disruption in EGFP^+^ H27 reporter cells triggered by cleaving Cas9:gRNA^GFP2^ when compared to those induced by nicking Cas9^D10A^:gRNA^GFP2^ or by Cas9^H840A^:gRNA^GFP2^ complexes (Supplementary Fig. 1c).

Secondly, we setup a PCR assay to compare the assembly of chromosomal translocations caused by the formation of DSBs vs. SSBs at two distinct loci. To this end, HeLa cells were transfected with plasmids coding for cleaving or nicking RGNs targeting *DMD* and *AAVS1* sequences. Amplicons diagnostic for translocation events between *DMD* and *AAVS1* were exclusively detected in cells exposed to the cleaving RGNs (Supplementary Fig. 1d). Sanger sequencing of individual amplicons established their origin at t(X;19)(p21;q13) (Supplementary Fig. 1e). Taken together, these experiments formally demonstrate that unwarranted, potentially adverse, genome-modifying events occur more frequently in cells receiving RGNs containing cleaving Cas9 than in those harboring nicking Cas9^D10A^.

### In trans paired nicking yields seamless *DMD* gene targeting

**Fig. 1:**
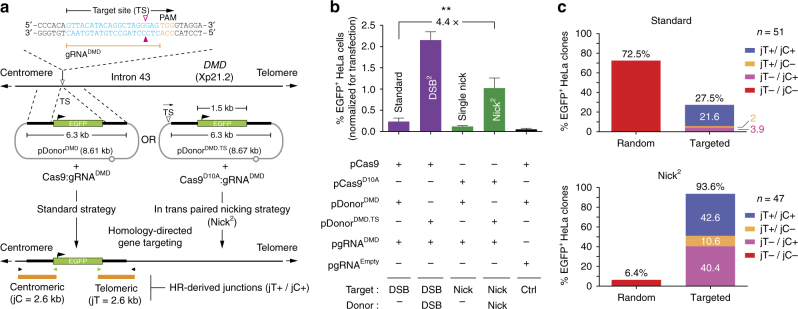
Homology-directed *DMD*-targeting using standard and in trans paired nicking strategies. **a** Schematics of standard and in trans paired nicking (Nick^2^) procedures. The former involve DSB formation only at the target sequence; the latter comprise SSB formation at target plus donor sequences. pDonor^DMD^ and pDonor^DMD.TS^ have their transgenes flanked by sequences identical to those framing the gRNA^DMD^ target site (TS). *Open* and *solid magenta arrowheads*, position of the phosphodiester bond cleavage induced by Cas9’s RuvC and HNH nuclease domains, respectively. *Solid arrowhead*, position of the SSB induced by Cas9^D10A^. The modified pDonor^DMD.TS^ differs from pDonor^DMD^ in that it has the gRNA^DMD^ TS next to its targeting module. The transgene is formed by human *PGK1* promoter, *EGFP* ORF, and bovine *GH1* polyadenylation signal sequences. Cas9:gRNA^DMD^ and Cas9^D10A^:gRNA^DMD^ are cleaving and nicking RGN complexes, respectively. PAM protospacer adjacent motif. An integrant generated by HR events at both ends of the targeting module is depicted. The amplicons specific for telomere-sided and centromere-sided transgenic-*DMD* junctions (jT and jC, respectively), are equally shown. *Horizontal arrowheads*, primers. **b** Quantification of stable transfection levels by flow cytometry. Flow cytometry of long-term HeLa cell cultures initially exposed to the indicated plasmids. The *bars* correspond to mean ± s.d. of three independent biological replicates done on different days. **P = 0.006 (two-tailed *t*-test). **c** Cumulative molecular characterization of integrants generated by the conventional vs. the in trans paired nicking strategies. The frequencies of clones with random insertions (jT−/jC−), HR-derived telomeric junctions (jT+/jC−), HR-derived centromeric junctions (jT−/jC+) and HR-derived telomeric and centromeric junctions (jT+/jC+) are plotted. The corresponding PCR screening data are presented in Supplementary Fig. 3

Next, we sought to investigate homology-directed gene targeting based on inducing DSBs vs. SSBs not only at acceptor chromosomal sequences but also at donor DNA templates. The *DMD* gene at Xp21.2 was chosen as target locus. By spanning over 2.4 Mb, *DMD* is the largest human protein-coding gene known. Of note, defective *DMD* alleles cause Duchenne muscular dystrophy (DMD), a progressive lethal neuromuscular disease affecting ∼1 in 3500–5000 boys^19, 20^. For these experiments, we generated plasmid pgRNA^DMD^, to address Cas9 proteins to *DMD* intron 43, and EGFP-encoding constructs pDonor^DMD^ and pDonor^DMD.TS^ to serve as exogenous HR substrates (Fig. 1a). Construct pDonor^DMD.TS^ differs from pDonor^DMD^ in that it has a target site (TS) for gRNA^DMD^ next to its targeting module (Fig. 1a). Importantly, all transgene-containing donors used in the present study have autonomous transcription units, which in contrast to splice acceptor-containing gene trapping constructions, avoid biased selection of on-target integrants^21^. Genome-editing experiments were initiated by exposing HeLa cells to pDonor^DMD^ and cleaving Cas9:gRNA^DMD^ complexes (standard setting) or to pDonor^DMD.TS^ and nicking Cas9^D10A^:gRNA^DMD^ complexes (in trans paired nicking; Nick^2^). After eliminating episomal DNA by sub-culturing, genetically modified cells were quantified through flow cytometry. This analysis revealed that the in trans paired nicking strategy led to significantly higher percentages of genetically modified cells when compared to those obtained through the standard approach (Fig. 1b). Similar results were obtained by using donor constructs whose *DMD*-targeting modules were flanked by the gRNA^DMD^ TS in a direct or inverted repeat orientation (Supplementary Fig. 2). These data are consistent with earlier theoretical models and more recent experimental systems indicating a role for nicked HR partners as recombination-initiating substrates^22, 23^. Of note, although at this target sequence paired DSB formation (in trans paired breaking; DSB^2^) yielded the highest frequencies of EGFP^+^ cells, the resulting free-ended HR substrates are prone to aberrant concatemer assembly (see below). Indeed, it has been previously shown that the DSB^2^ strategy results in higher frequencies of random chromosomal insertions through illegitimate recombination processes when compared to those obtained by the standard DSB-dependent gene targeting approach^6^. Conversely, consistent with previous studies^13, 15–17^, generating SSBs exclusively at chromosomal DNA yielded the lowest frequencies of stably transfected cells.

Subsequently, we compared in trans paired nicking with standard gene targeting in terms of their relative specificities and fidelities. The specificity is ascertained by detecting donor sequences at the target site; the fidelity is established by demonstrating that telomere-sided and centromere-sided junctions between donor and target DNA are formed through error-free HR (jT+ and jC+, respectively). Randomly selected EGFP^+^ HeLa clones (*n* = 98) were screened via PCR assays targeting both junctions (Fig. 1c and Supplementary Fig. 3). In the set of clones modified through the delivery of pDonor^DMD^, pCas9 and pgRNA^DMD^ (*n* = 51), the *DMD*-targeted fraction was 27.5% with 21.6% of these integrants being accurately targeted (jT+/jC+). Notably, in the set of clones modified via the transfer of pDonor^DMD.TS^, pCas9^D10A^ and pgRNA^DMD^ (*n* = 47), these fractions were 93.6% and 42.6%, respectively (Fig. 1c). We conclude that, when compared to conventional DSB-induced gene targeting, in trans paired nicking was more efficient, specific, and accurate at the *DMD* locus.

### In trans paired nicking yields seamless *AAVS1* gene targeting

**Fig. 2:**
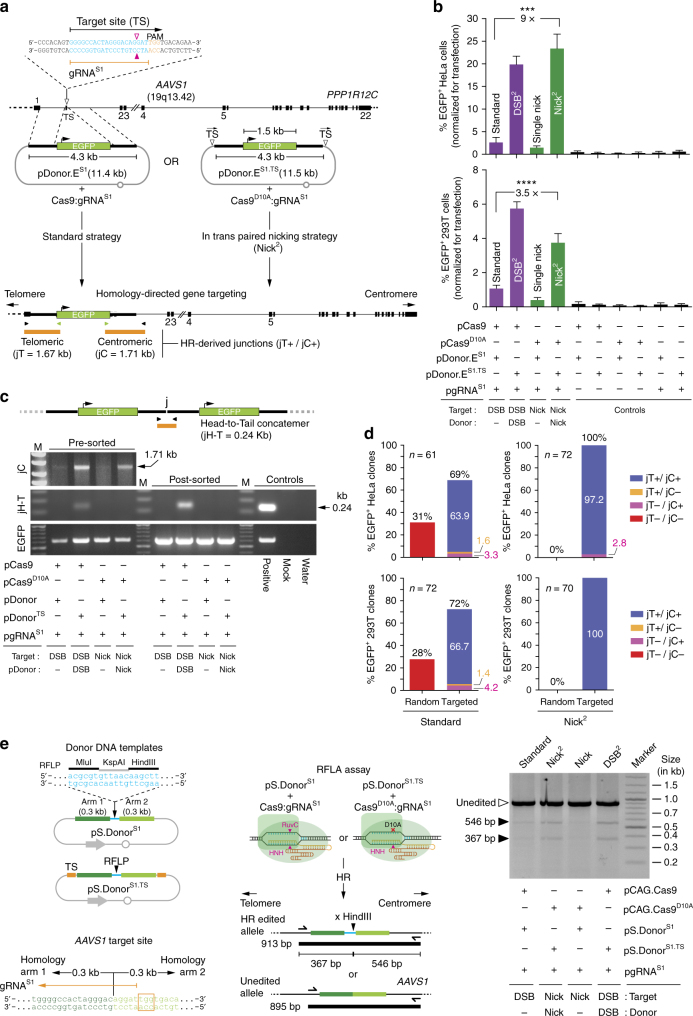
Homology-directed *AAVS1* targeting using standard and in trans paired nicking strategies. **a** Diagram of standard and in trans paired nicking (Nick^2^) procedures. The former involve DSB formation only at the target sequence; the latter comprise SSB formation at target plus donor sequences. pDonor^S1^ and pDonor^S1.TS^ have their transgenes framed by sequences homologous to *AAVS1*. pDonor^S1.TS^ differs from pDonor^S1^ in that it has the gRNA^S1^ target site (TS) bracketing its EGFP-encoding targeting module. Cas9:gRNA^S1^ and Cas9^D10A^:gRNA^S1^ are cleaving and nicking RGNs, respectively. *Open* and *solid magenta arrowheads*, position of the phosphodiester bond cleavage induced by Cas9’s RuvC and HNH nuclease domains, respectively. *Solid arrowhead*, position of the SSB induced by Cas9^D10A^. Amplicons diagnostic for telomere-sided and centromere-sided transgenic-*AAVS1* junctions (jT and jC, respectively), are depicted. **b** Quantification of stably transfected cells. Flow cytometry of long-term HeLa and 293 T cell cultures initially transfected with the indicated plasmids. The *bars* correspond to mean ± s.d. of six biological replicates from two independent experiments (three biological replicates per experiment). ****P < 0.0001 (two-tailed *t*-tests). **c** Probing for wanted (gene targeting) and unwanted (concatemerization) genome-modifying events. Amplicons diagnostic for gene targeting (jC) and head-to-tail concatemers (jH-T) in 293 T cell populations transfected with the indicated constructs are presented. This assay was also run on EGFP-sorted cells (post-sorted). *EGFP* served as an internal control template. **d** Cumulative molecular characterization of integrants generated by the conventional and in trans paired nicking strategies. The frequencies of clones with random insertions (jT−/jC−), HR-derived telomeric junctions (jT+/jC−), HR-derived centromeric junctions (jT−/jC+) and HR-derived telomeric and centromeric junctions (jT+/jC+) are plotted. The respective PCR screening data are presented in Supplementary Fig. 7. **e** Homology-directed *AAVS1* editing after inducing DSBs or SSBs. pS.Donor^S1^ and pS.Donor^S1.TS^ have a restriction-fragment length polymorphism (*RFLP*) flanked by 300-bp *AAVS1* sequences (“arms”). pDonor^S1.TS^ has the gRNA^S1^ TS flanking its targeting module (*orange boxes*). *RFLA* restriction-fragment length analysis; *half arrows* primers; PAM boxed sequence. RFLA products diagnostic for unedited and HR-edited *AAVS1* alleles retrieved from HeLa cells transfected with the indicated plasmid combinations are identified by *open* and *closed arrowheads*, respectively

We next examined the performance of in trans paired nicking and standard gene targeting at *AAVS1* (Fig. 2a). As aforementioned, this locus is commonly used as a “safe harbor” for the chromosomal insertion of exogenous DNA in human cells^18^. These experiments were initiated by transfecting HeLa and 293 T cells with pDonor.E^S1^ or pDonor.E^S1.TS^ each mixed with plasmids encoding either Cas9:gRNA^S1^ or Cas9^D10A^:gRNA^S1^ (Fig. 2a). The pDonor.E^S1.TS^ construct has its targeting module flanked by two gRNA^S1^ TS (Fig. 2a). The rationale for this donor design was provided by the experiments showing that such arrangement yields significantly higher frequencies of stably transfected cells when compared to isogenic templates containing a single gRNA^S1^ TS (Supplementary Fig. 4). In agreement with the *DMD*-targeting experiments, when compared to experiments involving single DSBs (standard setting) or single SSBs, in trans paired nicking of *AAVS1* and pDonor.E^S1.TS^ led to significantly higher percentages of genetically modified cells (Fig. 2b). Similar results were gathered by using different gRNA and donor DNA reagents or the alternative nicking Cas9^H840A^ variant whose inactivated HNH domain assures that SSBs occur at the DNA chain opposite to that hydrolyzed by its RuvC-disabled Cas9^D10A^ counterpart (Supplementary Fig. 5). Importantly, amplicons diagnostic for HR-derived integrants were readily retrieved not only from cells subjected to inaccurate DNA editing by paired DSB formation but also from cells exposed to the accurate in trans paired nicking procedure (Fig. 2c). Indeed, in striking contrast to inducing in trans paired DSBs (DSB^2^), generating in trans paired SSBs (Nick^2^), did not result in the assembly of disruptive donor DNA concatemers (Fig. 2c), presumably emerging through ligation of free-ended termini generated in cellula by Cas9:gRNA^S1^ ^6^. Finally, we probed an alternative in trans paired nicking gene targeting strategy in which two different gRNAs generate tandem SSBs within the interacting homologous sequences. This strategy, tandem paired nicking, yielded stable transfection levels that were within the range of those achieved by using the standard, DSB-dependent, gene targeting procedure (Supplementary Fig. 6).

To gauge the specificity and fidelity resulting from in trans paired nicking vs. standard gene targeting at *AAVS1*, randomly selected EGFP^+^ clones (*n* *=* 275) were isolated from HeLa and 293 T cell populations and were screened through junction PCR (Fig. 2d and Supplementary Fig. 7). We observed that 63.9% and 66.7% of the HeLa and 293 T cells exposed to the standard setting underwent accurate homology-directed gene targeting (jT+/jC+), respectively (Fig. 2d). In the remaining clones, illegitimate recombination led instead to off-target integrants (jT−/jC−) and to on-target integrants lacking HR-derived junctions either from the centromeric or telomeric side (jT+/jC− or jT−/jC+, respectively). Remarkably, the fraction of properly targeted HeLa and 293 T cells subjected to in trans paired nicking was as high as 97.2 and 100%, respectively (Fig. 2d). Finally, Sanger sequencing established that precisely targeted integrants resulting from in trans paired nicking and conventional gene targeting were undistinguishable (Supplementary Fig. 8).

To complement the previous gene targeting experiments involving sizable and transcriptionally active donor constructs, we next asked whether short, transcriptionally inert donor constructs, can equally serve as in trans paired nicking substrates. To this end, *AAVS1-*targeting plasmids pS.Donor^S1^ and pS.Donor^S1.TS^, resistant and susceptible to RGNs, respectively (Fig. 2e, *left panel*), were transfected into human cells together with constructs expressing Cas9:gRNA^S1^ or Cas9^D10A^:gRNA^S1^ (Fig. 2e, *middle panel*). HR engaging pS.Donor^S1^ or pS.Donor^S1.TS^ sequences should result in the targeted chromosomal insertion of 18-bp DNA fragments incorporating restriction enzyme polymorphisms (Fig. 2e, *middle panel*). Detection of these genome-editing events by restriction enzyme fragment length analysis (RFLA) revealed that in trans paired nicking is compatible with the use of short, transcriptionally inert, donor DNA templates (Fig. 2e, *right panel*).

Paired RGNs inducing offset nicks on opposite chromosomal DNA strands ensure that DSBs are mostly restricted to their bipartite target sequences owing to the coordinated and local formation of SSBs on both polynucleotide chains^24, 25^. The resulting gains in DNA cutting specificity render this dual RGN approach appealing, hereafter named in cis paired nicking for the sake of consistency. Hence, albeit dependent on two gRNAs and on the generation of mutagenic DSBs, we sought nonetheless to compare in cis with in trans paired nicking as stimuli for site-specific chromosomal DNA insertion (knock-in). Therefore, in addition to the four experimental conditions tested before (Fig. 2b), in these new experiments, we transfected human cells with pDonor^S1^ and pCAG.Cas9^D10A^ mixed with constructs expressing two different *AAVS1*-specific gRNA pairs (i.e., gRNA^S1^/gRNA^S1.2^ or gRNA^S1^/gRNA^S1.3^). Consistent with the previous data (Fig. 2b), the in trans paired nicking setup yielded the highest frequencies of genetically modified cells. The in cis paired nicking strategy led, in turn, to frequencies of genetically modified cells that were in the range of those obtained by inducing DSBs or SSBs exclusively at the target site (Supplementary Fig. 9).

### In trans paired nicking in pluripotent stem cells

**Fig. 3:**
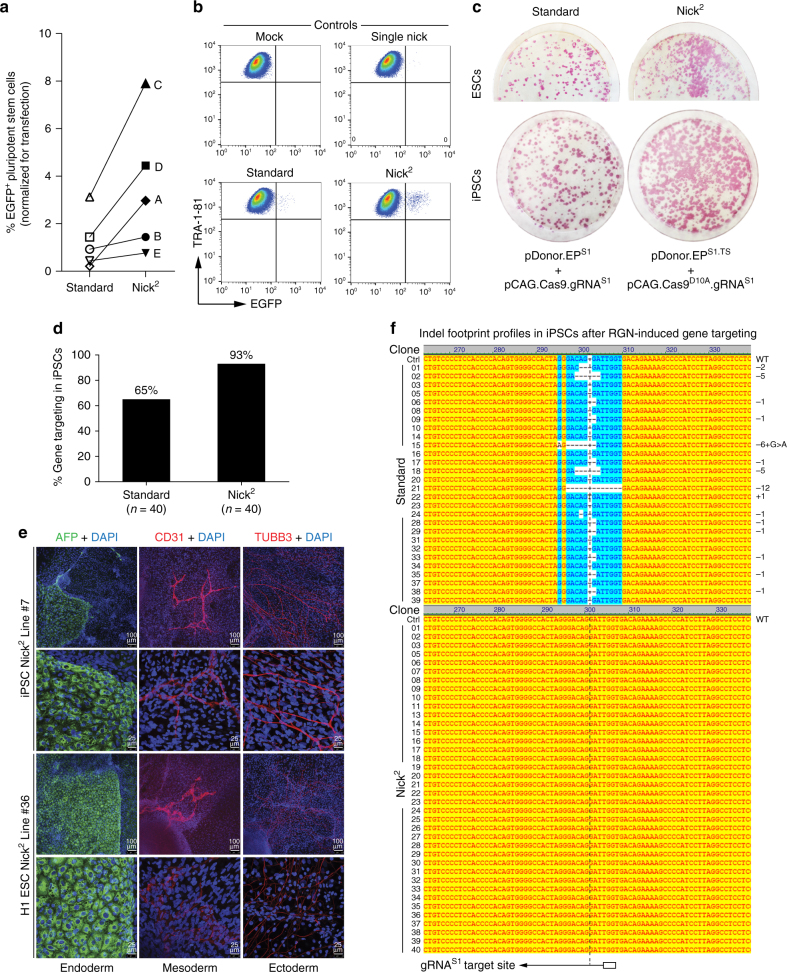
Comparing RGN-induced gene targeting based on standard and in trans paired nicking in human PSCs. **a** Quantification of genetically modified PSCs by flow cytometry. Cultures of iPSCs (A, B, and E) and ESCs (C and D) were exposed to *AAVS1*-specific cleaving Cas9:gRNA^S1^ (standard) or nicking Cas9^D10A^:gRNA^S1^ (Nick^2^) complexes mixed with RGN-resistant or RGN-susceptible donor constructs, respectively, encoding either EGFP or Puro^R^.T2A.EGFP. The frequencies of gene-modified PSCs were determined by flow cytometric quantification of EGFP^+^ and TRA-1-81^+^ dually labeled cells. **b** Representative flow cytometry dot plots corresponding to RGN-induced gene targeting experiments in PSCs. **c** Detection of gene-modified PSCs by colony-formation assays. ESCs (*top*) and iPSCs (*bottom*) were co-transfected with the indicated plasmids. After puromycin selection, alkaline phosphatase staining identified genetically modified PSC colonies. **d** RGN-induced gene targeting frequencies at *AAVS1* in iPSCs. Junction PCR analyses of puromycin-resistant colonies from iPSC cultures initially co-transfected with pDonor.EP^S1^ and pCas9.gRNA^S1^ (standard) or with pDonor.EP^S1.TS^ and pCas9^D10A^.gRNA^S1^ (Nick^2^). The respective PCR screening data are presented in Supplementary Fig. 13. **e** Differentiation potential of gene-edited PSCs. ESC and iPSC lines were targeted at *AAVS1* by in trans paired nicking. Cell types characteristic of ectoderm, endoderm, and mesoderm were identified by confocal immunofluorescence microscopy for TUBB3, AFP, and CD31, respectively. **f** Characterization of indel footprints in iPSCs subjected to standard vs. in trans paired nicking. Nucleotide sequencing of *AAVS1* target alleles in randomly selected iPSC clones (*n* = 68) genetically modified by DSB-dependent and in trans paired nicking methodologies (Standard and Nick^2^, respectively). Indel footprints were exclusively identified in iPSCs subjected to the standard gene targeting approach (15/28). The gRNA^S1^ target site is indicated underneath the sequence reads. *Open box* PAM; *vertical dashed line* position of expected RGN-induced phosphodiester bond cleavage; *Ctrl* reference wild-type nucleotide sequence from unedited cells

Despite their patent scientific and biomedical importance, genetic manipulation of human pluripotent stem cells ﻿(PSCs) remains limited by the typically low efficiency, specificity, and accuracy of homology-directed gene targeting, even when using programmable nucleases (see e.g., ref. ^21^). Therefore, we investigated the performance of in trans paired nicking in human induced PSCs (iPSCs; Supplementary Fig. 10) and human embryonic stem cells (ESCs)^26^. In addition to pDonor^S1^ and pDonor^S1.TS^ (Supplementary Fig. 4a), we included in these experiments, pDonor.EP^S1^ and pDonor.EP^S1.TS^ encoding Puro^R^.T2A.EGFP instead of EGFP. The data generated with these new HR substrates in HeLa cells (Supplementary Fig. 11) were similar to those of previous experiments showing the superiority of in trans paired nicking over standard gene targeting in achieving efficient cell engineering at *AAVS1* (Fig. 2b and Supplementary Figs. 4b–6 and 9). Importantly, this superiority was equally established in iPSCs and ESCs by using dual-color flow cytometry and colony-formation assays involving the detection of EGFP^+^/TRA-1-81^+^ cells (Fig. 3a, b) and puromycin-resistant colonies stained for alkaline phosphatase, respectively (Fig. 3c). In addition, when compared to in trans paired nicking, DSB-triggered *AAVS1* targeting induced higher frequencies of apoptotic Annexin V^+^ cells in ESC cultures (Supplementary Fig. 12). These results are consistent with the well-established sensitivity of PSCs to DSBs^27^.

To determine the precision of genome editing in iPSCs subjected to in trans paired nicking vs. standard genome-editing protocols, puromycin-resistant clones (*n* *=* 80) were screened with a PCR assay specific for HR-derived junctions (Fig. 3d and Supplementary Fig. 13). The gene targeting specificity in iPSCs exposed to standard and in trans paired nicking procedures was 65 and 93%, respectively (Fig. 3d and Supplementary Fig. 13). Contributing to the difficulty in isolating iPSC lines that undergo seamless genome editing is the fact that a sizable fraction of cells, in addition to the intended genetic modification at one of the target alleles, harbor mutations at the other allele^28^. These mutations correspond to unpredictable indel footprints created after NHEJ-mediated repair of targeted DSBs^28^. Hence, to further characterize the genetically modified iPSCs, nucleotide sequence analysis of target DNA was performed in individual iPSC clones subjected to standard and in trans paired nicking protocols. This analysis revealed the presence of a range of indel footprints exclusively in the iPSC lines generated by standard gene targeting (Fig. 3f). Indeed, the *AAVS1* target site remained pristine in all of the randomly selected iPSC lines obtained after applying the in trans paired nicking protocols (Fig. 3f). These results are in agreement with our previous data (Supplementary Fig. 1) and the fact that, in contrast to DSBs, SSBs are not canonical substrates for NHEJ.

Finally, iPSC lines genetically engineered through standard and in trans paired nicking remained pluripotent (Fig. 3e and Supplementary Fig. 14). We conclude that, instead of generating DSBs, targeted DNA integration at the *AAVS1* “safe harbor” in different cell types is best achieved via coordinated RGN-induced paired nicking of donor and acceptor DNA.

### Multiplexing gene targeting by in trans paired nicking

**Fig. 4:**
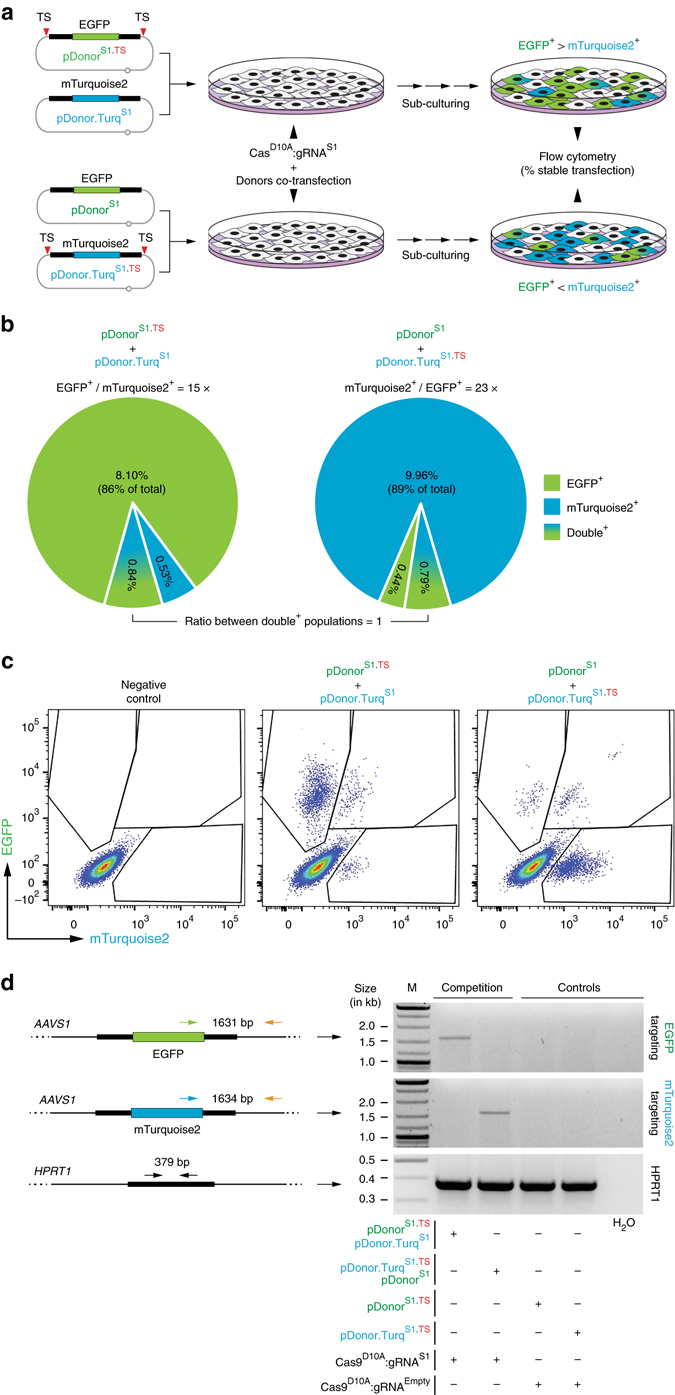
Competition for gene targeting between donor DNA resistant and sensitive to RGN-induced nicking. **a** Schematics of the experimental design. HeLa cells were co-transfected with the indicated donor templates together with plasmids encoding nicking Cas9^D10A^:gRNA^S1^. **b** Quantification of stably transfected cell populations. The frequencies of genetically modified cells were determined at 27 days post-transfection by EGFP-directed and mTurquoise2-directed flow cytometry. The ratios between the frequencies of the various gene-modified subpopulations are presented. **c** Flow cytometry dot plots corresponding to the end-point of the experiments. Mock-transfected cultures served to set the thresholds for background fluorescence (﻿n﻿egative control). **d** Gene targeting in cells containing donor DNA resistant and susceptible to RGN nicking. Amplicons diagnostic for homology-directed gene targeting involving EGFP-encoding and mTurquoise2-encoding donor templates are indicated. *HPRT1* provided for an internal control target sequence

To confirm that *AAVS1*-targeting donor DNA subjected to RGN nicking is a superior substrate for site-specific chromosomal DNA insertion, we setup competition experiments involving the co-targeting of two donors each encoding a different reporter, i.e., EGFP or mTurquoise2 (Fig. 4a). For these experiments, one of the two donors contained TS sequences, whereas the other did not (Fig. 4a). Flow cytometry showed that pDonor^S1.TS^ and pDonor.Turq^S1.TS^ subjected to RGN-induced nicking led to 15-fold and 23-fold higher frequencies of genetically modified cells, respectively, when compared to their competitor, RGN-resistant, donor counterparts pDonor^S1^ and pDonor.Turq^S1^ (Fig. 4b, c). Consistent with these results, homology-directed gene targeting in cells containing both RGN-resistant and RGN-susceptible donors involved primarily the latter substrates, independently of the product that they encoded (Fig. 4d).

Hitherto, multiplexing genome editing has primarily entailed NHEJ-based manipulations such as those involving RGN pairs for knocking-out two genes simultaneously or for creating chromosomal deletions^1^. Such approaches are, however, not applicable for the targeted addition of new genetic information. For this purpose, multiplexing homology-directed DNA insertion based on different donor constructs can, in principle, be used instead. Unfortunately, HR-dependent chromosomal knock-in of two different donors in individual cells is a very rare event. Moreover, in addition to generating high frequencies of indel footprints, the necessary programmable nuclease pairs can induce loss-of-heterozygosity and/or translocations (Supplementary Fig. 1). Therefore, engineering cells with exogenous DNA inserted at two different loci or at two alleles of a single locus (bi-allelic targeting) is normally a complex and time-consuming procedure. Indeed, these procedures include constructing donors with positive/negative selection markers for isolating and screening the few cells that undergo seamless gene targeting, often followed by marker removal. This lengthy process is subsequently repeated on the selected cell clone(s) using, this time, a second donor construct.

**Fig. 5:**
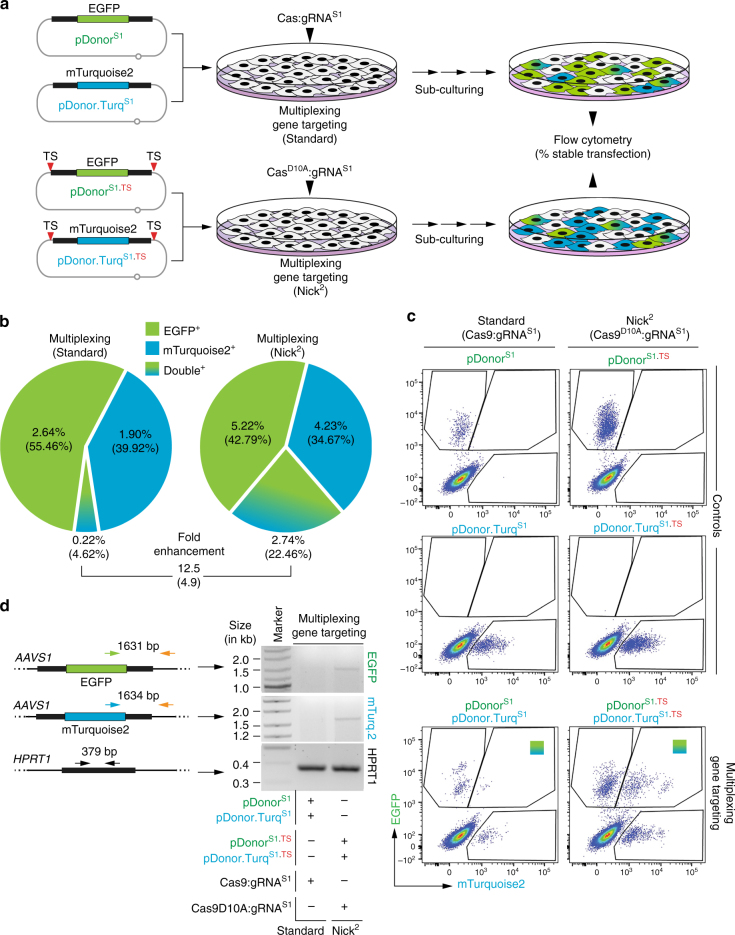
Multiplexing homology-directed DNA addition. **a** Diagram of the experimental design. HeLa cells were co-transfected with the indicated donor constructs together with plasmids encoding either cleaving Cas9:gRNA^S1^ or nicking Cas9^D10A^:gRNA^S1^ complexes. **b** Quantification of stably transfected cell populations. The frequencies of genetically modified cells were determined at 27 days post-transfection by EGFP-directed and mTurquoise2-directed flow cytometry. The ratios between the frequencies of the double-positive cell populations generated by standard and in trans paired nicking multiplexing, are presented. *Numerals* between *brackets* correspond to the fraction of each gene-modified subpopulation. **c** Flow cytometry dot plots corresponding to the end-point of the experiments. Parallel cultures transfected with a single donor construct mixed with plasmids expressing Cas9:gRNA^S1^ or Cas9^D10A^:gRNA^S1^ served as controls for setting the thresholds for EGFP and mTurquoise2 detection. **d** Gene co-targeting in cells containing a mixture of two donors resistant or susceptible to RGN nicking. PCR products specific for homology-directed gene targeting involving EGFP-encoding and mTurquoise2-encoding donor templates are indicated. *HPRT1* provided for an internal control target sequence

We thus sought to capitalize on the higher efficiency, specificity and accuracy of in trans paired nicking over the conventional DSB-dependent strategy at *AAVS1*, for testing one-step co-targeting of different alleles. These multiplexing knock-in experiments were initiated by exposing HeLa cells to pDonor^S1.TS^, pDonor.Turq^S1.TS^, and nicking Cas9^D10A^:gRNA^S1^ (Fig. 5a). Controls consisted of treating HeLa cells with pDonor^S1^, pDonor.Turq^S1^, and cleaving Cas9:gRNA^S1^ (Fig. 5a). Remarkably, in comparison with the control setting, the multiplexing approach based on in trans paired nicking yielded one order of magnitude higher amounts of doubly-labeled EGFP^+^/mTurquoise2^+^ cells as measured by flow cytometry (Fig. 5b, c). These results directly correlated with the detection of HR-specific amplicons in parallel genomic DNA samples (Fig. 5d). After flow cytometry-assisted sorting of these EGFP^+^/mTurquoise2^+^ cells (Supplementary Fig. 15), single-cell clonal analysis (*n* = 35) revealed that 89% of them underwent *AAVS1*-targeting events, of which 94% were bi-allelic events involving both donor DNA templates (Supplementary Fig. 16a, b). An independent assay based on Southern blot analysis confirmed co-targeting of both expression units in individual cells without evidence for random chromosomal DNA insertion (Supplementary Fig. 16a, c). Taken together, these data show that simultaneous in trans paired nicking of independent donor substrates can provide for a simpler and faster strategy for achieving, in a seamless manner, multiplexed addition of foreign DNA into the genome of human cells.

### In trans paired nicking yields seamless gene editing at *CCR5*

**Fig. 6:**
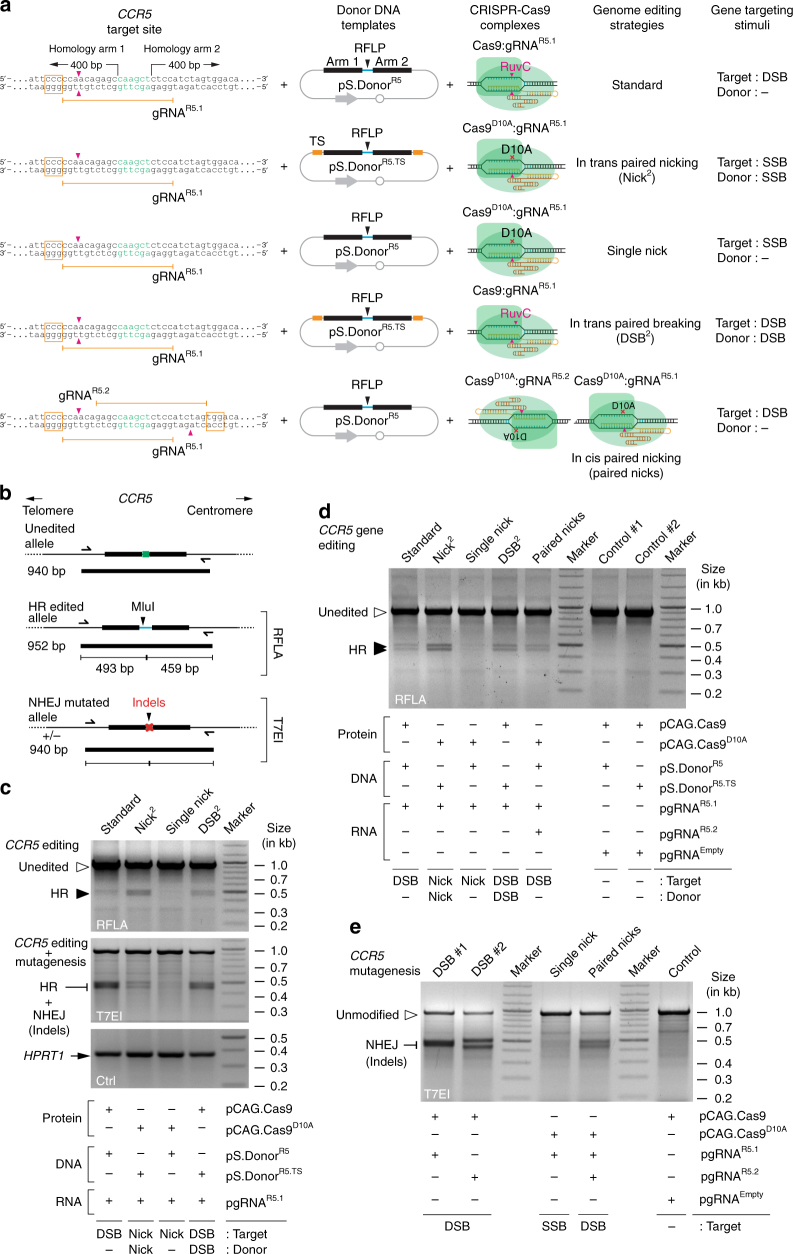
Homology-directed *CCR5* editing after DSB vs. SSB generation. **a** Diagram of the different DSB-dependent and SSB-dependent genome-editing strategies. pS.Donor^R5^ and pS.Donor^R5.TS^ have a restriction-fragment length polymorphism (RFLP) flanked by 400-bp *CCR5* sequences (“arms”). pS.Donor^R5.TS^ has the gRNA^R5.1^ target site (TS) bracketing its targeting module (*orange boxes*). Combining Cas9^D10A^:gRNA^R5.2^ and Cas9^D10A^:gRNA^R5.1^ complexes generates a targeted DSB by nicking on opposite DNA strands (in cis paired nicking strategy). PAMs boxed sequences; *magenta arrowheads*, positions of the DSBs and SSBs generated by Cas9 and Cas9^D10A^, respectively. **b** Schematics of the *CCR5* genotyping assays. DNA products diagnostic for unedited, edited, and mutagenized *CCR5* alleles are indicated. *RFLA* restriction-fragment length analysis; *T7EI* mismatch-sensing T7 endonucleases I assay; *half arrows*, primers **c** *CCR5* genotyping assays. Genotyping of *CCR5* sequences by RFLA and T7EI assays in HeLa cells transfected with the indicated plasmid sets. RFLA products specific for unedited and HR-edited *CCR5* alleles are identified by *open* and *closed arrowheads*, respectively; T7EI digestion products diagnostic for genetic changes induced at *CCR5* by HR and NHEJ are equally indicated. The genomic DNA analyses were performed at 3 days post-transfection. **d** Comparing genome-editing strategies based on single vs. dual RGNs. HeLa cells were co-transfected with the indicated plasmids and 3 days later RFLA was performed on their genomic DNA. *Open* and *solid arrowheads* point to unedited and HR-edited *CCR5* sequences, respectively. **e** Comparing *CCR5* mutagenesis in cells exposed to RGNs inducing DSBs vs. SSBs. HeLa cells were co-transfected with the indicated plasmids and T7EI genotyping assays were carried out 3 days later. T7EI products diagnostic for indel footprints left after NHEJ-mediated DSB repair are pinpointed by the *flat arrowhead*

The product of the C–C motif chemokine receptor 5 gene *CCR5*, located at 3p21.31, serves as an HIV-1 co-receptor on macrophages and T cells^29^. Crucially, individuals homozygous for a 32-bp deletion disrupting *CCR5* function (*CCR5Δ32*) are healthy and refractory to R5-tropic HIV-1 infection^29^. Hence, this locus is an appealing target for testing HIV therapies based on viral co-receptor knockout and site-specific “stacking” of restriction factor genes^29^. In addition, similarly to *AAVS1*, *CCR5* is frequently used as a generic “safe harbor” for the targeted chromosomal insertion of foreign DNA in human cells^18^. Thus, we next sought to compare DSB-dependent vs. SSB-dependent genome-editing approaches at *CCR5* after delivering RGNs together with *CCR5*-targeting constructs pS.Donor^R5^ or pS.Donor^R5.TS^ marked with restriction enzyme polymorphisms (Fig. 6a). In these experiments, RFLA and mismatch-sensing T7 endonuclease I (T7EI) genotyping assays were deployed for assessing genomic changes through HR and/or NHEJ (Fig. 6b). Human cells treated with in trans paired nicking (Nick^2^) and in trans paired breaking (DSB^2^) protocols readily yielded noticeable HR-specific RFLA products (Fig. 6c, *top panel*). A preponderance of T7EI-digested products, diagnostic for the cumulative build-up of NHEJ and HR events, was detected in cells subjected to DSB-inducing protocols (Fig. 6c, *middle panel*). This outcome is consistent with the prevalence of the former over the latter pathway during the repair of DSBs in mammalian cells^4^. Of note, T7EI-digested products corresponding to the in trans paired nicking protocol should mostly represent HR events as nicking exclusively at *CCR5* (single nick) led to the lowest signals in both genotyping assays (Fig. 6c). In a follow-up experiment, in addition to the four experimental conditions tested earlier (Fig. 6c), we included in cis paired nicking at *CCR5* by transfecting HeLa cells with pS.Donor^R5^ and pCas9^D10A^ mixed with plasmids expressing the gRNA pair gRNA^R5.1^/gRNA^R5.2^. In agreement with the previous data (Fig. 2c), in trans paired nicking induced robust accumulation of HR-specific RFLA products. Importantly, cells exposed to DSB-inducing single and dual RGN complexes had a higher proportion of disrupted *CCR*5 alleles when compared to those subjected to the SSB-inducing Cas9^D10A^:gRNA^R5.1^ complex (Fig. 6e). These results confirm that in trans paired nicking can achieve programmable nuclease-assisted genome editing without concomitantly introducing a high mutagenic load into target cell populations.

## Discussion

In this study, we have demonstrated that in trans paired nicking based on combining RGN “nickases” with RGN-targetable donors can trigger robust and seamless chromosomal insertion of small and large genetic payloads into specific genomic sequences in human cells without the catalytic induction of DSBs. We speculate that the rate-limiting HR steps of single-stranded DNA invasion, donor–acceptor synaptic formation and heteroduplex expansion are, to a great extent, overcome by coordinated presentation of 3′ termini on both interacting partners after in trans paired nicking. These events are shared by recent working models invoking SSBs as recombination-initiating substrates^30^. In addition, recent experiments indicate the involvement of distinct factors underlying canonical and SSB-induced HR pathways. For instance, recombination between donor DNA and a nicked target sequence can proceed through RAD51/BRAC2-independent pathways^30^. In this regard, the versatility of RGNs for inducing nicks at different positions and strands of HR templates, might constitute a valuable experimental system to dissect SSB-dependent HR pathways and, possibly, further improve genome editing based on in trans paired nicking concepts.

Importantly, we also showed that avoiding the use of DSB-inducing nucleases confers a low mutagenic load to this new genome-editing paradigm. Hence, our research complements and joins those of others on devising high-efficiency genome-editing strategies based on RGN “nickases”^31, 32^. In particular, a recent study has demonstrated that fusing cytidine deaminase and uracil DNA glycosylase activities to Cas9^D10A^ results in a large “base editor” capable of inducing C→T substitutions within a ∼5 nt target window^31^. Another recent study revealed that cleaving and nicking RGNs expose a DNA flap accessible to single-stranded oligodeoxyribonucleotide (ssODN) annealing^32^. On the basis of this information, rationally designed ssODNs and RGN “nickases” were combined and shown to yield homology-directed gene repair in ∼10% of treated 293 reporter cells^32^. An intrinsic limitation of these approaches is, however, their unsuitability for effecting extensive genetic changes. Moreover, the fidelity of “base editors” depends on the absence of extra cytidines within the ∼5 nt “activity window”, while that of coupling RGN “nickases” to ssODNs relies on the lack of adventitious mutations created during synthesis and processing of ssODNs in vitro and in cellula, respectively^33^.

The high specificity and accuracy conferred by in trans paired nicking genome editing coupled to its low mutagenic load should be particularly useful in instances in which the precise genetic manipulation of target cell populations is paramount. Examples include the modeling or the repairing of disease traits in stem/progenitor cells and the unbiased genetic screening of cellular phenotypes based on HR-mediated chromosomal insertion of donor DNA libraries^34^. Of note, however, regardless of the DNA targeting specificity and fidelity attained by a particular genome-editing procedure, there is always the risk for uncontrollable random chromosomal insertion of the exogenous DNA. Clearly, these unwanted events can take place in cells that lack or harbor the intended genetic modification.

We have confirmed that nicking RGNs are significantly less mutagenic than their cleaving counterparts at on-target sequences (Supplementary Fig. 1). Moreover, experiments done by others have demonstrated that, when compared to cleaving RGNs, nicking RGNs are also significantly less mutagenic at off-target sites^25^. However, regarding the use of “nickases” specifically, one should caution that SSBs can still trigger some mutagenic events if, for instance, after hitting such lesions, an advancing replication fork collapses resulting in DSB formation^5^. These outcomes will be most problematic at off-target sites. In this regard, sensitive and unbiased assays allowing the genome-wide detection of nick-induced mutagenesis will be instrumental in the future for determining the mutagenic load of gene-editing protocols based on programmable “nickases”. Equally related with off-target activities, programmable “nickases” with improved specificities are in demand. Possible candidates include RGN “nickases” built on recently described high-specificity Cas9 scaffolds such as Sp Cas9-HF1^35^ and eSpCas9(1.1)^36^. We anticipate that the simple and versatile in trans paired nicking procedure will be compatible with these latest generation tools and, possibly, with other fast-emerging DNA targeting systems.

Concluding, the performance of genome editing depends on its overall efficiency, specificity and fidelity^1^. In this work, we have shown that testing combinatorial interactions between different types of nucleases and foreign DNA structures, can improve these crucial parameters, expanding the options for high-fidelity genetic manipulation of mammalian cells.

## Methods

### Cells

Human cervix carcinoma HeLa cells (American Type Culture Collection) and its *EGFP* expressing single cell-derived clone H27^37^ were cultured in Dulbecco’s modified Eagle’s medium (DMEM; ThermoFisher Scientific) containing 5% fetal bovine serum (FBS; ThermoFisher Scientific). Human embryonic kidney (HEK) 293 T cells (American Type Culture Collection) were maintained in DMEM supplemented with 10% FBS. These cells were kept at 37 °C in an humidified-air 10% CO_2_ atmosphere. The human embryonic stem cell (ESC) line H1 (ref. ^26^; WiCell Research Institute) and the induced pluripotent stem cell (iPSC) lines LUMC0044iCtrl44 and LUMC0044iCtrl44.9 were cultured in pluripotent stem cell (PSC) growth medium in the presence of irradiated ICR mouse embryonic fibroblasts (MEFs), and mechanically passaged by a cut and paste method. The PSC growth medium consisted of DMEM/F12 medium with GlutaMax, 20% KnockOut Serum Replacement (KOSR), 10 mM non-essential amino acids (NEAAs), 25 U ml^−1^ penicillin, 25 μg ml^−1^ of streptomycin (all from ThermoFisher Scientific), and 10 ng ml^−1^ of basic fibroblast growth factor (bFGF; Peprotech). PSCs were cultured at 37 °C in an humidified-air 5% CO_2_ atmosphere. The cells used in all the experiments were mycoplasma-free. All human materials were collected based on individual written (parental) informed consent after approval by the “Medical Ethics Committee” of the LUMC (reference numbers: P08-087 and P13-080). The experiments involving human materials were done in accordance with the principles outlined in the “Declaration of Helsinki”. All animal experiments were approved by the “Animal Experiments Committee” of the LUMC (reference number: 12133) and were performed following the recommendations and guidelines set by the LUMC and the Dutch “Experiments on Animals Act”.

### Recombinant DNA

The constructs AU26_pCAG.Cas9 and AU28_pCAG.Cas9^D10A^ express Cas9 and Cas9^D10A^, respectively, from the hybrid CAGGS promoter^38^. The “two-in-one” plasmids AV15_pCAG.Cas9.gRNA^S1^ and AV44_pCAG.Cas9^D10A^.gRNA^S1^ encode Cas9 and Cas9^D10A^, respectively, together with the *AAVS1*-targeting gRNA^S1^. To serve as a negative control, construct AV13_pCas9.gRNA^NT^ expresses Cas9 and the non-targeting gRNA^NT^. This gRNA is irrelevant in human cells as it addresses Cas9 proteins to the recognition sequence of the *S. cerevisiae* I-*Sce*I homing endonuclease. The annotated maps and full-length nucleotide sequences of AU26_pCAG.Cas9, AU28_pCAG.Cas9^D10A^, AV15_pCAG.Cas9.gRNA^S1^, AV44_pCAG.Cas9^D10A^.gRNA^S1^, AV13_pCas9.gRNA^NT^, and AT61_pCas9^H840A^ can be found in the Supplementary Figs. 17–22, respectively). Likewise for the *DMD*-targeting donor plasmids AL05_pDonor^DMD^ (Addgene #100284), AL62_pDonor^DMD.TS^ (Addgene #100287), AC62_pDonor^DMD.TS.DR^ (Addgene #100288), and AZ28_pDonor^DMD.TS.IR^ (Supplementary Figs. 23–26, respectively), the *AAVS1*-targeting donor constructs AX44_pS.Donor^S1^ (Addgene #100289; Supplementary Fig. 27), AX53_pS.Donor^S1.TS^ (Addgene #100290; Supplementary Fig. 28) and the *CCR5*-targeting donor plasmids AY42_pS.Donor^R5^ (Addgene #100291; Supplementary Fig. 29) and AY10_pS.Donor^R5.TS^ (Addgene #100292; Supplementary Fig. 30). The plasmids hCas9 (ref. ^39^; #41815) and hCas9_D10A^39^ (#41816), herein named pCas9 and pCas9^D10A^, respectively, were obtained from the Addgene repository. The constructs gRNA_Cloning Vector^39^ (#41824), gRNA_AAVS1-T2 (ref. ^39^; #41818), and gRNA_GFP_T2 (ref. ^39^; #41820), herein called pgRNA^Empty^, pgRNA^S1^, and pgRNA^GFP1^, respectively, were also acquired from Addgene. The plasmid pgRNA^Empty^ expresses no gRNA, whereas pgRNA^S1^ and pgRNA^GFP1^ express gRNAs addressing Cas9 proteins to *AAVS1* and *EGFP* sequences, respectively. The gRNA expressing plasmids AL08_pgRNA^DMD^ (Addgene #100293), AD19_pgRNA^S1.2^, AD13_pgRNA^S1.3^, L06_pgRNA^OUT.1^, AA44_pgRNA^OUT.2^, X32_pgRNA^IN.1^, AA48_pgRNA^IN.2^, AY22_pgRNA^R5.1^ (Addgene #100294), and AY23_pgRNA^R5.2^ (Addgene #100295), were assembled by inserting the annealed oligonucleotides described in the Supplementary Table 1 into the *Bve*I-digested gRNA acceptor construct S7_pUC.U6.sgRNA.BveI-stuffer^40^. The plasmids AM51_pUC.U6.gRNA^NT^, herein called pgRNA^NT^ and Z46_pgRNA^GFP2^ encoding an irrelevant gRNA and an *EGFP*-specific gRNA, respectively, have been described before^40^. The *AAVS1*-targeting donor constructs pSh.AAVS1.eGFP and pAdV.donor^S1/T-TS^, herein named pDonor.E^S1^ and pDonor.E^S1.TS^, respectively, have been described elsewhere^6, 41^. The additional set of isogenic donor plasmids pDonor^S1^, pDonor^S1.1×TS^, and pDonor^S1.TS^ contain the *AAVS1*-targeting module cloned in the pMOLUC vector backbone (Addgene #12514). The pDonor^S1^ plasmid has no gRNA^S1^ target sites, whereas pDonor^S1.1×TS^ and pDonor^S1.TS^ have one and two gRNA^S1^ target sites, respectively, next to their *AAVS1*-targeting module. The AX35_pDonor.Turq^S1^ and AX28_pDonor.Turq^S1.TS^ have the same composition of pDonor^S1^ and pDonor^S1.TS^ except that they contain a *mTurquoise2* ORF in place of that of *EGFP*. The AT58_pDonor.37^S1^ and AE32_pDonor.37^S1.1×TS^ share the same EGFP-encoding expression unit present in pDonor^S1^ and pDonor^S1.1xTS^, respectively. However, they differ from pDonor^S1^ and pDonor^S1.1xTS^ in the spacing between their regions of homology (Supplementary Fig. 5a, b). The final set of *AAVS1*-targeting donor plasmids AV11_pDonor.EP^S1^ (Addgene #100296) and AV09_ pDonor.EP^S1.TS^ (Addgene #100297) have the same composition of pDonor^S1^ and pDonor^S1.TS^, respectively, except that they encode Puro^R^.T2A.EGFP in place of EGFP (Supplementary Fig. 31). The annotated maps and DNA sequences of the constructs generated for this study were assembled with the aid of SnapGene 3.3.4. Where indicated, plasmid pcDNA3.1 (ThermoFisher Scientific) was used as carrier DNA in transfection experiments.

### Cell Transfections

One day before transfection, HeLa cells, H27 cells, and 293 T cells were seeded in wells of 24-well plates (Greiner Bio-One; Supplementary Tables 2–20). The transfections were initiated by mixing each of the appropriate plasmids together with 1 mg ml^−1^ of polyethyleneimine (PEI, Polysciences) in 50 μl of a 150 mM NaCl solution (Merck). After ~10 s under vigorous vortexing, the transfection mixtures were incubated for 15 min at room temperature, after which they were directly added to the cell cultures (Supplementary Tables 2–20). At 3 days post-transfection, the transfection efficiencies were determined by EGFP-directed flow cytometry. Subsequently, the cells were sub-cultured for at least 2 weeks, for the removal of episomal exogenous DNA, after which stable transfection levels were determined by EGFP-directed flow cytometry.

Prior to the transfection of PSCs, the cells were adapted to passaging as single cells by using TrypLE Select (ThermoFisher Scientific) and 10 µM of the Rho kinase inhibitor Fasudil (LC Laboratories). After 2 to 5 single-cell passages, transfections based on Lipofectamine (ThermoFisher Scientific) were initiated using different experimental conditions as detailed in Supplementary Tables 21 and 22. In general, single-cell suspensions were generated and incubated for 5–10 min in Opti-MEM (ThermoFisher Scientific) containing the relevant DNA mixtures and a specific Lipofectamine formulation (Supplementary Tables 21 and 22). Next, the cell suspensions were seeded on 1 day-old MEF cultures containing PSC growth medium supplemented with 10 µM Fasudil and lacking antibiotics. At 24 h post-transfection the medium was replaced by complete PSC growth medium. After 3–4 days post-transfection, the PSCs were harvested and the transfection efficiencies were determined by EGFP-directed and TRA-1-81-directed flow cytometry. A fraction of the transfected PSCs were seeded and let to divide on MEF cultures for an additional period of 7 to 10 days, after which stable transfection levels were determined by EGFP-directed and TRA-1-81-directed flow cytometry (see below for details).

### Adenoviral vectors

The production, purification and titration of adenoviral vector particles AdV^Δ2^P.Cas9.F^50^ and AdV^Δ2^U6.gRNA^S1^.F^50^, herein dubbed AdV.Cas9 and AdV.gRNA^S1^, respectively, have been specified before^42^. The same methods were applied to generate and characterize AdV.Cas9^D10A^ particles. The genome of AdV.Cas9^D10A^ differs from that of AdV.Cas9 exclusively at codon 10 of the *Cas9* ORF.

### T7 endonuclease I-based genotyping assay

Genotyping assays based on the detection of indels by the mismatch-sensing T7 endonuclease I (T7EI) were used for establishing targeted DSB formation activity at *AAVS1*. To this end, 293 T cells were transfected using PEI as indicated in Supplementary Table 2. At 3 days post-transfection, cell pellets were collected for subsequent genomic DNA extraction. Genomic DNA was extracted by using the DNeasy Blood & Tissue kit (Qiagen) according to the manufacturer’s instructions. The target region was amplified by PCR with GoTaq G2 Flexi DNA Polymerase (Promega) following the manufacture’s recommendations. The composition of the PCR mixtures and the PCR cycling parameters are specified in Supplementary Tables 23 and 24, respectively. Next, the resulting 403 bp amplicons were subjected to T7EI assays^42^. In brief, PCR amplicons were first denatured and reannealed by applying the thermocycler program presented in Supplementary Table 25. Afterwards, 10-μl samples were incubated at 37 °C for 17 min in a 15-μl solution containing 1.5 μl of 10 × NEBuffer 2, 0.5 μl of 10 U μl^−1^ T7EI (New England Biolabs) and Milli-Q water. After agarose gel electrophoresis, the DNA fragments were stained with ethidium bromide and were imaged by using a Molecular Imager Gel-Doc™ XR+ apparatus together with the ImageLab 4.1 software (both from Bio-Rad).

### Detection of DSBs after AdV-mediate delivery of RGNs

Hela cells were seeded in wells of 24-well plates at a concentration of 6 × 10^4^ cells per well. The next day, they were transduced with different amounts and combinations of adenoviral vectors encoding Cas9, Cas9^D10A^, or gRNA^S1^ (Supplementary Fig. 1). Mock-transduced cells and cells transduced exclusively with each of these vectors alone served as negative controls. Transduction experiments were carried out in duplicate to generate parallel samples for genotyping and western blot analysis. At 3 days post-transduction, genomic DNA was extracted and T7EI-based genotyping assays were performed as detailed under the previous section. Likewise, at 3 days post-transduction, protein lysates were prepared under ice-cold conditions for western blot analysis as follows. The cells were first washed twice with phosphate-buffered saline (PBS; pH 7.4), after which 60 μl of lysis buffer consisting of 10 mM Tris-HCl (pH 7.5), 1% (v/v) Nonidet P-40, 150 mM NaCl, 0.1% sodium dodecyl sulphate(SDS), and 10 mg ml^−1^ sodium deoxycholate, was added onto the cells for 25 min under gentle plate tilting. The lysis buffer was supplemented with the protease inhibitors present in the cOmplete^TM^, Mini Protease Inhibitor Cocktail Roche-11836153001 (Sigma-Aldrich). Protein concentrations were determined by using the BCA protein assay kit (ThermoFisher Scientific) and a Precisely 1420 Multilabel Plate Counter (PerkinElmer) with the absorption wavelength set at *λ* *=* 545 nm. Next, 5-μg protein samples were diluted in bromophenol blue-containing loading buffer consisting of 187.5 mM Tris (pH 6.8), 30% (v/v) glycerol, 9% (w/v) SDS, and 7.5% (v/v) β-mercapthoethanol. Next, the protein samples were heated at 95 °C for 5 min and were resolved through a SDS-8% polyacrylamide gel. After overnight electro-blotting onto Immobilon-P membranes (Millipore), the subsequent blocking, antibody incubation and chemiluminescence protein detection steps, were performed essentially as detailed elsewhere^43^ using a monoclonal antibody specific for *S. pyogenes* Cas9 (Diagenode; clone 22B5) and a goat anti-mouse horseradish peroxidase-conjugated IgG (Santa Cruz; sc-2005). These primary and secondary antibodies were diluted 1:2000 and 1:10,000 in blocking buffer, respectively. To provide for an internal protein loading control, an antibody specific for the α/β tubulin heterodimer (Cell Signalling Technology; 2148) was combined with the aforementioned secondary antibody. These primary and secondary antibodies were diluted 1:5000 and 1:10,000 in blocking buffer, respectively.

### Sanger sequencing

The amplicons specific for translocation events between *AAVS1* and *DMD* sequences (Supplementary Fig. 1e) and for *AAVS1*-exogenous DNA junctions (Supplementary Fig. 8) were amplified, isolated and purified from agarose gel by using the JETquick Gel Extraction Spin kit (Genomed) according to the manufacturer’s recommendations. The PCR mixtures and cycling conditions used are presented in Supplementary Tables 23 and 24, respectively. Next, the recovered fragments were inserted into pJET1.2/blunt cloning vector provided in the CloneJET PCR Cloning Kit (ThermoFisher Scientific) following the manufacturer’s instructions. After transformation, randomly selected clones were grown and subjected to Sanger sequencing (Baseclear, Leiden, the Netherlands). The *AAVS1*-specific PCR products derived from randomly selected iPSC clones (Fig. 3f) were purified and subjected to Sanger sequencing for identifying indel footprints generated after RGN activity. All nucleotide sequence reads were aligned and analyzed with the aid of AlignX, Vector NTI Advance R_11.5.0 software. The Sanger sequencing chromatograms were generated by using the Chromas Lite 2.1.1 software (Technelysium Pty).

### Flow cytometry

The frequencies of cells expressing EGFP, mTurquoise2 and/or the TRA-1-81 antigen, characteristic of uncommitted PSCs, were determined by using a BD LSR II flow cytometer (BD Biosciences). The TRA-1-81 labeling was carried out by incubating single-cell suspensions of PSCs with a phycoerythrin-conjugated TRA-1-81 antibody (eBioscience) diluted 1:100 in a buffer consisting of PBS supplemented with 0.5% BSA and 2 mM ethylenediaminetetraacetic acid (EDTA). After an incubation period of 30 min at 4 °C in the dark, excess antibody was removed by thorough successive washes with large volumes of the aforementioned buffer. Data was analyzed with the aid FlowJo 7.2.2 software (Tree Star). Non-transfected cells were used to set background fluorescence levels. At least 10,000 events, each representing a single viable cell, were measured per sample.

### PCR analyses of gene-editing experiments

The composition of the PCR mixtures and thermocycling parameters used for the analyses of genome-modifying events are discriminated in the Supplementary Tables 23, 24, 26 and 27. These analyses were performed on whole target cell population as well as on individually sorted cells. The sorting of EGFP^+^ HeLa and 293 T cells was conducted by using a BD FACSAria III flow cytometer (BD Biosciences) after sub-culturing transfected cell populations for more than 20 days. EGFP^+^ cells were collected in a 1:1 mixture of regular culture medium containing 2 × penicillin–streptomycin (ThermoFisher Scientific) and FBS. The sorted, EGFP^+^ cells, were seeded at a density of 0.3 cells per well in wells of 96-well plates (Greiner Bio-One). To increase the efficiency of cell cloning, the culture media were supplemented with 50 μM α-thioglycerol and 20 nM bathocuprione disulphonate (both from Sigma-Aldrich)^44^. At ~3 weeks after seeding, single cell-derived clones were randomly collected for genomic DNA analysis by junction PCR with the Phire Tissue Direct PCR Master Mix (ThermoFisher Scientific) according to the manufacturer’s recommendations. The junction PCR screening of randomly selected puromycin-resistant iPSC colonies was also done by direct PCR. The various direct PCR conditions used in each experiment are specified in Supplementary Tables 26 and 27.

### Generation of iPSCs

Human fetal fibroblasts were isolated and reprogrammed to iPSCs as detailed elsewhere^45^. In brief, the cell reprogramming was induced by transducing 2 × 10^4^ human fetal fibroblasts seeded in a 12-well plate with the multi-cistronic lentiviral vector LV.RRL.PPT.SF.hOKSM.idTomato.-preFRT^45, 46^. The vector particles, encoding OCT3/4, KLF4, SOX2, and cMYC, were removed 24 h later. At 6 days post-transduction, the cells were harvested and 10^4^ of them were seeded on a 10-cm dish with 2 × 10^6^ MEFs cultured in KOSR PSC growth medium (ThermoFisher Scientific). The medium was replenished every other day until the appearance of ESC-like colonies. The resulting iPSCs were subsequently cultured on irradiated ICR MEFs in complete PSC growth medium. The iPSC clone LUMC0044iCtrl44, containing a single provirus, was selected. Subsequently, the chromosomally inserted provirus was removed through hcAd.FLPe.F50-mediated expression of *FLPe* recombinase^43^. To this end, LUMC0044iCtrl44 iPSCs were transduced with hcAd.FLPe.F50 at a multiplicity of infection (MOI) of 20 transduction units/ml. The resulting provirus-free iPSC clone LUMC0044iCtrl44.9 was characterized by immunofluorescence microscopy, COBRA-FISH karyotyping and teratoma assays as described below.

### Differentiation of iPSCs

The *in vitro* spontaneous differentiation of iPSCs into the three embryonic germ layers was triggered by culturing clumps of iPSCs on coverslips coated with Vitronectin XF (StemCell Technologies). The cells were incubated in PSC growth medium devoid of bFGF and containing 20% FBS in place of KOSR. The medium was replenished every 2 days. After 3 weeks under differentiation conditions, the cells were stained for the endoderm, mesoderm and ectoderm markers alpha-fetoprotein (AFP), platelet and endothelial cell adhesion molecule 1 (CD31), and tubulin beta 3 class III (TUBB3), respectively. The in vivo differentiation of iPSCs into cell types belonging to the three different embryonic germ layers was assessed through teratoma formation assays. To this end, immunodeficient female NOD.Cg-*Prkdc* ^*scid*^ *Il2rg* ^*tm1Wjl*^/SzJ (NSG) mice (8–12 weeks old) were injected subcutaneously with 1 × 10^6^ cells. After 10–16 weeks post-injection, teratomas were isolated and fixed in 4% paraformaldehyde (PFA) and, subsequently, embedded in paraffin and sectioned. Finally, pigmented epithelium (ectoderm), trachea-like epithelium (endoderm), and cartilage (mesoderm) present in iPSC-derived teratomas were identified by hematoxylin–phloxine–saffron (HPS) staining and standard visible light microscopy.

### Detection of apoptosis

Two days after transfecting H1 ESCs with Lipofectamine 3000™ (ThermoFisher Scientific) mixed with different construct combinations (Supplementary Table 22), the frequencies of apoptotic cells were measured by using Annexin-V Apoptosis Detection Kit-eFluro 450 according to the manufacturer’s instructions (ThermoFisher Scientific). H1 ESC cultures treated for 2 h with 1 μg ml^−1^ of staurosporine, stained, or not stained with fluorochrome-conjugated Annexin V, served as positive and negative controls, respectively.

### Immunofluorescence microscopy

The acquisition of pluripotency markers (i.e., TRA-1-81, SSEA4, OCT3/4, and NANOG) and differentiation markers (i.e., AFP, CD31, and TUBB3) by iPSCs and iPSC-derived cells, respectively, was assessed via immunofluorescence staining. In brief, cells were first fixed for 15 min in 4% PFA. After several washes with PBS, they were exposed for 1 h to a PBS solution containing 0.1% Triton X-100 and 4% normal swine serum (NSS; Jackson ImmunoResearch). Next, the cells were incubated overnight at 4 °C with primary antibodies directed against TRA-1-81 (1:125; Biolegend Cat. #330702), SSEA4 (1:30; Biolegend Cat. #330402), OCT3/4 (1:100; Santa Cruz Biotechnology Cat. #sc-5279); NANOG (1:500; R&D Systems Cat. #963488); AFP (1:25; Quartett Cat. #2011200530), CD31 (1:100; Dako Cat. #M0823), and TUBB3 (1:4000; Covance Cat. #MMS-435P). These antibodies were diluted in PBS containing 4% NSS. After three 10-min washes with PBS containing 0.05% Tween-20, the specimens were incubated for 1 h in the dark with secondary antibodies (ThermoFisher Scientific) conjugated with Alexa Fluor 488 (1:500) or with Alexa Fluor 568. For the detection of pluripotency and differentiation markers, the Alexa Fluor 568 secondary antibody was applied at 1:200 and 1:500 dilutions, respectively. Nuclei were stained for 5 min in the dark with 4′,6-diamidino-2-phenylindole, dihydrochloride (DAPI; ThermoFisher Scientific). The DAPI was diluted 1:1000 and 1:200 in PBS for the detection of nuclei of pluripotent and differentiated cells, respectively. Finally, the specimens were rinsed with sterile Milli-Q water (Millipore) and were mounted with Mowiol (Calbiochem). The in vitro PSC differentiation analyses were performed with an IX51 inverse fluorescence microscope equipped with a XC30 Peltier-cooled digital color camera (Olympus) or a Confocal laser scanning microscope TCS SP8 (Leica). The images were processed with the aid of Cell^F^ 3.4 imaging software (Olympus) or LAS AF software (Leica).

### COBRA-FISH karyotyping

Combined binary ratio labeling (COBRA)-FISH analysis was carried out for determining the karyotype of iPSCs essentially following the instructions indicated in a previously published protocol^47^. In short, slides with metaphase chromosomes from iPSCs were pre-treated with RNase I (Roche; 100 μg ml^−1^ in 2 × SSC) and pepsin (Sigma-Aldrich; 0.005% in 0.01 M HCl) at 37 °C for 10 min and 5 min, respectively. After a 10-min fixation with 1% (v/v) formaldehyde in PBS, the specimens were dehydrated by sequential 3-min incubations in 70%, 90% and 100% ethanol. Next, whole-chromosome painting probes, labeled with the fluorescent dyes diethylaminocoumarin, Cy3, Cy5, and rhodamine green using the Universal Linkage System (ULS) kit (Kreatech Biotechnology), were applied to the air-dried slides. After a denaturation step at 80 °C for 45–90 s, DNA hybridizations were let to proceed in a humidified chamber at 37 °C for 2 days. The unbound probes were removed by a series of post-hybridization washes and the samples were subsequently dehydrated by exposing them to increasing concentrations of ethanol as aforementioned. Finally, the chromosome specimens were sealed and counterstained with Citifluor AF1 mounting solution (Citifluor Ltd.) containing 500 ng ml^−1^ of the DNA dye DAPI. Digital images were acquired with the aid of a Leica DMRA fluorescence microscope coupled to a CCD camera.

### Colony-formation assays

In addition to flow cytometric analysis, stable transfections resulting from gene targeting experiments with pDonor.EP^S1^ and pDonor.EP^S1.TS^ were also assessed by colony-formation assays. These assays were applied to puromycin-resistant HeLa and PSC colonies by using, respectively, a standard Giemsa staining and the leukocyte AP kit for detecting alkaline phosphatase (AP) activity (Sigma-Aldrich). These colonies were derived from cell cultures initially exposed to pDonor.EP^S1^ and pCAG.Cas9.gRNA^S1^ (standard setting) or to pDonor.EP^S1.TS^ and pCAG.Cas9^D10A^.gRNA^S1^ (Nick^2^ setting). In brief, at 20 days post-transfection, HeLa cells were seeded at densities of 1 × 10^3^ and 1 × 10^4^ cells per 10-cm dish (Greiner Bio-One) in the presence of 1 μg ml^−1^ of puromycin. Puromycin-resistant HeLa cell colonies were identified by Giemsa staining 9 days later. Next to this, at 3–4 days post-transfection PSCs were seeded at a density of 2–3 × 10^5^ cells per MEF culture and, 1 day later, were exposed to puromycin at a final concentration of 1 μg ml^−1^. After 5 days under puromycin selection was assessed by using the aforementioned AP detection kit following the manufacturer’s instructions.

### Southern blot analysis

Genomic DNA was extracted from individual EGFP^+^/mTurquoise2^+^ HeLa cell clones and from a control EGFP^+^ HeLa cell clone according to a standard organic solvent-based protocol as follows. The cells were collected and incubated overnight at 50 °C in 250 μl of lysis buffer containing 10 mM Tris-HCl (pH 8), 25 mM EDTA (pH 8), 0.5% (w/v) SDS, and 100 mM NaCl. Prior to use, the lysis buffer was supplemented with freshly added proteinase K (ThermoFisher Scientific) at a final concentration of 0.1 μg μl^−1^. The resulting cell lysates were extracted twice by gentle pipetting in a 1:1 mixture with buffer-saturated phenol:chloroform:isoamyl alcohol (25:24:1). The aqueous phase was recovered and was subsequently subjected to one additional extraction cycle by gentle pipetting in a 1:1 mixture with chloroform. Next, the chromosomal DNA present in the aqueous phase was precipitated in 2.0 and 0.5 volumes of ethanol and 7.5 M ammonium acetate (pH 5.5), respectively. The recovered genomic DNA pellets were washed with 70% (v/v) ethanol, gently air-dried, and were finally dissolved in DNase-free sterile water at a concentration of 1–2 μg μl^−1^. Subsequently, DNA samples (10 μg each) were digested overnight with BlpI (New England BioLabs) and were resolved through a 1.0% agarose gel in 1 × Tris-acetate-EDTA buffer. The DNA was transferred by capillary action onto an Amersham Hybond-XL membrane (GE Healthcare Life Sciences) using an alkaline transfer buffer consisting of 0.4 N NaOH and 1 M NaCl. After overnight transfer, the membrane was neutralized with a pH 7.2 solution containing 0.5 M Tris-HCl and 1 M NaCl. The *EGFP*-specific and *mTurquoise2*-specific probes (994 bp each) were isolated from agarose gel after *Age*I/*Hind*III double digestion of AA63_pDonor^S1^ and AX28_pDonor.Turq^S1^, respectively. The purified DNA probes were radiolabeled with [α-^32^P]dATP (GE Healthcare Life Sciences) by using the DecaLabel DNA labeling Kit following the manufacturer’s instructions (ThermoFisher Scientific). The Pre-hybridization and hybridization steps, 2 h and overnight, respectively, were performed at 65 °C in Rapid-Hyb Buffer (GE Healthcare). Next, the membrane was washed at 65 °C once with a 2 × SSC solution supplemented with 0.1% (w/v) SDS (20 min) and twice with a 0.5 × SSC solution supplemented with 0.1% (w/v) SDS (20 min each). Finally, the membrane was gently air-dried, wrapped in Saran film and exposed to an Amersham Hyperfilm MP (GE Healthcare). The autoradiogram film was obtained by using standard developing solutions.

### Statistical analyses

The researchers were not blinded to sample allocation during experiments and data analyses. One-way ANOVA combined with Bonferroni tests were used for the statistical analyses of data sets obtained from three independent experiments comparing the performance of donors with no, one, or two gRNA target sites (*P* < 0.05 was considered significant). The IBM SPSS Statistics 23 software package was employed for these analyses. The comparison of data sets retrieved from standard and in trans paired nicking gene targeting experiments was performed by applying two-tailed Student’s *t*-tests (*P* < 0.05 was considered significant). The GraphPad Prism 6 software package was used for these analyses.

### Restriction-fragment length analyses

Amplicons spanning the *AAVS1* and *CCR5* target sites, and the BlpI polymorphism in the *mTurquoise2* coding sequence were generated with the PCR reagents and subsequently exposed to the restriction enzymes specified in Supplementary Table 28. The corresponding primers and PCR cycling conditions are indicated in Supplementary Tables 23, 24, 26, and 27.

### Data availability

All relevant results generated in this study are available within the paper and respective Supplementary Information or are available from the corresponding author on reasonable request. Sanger sequencing chromatograms are deposited in FigShare at 10.6084/m9.figshare.5208766.

## Electronic supplementary material

Supplementary Information

Peer Review File
