# Supplementary material for: In trans paired nicking triggers seamless genome editing without double-stranded DNA cutting
Source: Nat Commun. 2017 Sep 22;8:657. doi: 10.1038/s41467-017-00687-1 (PMC5610252; doi:10.1038/s41467-017-00687-1)
Supplement: Supplementary file 1 — Supplementary Information [file 41467_2017_687_MOESM1_ESM.pdf]

Description of Supplementary Files

Title: Supplementary Information

Description: Supplementary Figures and Supplementary Tables

Title: Peer Review File

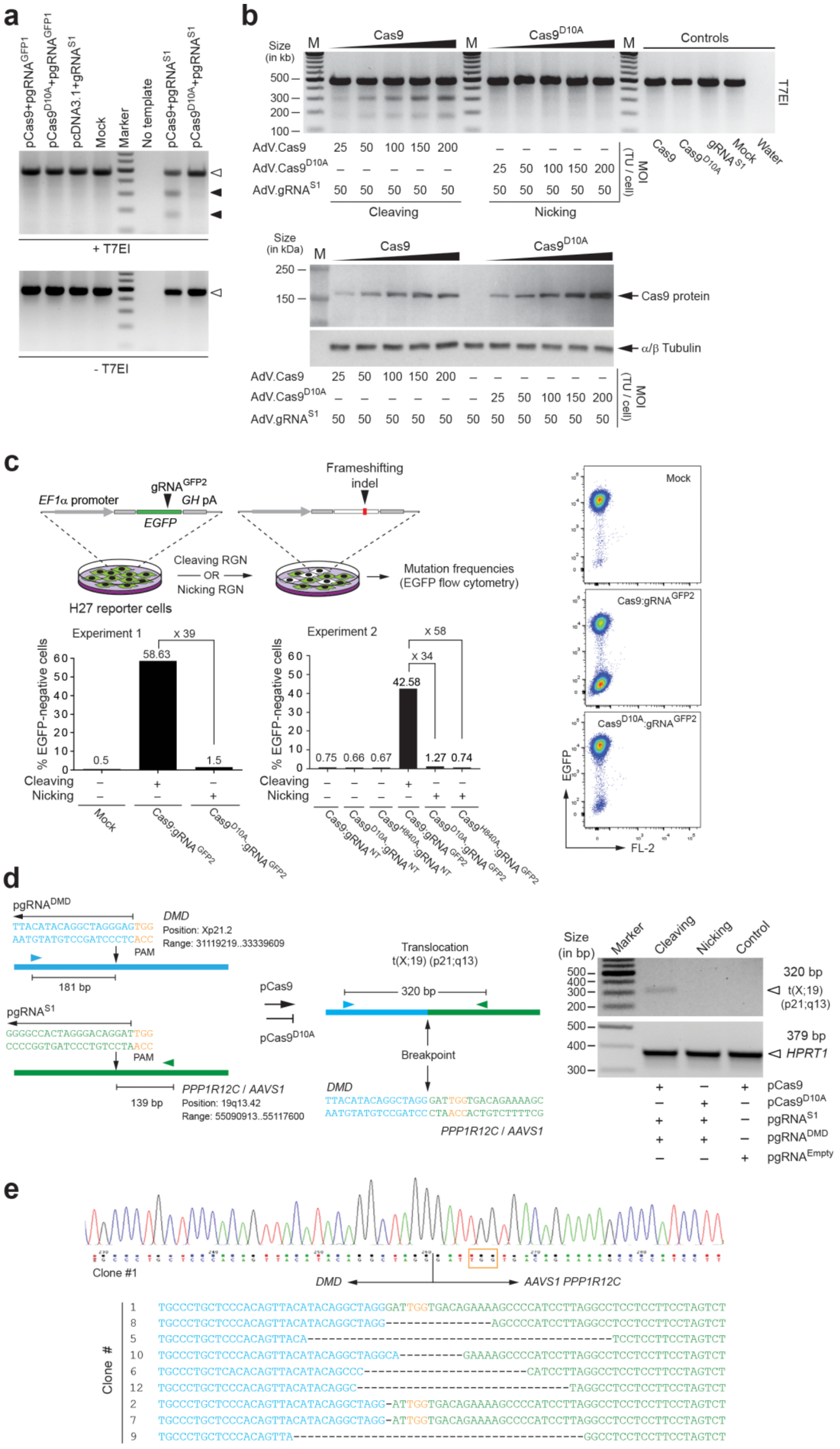

Supplementary Figure 1. Comparing the generation of allelic mutations and translocations in human cells provided with cleaving and nicking RGNs. (**a**) Detection of

target gene mutagenesis induced by RGNs with Cas9 and Cas9^{D10A}. PCR products spanning an *AAVS1* target sequence retrieved from 293T cells transfected with the indicated plasmids were exposed to the mismatch-sensing T7EI. Indels formed by NHEJ-mediated DSB repair were detected by the appearance of T7EI-digested products (solid arrowheads). Experimental controls consisted of introducing only the *AAVS1*-targeting gRNA^{S1} or each nuclease together with the *EGFP*-specific gRNA^{GFP1}. Assay controls were provided by PCR samples containing DNA from mock-transfected cells or lacking either template DNA or T7EI. (**b**) Indel detection in cells exposed to increasing concentrations of cleaving Cas9 or nicking Cas9^{D10A}. HeLa cells were transduced in duplicate with the indicated doses and combinations of adenoviral vectors AdV.Cas9, AdV.Cas9^{D10A} and AdV.gRNA^{S1}. At 3 days post-transduction, indels and nucleases were detected by using T7EI-based genotyping assays (top panel) and western blot analysis (bottom panel), respectively. MOI, multiplicity-of-infection; TU/cell, transducing units per cell; Tubulin, protein loading control. (**c**) Gene knockout levels induced by RGNs containing Cas9, Cas9^{D10A}, or Cas9^{H840A}. The schematics of the experimental system (top) and the quantification of gene disruption frequencies (bottom) are presented. EGFP^{+} H27 cells were either mock-transfected or were co-transfected with the indicated plasmids. The gRNA^{GFP2} and gRNA^{NT} moieties address Cas9 proteins to, respectively, *EGFP* and an irrelevant I-SceI recognition sequence. The mutation rates caused by DSBs and SSBs were determined through flow cytometric quantification of EGFP-negative cells. The bars correspond to the mean values of two experiments done in duplicate. Representative dot plots are shown. (**d**) Probing for RGN-induced translocations. Genomic DNA from HeLa cells exposed to cleaving and nicking RGN pairs targeting *DMD* and *AAVS1* was subjected to the depicted PCR assay. Translocation-specific amplicons were detected using the indicated primer pair (arrowheads). *HPRT1* served as an internal control template for genomic DNA integrity. (**e**) Molecular characterization of translocation events. Sanger sequencing of cloned amplicons corresponding to individual chromosomal translocation events confirmed the presence of various t(X;19)(p21;q13) species in cell populations subjected to DSBs at *DMD* and *AAVS1*.

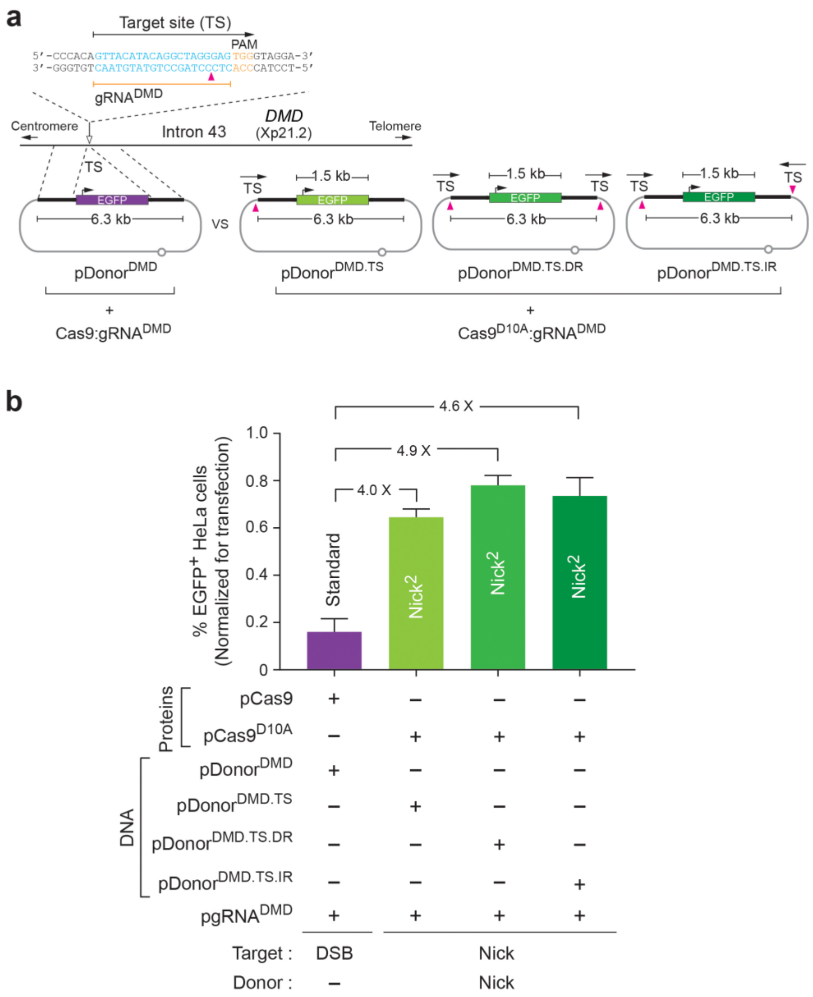

Supplementary Figure 2. Testing *in trans* paired nicking at *DMD* with donors containing different RGN target site arrangements. (**a**) Diagram of the standard and *in trans* paired nicking donor plasmids tested. Orange arrows define the orientations of the gRNA^{DMD} target site (TS). The plasmids pDonor$^{DMD.TS.DR}$ and pDonor$^{DMD.TS.IR}$ harbour the TS in a direct and inverted repeat configuration, respectively. Magenta arrowheads, position of sequence- and strand-specific DNA cleavage by Cas9^{D10A}. The transgene consists of the human *PGK1* promoter, the *EGFP* ORF and the bovine *GH1* polyadenylation signal. Cas9:gRNA^{DMD} and Cas9^{D10A}:gRNA^{DMD} are cleaving and nicking RGN complexes, respectively. PAM, protospacer adjacent motif; magenta arrowheads, position of sequence- and strand-specific DNA cleavage by Cas9^{D10A}. (**b**) Quantification of stable transfection levels by flow cytometry. Flow cytometric analysis of long-term HeLa cell cultures initially co-transfected with the indicated plasmids. The bars correspond to the mean ± s.d. of two independent experiments.

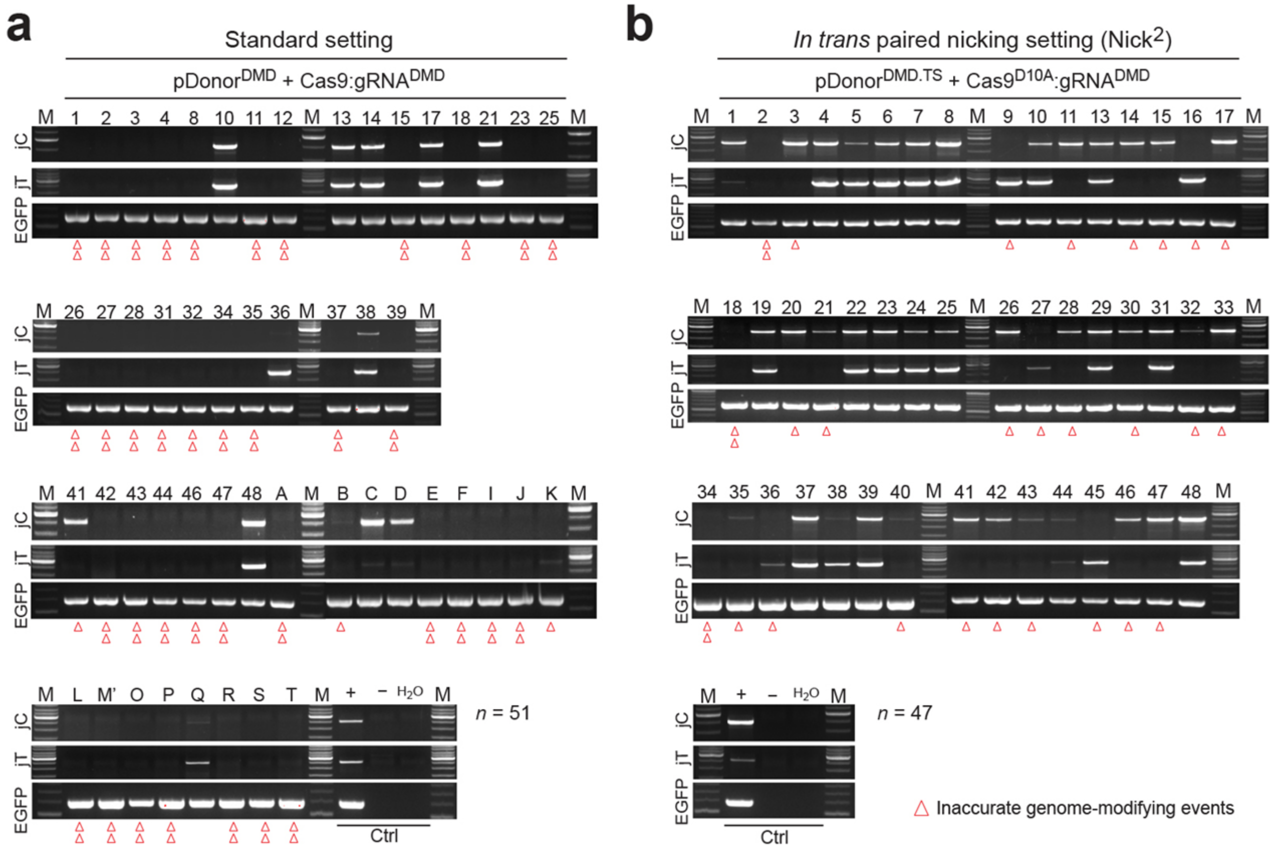

Supplementary Figure 3. Molecular characterization of human cells genetically modified by cleaving versus *in trans* paired nicking at *DMD*. (**a**) Screening of cells exposed to RGN-induced standard gene targeting. Junction PCR analyses of individual $EGFP^+$ HeLa cell clones isolated from cultures co-transfected with pCas9, $pgRNA^{DMD}$ and $pDonor^{DMD}$. (**b**) Screening of cells subjected to *in trans* paired nicking gene targeting ($Nick^2$). Junction PCR analyses of individual $EGFP^+$ HeLa cell clones derived from cultures co-transfected with $pCas9^{D10A}$, $pgRNA^{DMD}$ and $pDonor^{DMD.TS}$. The different PCR screenings were carried out with the primer pairs shown in **Fig. 1a**. The resulting amplicons are specific for HR-derived telomeric and centromeric junctions formed between native DMD and exogenous DNA (jT and jC, respectively). PCR mixtures with DNA from parental cells or with nuclease-free water served as negative controls. PCR amplifications targeting *EGFP* served as internal controls (EGFP). Lanes M, GeneRuler DNA Ladder Mix molecular weight marker.

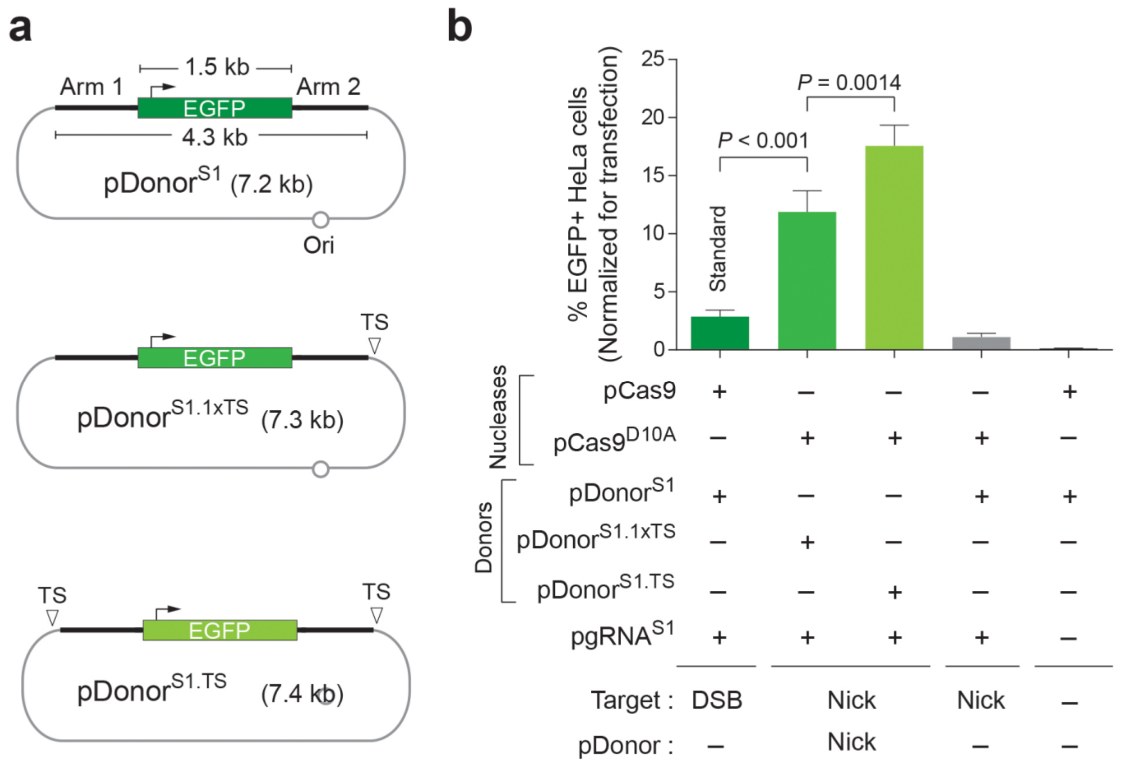

Supplementary Figure 4. Testing the performance of RGN-resistant versus RGN-susceptible donor plasmids. (**a**) Schematics of unmodified and modified *AAVS1*-targeting constructs. Arm 1 and Arm 2, DNA sequences sharing identity to those located "upstream" and "downstream", respectively, the target site of RGNs containing the gRNA^{S1} moiety. EGFP, transgene consisting human *PGK1* promoter, *EGFP* ORF and bovine *GH1* polyadenylation signal sequences. The constructs pDonor^{S1}, pDonor$^{S1.1\times TS}$ and pDonor$^{S1.TS}$ have zero, one and two target sites for *AAVS1*-specific RGN complexes (TS) located at the indicated positions. Ori, prokaryotic origin of replication. (**b**) Quantification of stable transfection levels by flow cytometry. EGFP-directed flow cytometry of HeLa cell populations transfected with the indicated plasmid combinations was done at 3 days and more than 2 weeks post-transfection, to determine transient and stable transfection levels, respectively. Ten thousand events, each corresponding to a single viable cell, were measured per sample. The bars represent mean ± s.d. of three independent experiments. The experimental groups were first compared by one-way ANOVA analysis. A subsequent statistical comparison between the indicated groups was carried out by Bonferroni analysis. The resulting P values are shown ($P < 0.05$ was considered significant).

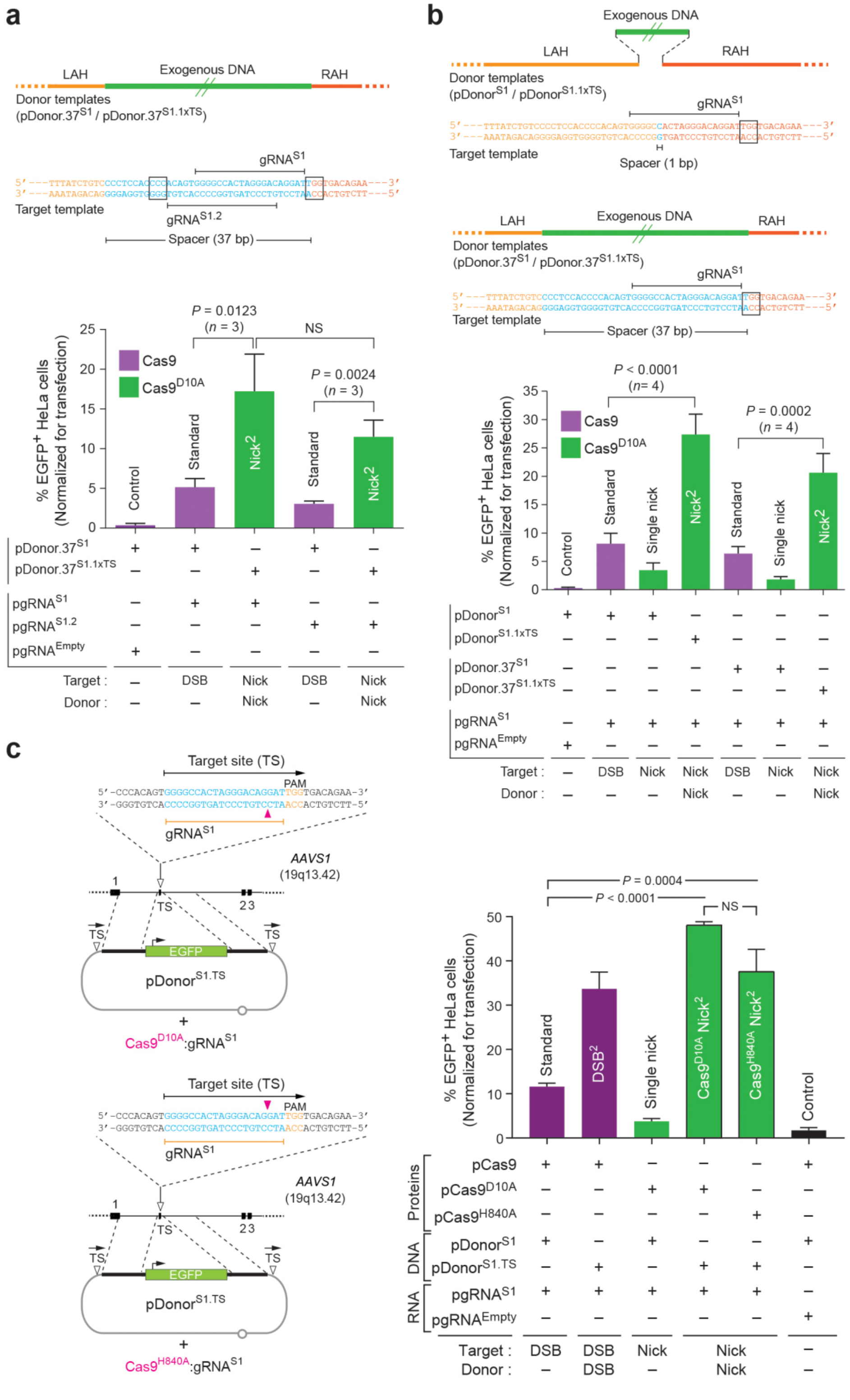

Supplementary Figure 5. Comparing the impact of different genome editing reagents target sequences and donor DNA compositions on *in trans* paired nicking. (**a**) Comparing standard versus *in trans* paired nicking using two different *AAVS1*-targeting RGN complexes.

Top panel, Schematics of RGN-resistant pDonor.37^{S1} and RGN-susceptible pDonor.37$^{S1.1xTS}$, drawn in relation to their target DNA. The gRNAs gRNA^{S1} and gRNA$^{S1.2}$ are also depicted in relation to their respective target sequences. Bottom panel, flow cytometric analysis of long-term HeLa cell cultures initially exposed to the indicated plasmid combinations. The bars correspond to mean ± s.d. of three independent experiments. *P* values were derived from two-tailed *t*-tests. (**b**) Comparing standard versus *in trans* paired nicking using *AAVS1*-targeting donors with different spacing between homology regions. Top panel, Schematics of donor DNA pairs pDonor^{S1}/pDonor$^{S1.1xTS}$ and pDonor.37^{S1}/pDonor.37$^{S1.1xTS}$ with 1-bp and 37-bp spacing between their arms of homology, respectively. LAH and RAH, "left" and "right" arms of homology, respectively. Bottom panel, flow cytometry of long-term HeLa cell cultures initially exposed to the indicated plasmid mixtures. The bars correspond to mean ± s.d. of four independent experiments. *P* values were obtained by applying two-tailed *t*-tests. (**c**) Comparing standard versus *in trans* paired nicking using different nicking Cas9 variants. The Cas9^{D10A} and Cas9^{H840A} proteins are sequence- and strand-specific endonucleases ("nickases") owing to having their RuvC and HNH catalytic domains disabled, respectively. Magenta arrowheads, position of sequence- and strand-specific DNA cleavage by Cas9^{D10A} or Cas9^{H840A}. RGNs harbouring the former and latter proteins nick the DNA chain complementary and non-complementary to the gRNA spacer sequence, respectively (left panel). Flow cytometric analysis of long-term HeLa cell populations initially co-transfected with the indicated constructs (right panel). Ten thousand events, each corresponding to a single viable cells, were acquired per sample. The bars represent mean ± s.e.m. of three biological replicates from two independent experiments. *P* values were determined by two-tailed *t*-tests ($P < 0.05$ was considered significant; NS, non-significant).

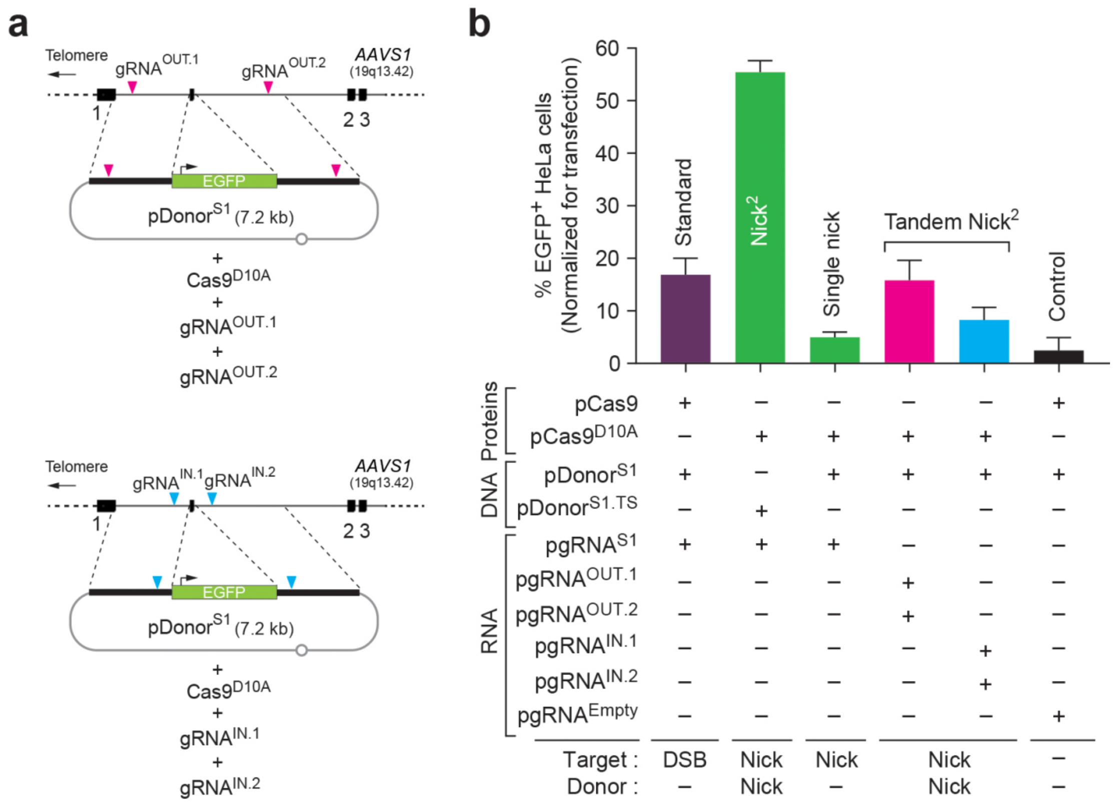

Supplementary Figure 6. Probing the tandem paired nicking concept. (a) Diagram of tandem paired nicking settings. The gRNAs used are drawn in relation to their target sequences located in *AAVS1* and in the "arms" of homology of donor plasmid pDonor ^{S1}. Magenta and cyan arrowheads indicate the target sites of outward and inward gRNA pairs gRNA$^{OUT.1}$/gRNA$^{OUT.2}$ and gRNA$^{IN.1}$/gRNA^{IN2}, respectively. (**b**) Comparing standard with SSB-dependent gene targeting strategies. Quantification of stable transfection levels by EGFP-directed flow cytometry of HeLa cell populations initially transfected with the indicated plasmid mixtures. The bars correspond to mean ± s.d. of three independent experiments.

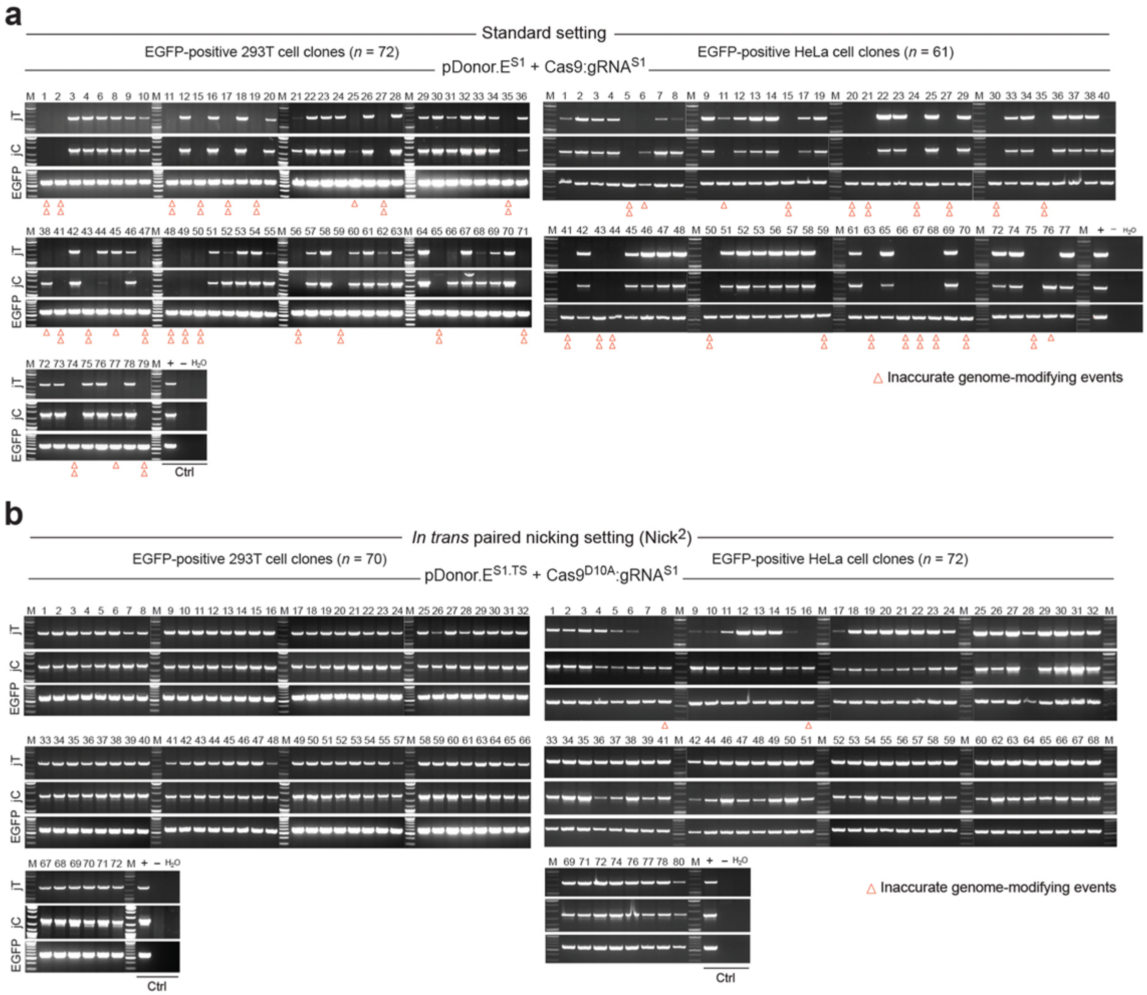

Supplementary Figure 7. Molecular characterization of human cells genetically modified by cleaving versus *in trans* paired nicking at *AAVS1*. (**a**) Screening of cells subjected to RGN-induced standard gene targeting. Junction PCR analyses of individual EGFP^{+} 293T and HeLa cell clones derived from cultures co-transfected with pCas9, pgRNA^{S1} and pDonor.E^{S1}. (**b**) Screening of cells exposed to *in trans* paired nicking gene targeting (Nick^{2}). PCR analyses of individual EGFP^{+} 293T and HeLa cell clones isolated from cultures co-transfected with pCas9^{D10A}, pgRNA^{S1} and pDonor.E$^{S1.TS}$. The various PCR screenings were performed with the primer pairs depicted in **Fig. 2a**. The resulting amplicons are specific for HR-derived telomeric and centromeric junctions involving native *AAVS1* and exogenous DNA (jT and jC, respectively). PCR mixtures containing DNA from parental cells or with nuclease-free water served as negative controls. PCR amplifications targeting *EGFP* provided for internal controls (EGFP). Lanes M, GeneRuler DNA Ladder Mix molecular weight marker.

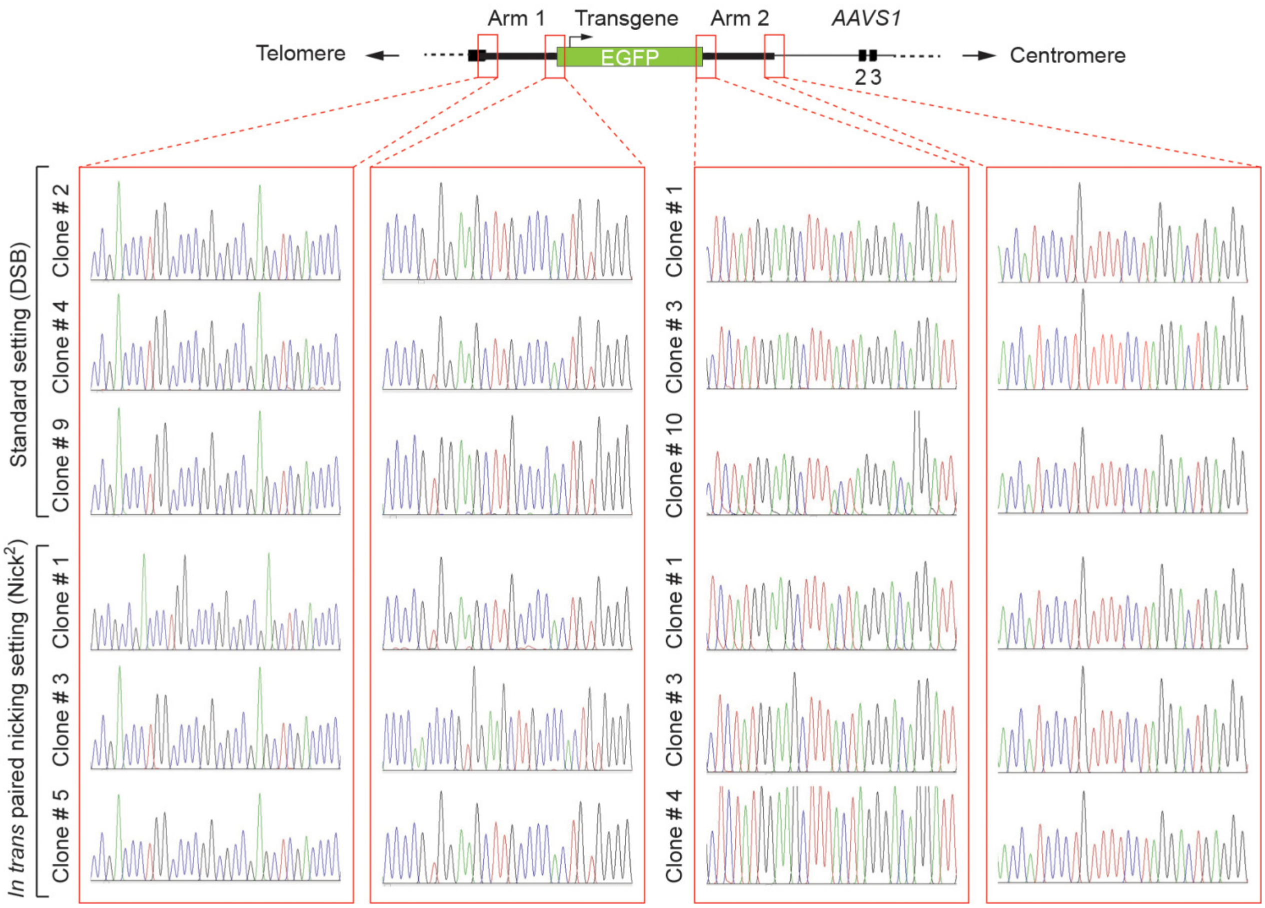

Supplementary Figure 8. DNA sequencing of transgenic-*AAVS1* junctions. Amplicons corresponding to telomeric and centromeric exogenous-endogenous DNA junctions formed in 293T cell populations initially co-transfected with pCas9, pgRNA^{S1} and pDonor (DSB) or with pCas9^{D10A}, pgRNA^{S1} and pDonor^{TS} (Nick^{2}), were cloned and sequenced. The chromatograms presented correspond to the border regions between the homology "arms" of donor templates and outward chromosomal DNA and between these "arms" and inward transgenic sequences. Six independent, randomly selected, molecular clones covering both integrant extremities were analysed per experimental condition. The sequence alignments detected neither large mutations (e.g. deletions, insertions or rearrangements) nor point mutations.

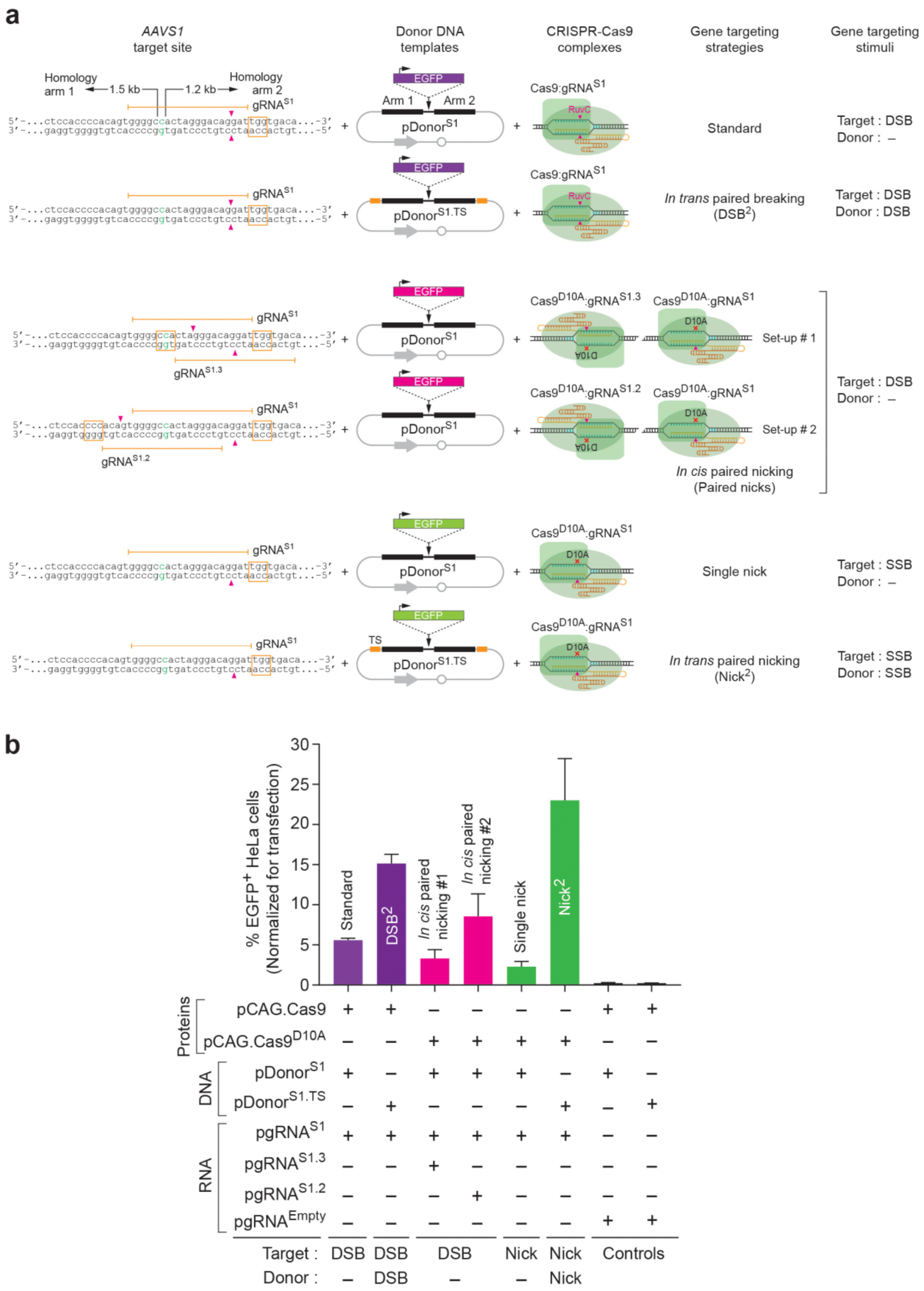

Supplementary Fig. 9. Comparing stable transfection levels achieved by using DSB- versus SSB-dependent gene targeting strategies. (**a**) Illustration of the different genome editing principles. Magenta arrowheads, position of sequence-specific DNA cleavage by Cas9 or Cas9^{D10A} (**b**) Quantification of stable transfection levels by flow cytometry. Flow cytometric analysis of long-term HeLa cell cultures initially co-transfected with the indicated plasmids. The bars correspond to the mean ± s.d. of two independent experiments.

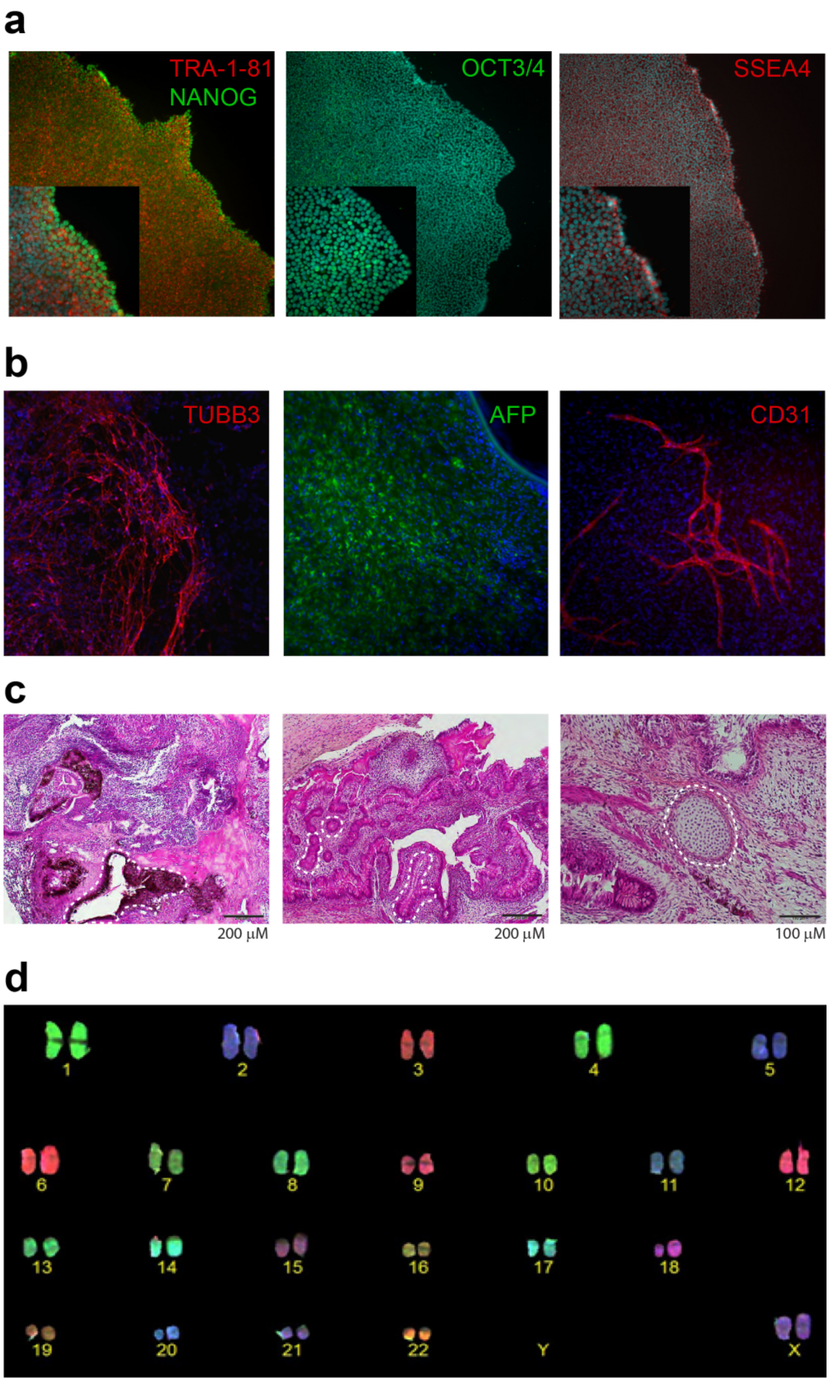

Supplementary Figure 10. Characterization of the iPSC line LUMC0044iCtrl44.9. (**a**) Immunofluorescence microscopy analysis of pluripotency markers in iPSCs. Undifferentiated iPSCs were stained for the pluripotent stem cell markers TRA-1-81, NANOG, OCT3/4 and SSEA4. (**b**) Analysis of *in vitro* differentiation of iPSCs by immunofluorescence microscopy. iPSCs were triggered to differentiate into cells belonging to the three different embryonic germ layers. Cells acquiring ectodermal, endodermal and mesodermal markers were

identified by staining with antibodies directed against tubulin beta 3 class III (TUBB3), alpha-fetoprotein (AFP) and platelet and endothelial cell adhesion molecule 1 (CD31). All immunofluorescence microscopy specimens were counterstained with the DNA dye DAPI (blue). (**c**) Analysis of *in vivo* differentiation of iPSCs by teratoma assays. iPSCs were injected subcutaneously into immunodeficient NOD.Cg-$Prkdc^{scid}Il2rg^{tm1Wjl}$/SzJ (NSG) mice. Teratomas derived from iPSCs were analyzed by hematoxylin-phloxine-saffron (HPS) staining and visible light microscopy. Pigmented epithelium (left panel), trachea-like epithelium (middle panel) and cartilage tissue (right panel) belonging to ectodermal, endodermal and mesodermal germ layers, respectively, were identified. Representative morphological structures are outlined. (**d**) Karyotyping of iPSCs. COBRA-FISH analysis of undifferentiated iPSCs revealed a normal diploid karyotype (46, XX).

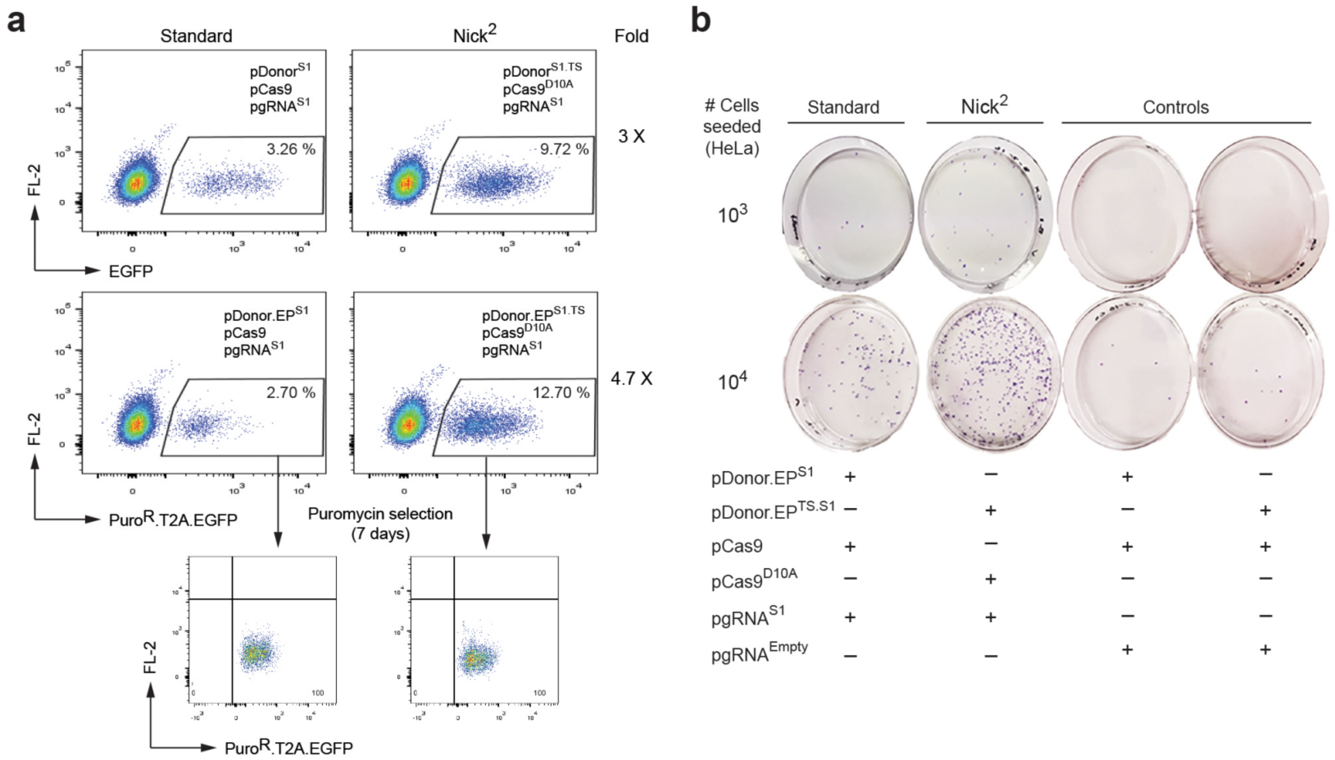

Supplementary Figure 11. Validation of *in trans* paired nicking with pDonor.EP^{S1} and pDonor.EP$^{S1.TS}$. (**a**) Quantification of genetically modified HeLa cells by flow cytometry. EGFP-directed flow cytometry dot plots of long-term HeLa cell cultures initially transfected with the indicated plasmids. The enhancement factors on the frequencies of stably transfected cells resulting from *in trans* paired nicking versus standard gene targeting approaches are indicated. Ten thousand events, each representing a single viable cell, were measured per sample. Stably transfected cells present in long-term Hela cell cultures were selected by an incubation with puromycin for 7 days. (**b**) Identification of genetically modified HeLa cells by colony-formation assays. Thousand and ten-thousand cells from long-term HeLa cell cultures initially transfected with the indicated plasmids were treated for 7 days with puromycin. Puromycin-resistant colonies present in cells subjected to RGN-induced standard and *in trans* paired nicking (Nick^2) gene targeting were identified by Giemsa staining.

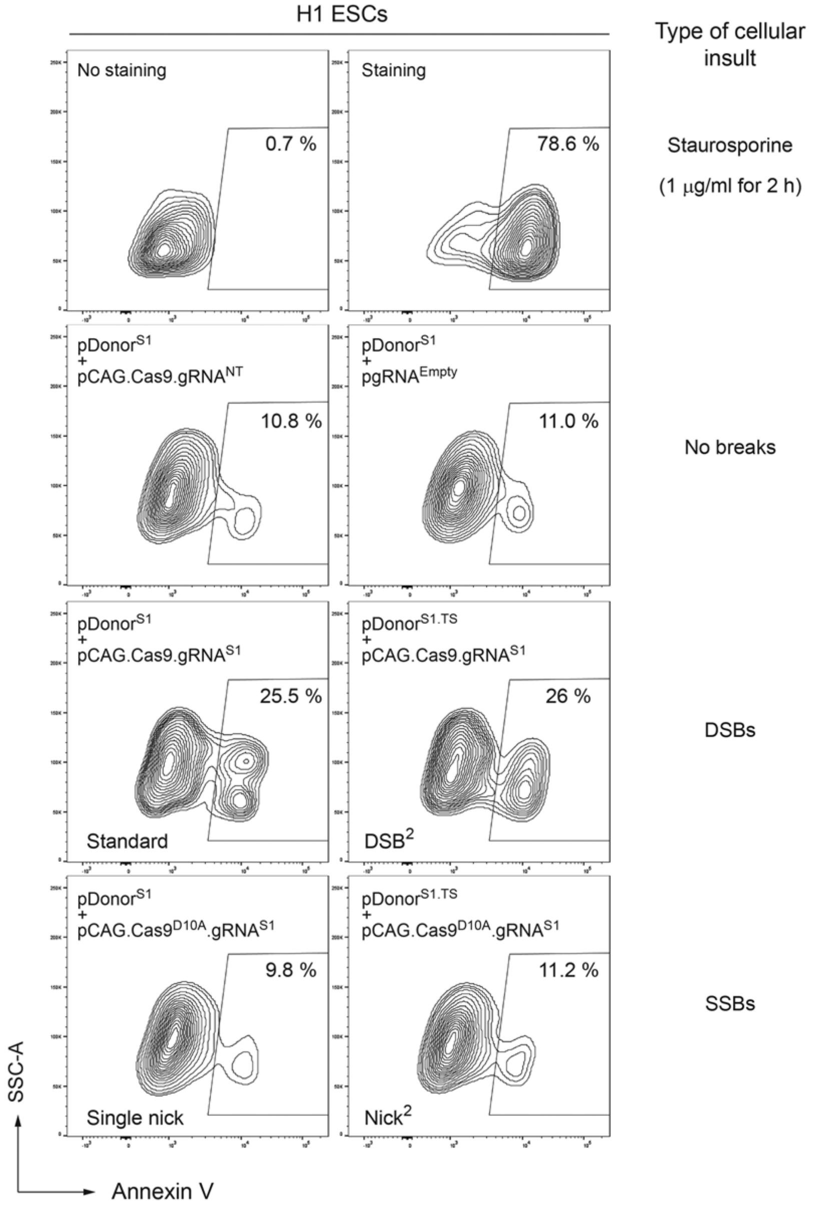

Supplementary Figure 12. Detection of apoptosis in ESCs subjected to DSB- versus SSB-dependent gene targeting strategies. Quantification of apoptosis by flow cytometric analysis of Annexin V binding to phosphatidylserine on ESCs treated with the indicated agents. The frequencies of $EGFP^+$ and Annexin V-binding cells in transfected ESC cultures were determined at 2 days post-transfection. Staurosporine-incubated ESCs stained or not stained with fluorochrome-conjugated Annexin V served as positive and negative controls, respectively.

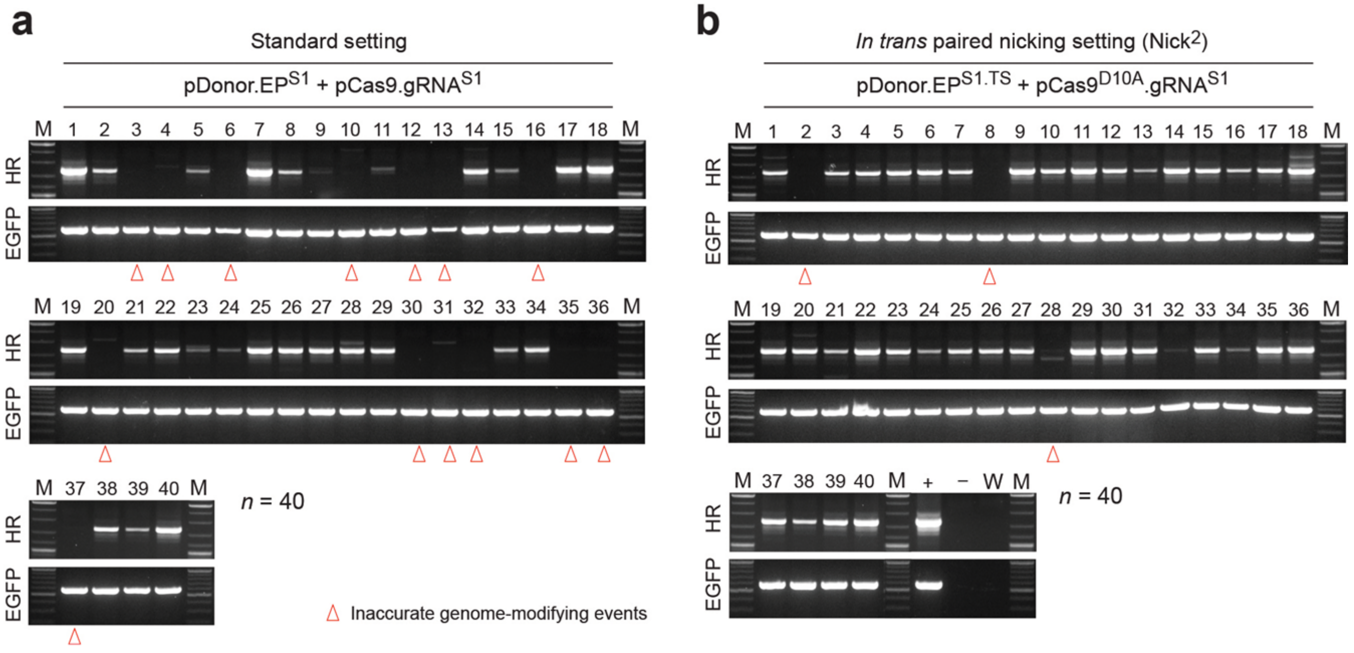

Supplementary Figure 13. Molecular characterization of iPSCs genetically modified by cleaving versus *in trans* paired nicking at *AAVS1*. (**a**) Screening of iPSCs subjected to RGN-induced standard gene targeting. Junction PCR analyses of individual puromycin-resistant iPSC colonies retrieved from cultures co-transfected with pDonor.EP^{S1} and pCAG.Cas9.gRNA^{S1}. (**b**) Screening of iPSCs treated subjected to *in trans* paired nicking gene targeting (Nick^{2}). Junction PCR analyses of individual puromycin-resistant iPSC colonies isolated from cultures co-transfected with pDonor.EP$^{S1.TS}$ and pCAG.Cas9^{D10A}.gRNA^{S1}. The PCR screening was carried out with the primers specific for the centromere-sided *AAVS1*-exogenous DNA junction (HR). PCR amplifications targeting EGFP provided for internal controls (EGFP). PCR mixtures containing unmodified genomic DNA (-) or nuclease-free water (W) served as negative controls. Genomic DNA from an AAVS1-targeted 293T clone was used as a positive control. Lanes M, GeneRuler DNA Ladder Mix molecular weight marker.

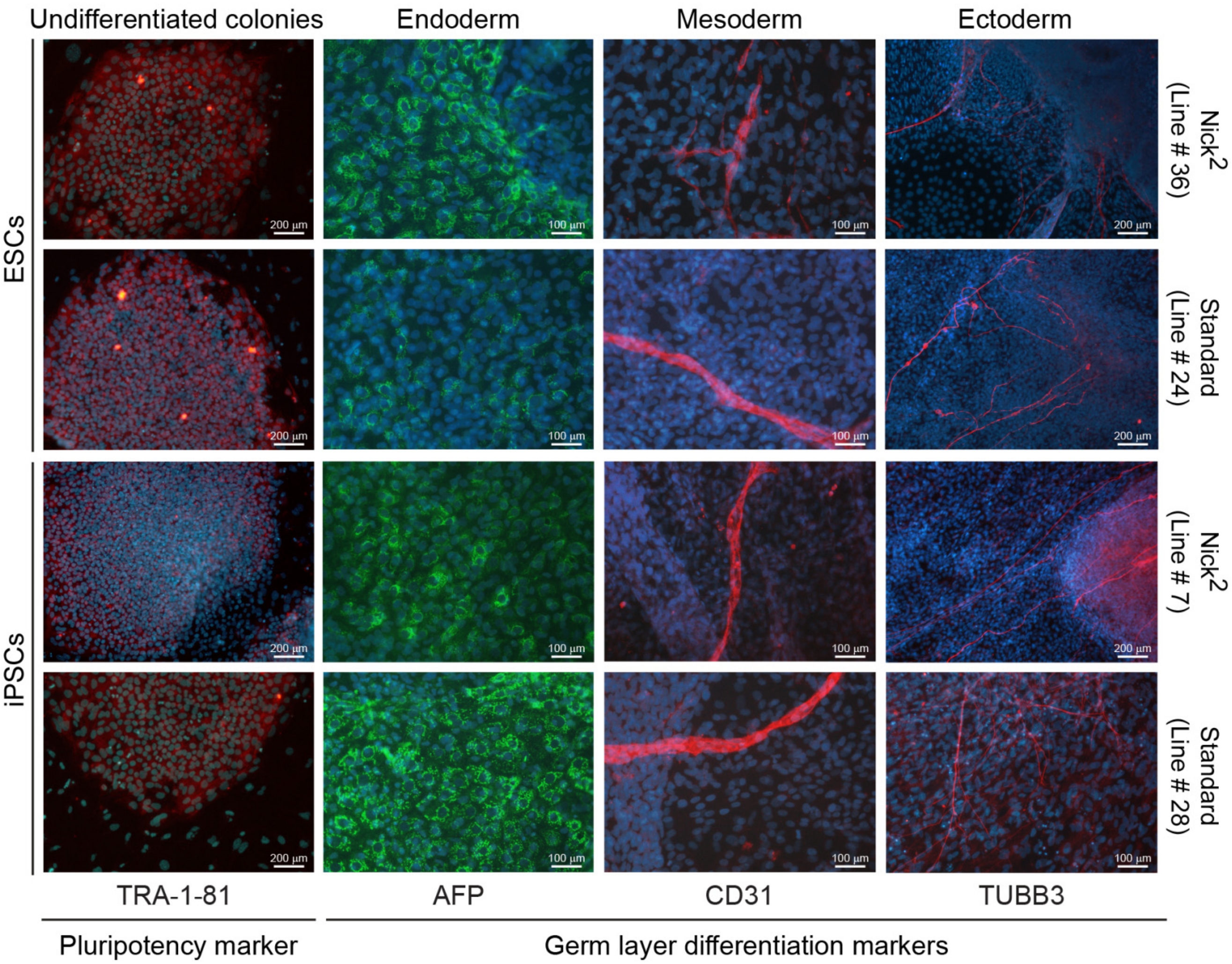

Supplementary Figure 14. Differentiation potential of PSC lines genetically modified by standard and *in trans* paired nicking. Immunofluorescence microscopy analysis of ESC and iPSC clones targeted at *AAVS1* by DSB-dependent (Standard) and *in trans* paired nicking ($Nick^2$) protocols before and after differentiation. The marker for pluripotency and for each of the three embryo germ layers are indicated.

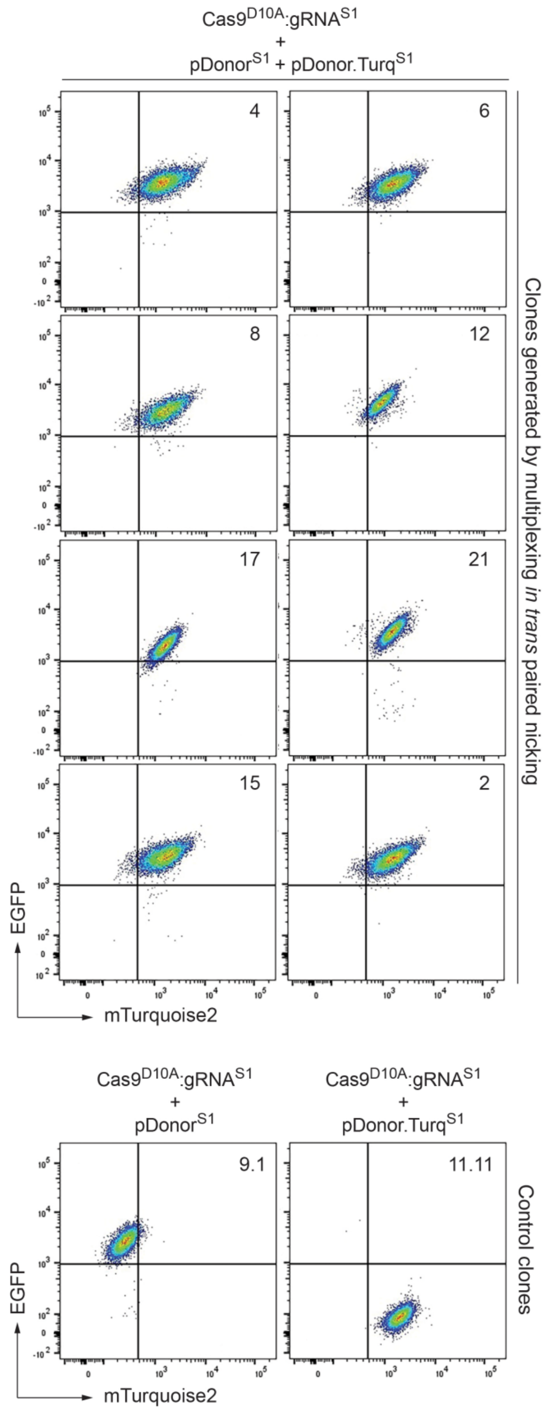

Supplementary Figure 15. Dual-colour flow cytometric analysis of gene co-targeting by multiplexing *in trans* paired nicking. Representative EGFP$^+$/mTurquoise2$^+$ HeLa clones generated by *in trans* paired nicking using two different donor plasmids. Inset numerals correspond to clone codes. Clones derived from HeLa cell cultures subjected to *in trans* paired nicking with a single donor plasmid aided in setting the fluorescence signal thresholds for the dual-colour flow cytometric analysis.

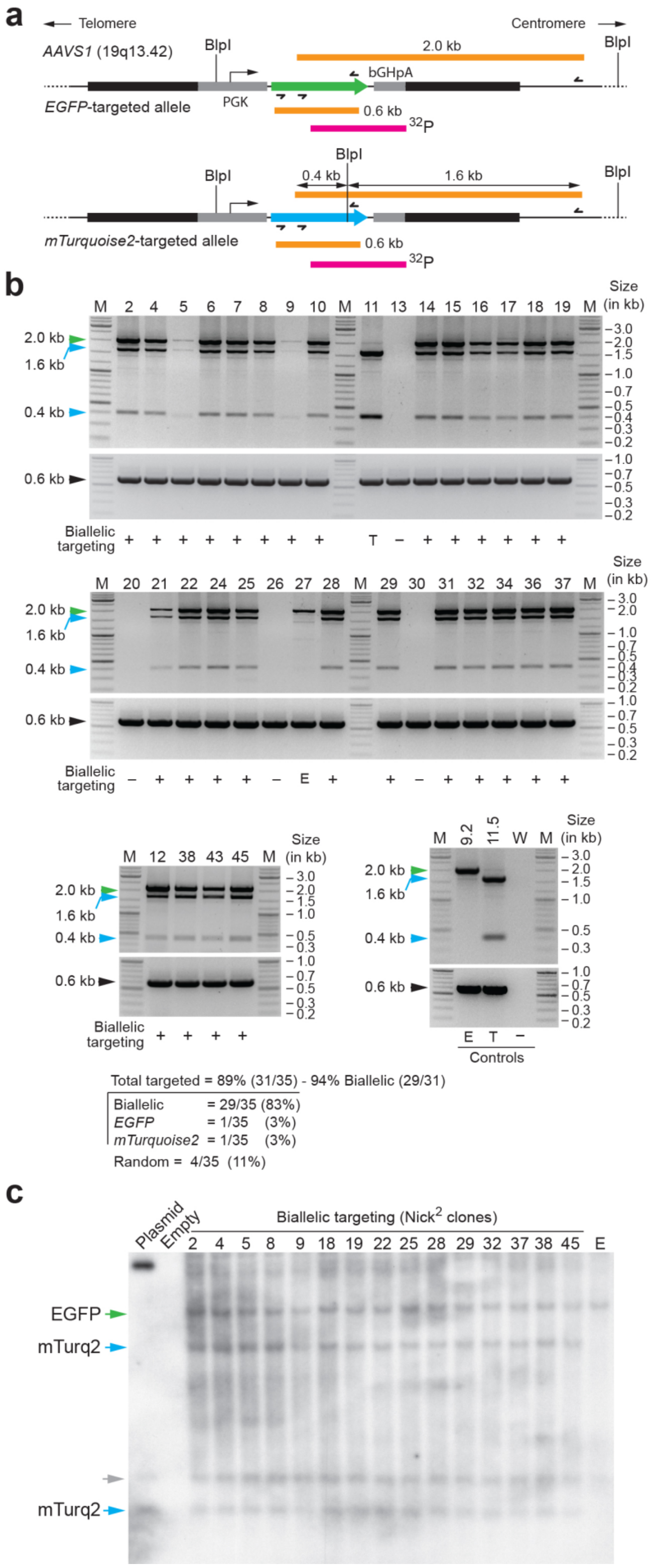

Supplementary Figure 16. Molecular analysis of gene co-targeting by multiplexing *in trans* paired nicking. (**a**) Schematics of the restriction enzyme polymorphism assay used for

identifying co-targeting events at *AAVS1*. The presence and absence of a BlpI recognition site marks integrants resulting from HR events between *AAVS1* and, respectively, pDonor.Turq^{S1} and pDonor^{S1} sequences. The 2-kb amplicons susceptible and resistant to BlpI digestion are diagnostic for *mTurquoise2*- and *EGFP*-targeted *AAVS1* alleles, respectively. Solid boxes, *AAVS1* nucleotide sequences shared by target and donor DNA ("arms of homology"); grey boxes with and without broken arrow, human *PGK1* promoter and bovine *GH1* polyadenylation signal, respectively; cyan and green horizontal arrows, *mTurquoise2* and *EGFP* ORFs, respectively; half arrows, primers for amplifying 0.6 kb of recombinant DNA or 2.0 kb segments specific for recombinant-endogenous DNA junctions formed by HR events; horizontal magenta bars, ^{32}P-labelled Southern blotting probe (**b**) Assessing co-targeting of *AAVS1* alleles after multiplexing *in trans* paired nicking. Genomic DNA samples from EGFP^{+}/mTurquoise2^{+} HeLa clones generated by multiplexing *in trans* paired nicking with donor plasmids pDonor^{S1} and pDonor.Turq^{S1}, were screened with the aid of the restriction enzyme polymorphism assay depicted in panel **a**. Genomic DNA samples from EGFP^{+} clone 9.2 and mTurquoise2^{+} clone 11.5 derived from HeLa cell cultures subjected to *in trans* paired nicking with pDonor^{S1} and pDonor.Turq^{S1}, respectively, served as controls. The 0.6 kb *mTurquoise2*- and *EGFP*-derived PCR products ascertained the integrity of the chromosomal DNA present in each sample. (**c**) Assessing co-targeting of *AAVS1* alleles after multiplexing *in trans* paired nicking by Southern blot analysis. BlpI-digested genomic DNA from randomly selected EGFP^{+}/mTurquoise2^{+} HeLa clones were screened by Southern blotting using the ^{32}P-labelled probes shown in panel **a**. Nonspecific binding of the probes (background) to a DNA fragment from BlpI-digested pDonor.Turq^{S1} and BlpI-digested genomic DNA is indicated by the grey arrow. Green and cyan arrows point to the *EGFP*- and *mTurquoise2*-specific DNA fragments, respectively. Lane E, genomic DNA from an EGFP^{+} HeLa cell clone.

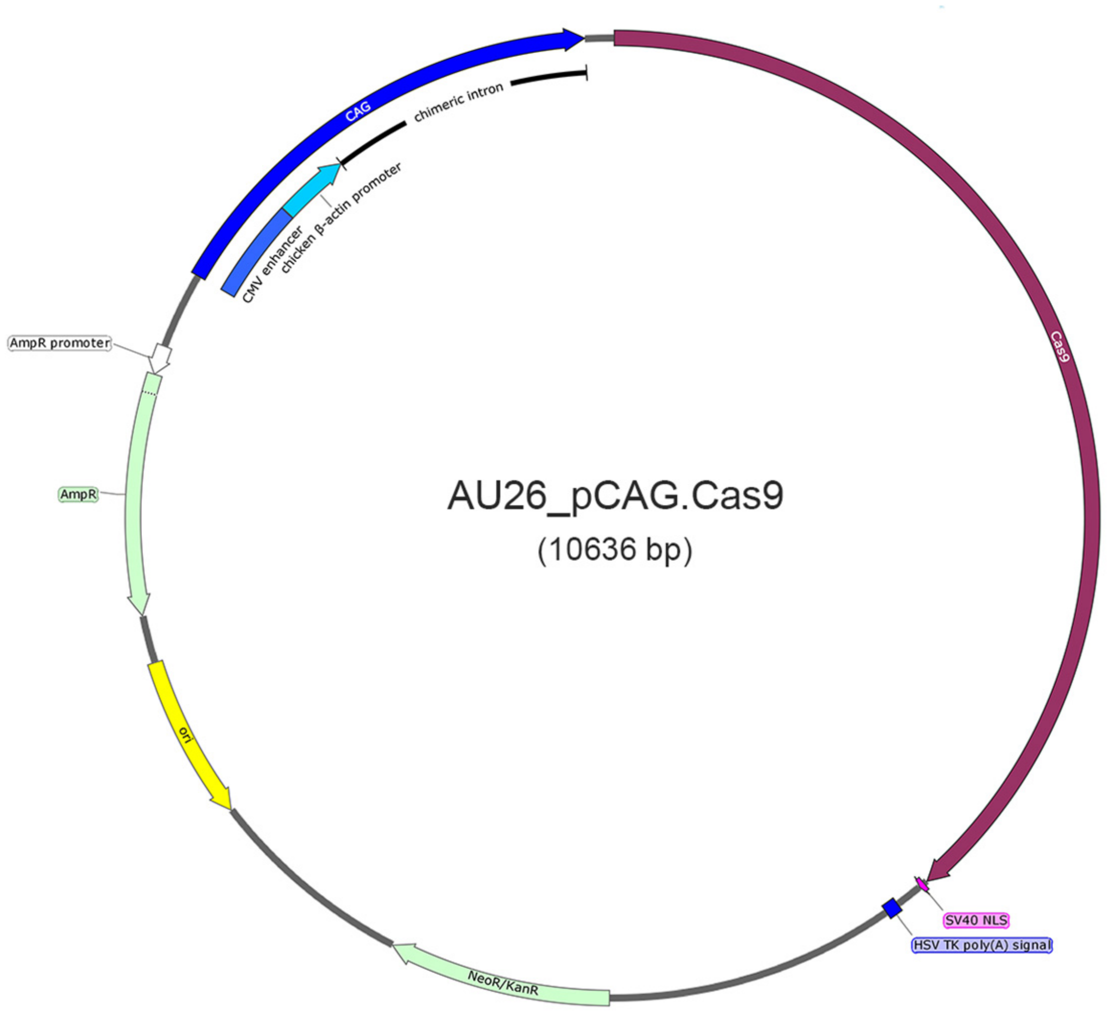

>AU26_pCAG.Cas9 (10,636 bp)
GCCACCATGGACAAGAAGTACTCCATTGGGCTCGATATCGGCACAAACAGCGTCGGCTGGGCCGTCATTACGGACGAGTA
CAAGGTGCCGAGCAAAAAATTCAAAGTTCTGGGCAATACCGATCGCCACAGCATAAAGAAGAACCTCATTGGCGCCCTCC
TGTTCGACTCCGGGGAGACGGCCGAAGCCACGCGGCTCAAAAGAACAGCACGGCGCAGATATACCCGCAGAAAGAATCGG
ATCTGCTACCTGCAGGAGATCTTTAGTAATGAGATGGCTAAGGTGGATGACTCTTTCTTCCATAGGCTGGAGGAGTCCTT
TTTGGTGGAGGAGGATAAAAAGCACGAGCGCCACCCAATCTTTGGCAATATCGTGGACGAGGTGGCGTACCATGAAAAGT
ACCCAACCATATATCATCTGAGGAAGAAGCTTGTAGACAGTACTGATAAGGCTGACTTGCGGTTGATCTATCTCGCGCTG
GCGCATATGATCAAATTTCGGGGACACTTCCTCATCGAGGGGGACCTGAACCCAGACAACAGCGATGTCGACAAACTCTT
TATCCAACTGGTTCAGACTTACAATCAGCTTTTCGAAGAGAACCCGATCAACGCATCCGGAGTTGACGCCAAAGCAATCC
TGAGCGCTAGGCTGTCCAAATCCCGGCGGCTCGAAAACCTCATCGCACAGCTCCCTGGGGAGAAGAAGAACGGCCTGTTT
GGTAATCTTATCGCCCTGTCACTCGGGCTGACCCCCAACTTTAAATCTAACTTCGACCTGGCCGAAGATGCCAAGCTTCA
ACTGAGCAAAGACACCTACGATGATGATCTCGACAATCTGCTGGCCCAGATCGGCGACCAGTACGCAGACCTTTTTTTGG
CGGCAAAGAACCTGTCAGACGCCATTCTGCTGAGTGATATTCTGCGAGTGAACACGGAGATCACCAAAGCTCCGCTGAGC
GCTAGTATGATCAAGCGCTATGATGAGCACCACCAAGACTTGACTTTGCTGAAGGCCCTTGTCAGACAGCAACTGCCTGA
GAAGTACAAGGAAATTTTCTTCGATCAGTCTAAAAATGGCTACGCCGGATACATTGACGGCGGAGCAAGCCAGGAGGAAT
TTTACAAATTTATTAAGCCCATCTTGGAAAAAATGGACGGCACCGAGGAGCTGCTGGTAAAGCTTAACAGAGAAGATCTG
TTGCGCAAACAGCGCACTTTCGACAATGGAAGCATCCCCCACCAGATTCACCTGGGCGAACTGCACGCTATCCTCAGGCG
GCAAGAGGATTTCTACCCCTTTTTGAAAGATAACAGGGAAAAGATTGAGAAAATCCTCACATTTCGGATACCCTACTATG
TAGGCCCCCTCGCCCGGGGAAATTCCAGATTCGCGTGGATGACTCGCAAATCAGAAGAGACCATCACTCCCTGGAACTTC
GAGGAAGTCGTGGATAAGGGGGCCTCTGCCCAGTCCTTCATCGAAAGGATGACTAACTTTGATAAAAATCTGCCTAACGA
AAAGGTGCTTCCTAAACACTCTCTGCTGTACGAGTACTTCACAGTTTATAACGAGCTCACCAAGGTCAAATACGTCACAG
AAGGGATGAGAAAGCCAGCATTCCTGTCTGGAGAGCAGAAGAAAGCTATCGTGGACCTCCTCTTCAAGACGAACCGGAAA
GTTACCGTGAAACAGCTCAAAGAAGACTATTTCAAAAAGATTGAATGTTTCGACTCTGTTGAAATCAGCGGAGTGGAGGA
TCGCTTCAACGCATCCCTGGGAACGTATCACGATCTCCTGAAAATCATTAAAGACAAGGACTTCCTGGACAATGAGGAGA
ACGAGGACATTCTTGAGGACATTGTCCTCACCCTTACGTTGTTTGAAGATAGGGAGATGATTGAAGAACGCTTGAAAACT
TACGCTCATCTCTTCGACGACAAAGTCATGAAACAGCTCAAGAGGCGCCGATATACAGGATGGGGGCGGCTGTCAAGAAA
ACTGATCAATGGGATCCGAGACAAGCAGAGTGGAAAGACAATCCTGGATTTTCTTAAGTCCGATGGATTTGCCAACCGGA

ACTTCATGCAGTTGATCCATGATGACTCTCTCACCTTTAAGGAGGACATCCAGAAAGCACAAGTTTCTGGCCAGGGGGAC
AGTCTTCACGAGCACATCGCTAATCTTGCAGGTAGCCCAGCTATCAAAAAGGGAATACTGCAGACCGTTAAGGTCGTGGA
TGAACTCGTCAAAGTAATGGGAAGGCATAAGCCCGAGAATATCGTTATCGAGATGGCCCGAGAGAACCAAACTACCCAGA
AGGGACAGAAGAACAGTAGGGAAAGGATGAAGAGGATTGAAGAGGGTATAAAAGAACTGGGGTCCCAAATCCTTAAGGAA
CACCCAGTTGAAAACACCCAGCTTCAGAATGAGAAGCTCTACCTGTACTACCTGCAGAACGGCAGGGACATGTACGTGGA
TCAGGAACTGGACATCAATCGGCTCTCCGACTACGACGTGGATCATATCGTGCCCCAGTCTTTTCTCAAAGATGATTCTA
TTGATAATAAAGTGTTGACAAGATCCGATAAAAATAGAGGGAAGAGTGATAACGTCCCCTCAGAAGAAGTTGTCAAGAAA
ATGAAAAATTATTGGCGGCAGCTGCTGAACGCCAAACTGATCACACAACGGAAGTTCGATAATCTGACTAAGGCTGAACG
AGGTGGCCTGTCTGAGTTGGATAAAGCCGGCTTCATCAAAAGGCAGCTTGTTGAGACACGCCAGATCACCAAGCACGTGG
CCCAAATTCTCGATTCACGCATGAACACCAAGTACGATGAAAATGACAAACTGATTCGAGAGGTGAAAGTTATTACTCTG
AAGTCTAAGCTGGTCTCAGATTTCAGAAAGGACTTTCAGTTTTATAAGGTGAGAGAGATCAACAATTACCACCATGCGCA
TGATGCCTACCTGAATGCAGTGGTAGGCACTGCACTTATCAAAAAATATCCCAAGCTTGAATCTGAATTTGTTTACGGAG
ACTATAAAGTGTACGATGTTAGGAAAATGATCGCAAAGTCTGAGCAGGAAATAGGCAAGGCCACCGCTAAGTACTTCTTT
TACAGCAATATTATGAATTTTTTCAAGACCGAGATTACACTGGCCAATGGAGAGATTCGGAAGCGACCACTTATCGAAAC
AAACGGAGAAACAGGAGAAATCGTGTGGGACAAGGGTAGGGATTTCGCGACAGTCCGGAAGGTCCTGTCCATGCCGCAGG
TGAACATCGTTAAAAAGACCGAAGTACAGACCGGAGGCTTCTCCAAGGAAAGTATCCTCCCGAAAAGGAACAGCGACAAG
CTGATCGCACGCAAAAAAGATTGGGACCCCAAGAAATACGGCGGATTCGATTCTCCTACAGTCGCTTACAGTGTACTGGT
TGTGGCCAAAGTGGAGAAAGGGAAGTCTAAAAAACTCAAAAGCGTCAAGGAACTGCTGGGCATCACAATCATGGAGCGAT
CAAGCTTCGAAAAAAACCCCATCGACTTTCTCGAGGCGAAAGGATATAAAGAGGTCAAAAAAGACCTCATCATTAAGCTT
CCCAAGTACTCTCTCTTTGAGCTTGAAAACGGCCGGAAACGAATGCTCGCTAGTGCGGGCGAGCTGCAGAAAGGTAACGA
GCTGGCACTGCCCTCTAAATACGTTAATTTCTTGTATCTGGCCAGCCACTATGAAAAGCTCAAAGGGTCTCCCGAAGATA
ATGAGCAGAAGCAGCTGTTCGTGGAACAACACAAACACTACCTTGATGAGATCATCGAGCAAATAAGCGAATTCTCCAAA
AGAGTGATCCTCGCCGACGCTAACCTCGATAAGGTGCTTTCTGCTTACAATAAGCACAGGGATAAGCCCATCAGGGAGCA
GGCAGAAAACATTATCCACTTGTTTACTCTGACCAACTTGGGCGCGCCTGCAGCCTTCAAGTACTTCGACACCACCATAG
ACAGAAAGCGGTACACCTCTACAAAGGAGGTCCTGGACGCCACACTGATTCATCAGTCAATTACGGGGCTCTATGAAACA
AGAATCGACCTCTCTCAGCTCGGTGGAGACAGCAGGGCTGACCCCAAGAAGAAGAGGAAGGTGTGAAAGGGTTCGATCCC
TACCGGTTAGTAATGAGTTTAAACGGGGGAGGCTAACTGAAACACGGAAGGAGACAATACCGGAAGGAACCCGCGCTATG
ACGGCAATAAAAAGACAGAATAAAACGCACGGGTGTTGGGTCGTTTGTTCATAAACGCGGGGTTCGGTCCCAGGGCTGGC
ACTCTGTCGATACCCCACCGAGACCCCATTGGGGCCAATACGCCCGCGTTTCTTCCTTTTCCCCACCCCACCCCCCAAGT
TCGGGTGAAGGCCCAGGGCTCGCAGCCAACGTCGGGGCGGCAGGCCCTGCCATAGCAGATCTGCGCAGCTGGGGCTCTAG
GGGGTATCCCCACGCGCCCTGTAGCGGCGCATTAAGCGCGGCGGGTGTGGTGGTTACGCGCAGCGTGACCGCTACACTTG
CCAGCGCCCTAGCGCCCGCTCCTTTCGCTTTCTTCCCTTCCTTTCTCGCCACGTTCGCCGGCTTTCCCCGTCAAGCTCTA
AATCGGGGGCTCCCTTTAGGGTTCCGATTTAGTGCTTTACGGCACCTCGACCCCAAAAAACTTGATTAGGGTGATGGTTC
ACGTAGTGGGCCATCGCCCTGATAGACGGTTTTTCGCCCTTTGACGTTGGAGTCCACGTTCTTTAATAGTGGACTCTTGT
TCCAAACTGGAACAACACTCAACCCTATCTCGGTCTATTCTTTTGATTTATAAGGGATTTTGCCGATTTCGGCCTATTGG
TTAAAAAATGAGCTGATTTAACAAAAATTTAACGCGAATTAATTCTGTGGAATGTGTGTCAGTTAGGGTGTGGAAAGTCC
CCAGGCTCCCCAGCAGGCAGAAGTATGCAAAGCATGCATCTCAATTAGTCAGCAACCAGGTGTGGAAAGTCCCCAGGCTC
CCCAGCAGGCAGAAGTATGCAAAGCATGCATCTCAATTAGTCAGCAACCATAGTCCCGCCCCTAACTCCGCCCATCCCGC
CCCTAACTCCGCCCAGTTCCGCCCATTCTCCGCCCCATGGCTGACTAATTTTTTTTATTTATGCAGAGGCCGAGGCCGCC
TCTGCCTCTGAGCTATTCCAGAAGTAGTGAGGAGGCTTTTTTGGAGGCCTAGGCTTTTGCAAAAAGCTCCCGGGAGCTTG
TATATCCATTTTCGGATCTGATCAAGAGACAGGATGAGGATCGTTTCGCATGATTGAACAAGATGGATTGCACGCAGGTT
CTCCGGCCGCTTGGGTGGAGAGGCTATTCGGCTATGACTGGGCACAACAGACAATCGGCTGCTCTGATGCCGCCGTGTTC
CGGCTGTCAGCGCAGGGGCGCCCGGTTCTTTTTGTCAAGACCGACCTGTCCGGTGCCCTGAATGAACTGCAGGACGAGGC
AGCGCGGCTATCGTGGCTGGCCACGACGGGCGTTCCTTGCGCAGCTGTGCTCGACGTTGTCACTGAAGCGGGAAGGGACT
GGCTGCTATTGGGCGAAGTGCCGGGGCAGGATCTCCTGTCATCTCACCTTGCTCCTGCCGAGAAAGTATCCATCATGGCT
GATGCAATGCGGCGGCTGCATACGCTTGATCCGGCTACCTGCCCATTCGACCACCAAGCGAAACATCGCATCGAGCGAGC
ACGTACTCGGATGGAAGCCGGTCTTGTCGATCAGGATGATCTGGACGAAGAGCATCAGGGGCTCGCGCCAGCCGAACTGT
TCGCCAGGCTCAAGGCGCGCATGCCCGACGGCGAGGATCTCGTCGTGACCCATGGCGATGCCTGCTTGCCGAATATCATG
GTGGAAAATGGCCGCTTTTCTGGATTCATCGACTGTGGCCGGCTGGGTGTGGCGGACCGCTATCAGGACATAGCGTTGGC
TACCCGTGATATTGCTGAAGAGCTTGGCGGCGAATGGGCTGACCGCTTCCTCGTGCTTTACGGTATCGCCGCTCCCGATT
CGCAGCGCATCGCCTTCTATCGCCTTCTTGACGAGTTCTTCTGAGCGGGACTCTGGGGTTCGCGAAATGACCGACCAAGC
GACGCCCAACCTGCCATCACGAGATTTCGATTCCACCGCCGCCTTCTATGAAAGGTTGGGCTTCGGAATCGTTTTCCGGG
ACGCCGGCTGGATGATCCTCCAGCGCGGGGATCTCATGCTGGAGTTCTTCGCCCACCCCAACTTGTTTATTGCAGCTTAT
AATGGTTACAAATAAAGCAATAGCATCACAAATTTCACAAATAAAGCATTTTTTTCACTGCATTCTAGTTGTGGTTTGTC
CAAACTCATCAATGTATCTTATCATGTCTGTATACCGTCGACCTCTAGCTAGAGCTTGGCGTAATCATGGTCATAGCTGT
TTCCTGTGTGAAATTGTTATCCGCTCACAATTCCACACAACATACGAGCCGGAAGCATAAAGTGTAAAGCCTGGGGTGCC
TAATGAGTGAGCTAACTCACATTAATTGCGTTGCGCTCACTGCCCGCTTTCCAGTCGGGAAACCTGTCGTGCCAGCTGCA
TTAATGAATCGGCCAACGCGCGGGGAGAGGCGGTTTGCGTATTGGGCGCTCTTCCGCTTCCTCGCTCACTGACTCGCTGC
GCTCGGTCGTTCGGCTGCGGCGAGCGGTATCAGCTCACTCAAAGGCGGTAATACGGTTATCCACAGAATCAGGGGATAAC
GCAGGAAAGAACATGTGAGCAAAAGGCCAGCAAAAGGCCAGGAACCGTAAAAAGGCCGCGTTGCTGGCGTTTTTCCATAG
GCTCCGCCCCCCTGACGAGCATCACAAAAATCGACGCTCAAGTCAGAGGTGGCGAAACCCGACAGGACTATAAAGATACC
AGGCGTTTCCCCCTGGAAGCTCCCTCGTGCGCTCTCCTGTTCCGACCCTGCCGCTTACCGGATACCTGTCCGCCTTTCTC
CCTTCGGGAAGCGTGGCGCTTTCTCATAGCTCACGCTGTAGGTATCTCAGTTCGGTGTAGGTCGTTCGCTCCAAGCTGGG
CTGTGTGCACGAACCCCCCGTTCAGCCCGACCGCTGCGCCTTATCCGGTAACTATCGTCTTGAGTCCAACCCGGTAAGAC
ACGACTTATCGCCACTGGCAGCAGCCACTGGTAACAGGATTAGCAGAGCGAGGTATGTAGGCGGTGCTACAGAGTTCTTG
AAGTGGTGGCCTAACTACGGCTACACTAGAAGAACAGTATTTGGTATCTGCGCTCTGCTGAAGCCAGTTACCTTCGGAAA
AAGAGTTGGTAGCTCTTGATCCGGCAAACAAACCACCGCTGGTAGCGGTGGTTTTTTTGTTTGCAAGCAGCAGATTACGC
GCAGAAAAAAAGGATCTCAAGAAGATCCTTTGATCTTTTCTACGGGGTCTGACGCTCAGTGGAACGAAAACTCACGTTAA

```
GGGATTTTGGTCATGAGATTATCAAAAAGGATCTTCACCTAGATCCTTTTAAATTAAAAATGAAGTTTTAAATCAATCTA
AAGTATATATGAGTAAACTTGGTCTGACAGTTACCAATGCTTAATCAGTGAGGCACCTATCTCAGCGATCTGTCTATTTC
GTTCATCCATAGTTGCCTGACTCCCCGTCGTGTAGATAACTACGATACGGGAGGGCTTACCATCTGGCCCCAGTGCTGCA
ATGATACCGCGAGACCCACGCTCACCGGCTCCAGATTTATCAGCAATAAACCAGCCAGCCGGAAGGGCCGAGCGCAGAAG
TGGTCCTGCAACTTTATCCGCCTCCATCCAGTCTATTAATTGTTGCCGGGAAGCTAGAGTAAGTAGTTCGCCAGTTAATA
GTTTGCGCAACGTTGTTGCCATTGCTACAGGCATCGTGGTGTCACGCTCGTCGTTTGGTATGGCTTCATTCAGCTCCGGT
TCCCAACGATCAAGGCGAGTTACATGATCCCCCATGTTGTGCAAAAAAGCGGTTAGCTCCTTCGGTCCTCCGATCGTTGT
CAGAAGTAAGTTGGCCGCAGTGTTATCACTCATGGTTATGGCAGCACTGCATAATTCTCTTACTGTCATGCCATCCGTAA
GATGCTTTTCTGTGACTGGTGAGTACTCAACCAAGTCATTCTGAGAATAGTGTATGCGGCGACCGAGTTGCTCTTGCCCG
GCGTCAATACGGGATAATACCGCGCCACATAGCAGAACTTTAAAAGTGCTCATCATTGGAAAACGTTCTTCGGGGCGAAA
ACTCTCAAGGATCTTACCGCTGTTGAGATCCAGTTCGATGTAACCCACTCGTGCACCCAACTGATCTTCAGCATCTTTTA
CTTTCACCAGCGTTTCTGGGTGAGCAAAAACAGGAAGGCAAAATGCCGCAAAAAAGGGAATAAGGGCGACACGGAAATGT
TGAATACTCATACTCTTCCTTTTTCAATATTATTGAAGCATTTATCAGGGTTATTGTCTCATGAGCGGATACATATTTGA
ATGTATTTAGAAAAATAAACAAATAGGGGTTCCGCGCACATTTCCCCGAAAAGTGCCACCTGACGTCGACGGATCGGGAG
ATCTCCCGATCCCCTATGGTGCACTCTCAGTACAATCTGCTCTGATGCCGCATAGTTAAGCCAGTATCTGCTCCCTGCTT
GTGTGTTGGAGGTCGCTGAGTAGTGCGCGAGCAAAATTTAAGCTACAACAAGGCAAGGCTTGACCGACAATTGCATGAAG
AATCTGCTTAGGGTTAGGCGTTTTGCGCTGCTTCGCGATGTACGGGCCAGATATACtcgagccatggattcGACATTGAT
TATTGACTAGTTATTAATAGTAATCAATTACGGGGTCATTAGTTCATAGCCCATATATGGAGTTCCGCGTTACATAACTT
ACGGTAAATGGCCCGCCTGGCTGACCGCCCAACGACCCCCGCCCATTGACGTCAATAATGACGTATGTTCCCATAGTAAC
GCCAATAGGGACTTTCCATTGACGTCAATGGGTGGAGTATTTACGGTAAACTGCCCACTTGGCAGTACATCAAGTGTATC
ATATGCCAAGTACGCCCCCTATTGACGTCAATGACGGTAAATGGCCCGCCTGGCATTATGCCCAGTACATGACCTTATGG
GACTTTCCTACTTGGCAGTACATCTACGTATTAGTCATCGCTATTACCATGGgTCGAGGTGAGCCCCACGTTCTGCTTCA
CTCTCCCCATCTCCCCCCCCTCCCCACCCCCAATTTTGTATTTATTTATTTTTTAATTATTTTGTGCAGCGATGGGGGCG
GGGGGGGGGGGGGCGCGCGCCAGGCGGGGCGGGGCGGGGCGAGGGGCGGGGCGGGGCGAGGCGGAGAGGTGCGGCGGCAG
CCAATCAGAGCGGCGCGCTCCGAAAGTTTCCTTTTATGGCGAGGCGGCGGCGGCGGCGGCCCTATAAAAAGCGAAGCGCG
CGGCGGGCGGGAGTCGCTGCGTTGCCTTCGCCCCGTGCCCCGCTCCGCGCCGCCTCGCGCCGCCCGCCCCGGCTCTGACT
GACCGCGTTACTCCCACAGGTGAGCGGGCGGGACGGCCCTTCTCCTCCGGGCTGTAATTAGCGCTTGGTTTAATGACGGC
TCGTTTCTTTTCTGTGGCTGCGTGAAAGCCTTAAAGGGCTCCGGGAGGGCCCTTTGTGCGGGGGGGAGCGGCTCGGGGGG
TGCGTGCGTGTGTGTGTGCGTGGGGAGCGCCGCGTGCGGCCCGCGCTGCCCGGCGGCTGTGAGCGCTGCGGGCGCGGCGC
GGGGCTTTGTGCGCTCCGCGTGTGCGCGAGGGGAGCGCGGCCGGGGGCGGTGCCCCGCGGTGCGGGGGGGCTGCGAGGGG
AACAAAGGCTGCGTGCGGGGTGTGTGCGTGGGGGGGTGAGCAGGGGGTGTGGGCGCGGCGGTCGGGCTGTAACCCCCCCC
TGCACCCCCCTCCCCGAGTTGCTGAGCACGGCCCGGCTTCGGGTGCGGGGCTCCGTGCGGGGCGTGGCGCGGGGCTCGCC
GTGCCGGGCGGGGGGTGGCGGCAGGTGGGGGTGCCGGGCGGGGCGGGGCCGCCTCGGGCCGGGGAGGGCTCGGGGGAGGG
GCGCGGCGGCCCCGGAGCGCCGGCGGCTGTCGAGGCGCGGCGAGCCGCAGCCATTGCCTTTTATGGTAATCGTGCGAGAG
GGCGCAGGGACTTCCTTTGTCCCAAATCTGGCGGAGCCGAAATCTGGGAGGCGCCGCCGCACCCCCTCTAGCGGGCGCGG
GCGAAGCGGTGCGGCGCCGGCAGGAAGGAAATGGGCGGGGAGGGCCTTCGTGCGTCGCCGCGCCGCCGTCCCCTTCTCCA
TCTCCAGCCTCGGGGCTGCCGCAGGGGGACGGCTGCCTTCGGGGGGGACGGGGCAGGGCGGGGTTCGGCTTCTGGCGTGT
GACCGGCGGCTCTAGAGCCTCTGCTAACCATGTTCATGCCTTCTTCTTTTTCCTACAGCTCCTGGGCAACGTGCTGGTTG
TTGTGCTGTCTCATCATTTTGGCAAAGAATTatcgcatgcctgcagagctctagagTCCTAGAGGATCGAACCCTT
```

Supplementary Figure 17. Map and nucleotide sequence of expression plasmid AU26_pCAG.Cas9. CAG, hybrid promoter (CMV enhancer, human cytomegalovirus *immediate early* enhancer; chicken *β-actin* promoter; chimeric intron, fusion between introns from the chicken *β-actin* and rabbit *β-globin* genes); Cas9, human codon-optimized Cas9 (Csn1) ORF coding for the endonuclease from the *Streptococcus pyogenes* type II CRISPR/Cas system; SV40 NLS, nuclear localization signal SV40 large T antigen; HSV TK poly(A) signal, herpes simplex virus *thymidine kinase* polyadenylation signal; NeoR/KanR, transposon Tn5 *aminoglycoside phosphotransferase* conferring resistance to the antibiotics G418 (geneticin) and kanamycin; AmpR, *β-lactamase* ampicillin resistance gene; ori, high-copy number ColE1 prokaryotic origin of replication.

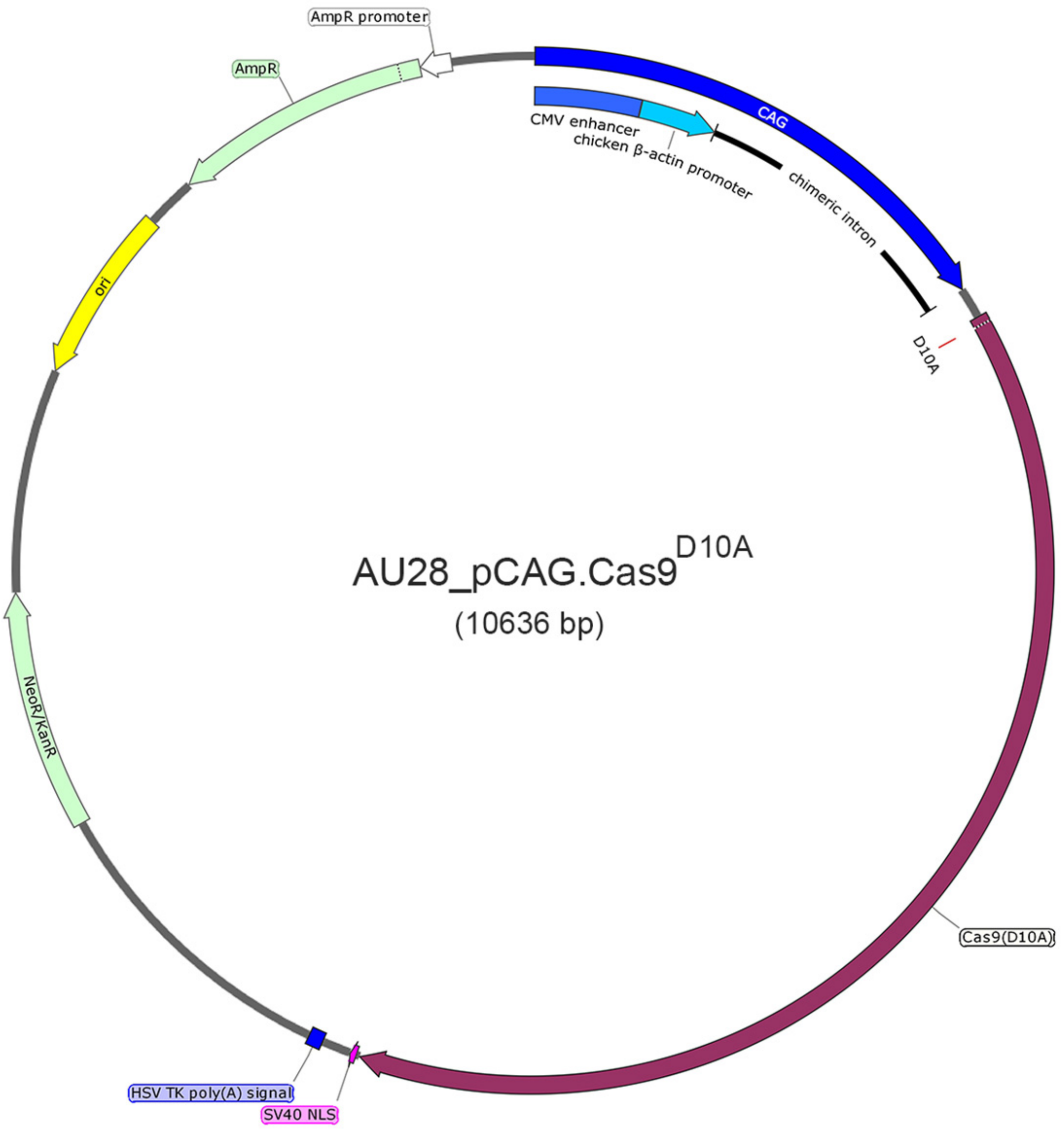

>AU28_pCAG.Cas9^{D10A} (10,636 bp)

```
TCGAGCCATGGATTCGACATTGATTATTGACTAGTTATTAATAGTAATCAATTACGGGGTCATTAGTTCATAGCCCATAT
ATGGAGTTCCGCGTTACATAACTTACGGTAAATGGCCCGCCTGGCTGACCGCCCAACGACCCCCGCCCATTGACGTCAAT
AATGACGTATGTTCCCATAGTAACGCCAATAGGGACTTTCCATTGACGTCAATGGGTGGAGTATTTACGGTAAACTGCCC
ACTTGGCAGTACATCAAGTGTATCATATGCCAAGTACGCCCCCTATTGACGTCAATGACGGTAAATGGCCCGCCTGGCAT
TATGCCCAGTACATGACCTTATGGGACTTTCCTACTTGGCAGTACATCTACGTATTAGTCATCGCTATTACCATGGGTCG
AGGTGAGCCCCACGTTCTGCTTCACTCTCCCCATCTCCCCCCCCTCCCCACCCCCAATTTTGTATTTATTTATTTTTTAA
TTATTTTGTGCAGCGATGGGGGCGGGGGGGGGGGGGGCGCGCGCCAGGCGGGGCGGGGCGGGGCGAGGGGCGGGGCGGGG
CGAGGCGGAGAGGTGCGGCGGCAGCCAATCAGAGCGGCGCGCTCCGAAAGTTTCCTTTTATGGCGAGGCGGCGGCGGCGG
CGGCCCTATAAAAAGCGAAGCGCGCGGCGGGCGGGAGTCGCTGCGTTGCCTTCGCCCCGTGCCCCGCTCCGCGCCGCCTC
GCGCCGCCCGCCCCGGCTCTGACTGACCGCGTTACTCCCACAGGTGAGCGGGCGGGACGGCCCTTCTCCTCCGGGCTGTA
ATTAGCGCTTGGTTTAATGACGGCTCGTTTCTTTTCTGTGGCTGCGTGAAAGCCTTAAAGGGCTCCGGGAGGGCCCTTTG
TGCGGGGGGGAGCGGCTCGGGGGGTGCGTGCGTGTGTGTGTGCGTGGGGAGCGCCGCGTGCGGCCCGCGCTGCCCGGCGG
CTGTGAGCGCTGCGGGCGCGGCGCGGGGCTTTGTGCGCTCCGCGTGTGCGCGAGGGGAGCGCGGCCGGGGGCGGTGCCCC
GCGGTGCGGGGGGGCTGCGAGGGGAACAAAGGCTGCGTGCGGGGTGTGTGCGTGGGGGGGTGAGCAGGGGGTGTGGGCGC
GGCGGTCGGGCTGTAACCCCCCCCTGCACCCCCCTCCCCGAGTTGCTGAGCACGGCCCGGCTTCGGGTGCGGGGCTCCGT
GCGGGGCGTGGCGCGGGGCTCGCCGTGCCGGGCGGGGGGTGGCGGCAGGTGGGGGTGCCGGGCGGGGCGGGGCCGCCTCG
GGCCGGGGAGGGCTCGGGGGAGGGGCGCGGCGGCCCCGGAGCGCCGGCGGCTGTCGAGGCGCGGCGAGCCGCAGCCATTG
```

CCTTTTATGGTAATCGTGCGAGAGGGCGCAGGGACTTCCTTTGTCCCAAATCTGGCGGAGCCGAAATCTGGGAGGCGCCG
CCGCACCCCCTCTAGCGGGCGCGGGCGAAGCGGTGCGGCGCCGGCAGGAAGGAAATGGGCGGGGAGGGCCTTCGTGCGTC
GCCGCGCCGCCGTCCCCTTCTCCATCTCCAGCCTCGGGGCTGCCGCAGGGGGACGGCTGCCTTCGGGGGGGACGGGGCAG
GGCGGGGTTCGGCTTCTGGCGTGTGACCGGCGGCTCTAGAGCCTCTGCTAACCATGTTCATGCCTTCTTCTTTTTCCTAC
AGCTCCTGGGCAACGTGCTGGTTGTTGTGCTGTCTCATCATTTTGGCAAAGAATTATCGCATGCCTGCAGAGCTCTAGAG
TCCTAGAGGATCGAACCCTTGCCACCATGGACAAGAAGTACTCCATTGGGCTCGCTATCGGCACAAACAGCGTCGGCTGG
GCCGTCATTACGGACGAGTACAAGGTGCCGAGCAAAAAATTCAAAGTTCTGGGCAATACCGATCGCCACAGCATAAAGAA
GAACCTCATTGGCGCCCTCCTGTTCGACTCCGGGGAGACGGCCGAAGCCACGCGGCTCAAAAGAACAGCACGGCGCAGAT
ATACCCGCAGAAAGAATCGGATCTGCTACCTGCAGGAGATCTTTAGTAATGAGATGGCTAAGGTGGATGACTCTTTCTTC
CATAGGCTGGAGGAGTCCTTTTTGGTGGAGGAGGATAAAAAGCACGAGCGCCACCCAATCTTTGGCAATATCGTGGACGA
GGTGGCGTACCATGAAAAGTACCCAACCATATATCATCTGAGGAAGAAGCTTGTAGACAGTACTGATAAGGCTGACTTGC
GGTTGATCTATCTCGCGCTGGCGCATATGATCAAATTTCGGGGACACTTCCTCATCGAGGGGGACCTGAACCCAGACAAC
AGCGATGTCGACAAACTCTTTATCCAACTGGTTCAGACTTACAATCAGCTTTTCGAAGAGAACCCGATCAACGCATCCGG
AGTTGACGCCAAAGCAATCCTGAGCGCTAGGCTGTCCAAATCCCGGCGGCTCGAAAACCTCATCGCACAGCTCCCTGGGG
AGAAGAAGAACGGCCTGTTTGGTAATCTTATCGCCCTGTCACTCGGGCTGACCCCCAACTTTAAATCTAACTTCGACCTG
GCCGAAGATGCCAAGCTTCAACTGAGCAAAGACACCTACGATGATGATCTCGACAATCTGCTGGCCCAGATCGGCGACCA
GTACGCAGACCTTTTTTTGGCGGCAAAGAACCTGTCAGACGCCATTCTGCTGAGTGATATTCTGCGAGTGAACACGGAGA
TCACCAAAGCTCCGCTGAGCGCTAGTATGATCAAGCGCTATGATGAGCACCACCAAGACTTGACTTTGCTGAAGGCCCTT
GTCAGACAGCAACTGCCTGAGAAGTACAAGGAAATTTTCTTCGATCAGTCTAAAAATGGCTACGCCGGATACATTGACGG
CGGAGCAAGCCAGGAGGAATTTTACAAATTTATTAAGCCCATCTTGGAAAAAATGGACGGCACCGAGGAGCTGCTGGTAA
AGCTTAACAGAGAAGATCTGTTGCGCAAACAGCGCACTTTCGACAATGGAAGCATCCCCCACCAGATTCACCTGGGCGAA
CTGCACGCTATCCTCAGGCGGCAAGAGGATTTCTACCCCTTTTTGAAAGATAACAGGGAAAAGATTGAGAAAATCCTCAC
ATTTCGGATACCCTACTATGTAGGCCCCCTCGCCCGGGGAAATTCCAGATTCGCGTGGATGACTCGCAAATCAGAAGAGA
CCATCACTCCCTGGAACTTCGAGGAAGTCGTGGATAAGGGGGCCTCTGCCCAGTCCTTCATCGAAAGGATGACTAACTTT
GATAAAAATCTGCCTAACGAAAAGGTGCTTCCTAAACACTCTCTGCTGTACGAGTACTTCACAGTTTATAACGAGCTCAC
CAAGGTCAAATACGTCACAGAAGGGATGAGAAAGCCAGCATTCCTGTCTGGAGAGCAGAAGAAAGCTATCGTGGACCTCC
TCTTCAAGACGAACCGGAAAGTTACCGTGAAACAGCTCAAAGAAGACTATTTCAAAAAGATTGAATGTTTCGACTCTGTT
GAAATCAGCGGAGTGGAGGATCGCTTCAACGCATCCCTGGGAACGTATCACGATCTCCTGAAAATCATTAAAGACAAGGA
CTTCCTGGACAATGAGGAGAACGAGGACATTCTTGAGGACATTGTCCTCACCCTTACGTTGTTTGAAGATAGGGAGATGA
TTGAAGAACGCTTGAAAACTTACGCTCATCTCTTCGACGACAAAGTCATGAAACAGCTCAAGAGGCGCCGATATACAGGA
TGGGGGCGGCTGTCAAGAAAACTGATCAATGGGATCCGAGACAAGCAGAGTGGAAAGACAATCCTGGATTTTCTTAAGTC
CGATGGATTTGCCAACAGGAACTTCATGCAGTTGATCCATGATGACTCTCTCACCTTTAAGGAGGACATCCAGAAAGCAC
AAGTTTCTGGCCAGGGGGACAGTCTTCACGAGCACATCGCTAATCTTGCAGGTAGCCCAGCTATCAAAAAGGGAATACTG
CAGACCGTTAAGGTCGTGGATGAACTCGTCAAAGTAATGGGAAGGCATAAGCCCGAGAATATCGTTATCGAGATGGCCCG
AGAGAACCAAACTACCCAGAAGGGACAGAAGAACAGTAGGGAAAGGATGAAGAGGATTGAAGAGGGTATAAAAGAACTGG
GGTCCCAAATCCTTAAGGAACACCCAGTTGAAAACACCCAGCTTCAGAATGAGAAGCTCTACCTGTACTACCTGCAGAAC
GGCAGGGACATGTACGTGGATCAGGAACTGGACATCAATCGGCTCTCCGACTACGACGTGGATCATATCGTGCCCCAGTC
TTTTCTCAAAGATGATTCTATTGATAATAAAGTGTTGACAAGATCCGATAAAAATAGAGGGAAGAGTGATAACGTCCCCT
CAGAAGAAGTTGTCAAGAAAATGAAAAATTATTGGCGGCAGCTGCTGAACGCCAAACTGATCACACAACGGAAGTTCGAT
AATCTGACTAAGGCTGAACGAGGTGGCCTGTCTGAGTTGGATAAAGCCGGCTTCATCAAAAGGCAGCTTGTTGAGACACG
CCAGATCACCAAGCACGTGGCCCAAATTCTCGATTCACGCATGAACACCAAGTACGATGAAAATGACAAACTGATTCGAG
AGGTGAAAGTTATTACTCTGAAGTCTAAGCTGGTCTCAGATTTCAGAAAGGACTTTCAGTTTTATAAGGTGAGAGAGATC
AACAATTACCACCATGCGCATGATGCCTACCTGAATGCAGTGGTAGGCACTGCACTTATCAAAAAATATCCCAAGCTTGA
ATCTGAATTTGTTTACGGAGACTATAAAGTGTACGATGTTAGGAAAATGATCGCAAAGTCTGAGCAGGAAATAGGCAAGG
CCACCGCTAAGTACTTCTTTTACAGCAATATTATGAATTTTTTCAAGACCGAGATTACACTGGCCAATGGAGAGATTCGG
AAGCGACCACTTATCGAAACAAACGGAGAAACAGGAGAAATCGTGTGGGACAAGGGTAGGGATTTCGCGACAGTCCGGAA
GGTCCTGTCCATGCCGCAGGTGAACATCGTTAAAAAGACCGAAGTACAGACCGGAGGCTTCTCCAAGGAAAGTATCCTCC
CGAAAAGGAACAGCGACAAGCTGATCGCACGCAAAAAAGATTGGGACCCCAAGAAATACGGCGGATTCGATTCTCCTACA
GTCGCTTACAGTGTACTGGTTGTGGCCAAAGTGGAGAAAGGGAAGTCTAAAAAACTCAAAAGCGTCAAGGAACTGCTGGG
CATCACAATCATGGAGCGATCAAGCTTCGAAAAAAACCCCATCGACTTTCTCGAGGCGAAAGGATATAAAGAGGTCAAAA
AAGACCTCATCATTAAGCTTCCCAAGTACTCTCTCTTTGAGCTTGAAAACGGCCGGAAACGAATGCTCGCTAGTGCGGGC
GAGCTGCAGAAAGGTAACGAGCTGGCACTGCCCTCTAAATACGTTAATTTCTTGTATCTGGCCAGCCACTATGAAAAGCT
CAAAGGGTCTCCCGAAGATAATGAGCAGAAGCAGCTGTTCGTGGAACAACACAAACACTACCTTGATGAGATCATCGAGC
AAATAAGCGAATTCTCCAAAAGAGTGATCCTCGCCGACGCTAACCTCGATAAGGTGCTTTCTGCTTACAATAAGCACAGG
GATAAGCCCATCAGGGAGCAGGCAGAAAACATTATCCACTTGTTTACTCTGACCAACTTGGGCGCGCCTGCAGCCTTCAA
GTACTTCGACACCACCATAGACAGAAAGCGGTACACCTCTACAAAGGAGGTCCTGGACGCCACACTGATTCATCAGTCAA
TTACGGGGCTCTATGAAACAAGAATCGACCTCTCTCAGCTCGGTGGAGACAGCAGGGCTGACCCCAAGAAGAAGAGGAAG
GTGTGAAAGGGTTCGATCCCTACCGGTTAGTAATGAGTTTAAACGGGGGAGGCTAACTGAAACACGGAAGGAGACAATAC
CGGAAGGAACCCGCGCTATGACGGCAATAAAAAGACAGAATAAAACGCACGGGTGTTGGGTCGTTTGTTCATAAACGCGG
GGTTCGGTCCCAGGGCTGGCACTCTGTCGATACCCCACCGAGACCCCATTGGGGCCAATACGCCCGCGTTTCTTCCTTTT
CCCCACCCCACCCCCCAAGTTCGGGTGAAGGCCCAGGGCTCGCAGCCAACGTCGGGGCGGCAGGCCCTGCCATAGCAGAT
CTGCGCAGCTGGGGCTCTAGGGGGTATCCCCACGCGCCCTGTAGCGGCGCATTAAGCGCGGCGGGTGTGGTGGTTACGCG
CAGCGTGACCGCTACACTTGCCAGCGCCCTAGCGCCCGCTCCTTTCGCTTTCTTCCCTTCCTTTCTCGCCACGTTCGCCG
GCTTTCCCCGTCAAGCTCTAAATCGGGGGCTCCCTTTAGGGTTCCGATTTAGTGCTTTACGGCACCTCGACCCCAAAAAA
CTTGATTAGGGTGATGGTTCACGTAGTGGGCCATCGCCCTGATAGACGGTTTTTCGCCCTTTGACGTTGGAGTCCACGTT
CTTTAATAGTGGACTCTTGTTCCAAACTGGAACAACACTCAACCCTATCTCGGTCTATTCTTTTGATTTATAAGGGATTT
TGCCGATTTCGGCCTATTGGTTAAAAAATGAGCTGATTTAACAAAAATTTAACGCGAATTAATTCTGTGGAATGTGTGTC
AGTTAGGGTGTGGAAAGTCCCCAGGCTCCCCAGCAGGCAGAAGTATGCAAAGCATGCATCTCAATTAGTCAGCAACCAGG

```
TGTGGAAAGTCCCCAGGCTCCCCAGCAGGCAGAAGTATGCAAAGCATGCATCTCAATTAGTCAGCAACCATAGTCCCGCC
CCTAACTCCGCCCATCCCGCCCCTAACTCCGCCCAGTTCCGCCCATTCTCCGCCCCATGGCTGACTAATTTTTTTTATTT
ATGCAGAGGCCGAGGCCGCCTCTGCCTCTGAGCTATTCCAGAAGTAGTGAGGAGGCTTTTTTGGAGGCCTAGGCTTTTGC
AAAAAGCTCCCGGGAGCTTGTATATCCATTTTCGGATCTGATCAAGAGACAGGATGAGGATCGTTTCGCATGATTGAACA
AGATGGATTGCACGCAGGTTCTCCGGCCGCTTGGGTGGAGAGGCTATTCGGCTATGACTGGGCACAACAGACAATCGGCT
GCTCTGATGCCGCCGTGTTCCGGCTGTCAGCGCAGGGGCGCCCGGTTCTTTTTGTCAAGACCGACCTGTCCGGTGCCCTG
AATGAACTGCAGGACGAGGCAGCGCGGCTATCGTGGCTGGCCACGACGGGCGTTCCTTGCGCAGCTGTGCTCGACGTTGT
CACTGAAGCGGGAAGGGACTGGCTGCTATTGGGCGAAGTGCCGGGGCAGGATCTCCTGTCATCTCACCTTGCTCCTGCCG
AGAAAGTATCCATCATGGCTGATGCAATGCGGCGGCTGCATACGCTTGATCCGGCTACCTGCCCATTCGACCACCAAGCG
AAACATCGCATCGAGCGAGCACGTACTCGGATGGAAGCCGGTCTTGTCGATCAGGATGATCTGGACGAAGAGCATCAGGG
GCTCGCGCCAGCCGAACTGTTCGCCAGGCTCAAGGCGCGCATGCCCGACGGCGAGGATCTCGTCGTGACCCATGGCGATG
CCTGCTTGCCGAATATCATGGTGGAAAATGGCCGCTTTTCTGGATTCATCGACTGTGGCCGGCTGGGTGTGGCGGACCGC
TATCAGGACATAGCGTTGGCTACCCGTGATATTGCTGAAGAGCTTGGCGGCGAATGGGCTGACCGCTTCCTCGTGCTTTA
CGGTATCGCCGCTCCCGATTCGCAGCGCATCGCCTTCTATCGCCTTCTTGACGAGTTCTTCTGAGCGGGACTCTGGGGTT
CGCGAAATGACCGACCAAGCGACGCCCAACCTGCCATCACGAGATTTCGATTCCACCGCCGCCTTCTATGAAAGGTTGGG
CTTCGGAATCGTTTTCCGGGACGCCGGCTGGATGATCCTCCAGCGCGGGGATCTCATGCTGGAGTTCTTCGCCCACCCCA
ACTTGTTTATTGCAGCTTATAATGGTTACAAATAAAGCAATAGCATCACAAATTTCACAAATAAAGCATTTTTTTCACTG
CATTCTAGTTGTGGTTTGTCCAAACTCATCAATGTATCTTATCATGTCTGTATACCGTCGACCTCTAGCTAGAGCTTGGC
GTAATCATGGTCATAGCTGTTTCCTGTGTGAAATTGTTATCCGCTCACAATTCCACACAACATACGAGCCGGAAGCATAA
AGTGTAAAGCCTGGGGTGCCTAATGAGTGAGCTAACTCACATTAATTGCGTTGCGCTCACTGCCCGCTTTCCAGTCGGGA
AACCTGTCGTGCCAGCTGCATTAATGAATCGGCCAACGCGCGGGGAGAGGCGGTTTGCGTATTGGGCGCTCTTCCGCTTC
CTCGCTCACTGACTCGCTGCGCTCGGTCGTTCGGCTGCGGCGAGCGGTATCAGCTCACTCAAAGGCGGTAATACGGTTAT
CCACAGAATCAGGGGATAACGCAGGAAAGAACATGTGAGCAAAAGGCCAGCAAAAGGCCAGGAACCGTAAAAAGGCCGCG
TTGCTGGCGTTTTTCCATAGGCTCCGCCCCCCTGACGAGCATCACAAAAATCGACGCTCAAGTCAGAGGTGGCGAAACCC
GACAGGACTATAAAGATACCAGGCGTTTCCCCCTGGAAGCTCCCTCGTGCGCTCTCCTGTTCCGACCCTGCCGCTTACCG
GATACCTGTCCGCCTTTCTCCCTTCGGGAAGCGTGGCGCTTTCTCATAGCTCACGCTGTAGGTATCTCAGTTCGGTGTAG
GTCGTTCGCTCCAAGCTGGGCTGTGTGCACGAACCCCCCGTTCAGCCCGACCGCTGCGCCTTATCCGGTAACTATCGTCT
TGAGTCCAACCCGGTAAGACACGACTTATCGCCACTGGCAGCAGCCACTGGTAACAGGATTAGCAGAGCGAGGTATGTAG
GCGGTGCTACAGAGTTCTTGAAGTGGTGGCCTAACTACGGCTACACTAGAAGAACAGTATTTGGTATCTGCGCTCTGCTG
AAGCCAGTTACCTTCGGAAAAAGAGTTGGTAGCTCTTGATCCGGCAAACAAACCACCGCTGGTAGCGGTGGTTTTTTTGT
TTGCAAGCAGCAGATTACGCGCAGAAAAAAAGGATCTCAAGAAGATCCTTTGATCTTTTCTACGGGGTCTGACGCTCAGT
GGAACGAAAACTCACGTTAAGGGATTTTGGTCATGAGATTATCAAAAAGGATCTTCACCTAGATCCTTTTAAATTAAAAA
TGAAGTTTTAAATCAATCTAAAGTATATATGAGTAAACTTGGTCTGACAGTTACCAATGCTTAATCAGTGAGGCACCTAT
CTCAGCGATCTGTCTATTTCGTTCATCCATAGTTGCCTGACTCCCCGTCGTGTAGATAACTACGATACGGGAGGGCTTAC
CATCTGGCCCCAGTGCTGCAATGATACCGCGAGACCCACGCTCACCGGCTCCAGATTTATCAGCAATAAACCAGCCAGCC
GGAAGGGCCGAGCGCAGAAGTGGTCCTGCAACTTTATCCGCCTCCATCCAGTCTATTAATTGTTGCCGGGAAGCTAGAGT
AAGTAGTTCGCCAGTTAATAGTTTGCGCAACGTTGTTGCCATTGCTACAGGCATCGTGGTGTCACGCTCGTCGTTTGGTA
TGGCTTCATTCAGCTCCGGTTCCCAACGATCAAGGCGAGTTACATGATCCCCCATGTTGTGCAAAAAAGCGGTTAGCTCC
TTCGGTCCTCCGATCGTTGTCAGAAGTAAGTTGGCCGCAGTGTTATCACTCATGGTTATGGCAGCACTGCATAATTCTCT
TACTGTCATGCCATCCGTAAGATGCTTTTCTGTGACTGGTGAGTACTCAACCAAGTCATTCTGAGAATAGTGTATGCGGC
GACCGAGTTGCTCTTGCCCGGCGTCAATACGGGATAATACCGCGCCACATAGCAGAACTTTAAAAGTGCTCATCATTGGA
AAACGTTCTTCGGGGCGAAAACTCTCAAGGATCTTACCGCTGTTGAGATCCAGTTCGATGTAACCCACTCGTGCACCCAA
CTGATCTTCAGCATCTTTTACTTTCACCAGCGTTTCTGGGTGAGCAAAAACAGGAAGGCAAAATGCCGCAAAAAAGGGAA
TAAGGGCGACACGGAAATGTTGAATACTCATACTCTTCCTTTTTCAATATTATTGAAGCATTTATCAGGGTTATTGTCTC
ATGAGCGGATACATATTTGAATGTATTTAGAAAAATAAACAAATAGGGGTTCCGCGCACATTTCCCCGAAAAGTGCCACC
TGACGTCGACGGATCGGGAGATCTCCCGATCCCCTATGGTGCACTCTCAGTACAATCTGCTCTGATGCCGCATAGTTAAG
CCAGTATCTGCTCCCTGCTTGTGTGTTGGAGGTCGCTGAGTAGTGCGCGAGCAAAATTTAAGCTACAACAAGGCAAGGCT
TGACCGACAATTGCATGAAGAATCTGCTTAGGGTTAGGCGTTTTGCGCTGCTTCGCGATGTACGGGCCAGATATAC
```

Supplementary Figure 18. Map and nucleotide sequence of expression plasmid AU28_pCAG.Cas9^{D10A}. CAG, hybrid promoter (CMV enhancer, human cytomegalovirus *immediate early* enhancer; chicken *β-actin* promoter; chimeric intron, fusion between introns from the chicken *β-actin* and rabbit *β-globin* genes); Cas9, human codon-optimized Cas9 (Csn1) ORF coding for the endonuclease nicking variant D10A from the *Streptococcus pyogenes* type II CRISPR/Cas system; SV40 NLS, nuclear localization signal SV40 large T antigen; HSV TK poly(A) signal, herpes simplex virus *thymidine kinase* polyadenylation signal; NeoR/KanR, transposon Tn5 *aminoglycoside phosphotransferase* conferring resistance to the antibiotics G418 (geneticin) and kanamycin; AmpR, *β-lactamase* ampicillin resistance gene; ori, high-copy number ColE1 prokaryotic origin of replication.

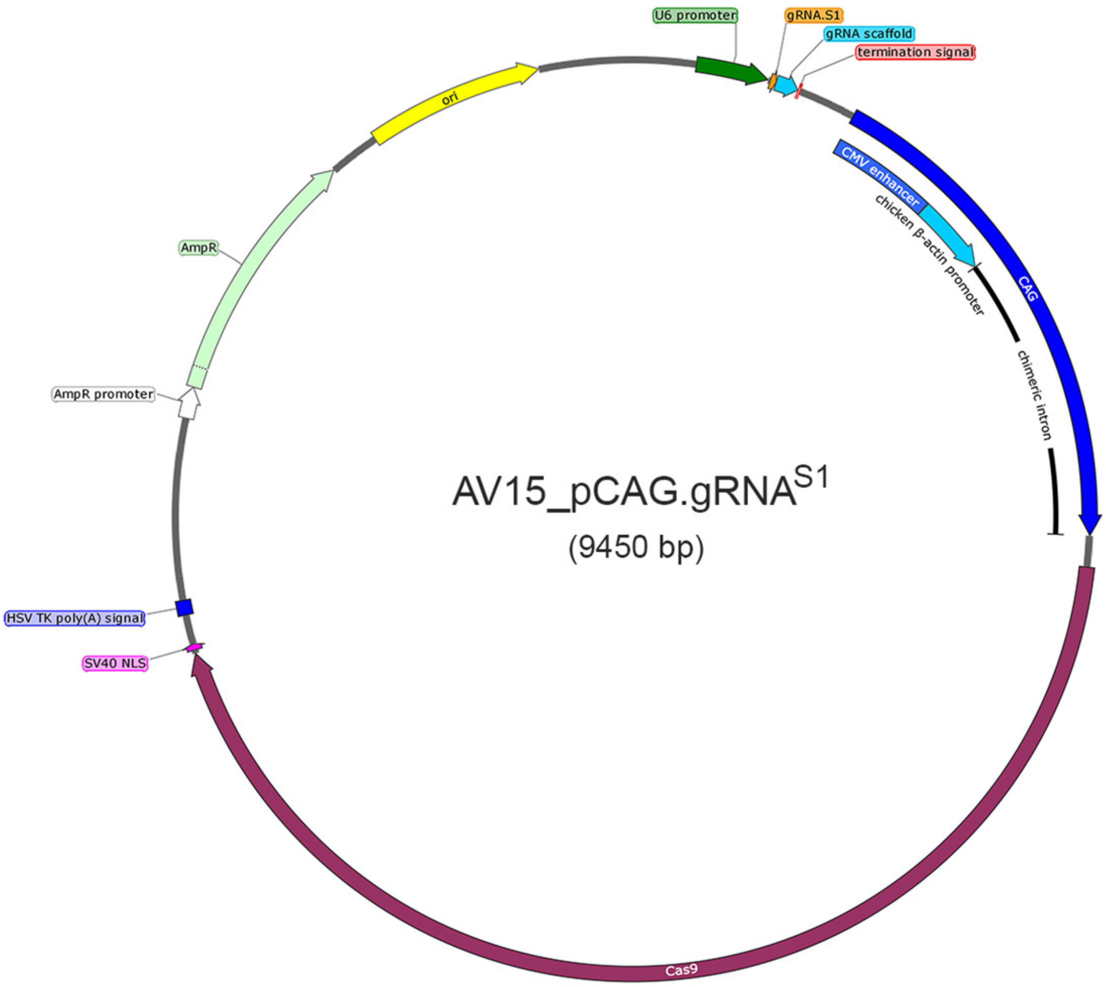

>AV15_pCAG.Cas9.gRNA^{S1} (9450 bp)
ATGTTATTCCTCCTTATTTAAAAGCTTAAGGTGCACGGCCCACGTGGCCACTAGTACTTCTCGAGCTCTGTACATGTCCG
CGGTCGCGACGTACGCGTATCGATGGCGCCAGCTGCAGGCGGCCGCCATATGCATCCTAGGCCTATTAATATTCCGGAGT
ATACGTAGCCGGCTAACGTTGACTGGATCCGGTACCAAGGTCGGGCAGGAAGAGGGCCTATTTCCCATGATTCCTTCATA
TTTGCATATACGATACAAGGCTGTTAGAGAGATAATTAGAATTAATTTGACTGTAAACACAAAGATATTAGTACAAAATA
CGTGACGTAGAAAGTAATAATTTCTTGGGTAGTTTGCAGTTTTAAAATTATGTTTTAAAATGGACTATCATATGCTTACC
GTAACTTGAAAGTATTTCGATTTCTTGGCTTTATATATCTTGTGGAAAGGACGAAACACCGGGGCCACTAGGGACAGGAT
GTTTTAGAGCTAGAAATAGCAAGTTAAAATAAGGCTAGTCCGTTATCAACTTGAAAAAGTGGCACCGAGTCGGTGCTTTT
TTTCTAGACCCAGCTTTCTTGTACAACAACCGGTACCTCTAGAACTATAGCTAGCATGCGCAAATTTAAAGCGCTGATAT
CGATCGCGCGCAGATCTGTCATGATGATCATTGCAATTGCATGAAGAATCTGCTTAGGGTTAGGCGTTTTGCGCTGCTTC
GCGATGTACGGGCCAGATATACtcgagccatggattcGACATTGATTATTGACTAGTTATTAATAGTAATCAATTACGGG
GTCATTAGTTCATAGCCCATATATGGAGTTCCGCGTTACATAACTTACGGTAAATGGCCCGCCTGGCTGACCGCCCAACG
ACCCCCGCCCATTGACGTCAATAATGACGTATGTTCCCATAGTAACGCCAATAGGGACTTTCCATTGACGTCAATGGGTG
GAGTATTTACGGTAAACTGCCCACTTGGCAGTACATCAAGTGTATCATATGCCAAGTACGCCCCCTATTGACGTCAATGA
CGGTAAATGGCCCGCCTGGCATTATGCCCAGTACATGACCTTATGGGACTTTCCTACTTGGCAGTACATCTACGTATTAG
TCATCGCTATTACCATGGgTCGAGGTGAGCCCCACGTTCTGCTTCACTCTCCCCATCTCCCCCCCCTCCCCACCCCCAAT
TTTGTATTTATTTATTTTTTAATTATTTTGTGCAGCGATGGGGGCGGGGGGGGGGGGGGCGCGCGCCAGGCGGGGCGGGG
CGGGGCGAGGGGCGGGGCGGGGCGAGGCGGAGAGGTGCGGCGGCAGCCAATCAGAGCGGCGCGCTCCGAAAGTTTCCTTT
TATGGCGAGGCGGCGGCGGCGGCGGCCCTATAAAAAGCGAAGCGCGCGGCGGGCGGGAGTCGCTGCGTTGCCTTCGCCCC
GTGCCCCGCTCCGCGCCGCCTCGCGCCGCCCGCCCCGGCTCTGACTGACCGCGTTACTCCCACAGGTGAGCGGGCGGGAC
GGCCCTTCTCCTCCGGGCTGTAATTAGCGCTTGGTTTAATGACGGCTCGTTTCTTTTCTGTGGCTGCGTGAAAGCCTTAA
AGGGCTCCGGGAGGGCCCTTTGTGCGGGGGGGAGCGGCTCGGGGGGTGCGTGCGTGTGTGTGTGCGTGGGGAGCGCCGCG
TGCGGCCCGCGCTGCCCGGCGGCTGTGAGCGCTGCGGGCGCGGCGCGGGGCTTTGTGCGCTCCGCGTGTGCGCGAGGGGA
GCGCGGCCGGGGGCGGTGCCCCGCGGTGCGGGGGGGCTGCGAGGGGAACAAAGGCTGCGTGCGGGGTGTGTGCGTGGGGG
GGTGAGCAGGGGGTGTGGGCGCGGCGGTCGGGCTGTAACCCCCCCCTGCACCCCCCTCCCCGAGTTGCTGAGCACGGCCC
GGCTTCGGGTGCGGGGCTCCGTGCGGGGCGTGGCGCGGGGCTCGCCGTGCCGGGCGGGGGGTGGCGGCAGGTGGGGGTGC
CGGGCGGGGCGGGGCCGCCTCGGCCGGGGAGGGCTCGGGGGAGGGGCGCGGCGGCCCCGGAGCGCCGGCGGCTGTCGAG

GCGCGGCGAGCCGCAGCCATTGCCTTTTATGGTAATCGTGCGAGAGGGCGCAGGGACTTCCTTTGTCCCAAATCTGGCGG
AGCCGAAATCTGGGAGGCGCCGCCGCACCCCCTCTAGCGGGCGCGGGCGAAGCGGTGCGGCGCCGGCAGGAAGGAAATGG
GCGGGGAGGGCCTTCGTGCGTCGCCGCGCCGCCGTCCCCTTCTCCATCTCCAGCCTCGGGGCTGCCGCAGGGGGACGGCT
GCCTTCGGGGGGGACGGGGCAGGGCGGGGTTCGGCTTCTGGCGTGTGACCGGCGGCTCTAGAGCCTCTGCTAACCATGTT
CATGCCTTCTTCTTTTTCCTACAGCTCCTGGGCAACGTGCTGGTTGTTGTGCTGTCTCATCATTTTGGCAAAGAATTatc
gcatgcctgcagagctctagagTCCTAGAGGATCGAACCCTTgccaccATGGACAAGAAGTACTCCATTGGGCTCGATAT
CGGCACAAACAGCGTCGGCTGGGCCGTCATTACGGACGAGTACAAGGTGCCGAGCAAAAAATTCAAAGTTCTGGGCAATA
CCGATCGCCACAGCATAAAGAAGAACCTCATTGGCGCCCTCCTGTTCGACTCCGGGGAGACGGCCGAAGCCACGCGGCTC
AAAAGAACAGCACGGCGCAGATATACCCGCAGAAAGAATCGGATCTGCTACCTGCAGGAGATCTTTAGTAATGAGATGGC
TAAGGTGGATGACTCTTTCTTCCATAGGCTGGAGGAGTCCTTTTTGGTGGAGGAGGATAAAAAGCACGAGCGCCACCCAA
TCTTTGGCAATATCGTGGACGAGGTGGCGTACCATGAAAAGTACCCAACCATATATCATCTGAGGAAGAAGCTTGTAGAC
AGTACTGATAAGGCTGACTTGCGGTTGATCTATCTCGCGCTGGCGCATATGATCAAATTTCGGGGACACTTCCTCATCGA
GGGGGACCTGAACCCAGACAACAGCGATGTCGACAAACTCTTTATCCAACTGGTTCAGACTTACAATCAGCTTTTCGAAG
AGAACCCGATCAACGCATCCGGAGTTGACGCCAAAGCAATCCTGAGCGCTAGGCTGTCCAAATCCCGGCGGCTCGAAAAC
CTCATCGCACAGCTCCCTGGGGAGAAGAAGAACGGCCTGTTTGGTAATCTTATCGCCCTGTCACTCGGGCTGACCCCCAA
CTTTAAATCTAACTTCGACCTGGCCGAAGATGCCAAGCTTCAACTGAGCAAAGACACCTACGATGATGATCTCGACAATC
TGCTGGCCCAGATCGGCGACCAGTACGCAGACCTTTTTTTGGCGGCAAAGAACCTGTCAGACGCCATTCTGCTGAGTGAT
ATTCTGCGAGTGAACACGGAGATCACCAAAGCTCCGCTGAGCGCTAGTATGATCAAGCGCTATGATGAGCACCACCAAGA
CTTGACTTTGCTGAAGGCCCTTGTCAGACAGCAACTGCCTGAGAAGTACAAGGAAATTTTCTTCGATCAGTCTAAAAATG
GCTACGCCGGATACATTGACGGCGGAGCAAGCCAGGAGGAATTTTACAAATTTATTAAGCCCATCTTGGAAAAAATGGAC
GGCACCGAGGAGCTGCTGGTAAAGCTTAACAGAGAAGATCTGTTGCGCAAACAGCGCACTTTCGACAATGGAAGCATCCC
CCACCAGATTCACCTGGGCGAACTGCACGCTATCCTCAGGCGGCAAGAGGATTTCTACCCCTTTTTGAAAGATAACAGGG
AAAAGATTGAGAAAATCCTCACATTTCGGATACCCTACTATGTAGGCCCCCTCGCCCGGGGAAATTCCAGATTCGCGTGG
ATGACTCGCAAATCAGAAGAGACCATCACTCCCTGGAACTTCGAGGAAGTCGTGGATAAGGGGGCCTCTGCCCAGTCCTT
CATCGAAAGGATGACTAACTTTGATAAAAATCTGCCTAACGAAAAGGTGCTTCCTAAACACTCTCTGCTGTACGAGTACT
TCACAGTTTATAACGAGCTCACCAAGGTCAAATACGTCACAGAAGGGATGAGAAAGCCAGCATTCCTGTCTGGAGAGCAG
AAGAAAGCTATCGTGGACCTCCTCTTCAAGACGAACCGGAAAGTTACCGTGAAACAGCTCAAAGAAGACTATTTCAAAAA
GATTGAATGTTTCGACTCTGTTGAAATCAGCGGAGTGGAGGATCGCTTCAACGCATCCCTGGGAACGTATCACGATCTCC
TGAAAATCATTAAAGACAAGGACTTCCTGGACAATGAGGAGAACGAGGACATTCTTGAGGACATTGTCCTCACCCTTACG
TTGTTTGAAGATAGGGAGATGATTGAAGAACGCTTGAAAACTTACGCTCATCTCTTCGACGACAAAGTCATGAAACAGCT
CAAGAGGCGCCGATATACAGGATGGGGGCGGCTGTCAAGAAAACTGATCAATGGGATCCGAGACAAGCAGAGTGGAAAGA
CAATCCTGGATTTTCTTAAGTCCGATGGATTTGCCAACCGGAACTTCATGCAGTTGATCCATGATGACTCTCTCACCTTT
AAGGAGGACATCCAGAAAGCACAAGTTTCTGGCCAGGGGGACAGTCTTCACGAGCACATCGCTAATCTTGCAGGTAGCCC
AGCTATCAAAAAGGGAATACTGCAGACCGTTAAGGTCGTGGATGAACTCGTCAAAGTAATGGGAAGGCATAAGCCCGAGA
ATATCGTTATCGAGATGGCCCGAGAGAACCAAACTACCCAGAAGGGACAGAAGAACAGTAGGGAAAGGATGAAGAGGATT
GAAGAGGGTATAAAAGAACTGGGGTCCCAAATCCTTAAGGAACACCCAGTTGAAAACACCCAGCTTCAGAATGAGAAGCT
CTACCTGTACTACCTGCAGAACGGCAGGGACATGTACGTGGATCAGGAACTGGACATCAATCGGCTCTCCGACTACGACG
TGGATCATATCGTGCCCCAGTCTTTTCTCAAAGATGATTCTATTGATAATAAAGTGTTGACAAGATCCGATAAAAATAGA
GGGAAGAGTGATAACGTCCCCTCAGAAGAAGTTGTCAAGAAAATGAAAAATTATTGGCGGCAGCTGCTGAACGCCAAACT
GATCACACAACGGAAGTTCGATAATCTGACTAAGGCTGAACGAGGTGGCCTGTCTGAGTTGGATAAAGCCGGCTTCATCA
AAAGGCAGCTTGTTGAGACACGCCAGATCACCAAGCACGTGGCCCAAATTCTCGATTCACGCATGAACACCAAGTACGAT
GAAAATGACAAACTGATTCGAGAGGTGAAAGTTATTACTCTGAAGTCTAAGCTGGTCTCAGATTTCAGAAAGGACTTTCA
GTTTTATAAGGTGAGAGAGATCAACAATTACCACCATGCGCATGATGCCTACCTGAATGCAGTGGTAGGCACTGCACTTA
TCAAAAAATATCCCAAGCTTGAATCTGAATTTGTTTACGGAGACTATAAAGTGTACGATGTTAGGAAAATGATCGCAAAG
TCTGAGCAGGAAATAGGCAAGGCCACCGCTAAGTACTTCTTTTACAGCAATATTATGAATTTTTTCAAGACCGAGATTAC
ACTGGCCAATGGAGAGATTCGGAAGCGACCACTTATCGAAACAAACGGAGAAACAGGAGAAATCGTGTGGGACAAGGGTA
GGGATTTCGCGACAGTCCGGAAGGTCCTGTCCATGCCGCAGGTGAACATCGTTAAAAAGACCGAAGTACAGACCGGAGGC
TTCTCCAAGGAAAGTATCCTCCCGAAAAGGAACAGCGACAAGCTGATCGCACGCAAAAAAGATTGGGACCCCAAGAAATA
CGGCGGATTCGATTCTCCTACAGTCGCTTACAGTGTACTGGTTGTGGCCAAAGTGGAGAAAGGGAAGTCTAAAAAACTCA
AAAGCGTCAAGGAACTGCTGGGCATCACAATCATGGAGCGATCAAGCTTCGAAAAAAACCCCATCGACTTTCTCGAGGCG
AAAGGATATAAAGAGGTCAAAAAAGACCTCATCATTAAGCTTCCCAAGTACTCTCTCTTTGAGCTTGAAAACGGCCGGAA
ACGAATGCTCGCTAGTGCGGGCGAGCTGCAGAAAGGTAACGAGCTGGCACTGCCCTCTAAATACGTTAATTTCTTGTATC
TGGCCAGCCACTATGAAAAGCTCAAAGGGTCTCCCGAAGATAATGAGCAGAAGCAGCTGTTCGTGGAACAACACAAACAC
TACCTTGATGAGATCATCGAGCAAATAAGCGAATTCTCCAAAAGAGTGATCCTCGCCGACGCTAACCTCGATAAGGTGCT
TTCTGCTTACAATAAGCACAGGGATAAGCCCATCAGGGAGCAGGCAGAAAACATTATCCACTTGTTTACTCTGACCAACT
TGGGCGCGCCTGCAGCCTTCAAGTACTTCGACACCACCATAGACAGAAAGCGGTACACCTCTACAAAGGAGGTCCTGGAC
GCCACACTGATTCATCAGTCAATTACGGGGCTCTATGAAACAAGAATCGACCTCTCTCAGCTCGGTGGAGACAGCAGGGC
TGACCCCAAGAAGAAGAGGAAGGTGTGAAAGGGTTCGATCCCTACCGGTTAGTAATGAGTTTAAACGGGGGAGGCTAACT
GAAACACGGAAGGAGACAATACCGGAAGGAACCCGCGCTATGACGGCAATAAAAAGACAGAATAAAACGCACGGGTGTTG
GGTCGTTTGTTCATAAACGCGGGGTTCGGTCCCAGGGCTGGCACTCTGTCGATACCCCACCGAGACCCCATTGGGGCCAA
TACGCCCGCGTTTCTTCCTTTTCCCCACCCCACCCCCCAAGTTCGGGTGAAGGCCCAGGGCTCGCAGCCAACGTCGGGGC
GGCAGGCCCTGCCATAGCAGATCTGCGCAGCTGGGGCTCTAGGGGGTATCCCCACGCGCCCTGTAGCGGCGCATTAAGCG
CGGCGGGTGTGGTGGTTACGCGCAGCGTGACCGCTACACTTGCCAGCGCCCTAGCGCCCGGGGTTATAATTACCTCAGGT
CGACGTCCCATGTGCAGGTGCTGAATTCGAAGGCATTCCACGACGTGGTCACCAGCCATGGCCATGTCCAACTCCATCAA
ATCAGCTTGAGTAGCCATGCCCATGGCTCCAGCCTGTCCTCGTACCAGCTTTGTTCCCTTTAGTGAGGGGTAATTTCGAG
CTTGGCGTAATCATGGTCATAGCTGTTTCCaattCTTGAAGACGAAAGGGCCTCGTGATACGCCTATTTTTATAGGTTAA
TGTCATGATAATAATGGTTTCTTAGACGTCAGGTGGCACTTTTCGGGGAAATGTGCGCGGAACCCCTATTTGTTTATTTT
TCTAAATACATTCAAATATGTATCCGCTCATGAGACAATAACCCTGATAAATGCTTCAATAATATTGAAAAAGGAAGAGT

ATGAGTATTCAACATTTCCGTGTCGCCCTTATTCCCTTTTTTGCGGCATTTTGCCTTCCTGTTTTTGCTCACCCAGAAAC
GCTGGTGAAAGTAAAAGATGCTGAAGATCAGTTGGGTGCACGAGTGGGTTACATCGAACTGGATCTCAACAGCGGTAAGA
TCCTTGAGAGTTTTCGCCCCGAAGAACGTTTTCCAATGATGAGCACTTTTAAAGTTCTGCTATGTGGCGCGGTATTATCC
CGTGTTGACGCCGGGCAAGAGCAACTCGGTCGCCGCATACACTATTCTCAGAATGACTTGGTTGAGTACTCACCAGTCAC
AGAAAAGCATCTTACGGATGGCATGACAGTAAGAGAATTATGCAGTGCTGCCATAACCATGAGTGATAACACTGCGGCCA
ACTTACTTCTGACAACGATCGGAGGACCGAAGGAGCTAACCGCTTTTTTGCACAACATGGGGGATCATGTAACTCGCCTT
GATCGTTGGGAACCGGAGCTGAATGAAGCCATACCAAACGACGAGCGTGACACCACGATGCCTGCAGCAATGGCAACAAC
GTTGCGCAAACTATTAACTGGCGAACTACTTACTCTAGCTTCCCGGCAACAATTAATAGACTGGATGGAGGCGGATAAAG
TTGCAGGACCACTTCTGCGCTCGGCCCTTCCGGCTGGCTGGTTTATTGCTGATAAATCTGGAGCCGGTGAGCGTGGGTCT
CGCGGTATCATTGCAGCACTGGGGCCAGATGGTAAGCCCTCCCGTATCGTAGTTATCTACACGACGGGGAGTCAGGCAAC
TATGGATGAACGAAATAGACAGATCGCTGAGATAGGTGCCTCACTGATTAAGCATTGGTAACTGTCAGACCAAGTTTACT
CATATATACTTTAGATTGATTTAAAACTTCATTTTTAATTTAAAAGGATCTAGGTGAAGATCCTTTTTGATAATCTCATG
ACCAAAATCCCTTAACGTGAGTTTTCGTTCCACTGAGCGTCAGACCCCGTAGAAAAGATCAAAGGATCTTCTTGAGATCC
TTTTTTTCTGCGCGTAATCTGCTGCTTGCAAACAAAAAAACCACCGCTACCAGCGGTGGTTTGTTTGCCGGATCAAGAGC
TACCAACTCTTTTTCCGAAGGTAACTGGCTTCAGCAGAGCGCAGATACCAAATACTGTCCTTCTAGTGTAGCCGTAGTTA
GGCCACCACTTCAAGAACTCTGTAGCACCGCCTACATACCTCGCTCTGCTAATCCTGTTACCAGTGGCTGCTGCCAGTGG
CGATAAGTCGTGTCTTACCGGGTTGGACTCAAGACGATAGTTACCGGATAAGGCGCAGCGGTCGGGCTGAACGGGGGGTT
CGTGCACACAGCCCAGCTTGGAGCGAACGACCTACACCGAACTGAGATACCTACAGCGTGAGCTATGAGAAAGCGCCACG
CTTCCCGAAGGGAGAAAGGCGGACAGGTATCCGGTAAGCGGCAGGGTCGGAACAGGAGAGCGCACGAGGGAGCTTCCAGG
GGGAAACGCCTGGTATCTTTATAGTCCTGTCGGGTTTCGCCACCTCTGACTTGAGCGTCGATTTTTGTGATGCTCGTCAG
GGGGGCGGAGCCTATGGAAAAACGCCAGCAACGCGGCCTTTTTACGGTTCCTGGCCTTTTGCTGGCCTTTTGCTCACATG
TTCTTTCCTGCGTTATCCCCTGATTCTGTGGATAACCGTATTACCGCCTTTGAGTGAGCTGATACCGCTCGCCGCAGCCG
AACGACCGAGCGCAGCGAGTCAGTGAGCGAGGAAGCGGAAGAGCGCCTGATGCGGTATTTTCTCCTTACGCATCTGTGCG
GTATTTCACACCGCATAGTAAAACGACGGCCAGTGAATTGTAATACGACTCACTATAGGGCGAATTGGAGCTTGGCGTAA
TCATGGTCAT

Supplementary Figure 19. Map and nucleotide sequence of expression plasmid AV15_pCAG.Cas9gRNA^{S1}. CAG, hybrid promoter (CMV enhancer, human cytomegalovirus *immediate early* enhancer; chicken *β-actin* promoter; chimeric intron, fusion between introns from the chicken *β-actin* and rabbit *β-globin* genes); Cas9, human codon-optimized Cas9 (Csn1) ORF coding for the endonuclease from the *Streptococcus pyogenes* type II CRISPR/Cas system; SV40 NLS, nuclear localization signal SV40 large T antigen; HSV TK poly(A) signal, herpes simplex virus *thymidine kinase* polyadenylation signal; NeoR/KanR, transposon Tn5 *aminoglycoside phosphotransferase* conferring resistance to the antibiotics G418 (geneticin) and kanamycin; AmpR, *β-lactamase* ampicillin resistance gene; ori, high-copy number ColE1 prokaryotic origin of replication; U6 promoter, RNA polymerase III promoter for human snRNA; gRNA.S1, *AAVS1*-targeting gRNA^{S1} spacer; termination signal, RNA polymerase III termination signal.

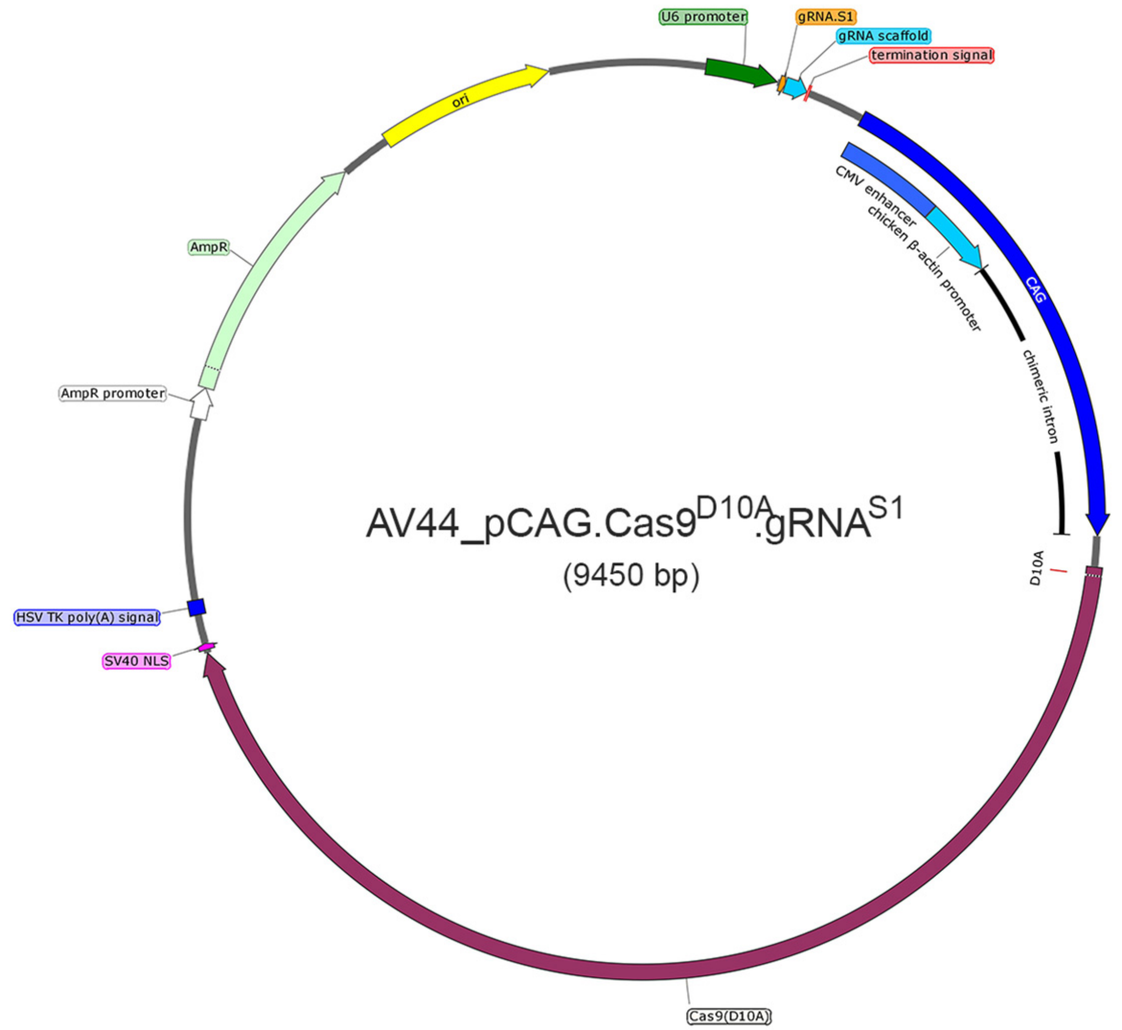

>AV44_p.CAG.Cas9^{D10A}.gRNA^{S1} (9450 bp)
ATGTTATTCCTCCTTATTTAAAAGCTTAAGGTGCACGGCCCACGTGGCCACTAGTACTTCTCGAGCTCTGTACATGTCCG
CGGTCGCGACGTACGCGTATCGATGGCGCCAGCTGCAGGCGGCCGCCATATGCATCCTAGGCCTATTAATATTCCGGAGT
ATACGTAGCCGGCTAACGTTGACTGGATCCGGTACCAAGGTCGGGCAGGAAGAGGGCCTATTTCCCATGATTCCTTCATA
TTTGCATATACGATACAAGGCTGTTAGAGAGATAATTAGAATTAATTTGACTGTAAACACAAAGATATTAGTACAAAATA
CGTGACGTAGAAAGTAATAATTTCTTGGGTAGTTTGCAGTTTTAAAATTATGTTTTAAAATGGACTATCATATGCTTACC
GTAACTTGAAAGTATTTCGATTTCTTGGCTTTATATATCTTGTGGAAAGGACGAAACACCGGGGCCACTAGGGACAGGAT
GTTTTAGAGCTAGAAATAGCAAGTTAAAATAAGGCTAGTCCGTTATCAACTTGAAAAAGTGGCACCGAGTCGGTGCTTTT
TTTCTAGACCCAGCTTTCTTGTACAACAACCGGTACCTCTAGAACTATAGCTAGCATGCGCAAATTTAAAGCGCTGATAT
CGATCGCGCGCAGATCTGTCATGATGATCATTGCAATTGCATGAAGAATCTGCTTAGGGTTAGGCGTTTTGCGCTGCTTC
GCGATGTACGGGCCAGATATACTCGAGCCATGGATTCGACATTGATTATTGACTAGTTATTAATAGTAATCAATTACGGG
GTCATTAGTTCATAGCCCATATATGGAGTTCCGCGTTACATAACTTACGGTAAATGGCCCGCCTGGCTGACCGCCCAACG
ACCCCCGCCCATTGACGTCAATAATGACGTATGTTCCCATAGTAACGCCAATAGGGACTTTCCATTGACGTCAATGGGTG
GAGTATTTACGGTAAACTGCCCACTTGGCAGTACATCAAGTGTATCATATGCCAAGTACGCCCCCTATTGACGTCAATGA
CGGTAAATGGCCCGCCTGGCATTATGCCCAGTACATGACCTTATGGGACTTTCCTACTTGGCAGTACATCTACGTATTAG
TCATCGCTATTACCATGGTCGAGGTGAGCCCCACGTTCTGCTTCACTCTCCCCATCTCCCCCCCCTCCCCACCCCCAAT
TTTGTATTTATTTATTTTTTAATTATTTTGTGCAGCGATGGGGGCGGGGGGGGGGGGGGCGCGCGCCAGGCGGGGCGGGG
CGGGGCGAGGGGCGGGGCGGGGCGAGGCGGAGAGGTGCGGCGGCAGCCAATCAGAGCGGCGCGCTCCGAAAGTTTCCTTT
TATGGCGAGGCGGCGGCGGCGGCGGCCCTATAAAAAGCGAAGCGCGCGGCGGGCGGGAGTCGCTGCGTTGCCTTCGCCCC
GTGCCCCGCTCCGCGCCGCCTCGCGCCGCCCGCCCCGGCTCTGACTGACCGCGTTACTCCCACAGGTGAGCGGGCGGGAC
GGCCCTTCTCCTCCGGGCTGTAATTAGCGCTTGGTTTAATGACGGCTCGTTTCTTTTCTGTGGCTGCGTGAAAGCCTTAA
AGGGCTCCGGGAGGGCCCTTTGTGCGGGGGGGAGCGGCTCGGGGGGTGCGTGCGTGTGTGTGTGCGTGGGGAGCGCCGCG
TGCGGCCCGCGCTGCCCGGCGGCTGTGAGCGCTGCGGGCGCGGCGCGGGGCTTTGTGCGCTCCGCGTGTGCGCGAGGGGA
GCGCGGCCGGGGGCGGTGCCCCGCGGTGCGGGGGGGCTGCGAGGGGAACAAAGGCTGCGTGCGGGGTGTGTGCGTGGGGG
GGTGAGCAGGGGGTGTGGGCGCGGCGGTCGGGCTGTAACCCCCCCCTGCACCCCCCTCCCCGAGTTGCTGAGCACGGCCC

GGCTTCGGGTGCGGGGCTCCGTGCGGGGCGTGGCGCGGGGCTCGCCGTGCCGGGCGGGGGGTGGCGGCAGGTGGGGGTGC
CGGGCGGGGCGGGGCCGCCTCGGGCCGGGGAGGGCTCGGGGGAGGGGCGCGGCGGCCCCGGAGCGCCGGCGGCTGTCGAG
GCGCGGCGAGCCGCAGCCATTGCCTTTTATGGTAATCGTGCGAGAGGGCGCAGGGACTTCCTTTGTCCCAAATCTGGCGG
AGCCGAAATCTGGGAGGCGCCGCCGCACCCCCTCTAGCGGGCGCGGGCGAAGCGGTGCGGCGCCGGCAGGAAGGAAATGG
GCGGGGAGGGCCTTCGTGCGTCGCCGCGCCGCCGTCCCCTTCTCCATCTCCAGCCTCGGGGCTGCCGCAGGGGGACGGCT
GCCTTCGGGGGGGACGGGGCAGGGCGGGGTTCGGCTTCTGGCGTGTGACCGGCGGCTCTAGAGCCTCTGCTAACCATGTT
CATGCCTTCTTCTTTTTCCTACAGCTCCTGGGCAACGTGCTGGTTGTTGTGCTGTCTCATCATTTTGGCAAAGAATTATC
GCATGCCTGCAGAGCTCTAGAGTCCTAGAGGATCGAACCCTTGCCACCATGGACAAGAAGTACTCCATTGGGCTCGCTAT
CGGCACAAACAGCGTCGGCTGGGCCGTCATTACGGACGAGTACAAGGTGCCGAGCAAAAAATTCAAAGTTCTGGGCAATA
CCGATCGCCACAGCATAAAGAAGAACCTCATTGGCGCCCTCCTGTTCGACTCCGGGGAGACGGCCGAAGCCACGCGGCTC
AAAAGAACAGCACGGCGCAGATATACCCGCAGAAAGAATCGGATCTGCTACCTGCAGGAGATCTTTAGTAATGAGATGGC
TAAGGTGGATGACTCTTTCTTCCATAGGCTGGAGGAGTCCTTTTTGGTGGAGGAGGATAAAAAGCACGAGCGCCACCCAA
TCTTTGGCAATATCGTGGACGAGGTGGCGTACCATGAAAAGTACCCAACCATATATCATCTGAGGAAGAAGCTTGTAGAC
AGTACTGATAAGGCTGACTTGCGGTTGATCTATCTCGCGCTGGCGCATATGATCAAATTTCGGGGACACTTCCTCATCGA
GGGGGACCTGAACCCAGACAACAGCGATGTCGACAAACTCTTTATCCAACTGGTTCAGACTTACAATCAGCTTTTCGAAG
AGAACCCGATCAACGCATCCGGAGTTGACGCCAAAGCAATCCTGAGCGCTAGGCTGTCCAAATCCCGGCGGCTCGAAAAC
CTCATCGCACAGCTCCCTGGGGAGAAGAAGAACGGCCTGTTTGGTAATCTTATCGCCCTGTCACTCGGGCTGACCCCCAA
CTTTAAATCTAACTTCGACCTGGCCGAAGATGCCAAGCTTCAACTGAGCAAAGACACCTACGATGATGATCTCGACAATC
TGCTGGCCCAGATCGGCGACCAGTACGCAGACCTTTTTTTGGCGGCAAAGAACCTGTCAGACGCCATTCTGCTGAGTGAT
ATTCTGCGAGTGAACACGGAGATCACCAAAGCTCCGCTGAGCGCTAGTATGATCAAGCGCTATGATGAGCACCACCAAGA
CTTGACTTTGCTGAAGGCCCTTGTCAGACAGCAACTGCCTGAGAAGTACAAGGAAATTTTCTTCGATCAGTCTAAAAATG
GCTACGCCGGATACATTGACGGCGGAGCAAGCCAGGAGGAATTTTACAAATTTATTAAGCCCATCTTGGAAAAAATGGAC
GGCACCGAGGAGCTGCTGGTAAAGCTTAACAGAGAAGATCTGTTGCGCAAACAGCGCACTTTCGACAATGGAAGCATCCC
CCACCAGATTCACCTGGGCGAACTGCACGCTATCCTCAGGCGGCAAGAGGATTTCTACCCCTTTTTGAAAGATAACAGGG
AAAAGATTGAGAAAATCCTCACATTTCGGATACCCTACTATGTAGGCCCCCTCGCCCGGGGAAATTCCAGATTCGCGTGG
ATGACTCGCAAATCAGAAGAGACCATCACTCCCTGGAACTTCGAGGAAGTCGTGGATAAGGGGGCCTCTGCCCAGTCCTT
CATCGAAAGGATGACTAACTTTGATAAAAATCTGCCTAACGAAAAGGTGCTTCCTAAACACTCTCTGCTGTACGAGTACT
TCACAGTTTATAACGAGCTCACCAAGGTCAAATACGTCACAGAAGGGATGAGAAAGCCAGCATTCCTGTCTGGAGAGCAG
AAGAAAGCTATCGTGGACCTCCTCTTCAAGACGAACCGGAAAGTTACCGTGAAACAGCTCAAAGAAGACTATTTCAAAAA
GATTGAATGTTTCGACTCTGTTGAAATCAGCGGAGTGGAGGATCGCTTCAACGCATCCCTGGGAACGTATCACGATCTCC
TGAAAATCATTAAAGACAAGGACTTCCTGGACAATGAGGAGAACGAGGACATTCTTGAGGACATTGTCCTCACCCTTACG
TTGTTTGAAGATAGGGAGATGATTGAAGAACGCTTGAAAACTTACGCTCATCTCTTCGACGACAAAGTCATGAAACAGCT
CAAGAGGCGCCGATATACAGGATGGGGGCGGCTGTCAAGAAAACTGATCAATGGGATCCGAGACAAGCAGAGTGGAAAGA
CAATCCTGGATTTTCTTAAGTCCGATGGATTTGCCAACAGGAACTTCATGCAGTTGATCCATGATGACTCTCTCACCTTT
AAGGAGGACATCCAGAAAGCACAAGTTTCTGGCCAGGGGGACAGTCTTCACGAGCACATCGCTAATCTTGCAGGTAGCCC
AGCTATCAAAAAGGGAATACTGCAGACCGTTAAGGTCGTGGATGAACTCGTCAAAGTAATGGGAAGGCATAAGCCCGAGA
ATATCGTTATCGAGATGGCCCGAGAGAACCAAACTACCCAGAAGGGACAGAAGAACAGTAGGGAAAGGATGAAGAGGATT
GAAGAGGGTATAAAAGAACTGGGGTCCCAAATCCTTAAGGAACACCCAGTTGAAAACACCCAGCTTCAGAATGAGAAGCT
CTACCTGTACTACCTGCAGAACGGCAGGGACATGTACGTGGATCAGGAACTGGACATCAATCGGCTCTCCGACTACGACG
TGGATCATATCGTGCCCCAGTCTTTTCTCAAAGATGATTCTATTGATAATAAAGTGTTGACAAGATCCGATAAAAATAGA
GGGAAGAGTGATAACGTCCCCTCAGAAGAAGTTGTCAAGAAAATGAAAAATTATTGGCGGCAGCTGCTGAACGCCAAACT
GATCACACAACGGAAGTTCGATAATCTGACTAAGGCTGAACGAGGTGGCCTGTCTGAGTTGGATAAAGCCGGCTTCATCA
AAAGGCAGCTTGTTGAGACACGCCAGATCACCAAGCACGTGGCCCAAATTCTCGATTCACGCATGAACACCAAGTACGAT
GAAAATGACAAACTGATTCGAGAGGTGAAAGTTATTACTCTGAAGTCTAAGCTGGTCTCAGATTTCAGAAAGGACTTTCA
GTTTTATAAGGTGAGAGAGATCAACAATTACCACCATGCGCATGATGCCTACCTGAATGCAGTGGTAGGCACTGCACTTA
TCAAAAAATATCCCAAGCTTGAATCTGAATTTGTTTACGGAGACTATAAAGTGTACGATGTTAGGAAAATGATCGCAAAG
TCTGAGCAGGAAATAGGCAAGGCCACCGCTAAGTACTTCTTTTACAGCAATATTATGAATTTTTTCAAGACCGAGATTAC
ACTGGCCAATGGAGAGATTCGGAAGCGACCACTTATCGAAACAAACGGAGAAACAGGAGAAATCGTGTGGGACAAGGGTA
GGGATTTCGCGACAGTCCGGAAGGTCCTGTCCATGCCGCAGGTGAACATCGTTAAAAAGACCGAAGTACAGACCGGAGGC
TTCTCCAAGGAAAGTATCCTCCCGAAAAGGAACAGCGACAAGCTGATCGCACGCAAAAAAGATTGGGACCCCAAGAAATA
CGGCGGATTCGATTCTCCTACAGTCGCTTACAGTGTACTGGTTGTGGCCAAAGTGGAGAAAGGGAAGTCTAAAAAACTCA
AAAGCGTCAAGGAACTGCTGGGCATCACAATCATGGAGCGATCAAGCTTCGAAAAAAACCCCATCGACTTTCTCGAGGCG
AAAGGATATAAAGAGGTCAAAAAAGACCTCATCATTAAGCTTCCCAAGTACTCTCTCTTTGAGCTTGAAAACGGCCGGAA
ACGAATGCTCGCTAGTGCGGGCGAGCTGCAGAAAGGTAACGAGCTGGCACTGCCCTCTAAATACGTTAATTTCTTGTATC
TGGCCAGCCACTATGAAAAGCTCAAAGGGTCTCCCGAAGATAATGAGCAGAAGCAGCTGTTCGTGGAACAACACAAACAC
TACCTTGATGAGATCATCGAGCAAATAAGCGAATTCTCCAAAAGAGTGATCCTCGCCGACGCTAACCTCGATAAGGTGCT
TTCTGCTTACAATAAGCACAGGGATAAGCCCATCAGGGAGCAGGCAGAAAACATTATCCACTTGTTTACTCTGACCAACT
TGGGCGCGCCTGCAGCCTTCAAGTACTTCGACACCACCATAGACAGAAAGCGGTACACCTCTACAAAGGAGGTCCTGGAC
GCCACACTGATTCATCAGTCAATTACGGGGCTCTATGAAACAAGAATCGACCTCTCTCAGCTCGGTGGAGACAGCAGGGC
TGACCCCAAGAAGAAGAGGAAGGTGTGAAAGGGTTCGATCCCTACCGGTTAGTAATGAGTTTAAACGGGGGAGGCTAACT
GAAACACGGAAGGAGACAATACCGGAAGGAACCCGCGCTATGACGGCAATAAAAAGACAGAATAAAACGCACGGGTGTTG
GGTCGTTTGTTCATAAACGCGGGGTTCGGTCCCAGGGCTGGCACTCTGTCGATACCCCACCGAGACCCCATTGGGGCCAA
TACGCCCGCGTTTCTTCCTTTTCCCCACCCCACCCCCCAAGTTCGGGTGAAGGCCCAGGGCTCGCAGCCAACGTCGGGGC
GGCAGGCCCTGCCATAGCAGATCTGCGCAGCTGGGGCTCTAGGGGGTATCCCCACGCGCCCTGTAGCGGCGCATTAAGCG
CGGCGGGTGTGGTGGTTACGCGCAGCGTGACCGCTACACTTGCCAGCGCCCTAGCGCCCGGGGTTATAATTACCTCAGGT
CGACGTCCCATGTGCAGGTGCTGAATTCGAAGGCATTCCACGACGTGGTCACCAGCCATGGCCATGTCCAACTCCATCAA
ATCAGCTTGAGTAGCCATGCCCATGGCTCCAGCCTGTCCTCGTACCAGCTTTGTTCCCTTTAGTGAGGGGTAATTTCGAG
CTTGGCGTAATCATGGTCATAGCTGTTTCCAATTCTTGAAGACGAAAGGGCCTCGTGATACGCCTATTTTTATAGGTTAA

```
TGTCATGATAATAATGGTTTCTTAGACGTCAGGTGGCACTTTTCGGGGAAATGTGCGCGGAACCCCTATTTGTTTATTTT
TCTAAATACATTCAAATATGTATCCGCTCATGAGACAATAACCCTGATAAATGCTTCAATAATATTGAAAAAGGAAGAGT
ATGAGTATTCAACATTTCCGTGTCGCCCTTATTCCCTTTTTTGCGGCATTTTGCCTTCCTGTTTTTGCTCACCCAGAAAC
GCTGGTGAAAGTAAAAGATGCTGAAGATCAGTTGGGTGCACGAGTGGGTTACATCGAACTGGATCTCAACAGCGGTAAGA
TCCTTGAGAGTTTTCGCCCCGAAGAACGTTTTCCAATGATGAGCACTTTTAAAGTTCTGCTATGTGGCGCGGTATTATCC
CGTGTTGACGCCGGGCAAGAGCAACTCGGTCGCCGCATACACTATTCTCAGAATGACTTGGTTGAGTACTCACCAGTCAC
AGAAAAGCATCTTACGGATGGCATGACAGTAAGAGAATTATGCAGTGCTGCCATAACCATGAGTGATAACACTGCGGCCA
ACTTACTTCTGACAACGATCGGAGGACCGAAGGAGCTAACCGCTTTTTTGCACAACATGGGGGATCATGTAACTCGCCTT
GATCGTTGGGAACCGGAGCTGAATGAAGCCATACCAAACGACGAGCGTGACACCACGATGCCTGCAGCAATGGCAACAAC
GTTGCGCAAACTATTAACTGGCGAACTACTTACTCTAGCTTCCCGGCAACAATTAATAGACTGGATGGAGGCGGATAAAG
TTGCAGGACCACTTCTGCGCTCGGCCCTTCCGGCTGGCTGGTTTATTGCTGATAAATCTGGAGCCGGTGAGCGTGGGTCT
CGCGGTATCATTGCAGCACTGGGGCCAGATGGTAAGCCCTCCCGTATCGTAGTTATCTACACGACGGGGAGTCAGGCAAC
TATGGATGAACGAAATAGACAGATCGCTGAGATAGGTGCCTCACTGATTAAGCATTGGTAACTGTCAGACCAAGTTTACT
CATATATACTTTAGATTGATTTAAAACTTCATTTTTAATTTAAAAGGATCTAGGTGAAGATCCTTTTTGATAATCTCATG
ACCAAAATCCCTTAACGTGAGTTTTCGTTCCACTGAGCGTCAGACCCCGTAGAAAAGATCAAAGGATCTTCTTGAGATCC
TTTTTTTCTGCGCGTAATCTGCTGCTTGCAAACAAAAAAACCACCGCTACCAGCGGTGGTTTGTTTGCCGGATCAAGAGC
TACCAACTCTTTTTCCGAAGGTAACTGGCTTCAGCAGAGCGCAGATACCAAATACTGTCCTTCTAGTGTAGCCGTAGTTA
GGCCACCACTTCAAGAACTCTGTAGCACCGCCTACATACCTCGCTCTGCTAATCCTGTTACCAGTGGCTGCTGCCAGTGG
CGATAAGTCGTGTCTTACCGGGTTGGACTCAAGACGATAGTTACCGGATAAGGCGCAGCGGTCGGGCTGAACGGGGGGTT
CGTGCACACAGCCCAGCTTGGAGCGAACGACCTACACCGAACTGAGATACCTACAGCGTGAGCTATGAGAAAGCGCCACG
CTTCCCGAAGGGAGAAAGGCGGACAGGTATCCGGTAAGCGGCAGGGTCGGAACAGGAGAGCGCACGAGGGAGCTTCCAGG
GGGAAACGCCTGGTATCTTTATAGTCCTGTCGGGTTTCGCCACCTCTGACTTGAGCGTCGATTTTTGTGATGCTCGTCAG
GGGGGCGGAGCCTATGGAAAAACGCCAGCAACGCGGCCTTTTTACGGTTCCTGGCCTTTTGCTGGCCTTTTGCTCACATG
TTCTTTCCTGCGTTATCCCCTGATTCTGTGGATAACCGTATTACCGCCTTTGAGTGAGCTGATACCGCTCGCCGCAGCCG
AACGACCGAGCGCAGCGAGTCAGTGAGCGAGGAAGCGGAAGAGCGCCTGATGCGGTATTTTCTCCTTACGCATCTGTGCG
GTATTTCACACCGCATAGTAAAACGACGGCCAGTGAATTGTAATACGACTCACTATAGGGCGAATTGGAGCTTGGCGTAA
TCATGGTCAT
```

Supplementary Figure 20. Map and nucleotide sequence of expression plasmid AV44_pCAG.Cas9^{D10A}.gRNA^{S1}. CAG, hybrid promoter (CMV enhancer, human cytomegalovirus *immediate early* enhancer; chicken *β-actin* promoter; chimeric intron, fusion between introns from the chicken *β-actin* and rabbit *β-globin* genes); Cas9, human codon-optimized Cas9 (Csn1) ORF coding for the endonuclease nicking variant D10A from the *Streptococcus pyogenes* type II CRISPR/Cas system; SV40 NLS, nuclear localization signal SV40 large T antigen; HSV TK poly(A) signal, herpes simplex virus *thymidine kinase* polyadenylation signal; NeoR/KanR, transposon Tn5 *aminoglycoside phosphotransferase* conferring resistance to the antibiotics G418 (geneticin) and kanamycin; AmpR, *β-lactamase* ampicillin resistance gene; ori, high-copy number ColE1 prokaryotic origin of replication; U6 promoter, RNA polymerase III promoter for human snRNA; gRNA.S1, *AAVS1*-targeting gRNA^{S1} spacer; termination signal, RNA polymerase III termination signal.

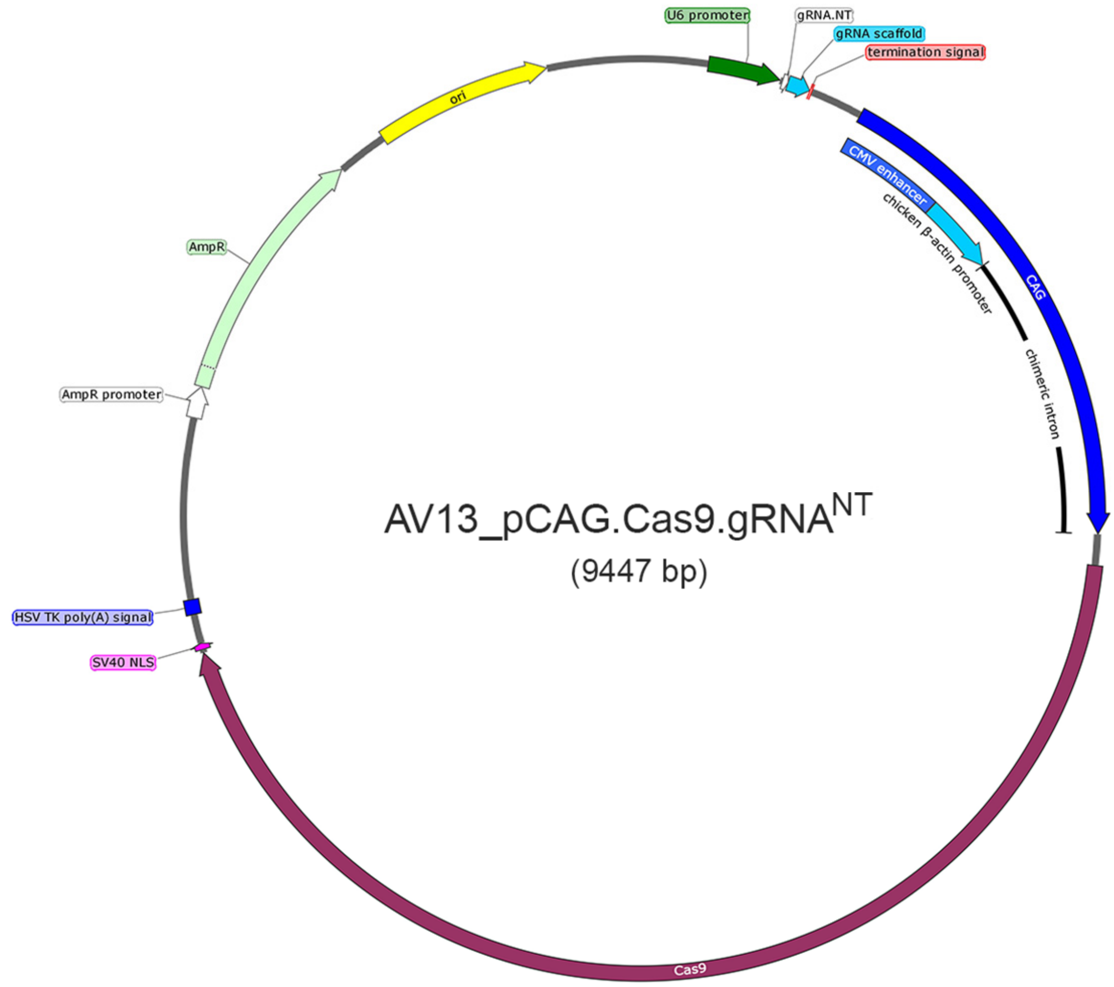

>AV13_pCAG.Cas9.gRNA^{NT} (9447 bp)
ATGTTATTCCTCCTTATTTAAAAGCTTAAGGTGCACGGCCCACGTGGCCACTAGTACTTCTCGAGCTCTGTACATGTCCG
CGGTCGCGACGTACGCGTATCGATGGCGCCAGCTGCAGGCGGCCGCCATATGCATCCTAGGCCTATTAATATTCCGGAGT
ATACGTAGCCGGCTAACGTTatcgcatgcctgcagagctctagagtcCGGCCGCCCCCTTCACCGAGGGCCTATTTCCCA
TGATTCCTTCATATTTGCATATACGATACAAGGCTGTTAGAGAGATAATTGGAATTAATTTGACTGTAAACACAAAGATA
TTAGTACAAAATACGTGACGTAGAAAGTAATAATTTCTTGGGTAGTTTGCAGTTTTAAAATTATGTTTTAAAATGGACTA
TCATATGCTTACCGTAACTTGAAAGTATTTCGATTTCTTGGCTTTATATATCTTGTGGAAAGGACGAAACACCGGTGAGC
TCTTATTTGCGTAGTTTTAGAGCTAGAAATAGCAAGTTAAAATAAGGCTAGTCCGTTATCAACTTGAAAAAGTGGCACCG
AGTCGGTGCTTTTTTTGaattAACAACCGGTACCTCTAGAACTATAGCTAGCATGCGCAAATTTAAAGCGCTGATATCGA
TCGCGCGCAGATCTGTCATGATGATCATTGCAATTGCATGAAGAATCTGCTTAGGGTTAGGCGTTTTGCGCTGCTTCGCG
ATGTACGGGCCAGATATACtcgagccatggattcGACATTGATTATTGACTAGTTATTAATAGTAATCAATTACGGGGTC
ATTAGTTCATAGCCCATATATGGAGTTCCGCGTTACATAACTTACGGTAAATGGCCCGCCTGGCTGACCGCCCAACGACC
CCCGCCCATTGACGTCAATAATGACGTATGTTCCCATAGTAACGCCAATAGGGACTTTCCATTGACGTCAATGGGTGGAG
TATTTACGGTAAACTGCCCACTTGGCAGTACATCAAGTGTATCATATGCCAAGTACGCCCCCTATTGACGTCAATGACGG
TAAATGGCCCGCCTGGCATTATGCCCAGTACATGACCTTATGGGACTTTCCTACTTGGCAGTACATCTACGTATTAGTCA
TCGCTATTACCATGGgTCGAGGTGAGCCCCACGTTCTGCTTCACTCTCCCCATCTCCCCCCCCTCCCCACCCCCAATTTT
GTATTTATTTATTTTTTAATTATTTTGTGCAGCGATGGGGGCGGGGGGGGGGGGGGCGCGCGCCAGGCGGGGCGGGGCGG
GGCGAGGGGCGGGGCGGGGCGAGGCGGAGAGGTGCGGCGGCAGCCAATCAGAGCGGCGCGCTCCGAAAGTTTCCTTTTAT
GGCGAGGCGGCGGCGGCGGCGGCCCTATAAAAAGCGAAGCGCGCGGCGGGCGGGAGTCGCTGCGTTGCCTTCGCCCCGTG
CCCCGCTCCGCGCCGCCTCGCGCCGCCCGCCCCGGCTCTGACTGACCGCGTTACTCCCACAGGTGAGCGGGCGGGACGGC
CCTTCTCCTCCGGGCTGTAATTAGCGCTTGGTTTAATGACGGCTCGTTTCTTTTCTGTGGCTGCGTGAAAGCCTTAAAGG
GCTCCGGGAGGGCCCTTTGTGCGGGGGGGAGCGGCTCGGGGGGTGCGTGCGTGTGTGTGTGCGTGGGGAGCGCCGCGTGC
GGCCCGCGCTGCCCGGCGGCTGTGAGCGCTGCGGGCGCGGCGCGGGGCTTTGTGCGCTCCGCGTGTGCGCGAGGGGAGCG

CGGCCGGGGGCGGTGCCCCGCGGTGCGGGGGGGCTGCGAGGGGAACAAAGGCTGCGTGCGGGGTGTGTGCGTGGGGGGGT
GAGCAGGGGGTGTGGGCGCGGCGGTCGGGCTGTAACCCCCCCCTGCACCCCCCTCCCCGAGTTGCTGAGCACGGCCCGGC
TTCGGGTGCGGGGCTCCGTGCGGGGCGTGGCGCGGGGCTCGCCGTGCCGGGCGGGGGGTGGCGGCAGGTGGGGGTGCCGG
GCGGGGCGGGGCCGCCTCGGGCCGGGGAGGGCTCGGGGGAGGGGCGCGGCGGCCCCGGAGCGCCGGCGGCTGTCGAGGCG
CGGCGAGCCGCAGCCATTGCCTTTTATGGTAATCGTGCGAGAGGGCGCAGGGACTTCCTTTGTCCCAAATCTGGCGGAGC
CGAAATCTGGGAGGCGCCGCCGCACCCCCTCTAGCGGGCGCGGGCGAAGCGGTGCGGCGCCGGCAGGAAGGAAATGGGCG
GGGAGGGCCTTCGTGCGTCGCCGCGCCGCCGTCCCCTTCTCCATCTCCAGCCTCGGGGCTGCCGCAGGGGGACGGCTGCC
TTCGGGGGGGACGGGGCAGGGCGGGGTTCGGCTTCTGGCGTGTGACCGGCGGCTCTAGAGCCTCTGCTAACCATGTTCAT
GCCTTCTTCTTTTTCCTACAGCTCCTGGGCAACGTGCTGGTTGTTGTGCTGTCTCATCATTTTGGCAAAGAATTatcgca
tgcctgcagagctctagagTCCTAGAGGATCGAACCCTTgccaccATGGACAAGAAGTACTCCATTGGGCTCGATATCGG
CACAAACAGCGTCGGCTGGGCCGTCATTACGGACGAGTACAAGGTGCCGAGCAAAAAATTCAAAGTTCTGGGCAATACCG
ATCGCCACAGCATAAAGAAGAACCTCATTGGCGCCCTCCTGTTCGACTCCGGGGAGACGGCCGAAGCCACGCGGCTCAAA
AGAACAGCACGGCGCAGATATACCCGCAGAAAGAATCGGATCTGCTACCTGCAGGAGATCTTTAGTAATGAGATGGCTAA
GGTGGATGACTCTTTCTTCCATAGGCTGGAGGAGTCCTTTTTGGTGGAGGAGGATAAAAAGCACGAGCGCCACCCAATCT
TTGGCAATATCGTGGACGAGGTGGCGTACCATGAAAAGTACCCAACCATATATCATCTGAGGAAGAAGCTTGTAGACAGT
ACTGATAAGGCTGACTTGCGGTTGATCTATCTCGCGCTGGCGCATATGATCAAATTTCGGGGACACTTCCTCATCGAGGG
GGACCTGAACCCAGACAACAGCGATGTCGACAAACTCTTTATCCAACTGGTTCAGACTTACAATCAGCTTTTCGAAGAGA
ACCCGATCAACGCATCCGGAGTTGACGCCAAAGCAATCCTGAGCGCTAGGCTGTCCAAATCCCGGCGGCTCGAAAACCTC
ATCGCACAGCTCCCTGGGGAGAAGAAGAACGGCCTGTTTGGTAATCTTATCGCCCTGTCACTCGGGCTGACCCCCAACTT
TAAATCTAACTTCGACCTGGCCGAAGATGCCAAGCTTCAACTGAGCAAAGACACCTACGATGATGATCTCGACAATCTGC
TGGCCCAGATCGGCGACCAGTACGCAGACCTTTTTTTGGCGGCAAAGAACCTGTCAGACGCCATTCTGCTGAGTGATATT
CTGCGAGTGAACACGGAGATCACCAAAGCTCCGCTGAGCGCTAGTATGATCAAGCGCTATGATGAGCACCACCAAGACTT
GACTTTGCTGAAGGCCCTTGTCAGACAGCAACTGCCTGAGAAGTACAAGGAAATTTTCTTCGATCAGTCTAAAAATGGCT
ACGCCGGATACATTGACGGCGGAGCAAGCCAGGAGGAATTTTACAAATTTATTAAGCCCATCTTGGAAAAAATGGACGGC
ACCGAGGAGCTGCTGGTAAAGCTTAACAGAGAAGATCTGTTGCGCAAACAGCGCACTTTCGACAATGGAAGCATCCCCCA
CCAGATTCACCTGGGCGAACTGCACGCTATCCTCAGGCGGCAAGAGGATTTCTACCCCTTTTTGAAAGATAACAGGGAAA
AGATTGAGAAAATCCTCACATTTCGGATACCCTACTATGTAGGCCCCCTCGCCCGGGGAAATTCCAGATTCGCGTGGATG
ACTCGCAAATCAGAAGAGACCATCACTCCCTGGAACTTCGAGGAAGTCGTGGATAAGGGGGCCTCTGCCCAGTCCTTCAT
CGAAAGGATGACTAACTTTGATAAAAATCTGCCTAACGAAAAGGTGCTTCCTAAACACTCTCTGCTGTACGAGTACTTCA
CAGTTTATAACGAGCTCACCAAGGTCAAATACGTCACAGAAGGGATGAGAAAGCCAGCATTCCTGTCTGGAGAGCAGAAG
AAAGCTATCGTGGACCTCCTCTTCAAGACGAACCGGAAAGTTACCGTGAAACAGCTCAAAGAAGACTATTTCAAAAAGAT
TGAATGTTTCGACTCTGTTGAAATCAGCGGAGTGGAGGATCGCTTCAACGCATCCCTGGGAACGTATCACGATCTCCTGA
AAATCATTAAAGACAAGGACTTCCTGGACAATGAGGAGAACGAGGACATTCTTGAGGACATTGTCCTCACCCTTACGTTG
TTTGAAGATAGGGAGATGATTGAAGAACGCTTGAAAACTTACGCTCATCTCTTCGACGACAAAGTCATGAAACAGCTCAA
GAGGCGCCGATATACAGGATGGGGGCGGCTGTCAAGAAAACTGATCAATGGGATCCGAGACAAGCAGAGTGGAAAGACAA
TCCTGGATTTTCTTAAGTCCGATGGATTTGCCAACCGGAACTTCATGCAGTTGATCCATGATGACTCTCTCACCTTTAAG
GAGGACATCCAGAAAGCACAAGTTTCTGGCCAGGGGGACAGTCTTCACGAGCACATCGCTAATCTTGCAGGTAGCCCAGC
TATCAAAAAGGGAATACTGCAGACCGTTAAGGTCGTGGATGAACTCGTCAAAGTAATGGGAAGGCATAAGCCCGAGAATA
TCGTTATCGAGATGGCCCGAGAGAACCAAACTACCCAGAAGGGACAGAAGAACAGTAGGGAAAGGATGAAGAGGATTGAA
GAGGGTATAAAAGAACTGGGGTCCCAAATCCTTAAGGAACACCCAGTTGAAAACACCCAGCTTCAGAATGAGAAGCTCTA
CCTGTACTACCTGCAGAACGGCAGGGACATGTACGTGGATCAGGAACTGGACATCAATCGGCTCTCCGACTACGACGTGG
ATCATATCGTGCCCCAGTCTTTTCTCAAAGATGATTCTATTGATAATAAAGTGTTGACAAGATCCGATAAAAATAGAGGG
AAGAGTGATAACGTCCCCTCAGAAGAAGTTGTCAAGAAAATGAAAAATTATTGGCGGCAGCTGCTGAACGCCAAACTGAT
CACACAACGGAAGTTCGATAATCTGACTAAGGCTGAACGAGGTGGCCTGTCTGAGTTGGATAAAGCCGGCTTCATCAAAA
GGCAGCTTGTTGAGACACGCCAGATCACCAAGCACGTGGCCCAAATTCTCGATTCACGCATGAACACCAAGTACGATGAA
AATGACAAACTGATTCGAGAGGTGAAAGTTATTACTCTGAAGTCTAAGCTGGTCTCAGATTTCAGAAAGGACTTTCAGTT
TTATAAGGTGAGAGAGATCAACAATTACCACCATGCGCATGATGCCTACCTGAATGCAGTGGTAGGCACTGCACTTATCA
AAAAATATCCCAAGCTTGAATCTGAATTTGTTTACGGAGACTATAAAGTGTACGATGTTAGGAAAATGATCGCAAAGTCT
GAGCAGGAAATAGGCAAGGCCACCGCTAAGTACTTCTTTTACAGCAATATTATGAATTTTTTCAAGACCGAGATTACACT
GGCCAATGGAGAGATTCGGAAGCGACCACTTATCGAAACAAACGGAGAAACAGGAGAAATCGTGTGGGACAAGGGTAGGG
ATTTCGCGACAGTCCGGAAGGTCCTGTCCATGCCGCAGGTGAACATCGTTAAAAAGACCGAAGTACAGACCGGAGGCTTC
TCCAAGGAAAGTATCCTCCCGAAAAGGAACAGCGACAAGCTGATCGCACGCAAAAAAGATTGGGACCCCAAGAAATACGG
CGGATTCGATTCTCCTACAGTCGCTTACAGTGTACTGGTTGTGGCCAAAGTGGAGAAAGGGAAGTCTAAAAAACTCAAAA
GCGTCAAGGAACTGCTGGGCATCACAATCATGGAGCGATCAAGCTTCGAAAAAAACCCCATCGACTTTCTCGAGGCGAAA
GGATATAAAGAGGTCAAAAAAGACCTCATCATTAAGCTTCCCAAGTACTCTCTCTTTGAGCTTGAAAACGGCCGGAAACG
AATGCTCGCTAGTGCGGGCGAGCTGCAGAAAGGTAACGAGCTGGCACTGCCCTCTAAATACGTTAATTTCTTGTATCTGG
CCAGCCACTATGAAAAGCTCAAAGGGTCTCCCGAAGATAATGAGCAGAAGCAGCTGTTCGTGGAACAACACAAACACTAC
CTTGATGAGATCATCGAGCAAATAAGCGAATTCTCCAAAAGAGTGATCCTCGCCGACGCTAACCTCGATAAGGTGCTTTC
TGCTTACAATAAGCACAGGGATAAGCCCATCAGGGAGCAGGCAGAAAACATTATCCACTTGTTTACTCTGACCAACTTGG
GCGCGCCTGCAGCCTTCAAGTACTTCGACACCACCATAGACAGAAAGCGGTACACCTCTACAAAGGAGGTCCTGGACGCC
ACACTGATTCATCAGTCAATTACGGGGCTCTATGAAACAAGAATCGACCTCTCTCAGCTCGGTGGAGACAGCAGGGCTGA
CCCCAAGAAGAAGAGGAAGGTGTGAAAGGGTTCGATCCCTACCGGTTAGTAATGAGTTTAAACGGGGGAGGCTAACTGAA
ACACGGAAGGAGACAATACCGGAAGGAACCCGCGCTATGACGGCAATAAAAAGACAGAATAAAACGCACGGGTGTTGGGT
CGTTTGTTCATAAACGCGGGGTTCGGTCCCAGGGCTGGCACTCTGTCGATACCCCACCGAGACCCCATTGGGGCCAATAC
GCCCGCGTTTCTTCCTTTTCCCCACCCCACCCCCCAAGTTCGGGTGAAGGCCCAGGGCTCGCAGCCAACGTCGGGGCGGC
AGGCCCTGCCATAGCAGATCTGCGCAGCTGGGGCTCTAGGGGGTATCCCCACGCGCCCTGTAGCGGCGCATTAAGCGCGG
CGGGTGTGGTGGTTACGCGCAGCGTGACCGCTACACTTGCCAGCGCCCTAGCGCCCGGGGTTATAATTACCTCAGGTCGA
CGTCCCATGTGCAGGTGCTGAATTCGAAGGCATTCCACGACGTGGTCACCAGCCATGGCCATGTCCAACTCCATCAAATC

```
AGCTTGAGTAGCCATGCCCATGGCTCCAGCCTGTCCTCGTACCAGCTTTGTTCCCTTTAGTGAGGGGTAATTTCGAGCTT
GGCGTAATCATGGTCATAGCTGTTTCCaattCTTGAAGACGAAAGGGCCTCGTGATACGCCTATTTTTATAGGTTAATGT
CATGATAATAATGGTTTCTTAGACGTCAGGTGGCACTTTTCGGGGAAATGTGCGCGGAACCCCTATTTGTTTATTTTTCT
AAATACATTCAAATATGTATCCGCTCATGAGACAATAACCCTGATAAATGCTTCAATAATATTGAAAAAGGAAGAGTATG
AGTATTCAACATTTCCGTGTCGCCCTTATTCCCTTTTTTGCGGCATTTTGCCTTCCTGTTTTTGCTCACCCAGAAACGCT
GGTGAAAGTAAAAGATGCTGAAGATCAGTTGGGTGCACGAGTGGGTTACATCGAACTGGATCTCAACAGCGGTAAGATCC
TTGAGAGTTTTCGCCCCGAAGAACGTTTTCCAATGATGAGCACTTTTAAAGTTCTGCTATGTGGCGCGGTATTATCCCGT
GTTGACGCCGGGCAAGAGCAACTCGGTCGCCGCATACACTATTCTCAGAATGACTTGGTTGAGTACTCACCAGTCACAGA
AAAGCATCTTACGGATGGCATGACAGTAAGAGAATTATGCAGTGCTGCCATAACCATGAGTGATAACACTGCGGCCAACT
TACTTCTGACAACGATCGGAGGACCGAAGGAGCTAACCGCTTTTTTGCACAACATGGGGGATCATGTAACTCGCCTTGAT
CGTTGGGAACCGGAGCTGAATGAAGCCATACCAAACGACGAGCGTGACACCACGATGCCTGCAGCAATGGCAACAACGTT
GCGCAAACTATTAACTGGCGAACTACTTACTCTAGCTTCCCGGCAACAATTAATAGACTGGATGGAGGCGGATAAAGTTG
CAGGACCACTTCTGCGCTCGGCCCTTCCGGCTGGCTGGTTTATTGCTGATAAATCTGGAGCCGGTGAGCGTGGGTCTCGC
GGTATCATTGCAGCACTGGGGCCAGATGGTAAGCCCTCCCGTATCGTAGTTATCTACACGACGGGGAGTCAGGCAACTAT
GGATGAACGAAATAGACAGATCGCTGAGATAGGTGCCTCACTGATTAAGCATTGGTAACTGTCAGACCAAGTTTACTCAT
ATATACTTTAGATTGATTTAAAACTTCATTTTTAATTTAAAAGGATCTAGGTGAAGATCCTTTTTGATAATCTCATGACC
AAAATCCCTTAACGTGAGTTTTCGTTCCACTGAGCGTCAGACCCCGTAGAAAAGATCAAAGGATCTTCTTGAGATCCTTT
TTTTCTGCGCGTAATCTGCTGCTTGCAAACAAAAAAACCACCGCTACCAGCGGTGGTTTGTTTGCCGGATCAAGAGCTAC
CAACTCTTTTTCCGAAGGTAACTGGCTTCAGCAGAGCGCAGATACCAAATACTGTCCTTCTAGTGTAGCCGTAGTTAGGC
CACCACTTCAAGAACTCTGTAGCACCGCCTACATACCTCGCTCTGCTAATCCTGTTACCAGTGGCTGCTGCCAGTGGCGA
TAAGTCGTGTCTTACCGGGTTGGACTCAAGACGATAGTTACCGGATAAGGCGCAGCGGTCGGGCTGAACGGGGGGTTCGT
GCACACAGCCCAGCTTGGAGCGAACGACCTACACCGAACTGAGATACCTACAGCGTGAGCTATGAGAAAGCGCCACGCTT
CCCGAAGGGAGAAAGGCGGACAGGTATCCGGTAAGCGGCAGGGTCGGAACAGGAGAGCGCACGAGGGAGCTTCCAGGGGG
AAACGCCTGGTATCTTTATAGTCCTGTCGGGTTTCGCCACCTCTGACTTGAGCGTCGATTTTTGTGATGCTCGTCAGGGG
GGCGGAGCCTATGGAAAAACGCCAGCAACGCGGCCTTTTTACGGTTCCTGGCCTTTTGCTGGCCTTTTGCTCACATGTTC
TTTCCTGCGTTATCCCCTGATTCTGTGGATAACCGTATTACCGCCTTTGAGTGAGCTGATACCGCTCGCCGCAGCCGAAC
GACCGAGCGCAGCGAGTCAGTGAGCGAGGAAGCGGAAGAGCGCCTGATGCGGTATTTTCTCCTTACGCATCTGTGCGGTA
TTTCACACCGCATAGTAAAACGACGGCCAGTGAATTGTAATACGACTCACTATAGGGCGAATTGGAGCTTGGCGTAATCA
TGGTCAT
```

Supplementary Figure 21. Map and nucleotide sequence of expression plasmid AV13_pCAG.Cas9gRNA^{NT}. CAG, hybrid promoter (CMV enhancer, human cytomegalovirus *immediate early* enhancer; chicken *β-actin* promoter; chimeric intron, fusion between introns from the chicken *β-actin* and rabbit *β-globin* genes); Cas9, human codon-optimized Cas9 (Csn1) ORF coding for the endonuclease from the *Streptococcus pyogenes* type II CRISPR/Cas system; SV40 NLS, nuclear localization signal SV40 large T antigen; HSV TK poly(A) signal, herpes simplex virus *thymidine kinase* polyadenylation signal; NeoR/KanR, transposon Tn5 *aminoglycoside phosphotransferase* conferring resistance to the antibiotics G418 (geneticin) and kanamycin; AmpR, *β-lactamase* ampicillin resistance gene; ori, high-copy number ColE1 prokaryotic origin of replication; U6 promoter, RNA polymerase III promoter for human snRNA; gRNA.NT, gRNA^{NT} spacer targeting Cas9 to the recognition site of the *S. cerevisiae* I-SceI homing endonuclease; termination signal, RNA polymerase III termination signal.

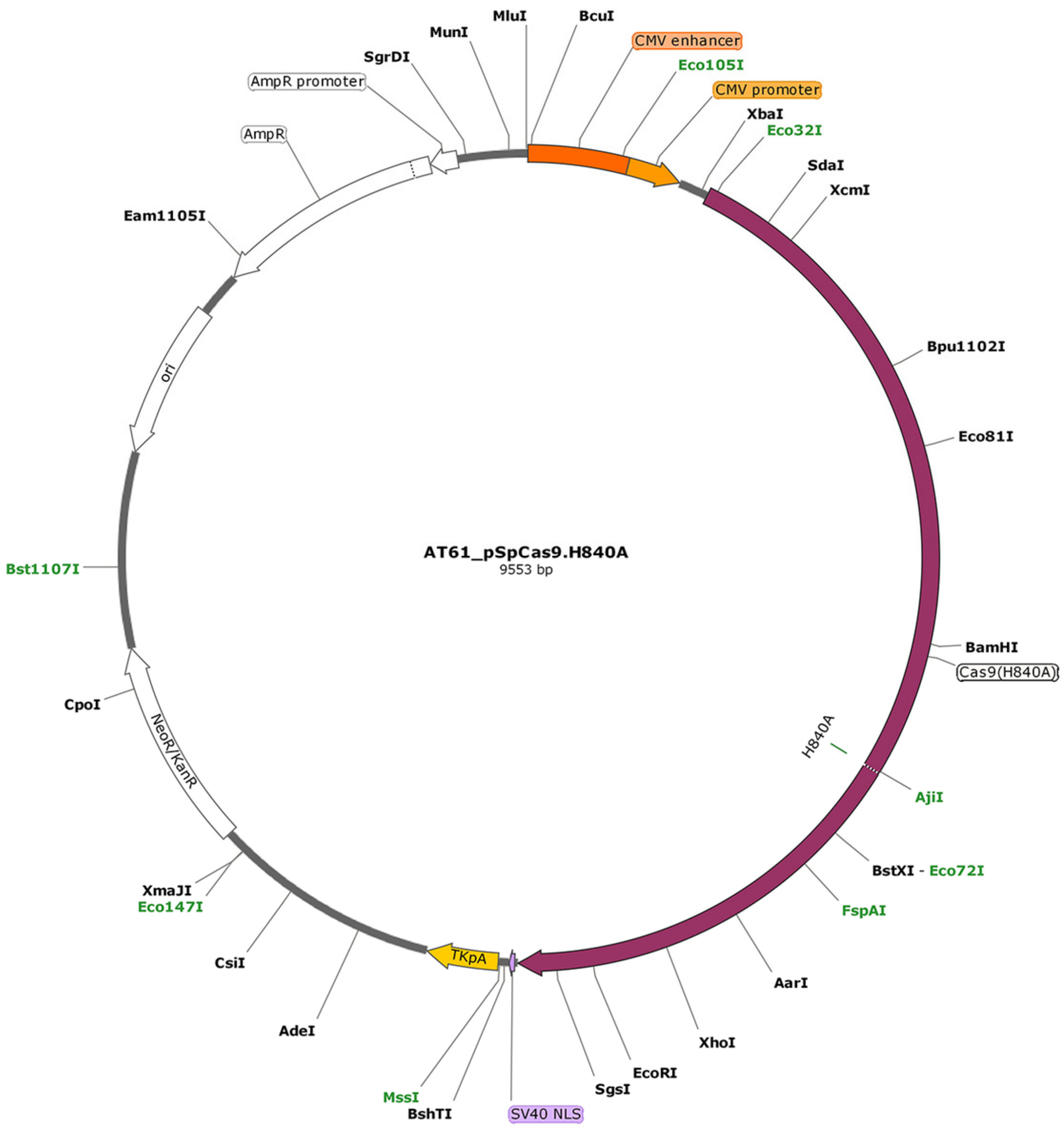

>AT61_pSpCas9^{H840A}

CGCGTTGACATTGATTATTGACTAGTTATTAATAGTAATCAATTACGGGGTCATTAGTTCATAGCCCATATATGGAGTTCCGC
GTTACATAACTTACGGTAAATGGCCCGCCTGGCTGACCGCCCAACGACCCCCGCCCATTGACGTCAATAATGACGTATGTTCC
CATAGTAACGCCAATAGGGACTTTCCATTGACGTCAATGGGTGGAGTATTTACGGTAAACTGCCCACTTGGCAGTACATCAAG
TGTATCATATGCCAAGTACGCCCCCTATTGACGTCAATGACGGTAAATGGCCCGCCTGGCATTATGCCCAGTACATGACCTTA
TGGGACTTTCCTACTTGGCAGTACATCTACGTATTAGTCATCGCTATTACCATGGTGATGCGGTTTTGGCAGTACATCAATGG
GCGTGGATAGCGGTTTGACTCACGGGGATTTCCAAGTCTCCACCCCATTGACGTCAATGGGAGTTTGTTTTGGCACCAAAATC
AACGGGACTTTCCAAAATGTCGTAACAACTCCGCCCCATTGACGCAAATGGGCGGTAGGCGTGTACGGTGGGAGGTCTATATA
AGCAGAGCTCGTTTAGTGAACCGTCAGATCGCCTGGAGACGCCATCCACGCTGTTTTGACCTCCATAGAAGACACCGGGACCG
ATCCAGCCTCCGGACTCTAGAGGATCGAACCCTTgccaccATGGACAAGAAGTACTCCATTGGGCTCGATATCGGCACAAACA
GCGTCGGCTGGGCCGTCATTACGGACGAGTACAAGGTGCCGAGCAAAAAATTCAAAGTTCTGGGCAATACCGATCGCCACAGC
ATAAAGAAGAACCTCATTGGCGCCCTCCTGTTCGACTCCGGGGAGACGGCCGAAGCCACGCGGCTCAAAAGAACAGCACGGCG
CAGATATACCCGCAGAAAGAATCGGATCTGCTACCTGCAGGAGATCTTTAGTAATGAGATGGCTAAGGTGGATGACTCTTTCT
TCCATAGGCTGGAGGAGTCCTTTTTGGTGGAGGAGGATAAAAAGCACGAGCGCCACCCAATCTTTGGCAATATCGTGGACGAG
GTGGCGTACCATGAAAAGTACCCAACCATATATCATCTGAGGAAGAAGCTTGTAGACAGTACTGATAAGGCTGACTTGCGGTT
GATCTATCTCGCGCTGGCGCATATGATCAAATTTCGGGGACACTTCCTCATCGAGGGGGACCTGAACCCAGACAACAGCGATG
TCGACAAACTCTTTATCCAACTGGTTCAGACTTACAATCAGCTTTTCGAAGAGAACCCGATCAACGCATCCGGAGTTGACGCC
AAAGCAATCCTGAGCGCTAGGCTGTCCAAATCCCGGCGGCTCGAAAACCTCATCGCACAGCTCCCTGGGGAGAAGAAGAACGG

CCTGTTTGGTAATCTTATCGCCCTGTCACTCGGGCTGACCCCCAACTTTAAATCTAACTTCGACCTGGCCGAAGATGCCAAGC
TTCAACTGAGCAAAGACACCTACGATGATGATCTCGACAATCTGCTGGCCCAGATCGGCGACCAGTACGCAGACCTTTTTTTG
GCGGCAAAGAACCTGTCAGACGCCATTCTGCTGAGTGATATTCTGCGAGTGAACACGGAGATCACCAAAGCTCCGCTGAGCGC
TAGTATGATCAAGCGCTATGATGAGCACCACCAAGACTTGACTTTGCTGAAGGCCCTTGTCAGACAGCAACTGCCTGAGAAGT
ACAAGGAAATTTTCTTCGATCAGTCTAAAAATGGCTACGCCGGATACATTGACGGCGGAGCAAGCCAGGAGGAATTTTACAAA
TTTATTAAGCCCATCTTGGAAAAAATGGACGGCACCGAGGAGCTGCTGGTAAAGCTTAACAGAGAAGATCTGTTGCGCAAACA
GCGCACTTTCGACAATGGAAGCATCCCCCACCAGATTCACCTGGGCGAACTGCACGCTATCCTCAGGCGGCAAGAGGATTTCT
ACCCCTTTTTGAAAGATAACAGGGAAAAGATTGAGAAAATCCTCACATTTCGGATACCCTACTATGTAGGCCCCCTCGCCCGG
GGAAATTCCAGATTCGCGTGGATGACTCGCAAATCAGAAGAGACCATCACTCCCTGGAACTTCGAGGAAGTCGTGGATAAGGG
GGCCTCTGCCCAGTCCTTCATCGAAAGGATGACTAACTTTGATAAAAATCTGCCTAACGAAAAGGTGCTTCCTAAACACTCTC
TGCTGTACGAGTACTTCACAGTTTATAACGAGCTCACCAAGGTCAAATACGTCACAGAAGGGATGAGAAAGCCAGCATTCCTG
TCTGGAGAGCAGAAGAAAGCTATCGTGGACCTCCTCTTCAAGACGAACCGGAAAGTTACCGTGAAACAGCTCAAAGAAGACTA
TTTCAAAAAGATTGAATGTTTCGACTCTGTTGAAATCAGCGGAGTGGAGGATCGCTTCAACGCATCCCTGGGAACGTATCACG
ATCTCCTGAAAATCATTAAAGACAAGGACTTCCTGGACAATGAGGAGAACGAGGACATTCTTGAGGACATTGTCCTCACCCTT
ACGTTGTTTGAAGATAGGGAGATGATTGAAGAACGCTTGAAAACTTACGCTCATCTCTTCGACGACAAAGTCATGAAACAGCT
CAAGAGGCGCCGATATACAGGATGGGGGCGGCTGTCAAGAAAACTGATCAATGGGATCCGAGACAAGCAGAGTGGAAAGACAA
TCCTGGATTTTCTTAAGTCCGATGGATTTGCCAACCGGAACTTCATGCAGTTGATCCATGATGACTCTCTCACCTTTAAGGAG
GACATCCAGAAAGCACAAGTTTCTGGCCAGGGGGACAGTCTTCACGAGCACATCGCTAATCTTGCAGGTAGCCCAGCTATCAA
AAAGGGAATACTGCAGACCGTTAAGGTCGTGGATGAACTCGTCAAAGTAATGGGAAGGCATAAGCCCGAGAATATCGTTATCG
AGATGGCCCGAGAGAACCAAACTACCCAGAAGGGACAGAAGAACAGTAGGGAAAGGATGAAGAGGATTGAAGAGGGTATAAAA
GAACTGGGGTCCCAAATCCTTAAGGAACACCCAGTTGAAAACACCCAGCTTCAGAATGAGAAGCTCTACCTGTACTACCTGCA
GAACGGCAGGGACATGTACGTGGATCAGGAACTGGACATCAATCGGCTCTCCGACTACGACGTGGATGCTATCGTCCCCCAGT
CTTTTCTCAAAGATGATTCTATTGATAATAAAGTGTTGACAAGATCCGATAAAAATAGAGGGAAGAGTGATAACGTCCCCTCA
GAAGAAGTTGTCAAGAAAATGAAAAATTATTGGCGGCAGCTGCTGAACGCCAAACTGATCACACAACGGAAGTTCGATAATCT
GACTAAGGCTGAACGAGGTGGCCTGTCTGAGTTGGATAAAGCCGGCTTCATCAAAAGGCAGCTTGTTGAGACACGCCAGATCA
CCAAGCACGTGGCCCAAATTCTCGATTCACGCATGAACACCAAGTACGATGAAAATGACAAACTGATTCGAGAGGTGAAAGTT
ATTACTCTGAAGTCTAAGCTGGTCTCAGATTTCAGAAAGGACTTTCAGTTTTATAAGGTGAGAGAGATCAACAATTACCACCA
TGCGCATGATGCCTACCTGAATGCAGTGGTAGGCACTGCACTTATCAAAAAATATCCCAAGCTTGAATCTGAATTTGTTTACG
GAGACTATAAAGTGTACGATGTTAGGAAAATGATCGCAAAGTCTGAGCAGGAAATAGGCAAGGCCACCGCTAAGTACTTCTTT
TACAGCAATATTATGAATTTTTTCAAGACCGAGATTACACTGGCCAATGGAGAGATTCGGAAGCGACCACTTATCGAAACAAA
CGGAGAAACAGGAGAAATCGTGTGGGACAAGGGTAGGGATTTCGCGACAGTCCGGAAGGTCCTGTCCATGCCGCAGGTGAACA
TCGTTAAAAAGACCGAAGTACAGACCGGAGGCTTCTCCAAGGAAAGTATCCTCCCGAAAAGGAACAGCGACAAGCTGATCGCA
CGCAAAAAAGATTGGGACCCCAAGAAATACGGCGGATTCGATTCTCCTACAGTCGCTTACAGTGTACTGGTTGTGGCCAAAGT
GGAGAAAGGGAAGTCTAAAAAACTCAAAAGCGTCAAGGAACTGCTGGGCATCACAATCATGGAGCGATCAAGCTTCGAAAAAA
ACCCCATCGACTTTCTCGAGGCGAAAGGATATAAAGAGGTCAAAAAAGACCTCATCATTAAGCTTCCCAAGTACTCTCTCTTT
GAGCTTGAAAACGGCCGGAAACGAATGCTCGCTAGTGCGGGCGAGCTGCAGAAAGGTAACGAGCTGGCACTGCCCTCTAAATA
CGTTAATTTCTTGTATCTGGCCAGCCACTATGAAAAGCTCAAAGGGTCTCCCGAAGATAATGAGCAGAAGCAGCTGTTCGTGG
AACAACACAAACACTACCTTGATGAGATCATCGAGCAAATAAGCGAATTCTCCAAAAGAGTGATCCTCGCCGACGCTAACCTC
GATAAGGTGCTTTCTGCTTACAATAAGCACAGGGATAAGCCCATCAGGGAGCAGGCAGAAAACATTATCCACTTGTTTACTCT
GACCAACTTGGGCGCGCCTGCAGCCTTCAAGTACTTCGACACCACCATAGACAGAAAGCGGTACACCTCTACAAAGGAGGTCC
TGGACGCCACACTGATTCATCAGTCAATTACGGGGCTCTATGAAACAAGAATCGACCTCTCTCAGCTCGGTGGAGACAGCAGG
GCTGACCCCAAGAAGAAGAGGAAGGTGTGAAAGGGTTCGATCCCTACCGGTTAGTAATGAGTTTAAACGGGGGAGGCTAACTG
AAACACGGAAGGAGACAATACCGGAAGGAACCCGCGCTATGACGGCAATAAAAAGACAGAATAAAACGCACGGGTGTTGGGTC
GTTTGTTCATAAACGCGGGGTTCGGTCCCAGGGCTGGCACTCTGTCGATACCCCACCGAGACCCCATTGGGGCCAATACGCCC
GCGTTTCTTCCTTTTCCCCACCCCACCCCCCAAGTTCGGGTGAAGGCCCAGGGCTCGCAGCCAACGTCGGGGCGGCAGGCCCT
GCCATAGCAGATCTGCGCAGCTGGGGCTCTAGGGGGTATCCCCACGCGCCCTGTAGCGGCGCATTAAGCGCGGCGGGTGTGGT
GGTTACGCGCAGCGTGACCGCTACACTTGCCAGCGCCCTAGCGCCCGCTCCTTTCGCTTTCTTCCCTTCCTTTCTCGCCACGT
TCGCCGGCTTTCCCCGTCAAGCTCTAAATCGGGGGCTCCCTTTAGGGTTCCGATTTAGTGCTTTACGGCACCTCGACCCCAAA
AAACTTGATTAGGGTGATGGTTCACGTAGTGGGCCATCGCCCTGATAGACGGTTTTTCGCCCTTTGACGTTGGAGTCCACGTT
CTTTAATAGTGGACTCTTGTTCCAAACTGGAACAACACTCAACCCTATCTCGGTCTATTCTTTTGATTTATAAGGGATTTTGC
CGATTTCGGCCTATTGGTTAAAAAATGAGCTGATTTAACAAAAATTTAACGCGAATTAATTCTGTGGAATGTGTGTCAGTTAG
GGTGTGGAAAGTCCCCAGGCTCCCCAGCAGGCAGAAGTATGCAAAGCATGCATCTCAATTAGTCAGCAACCAGGTGTGGAAAG
TCCCCAGGCTCCCCAGCAGGCAGAAGTATGCAAAGCATGCATCTCAATTAGTCAGCAACCATAGTCCCGCCCCTAACTCCGCC
CATCCCGCCCCTAACTCCGCCCAGTTCCGCCCATTCTCCGCCCCATGGCTGACTAATTTTTTTTATTTATGCAGAGGCCGAGG
CCGCCTCTGCCTCTGAGCTATTCCAGAAGTAGTGAGGAGGCTTTTTTGGAGGCCTAGGCTTTTGCAAAAAGCTCCCGGGAGCT
TGTATATCCATTTTCGGATCTGATCAAGAGACAGGATGAGGATCGTTTCGCATGATTGAACAAGATGGATTGCACGCAGGTTC
TCCGGCCGCTTGGGTGGAGAGGCTATTCGGCTATGACTGGGCACAACAGACAATCGGCTGCTCTGATGCCGCCGTGTTCCGGC
TGTCAGCGCAGGGGCGCCCGGTTCTTTTTGTCAAGACCGACCTGTCCGGTGCCCTGAATGAACTGCAGGACGAGGCAGCGCGG
CTATCGTGGCTGGCCACGACGGGCGTTCCTTGCGCAGCTGTGCTCGACGTTGTCACTGAAGCGGGAAGGGACTGGCTGCTATT
GGGCGAAGTGCCGGGGCAGGATCTCCTGTCATCTCACCTTGCTCCTGCCGAGAAAGTATCCATCATGGCTGATGCAATGCGGC
GGCTGCATACGCTTGATCCGGCTACCTGCCCATTCGACCACCAAGCGAAACATCGCATCGAGCGAGCACGTACTCGGATGGAA
GCCGGTCTTGTCGATCAGGATGATCTGGACGAAGAGCATCAGGGGCTCGCGCCAGCCGAACTGTTCGCCAGGCTCAAGGCGCG
CATGCCCGACGGCGAGGATCTCGTCGTGACCCATGGCGATGCCTGCTTGCCGAATATCATGGTGGAAAATGGCCGCTTTTCTG
GATTCATCGACTGTGGCCGGCTGGGTGTGGCGGACCGCTATCAGGACATAGCGTTGGCTACCCGTGATATTGCTGAAGAGCTT
GGCGGCGAATGGGCTGACCGCTTCCTCGTGCTTTACGGTATCGCCGCTCCCGATTCGCAGCGCATCGCCTTCTATCGCCTTCT
TGACGAGTTCTTCTGAGCGGGACTCTGGGGTTCGCGAAATGACCGACCAAGCGACGCCCAACCTGCCATCACGAGATTTCGAT
TCCACCGCCGCCTTCTATGAAAGGTTGGGCTTCGGAATCGTTTTCCGGGACGCCGGCTGGATGATCCTCCAGCGCGGGGATCT
CATGCTGGAGTTCTTCGCCCACCCCAACTTGTTTATTGCAGCTTATAATGGTTACAAATAAAGCAATAGCATCACAAATTTCA

CAAATAAAGCATTTTTTTCACTGCATTCTAGTTGTGGTTTGTCCAAACTCATCAATGTATCTTATCATGTCTGTATACCGTCG
ACCTCTAGCTAGAGCTTGGCGTAATCATGGTCATAGCTGTTTCCTGTGTGAAATTGTTATCCGCTCACAATTCCACACAACAT
ACGAGCCGGAAGCATAAAGTGTAAAGCCTGGGGTGCCTAATGAGTGAGCTAACTCACATTAATTGCGTTGCGCTCACTGCCCG
CTTTCCAGTCGGGAAACCTGTCGTGCCAGCTGCATTAATGAATCGGCCAACGCGCGGGGAGAGGCGGTTTGCGTATTGGGCGC
TCTTCCGCTTCCTCGCTCACTGACTCGCTGCGCTCGGTCGTTCGGCTGCGGCGAGCGGTATCAGCTCACTCAAAGGCGGTAAT
ACGGTTATCCACAGAATCAGGGGATAACGCAGGAAAGAACATGTGAGCAAAAGGCCAGCAAAAGGCCAGGAACCGTAAAAAGG
CCGCGTTGCTGGCGTTTTTCCATAGGCTCCGCCCCCCTGACGAGCATCACAAAAATCGACGCTCAAGTCAGAGGTGGCGAAAC
CCGACAGGACTATAAAGATACCAGGCGTTTCCCCCTGGAAGCTCCCTCGTGCGCTCTCCTGTTCCGACCCTGCCGCTTACCGG
ATACCTGTCCGCCTTTCTCCCTTCGGGAAGCGTGGCGCTTTCTCATAGCTCACGCTGTAGGTATCTCAGTTCGGTGTAGGTCG
TTCGCTCCAAGCTGGGCTGTGTGCACGAACCCCCCGTTCAGCCCGACCGCTGCGCCTTATCCGGTAACTATCGTCTTGAGTCC
AACCCGGTAAGACACGACTTATCGCCACTGGCAGCAGCCACTGGTAACAGGATTAGCAGAGCGAGGTATGTAGGCGGTGCTAC
AGAGTTCTTGAAGTGGTGGCCTAACTACGGCTACACTAGAAGAACAGTATTTGGTATCTGCGCTCTGCTGAAGCCAGTTACCT
TCGGAAAAAGAGTTGGTAGCTCTTGATCCGGCAAACAAACCACCGCTGGTAGCGGTGGTTTTTTTGTTTGCAAGCAGCAGATT
ACGCGCAGAAAAAAAGGATCTCAAGAAGATCCTTTGATCTTTTCTACGGGGTCTGACGCTCAGTGGAACGAAAACTCACGTTA
AGGGATTTTGGTCATGAGATTATCAAAAAGGATCTTCACCTAGATCCTTTTAAATTAAAAATGAAGTTTTAAATCAATCTAAA
GTATATATGAGTAAACTTGGTCTGACAGTTACCAATGCTTAATCAGTGAGGCACCTATCTCAGCGATCTGTCTATTTCGTTCA
TCCATAGTTGCCTGACTCCCCGTCGTGTAGATAACTACGATACGGGAGGGCTTACCATCTGGCCCCAGTGCTGCAATGATACC
GCGAGACCCACGCTCACCGGCTCCAGATTTATCAGCAATAAACCAGCCAGCCGGAAGGGCCGAGCGCAGAAGTGGTCCTGCAA
CTTTATCCGCCTCCATCCAGTCTATTAATTGTTGCCGGGAAGCTAGAGTAAGTAGTTCGCCAGTTAATAGTTTGCGCAACGTT
GTTGCCATTGCTACAGGCATCGTGGTGTCACGCTCGTCGTTTGGTATGGCTTCATTCAGCTCCGGTTCCCAACGATCAAGGCG
AGTTACATGATCCCCCATGTTGTGCAAAAAAGCGGTTAGCTCCTTCGGTCCTCCGATCGTTGTCAGAAGTAAGTTGGCCGCAG
TGTTATCACTCATGGTTATGGCAGCACTGCATAATTCTCTTACTGTCATGCCATCCGTAAGATGCTTTTCTGTGACTGGTGAG
TACTCAACCAAGTCATTCTGAGAATAGTGTATGCGGCGACCGAGTTGCTCTTGCCCGGCGTCAATACGGGATAATACCGCGCC
ACATAGCAGAACTTTAAAAGTGCTCATCATTGGAAAACGTTCTTCGGGGCGAAAACTCTCAAGGATCTTACCGCTGTTGAGAT
CCAGTTCGATGTAACCCACTCGTGCACCCAACTGATCTTCAGCATCTTTTACTTTCACCAGCGTTTCTGGGTGAGCAAAAACA
GGAAGGCAAAATGCCGCAAAAAAGGGAATAAGGGCGACACGGAAATGTTGAATACTCATACTCTTCCTTTTTCAATATTATTG
AAGCATTTATCAGGGTTATTGTCTCATGAGCGGATACATATTTGAATGTATTTAGAAAAATAAACAAATAGGGGTTCCGCGCA
CATTTCCCCGAAAAGTGCCACCTGACGTCGACGGATCGGGAGATCTCCCGATCCCCTATGGTGCACTCTCAGTACAATCTGCT
CTGATGCCGCATAGTTAAGCCAGTATCTGCTCCCTGCTTGTGTGTTGGAGGTCGCTGAGTAGTGCGCGAGCAAAATTTAAGCT
ACAACAAGGCAAGGCTTGACCGACAATTGCATGAAGAATCTGCTTAGGGTTAGGCGTTTTGCGCTGCTTCGCGATGTACGGGC
CAGATATA

Supplementary Figure 22. Map and nucleotide sequence of expression plasmid AT61_pSpCas9^{H840A}. CMV, regulatory sequences from the human cytomegalovirus *immediate-early* gene; nicking mutant of the Cas9 nuclease from the *Streptococcus pyogenes* Type II CRISPR/Cas system (together with an appropriate gRNA generates sequence- and strand-specific DNA breaks owing to the H840A mutation in its HNH catalytic domain); SV40 NLS, nuclear localization signal from the simian virus 40 large T antigen; TKpA, polyadenylation signal from the herpes simplex virus type 1 *thymide kinase* gene; NeoR/KanR, gene coding for aminoglycoside phosphotransferase from transposon Tn5 (confers resistance to neomycin and kanamycin); AmpR, *β-lactamase* gene (confers resistance to ampicillin); Ori, prokaryotic ColE1 origin of replication.

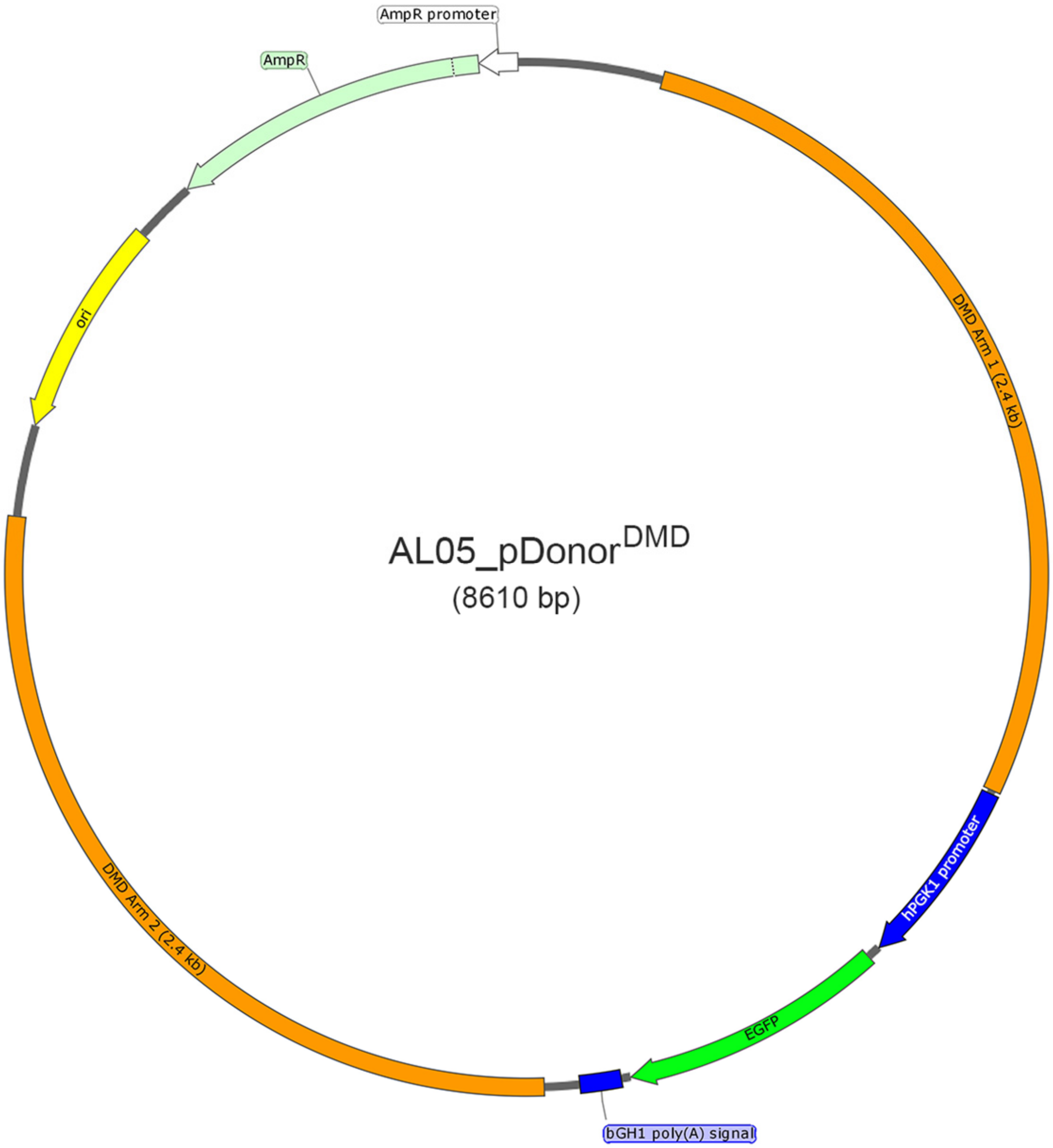


```
>AL05_pDonor.DMD  (8610 bp)
CTAAATTGTAAGCGTTAATATTTTGTTAAAATTCGCGTTAAATTTTTGTTAAATCAGCTCATTTTTTAACCAATAGGCCG
AAATCGGCAAAATCCCTTATAAATCAAAAGAATAGACCGAGATAGGGTTGAGTGGCCGCTACAGGGCGCTCCCATTCGCC
ATTCAGGCTGCGCAACTGTTGGGAAGGGCGTTTCGGTGCGGGCCTCTTCGCTATTACGCCAGCTGGCGAAAGGGGGATGT
GCTGCAAGGCGATTAAGTTGGGTAACGCCAGGGTTTTCCCAGTCACGACGTTGTAAAACGACGGCCAGTGAGCGCGACGT
AATACGACTCACTATAGGGCGAATTGGCGGAAGGCCGTCAAGGCCTAGGCCATAAGGTAAAGAGGACACCAAATACAGTA
AATAATAACATAATAATAAAGAAACCTTATGAATTCTATTCAGTGATATCCATATTGAGGTATTTAAGGTGAATGATACT
GATGTCAGCAGTTTACCTGGATATGCATCAAAAATATGATTGATTGATGGATGGATCGGAGGAAGGAAAAGATATGTGAT
CAAAACGAGTATAGTAAAATGTTCATGGTGGAATCTAGATATATAGATATCACTTGTAATTTTTAAAAATGTTATTGCAT
TTTTGAAATTTGTTATGTTAAAATATTGGGTGAAAATTCTGATGCCCTGGCCATGTCCCAAATCAGTTGAATCAGATCTC
TGGTGTATTTTTTTTTTTAAGATCCTTGGATGACTCCATTGTACAGCAAGTTTGCAAACCACTATTCTAACTTCTCACCAC
CCCCTTCTTTATAGCTTACAAAATTATATTTTTAAATGTCAAATGTCACAATTTCACTTTCCTTCTTCAAGAACTGATTA
GACTTCTAGCTTCTCCTATGCTCTCATCTTGCATAACGTTCATTTGCACGTGGTTAAAACTGATAGGCAATAAATAATAA
CCCATCTGTCTTTTCCTGACCCAACATAACTAAAACCTTCCTTTCAAGGCGAAAATGAACTATAATCTCACAGACAAGAG
TAGCTATAGACAACTAATTCTTTCTGGTCTCTCGTGGTCATGGGACTATTCACCACTGCCGTCCCTGCTGTCTATTTGGT
GGACCAGCCAGTTTTACTGTGACCCATACCATGGCTCTTGAGAAGCATGAAGCTGCCGAAATCGCAGTTGTAGGGTTAAT
GGAAAACATGGTGAAGTATAGCTTACATATATATACAATTTTGGCACAAAATGAAGCCTAAGAAATTGTACCGGGCTGGG
TGTGGTGACTCATGCCTGTAATCCCAGCACTTTGAGAGACCAAGGTGGGCGGATCACCTGAGGTCGGGAGTTCGAGACCA
GACTGAGCAACATGGAGAAACCCTCTCTACTTAAAATACAAAATTAGCCAGGCATGGTGGCGCATGCCTGTAACCCCAGC
```

TACTCAGGAGGCTAAGGCAAGAGAATCTCTTCCACCCTGGAGGCATAGGTTGTGGTGAGAGAAGATCACACCATTGCACT
CCAGCTGGGGCAACAAGAGCGAAGCTCCGTCTCAATAAATAAATAAATAAATAAATAAATAAATAAATAAATAAATAAAT
AAAAGAAAAAGAAATTGTACCAGGAAGTGGATTGGGAATGCTCTATATATTCTCCCCCTCCAAGAAGTTTTTTTAATTTA
TTGTATTGTGAAAGATCTGCAGCTATTTATTTTGTGTGGTACTCTTTTGCAATAAACTACTTCTGCATAAATGAACTCAA
AAACTTTGATATTCATGAACCTTCATAATGTTGATCTAGTACATCTTGCCCAACTAGCCCTTGGGCCATTCCTTCCATGC
CACTGGTTGAAAAATATGCTCCCCACCCCAGTAACATCAGCATCAGACCTCTGCAACCAGAAAGATTCCATAGGTAATGA
AATCCAAATGCTGGCATTTGGAAGTCAAACCAGTGTATTTTGAAATGGAAAGGCAAGGGGAAGTGCATGGAGCAAGTACA
GCATGTGCTACAACTTCAGTTCATACCCTCCTTAAAATACTGTGCAACTAAACTGTCAATTGCCCATTGATAGAGATAGT
ATCTTTTTCAGCTTTACACCTTCCGAATTCAACACACTGCTAGCCTCAGGACTAAGCGCTCAATAAATGTTTATCAAAAG
ATTGAGTAAATGAATCAGTGGTGAAATAAAATGTACAAACACATACGCTTCATACACGTTTAGGAAGCATTTCTAGTGAG
CTTTGTGGATATTTTATTTCTTAAAGGTTCTTTTTCTTTCCATGTTCATGTTATTCTTTATTTTTTAAAATGTTGCTTCT
GCTTATTTCTCTTTCTACGGCAGCTATATTTACTTGCCTGAGAGCACAGCATATACTGATGTTAATCAAACTTTAGATGA
AACACCTAAAATGATAGAAAAGAATCTGCTCTAATTAAATAATAAAGCAACCCCTACAGGTTTAGACATGTGCCTTCCGG
TGTGGGAAAGAAAAAATTTAATGAATGTAGTAGTTTTATGCCAAGAACATTTCCCTCGTGCACCTGTGTTTTAATAAGAT
AGAATATAAAATAGCAAAAGGGGCCCGACTTTTGTGATGATATTTACTCATAAGGTAGTAAGTCAGATGTGATGACATTT
TCTTTGACTTAGAGCTGCTATACTTGGGTCAGATTTCAGTTCAGTAAATTTGCAGTGAAGTTGTCTTTCTAACATGGTGT
CATCCTGGAACTGCCCTGCTCCCACAGTAAGCTTCCACGGGGTTGGGGTTGCGCCTTTTCCAAGGCAGCCCTGGGTTTGC
GCAGGGACGCGGCTGCTCTGGGCGTGGTTCCGGGAAACGCAGCGGCGCCGACCCTGGGTCTCGCACATTCTTCACGTCCG
TTCGCAGCGTCACCCGGATCTTCGCCGCTACCCTTGTGGGCCCCCCGGCGACGCTTCCTGCTCCGCCCCTAAGTCGGGAA
GGTTCCTTGCGGTTCGCGGCGTGCCGGACGTGACAAACGGAAGCCGCACGTCTCACTAGTACCCTCGCAGACGGACAGCG
CCAGGGAGCAATGGCAGCGCGCCGACCGCGATGGGCTGTGGCCAATAGCGGCTGCTCAGCAGGGCGCGCCGAGAGCAGCG
GCCGGGAAGGGGCGGTGCGGGAGGCGGGGTGTGGGGCGGTAGTGTGGGCCCTGTTCCTGCCCGCGCGGTGTTCCGCATTC
TGCAAGCCTCCGGAGCGCACGTCGGCAGTCGGCTCCCTCGTTGACCGAATCACCGACCTCTCTCCCCACACGACTCTAGA
GGATCCCCGGGTACCGGTCGCCACCATGGTGAGCAAGGGCGAGGAGCTGTTCACCGGGGTGGTGCCCATCCTGGTCGAGC
TGGACGGCGACGTAAACGGCCACAAGTTCAGCGTGTCCGGCGAGGGCGAGGGCGATGCCACCTACGGCAAGCTGACCCTG
AAGTTCATCTGCACCACCGGCAAGCTGCCCGTGCCCTGGCCCACCCTCGTGACCACCCTGACCTACGGCGTGCAGTGCTT
CAGCCGCTACCCCGACCACATGAAGCAGCACGACTTCTTCAAGTCCGCCATGCCCGAAGGCTACGTCCAGGAGCGCACCA
TCTTCTTCAAGGACGACGGCAACTACAAGACCCGCGCCGAGGTGAAGTTCGAGGGCGACACCCTGGTGAACCGCATCGAG
CTGAAGGGCATCGACTTCAAGGAGGACGGCAACATCCTGGGGCACAAGCTGGAGTACAACTACAACAGCCACAACGTCTA
TATCATGGCCGACAAGCAGAAGAACGGCATCAAGGTGAACTTCAAGATCCGCCACAACATCGAGGACGGCAGCGTGCAGC
TCGCCGACCACTACCAGCAGAACACCCCCATCGGCGACGGCCCCGTGCTGCTGCCCGACAACCACTACCTGAGCACCCAG
TCCGCCCTGAGCAAAGACCCCAACGAGAAGCGCGATCACATGGTCCTGCTGGAGTTCGTGACCGCCGCCGGGATCACTCT
CGGCATGGACGAGCTGTACAAGTAAAGCGGCCGCGACTCTAGAATTTTAAGCTGTGCCTTCTAGTTGCCAGCCATCTGTT
GTTTGCCCCTCCCCCGTGCCTTCCTTGACCCTGGAAGGTGCCACTCCCACTGTCCTTTCCTAATAAAATGAGGAAATTGC
ATCGCATTGTCTGAGTAGGTGTCATTCTATTCTGGGGGGTGGGGTGGGGCAGGACAGCAAGGGGGAGGATTGGGAAGACA
ATAGCAGGCATGCACGTGTACAGGCTAGGGAGTGGGTAGGAGTGGGGGTGAAATCCTCTTAATGTTTATGGTGTCAGTAG
ATTCAAACTAAAATTAGCCTTACAGCCATACTCCTAATAAGGGGCCCCTGGCATATTTAATTGATTTAACAAATTTATCA
AAAATAGATAAACTGAAATCTGCCTTGAAATTAATTACTGTATCTCTATTTTTATAAGAAAATATTTTGGACCCGTTCCC
TCTGCCTTATGGGTGCAACTCCCGGCAGAATAGGTCATGTCTCCTGAAAATAGTTCTTATTTTCTTTACTACTTATGACC
TCTTATAGCCTAGAAGTTTTTCCTTGCTATTTCTAGTTGAAATGCTAATCTGGCATAATTTCTAGTTGAAATGCTAATCT
GGCATAATTTCAGAACTAATTTTCTGTTAATGCCACTTGGAACATCTAAATTCCTCCTTTTTCAATAATACTATATTTGT
GTGTTGCAAACACACACAAATCATACTATATTTGTGTGTATATATAGTAAATTCCAACTTATAAAAGTAAAATTGGAATT
TTTTTCTTTCTTTGGAAAATTATTTTATAATTGAGAATTATAAATGTCACTTTTTTAAATGCTGCAACCTTTGAGATTGG
TTTCAATAAAGTAAAACTTAGTAAACATTAAGAAAAATGATAGCTTGATATGTTCACTAATATGGTAAATGAAACTTTT
ATGTGTGTATAGGCTCTTGTTAATTATAACTACCTTCACAGGAAAAACAGTCTTGTGGAAGGTAATGGTGCCAGTGAGAG
AAAATAGAGAAAGTAGAATAGTAGTAAGAGAGAAAATAGAATTTCCCGTTTAGAATAAAGCTAAGTAAAAAGTGAATTGT
TTCAGTTTTATTTATGTATTTGTAAGTGTGGTCTCATGCCATATATATTCAAAGATAGACAGAGATCTTAATCTTTAATT
TTTTATGGCCAAAGAAAATGAGTCCCATGTAAAAGGGACATTTTCGTAAGCTGATCTCAAGTGGTGTATTTTGATTTGCC
GCAGCAGCCTATGGAAGAAATATCATTTTGGCTGCCTAAGAAGAAAAATGTCATACCTTTCTATTTTTTATTCATACTCC
GCATATTTGGAATTCGTATTCTCACCATACTTTTGGGAAGATAACACGAAATTGCCTTACTTGCCTCGAAATCAAAATCT
GCCATTCGTTTTAAAATAAAATGGCTTTTTCATCTCATTTTCCTTTAGCAGAAAAAAGTAAGTTAAATTTTTTTGTAGCT
GTGTTAAGTGTACTGTTCCTCCCTCGTTGAGAACGAAACAAAGACATATGATTCCATTTACTGTAATTGTTTTGGTTTGC
CCAATTACAATAGCAGTAAAATCATTTACACATAGTAAAATGTTTGAGCCTTAAAGGGCAAGAAGACTAAATTTAGCTGA
AGATATACAACTTTCATGTGCCAATGAGGAAATGTATAAAATCAGTTATACATATTTTCCTCATTCATAGATATTGAAAA
TATTCATATATGTGTACATGTATTTGATAACTTAATATTTTCTAAATAACAGAAATTATATACTGTATATATATATTTGGAT
ATTATGATTATTGTTATTGCCACCATTGAGCACTTACTATGGGCTCAGAAAACTTCACTGTATTATTTTTAATTCTTATA
ACAACCCTGTGAAATATATATTATGCCTATTTCACAAATGAGAACTCTGAAATCAGGAAATTAAATCACTTGCTGAAGTT
TACAAAGCACTGTTAGGCAATAAAACAGAATTTCAAACTCACGTTTGCTTGTCTTCAAATTTTGTATGCTGTACAACCTG
ATTGTTTTACTACTTGTTCTTAATTTCAAAAAAAATCTTCATACTAAAAGATGATACTTTGGGAGTTCTAAAGAACATGT
TTTTGGCCGGGCGCGGTGGCTCACGCCTGTAATCCCAGCACTTTGGGAGGCCGAGGCAGAAGGATCACGAGGTCAGGAGA
TCGAGACCGTCCTGGCCAACAAGGTGAAACCCCGTCTCTACTAAAAAATACAAAAAATTAGCCGGGCGTGGTGGCGGGCG
CCTGTAGTCCCAGCTACTCGGGAAGCTGAGGCGGGAGAAAGGCGTGAACCTGGGAGGCAGAGCTTGTAGTGAGCCGAGAT
CGCGCCGCTGCACTCCAGCCTGGTTGACAGAGCGAGACTCCGTCTCAAAAATAACAAAACAAAACAAAACAAAACAAAAA
AAACCATGTTTCTTTTCAGAAGGTATACTCATTTGAAGTGGATACCAATTATTTGTATTAAAATTACTTATGGATAAATT
TGAATCTGCAAAAATTAAGTGCAACATTATTTTTTGGCACCTGTTATAGGAGGGTAACTCGAGGGTACCTCTTAATTAAC
TGGCCTCATGGGCCTTCCGCTCACTGCCCGCTTTCCAGTCGGGAAACCTGTCGTGCCAGCTGCATTAACATGGTCATAGC
TGTTTCCTTGCGTATTGGGCGCTCTCCGCTTCCTCGCTCACTGACTCGCTGCGCTCGGTCGTTCGGGTAAAGCCTGGGGT
GCCTAATGAGCAAAAGGCCAGCAAAAGGCCAGGAACCGTAAAAAGGCCGCGTTGCTGGCGTTTTTCCATAGGCTCCGCCC

```
CCCTGACGAGCATCACAAAAATCGACGCTCAAGTCAGAGGTGGCGAAACCCGACAGGACTATAAAGATACCAGGCGTTTC
CCCCTGGAAGCTCCCTCGTGCGCTCTCCTGTTCCGACCCTGCCGCTTACCGGATACCTGTCCGCCTTTCTCCCTTCGGGA
AGCGTGGCGCTTTCTCATAGCTCACGCTGTAGGTATCTCAGTTCGGTGTAGGTCGTTCGCTCCAAGCTGGGCTGTGTGCA
CGAACCCCCCGTTCAGCCCGACCGCTGCGCCTTATCCGGTAACTATCGTCTTGAGTCCAACCCGGTAAGACACGACTTAT
CGCCACTGGCAGCAGCCACTGGTAACAGGATTAGCAGAGCGAGGTATGTAGGCGGTGCTACAGAGTTCTTGAAGTGGTGG
CCTAACTACGGCTACACTAGAAGAACAGTATTTGGTATCTGCGCTCTGCTGAAGCCAGTTACCTTCGGAAAAAGAGTTGG
TAGCTCTTGATCCGGCAAACAAACCACCGCTGGTAGCGGTGGTTTTTTTGTTTGCAAGCAGCAGATTACGCGCAGAAAAA
AAGGATCTCAAGAAGATCCTTTGATCTTTTCTACGGGGTCTGACGCTCAGTGGAACGAAAACTCACGTTAAGGGATTTTG
GTCATGAGATTATCAAAAAGGATCTTCACCTAGATCCTTTTAAATTAAAAATGAAGTTTTAAATCAATCTAAAGTATATA
TGAGTAAACTTGGTCTGACAGTTACCAATGCTTAATCAGTGAGGCACCTATCTCAGCGATCTGTCTATTTCGTTCATCCA
TAGTTGCCTGACTCCCCGTCGTGTAGATAACTACGATACGGGAGGGCTTACCATCTGGCCCCAGTGCTGCAATGATACCG
CGAGAACCACGCTCACCGGCTCCAGATTTATCAGCAATAAACCAGCCAGCCGGAAGGGCCGAGCGCAGAAGTGGTCCTGC
AACTTTATCCGCCTCCATCCAGTCTATTAATTGTTGCCGGGAAGCTAGAGTAAGTAGTTCGCCAGTTAATAGTTTGCGCA
ACGTTGTTGCCATTGCTACAGGCATCGTGGTGTCACGCTCGTCGTTTGGTATGGCTTCATTCAGCTCCGGTTCCCAACGA
TCAAGGCGAGTTACATGATCCCCCATGTTGTGCAAAAAAGCGGTTAGCTCCTTCGGTCCTCCGATCGTTGTCAGAAGTAA
GTTGGCCGCAGTGTTATCACTCATGGTTATGGCAGCACTGCATAATTCTCTTACTGTCATGCCATCCGTAAGATGCTTTT
CTGTGACTGGTGAGTACTCAACCAAGTCATTCTGAGAATAGTGTATGCGGCGACCGAGTTGCTCTTGCCCGGCGTCAATA
CGGGATAATACCGCGCCACATAGCAGAACTTTAAAAGTGCTCATCATTGGAAAACGTTCTTCGGGGCGAAAACTCTCAAG
GATCTTACCGCTGTTGAGATCCAGTTCGATGTAACCCACTCGTGCACCCAACTGATCTTCAGCATCTTTTACTTTCACCA
GCGTTTCTGGGTGAGCAAAAACAGGAAGGCAAAATGCCGCAAAAAAGGGAATAAGGGCGACACGGAAATGTTGAATACTC
ATACTCTTCCTTTTTCAATATTATTGAAGCATTTATCAGGGTTATTGTCTCATGAGCGGATACATATTTGAATGTATTTA
GAAAAATAAACAAATAGGGGTTCCGCGCACATTTCCCCGAAAAGTGCCAC
```

Supplementary Figure 23. Map and nucleotide sequence of the unmodified donor construct targeting the human *DMD* locus. DNA sequences sharing identity to the human *DMD* gene are indicated in orange; hPGK1 promoter, human phosphoglycerate kinase 1 gene (*PGK1*) regulatory sequences; EGFP, enhanced green fluorescence protein gene (*EGFP*) open reading frame; bGH1 poly(A) signal, bovine growth hormone gene (*GH1*) polyadenylation signal; AmpR, *β-lactamase* ampicillin resistance gene; ori, high-copy number ColE1 prokaryotic origin of replication.

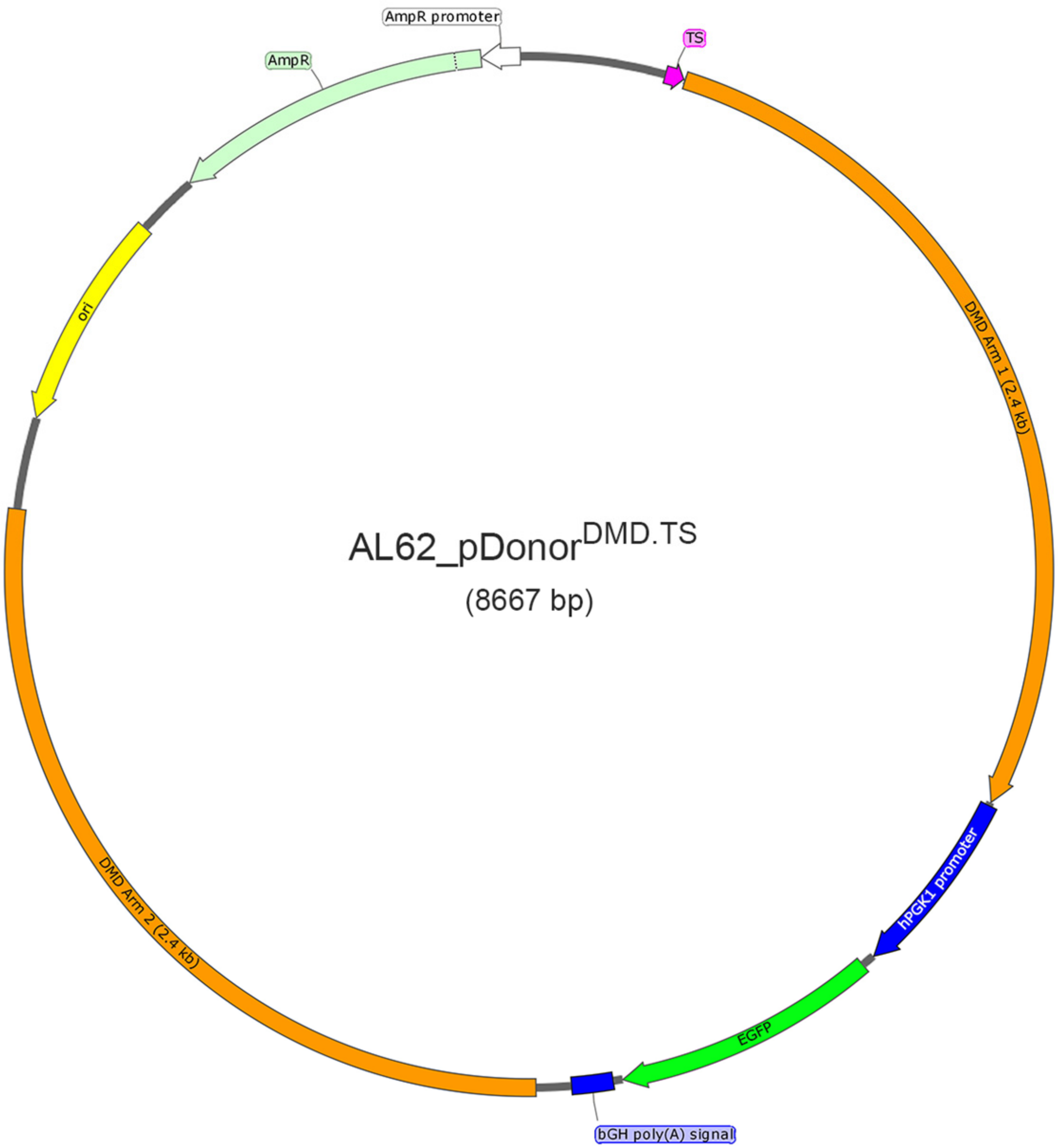

>AL62_pDonor.DMD.TS (8667 bp)
CTAAATTGTAAGCGTTAATATTTTGTTAAAATTCGCGTTAAATTTTTGTTAAATCAGCTCATTTTTTAACCAATAGGCCG
AAATCGGCAAAATCCCTTATAAATCAAAAGAATAGACCGAGATAGGGTTGAGTGGCCGCTACAGGGCGCTCCCATTCGCC
ATTCAGGCTGCGCAACTGTTGGGAAGGGCGTTTCGGTGCGGGCCTCTTCGCTATTACGCCAGCTGGCGAAAGGGGGATGT
GCTGCAAGGCGATTAAGTTGGGTAACGCCAGGGTTTTCCCAGTCACGACGTTGTAAAACGACGGCCAGTGAGCGCGACGT
AATACGACTCACTATAGGGCGAATTGGCGGAAGGCCGTCAAGGCCTAGGTGGAACTGCCCTGCTCCCACAGTTACATACA
GGCTAGGGAGTGGGTAGGAGCCTAGGCCATAAGGTAAAGAGGACACCAAATACAGTAAATAATAACATAATAATAAAGAA
ACCTTATGAATTCTATTCAGTGATATCCATATTGAGGTATTTAAGGTGAATGATACTGATGTCAGCAGTTTACCTGGATA
TGCATCAAAAATATGATTGATTGATGGATGGATCGGAGGAAGGAAAAGATATGTGATCAAAACGAGTATAGTAAAATGTT
CATGGTGGAATCTAGATATATAGATATCACTTGTAATTTTTAAAAATGTTATTGCATTTTTGAAATTTGTTATGTTAAAA
TATTGGGTGAAAATTCTGATGCCCTGGCCATGTCCCAAATCAGTTGAATCAGATCTCTGGTGTATTTTTTTTTTTAAGATC
CTTGGATGACTCCATTGTACAGCAAGTTTGCAAACCACTATTCTAACTTCTCACCACCCCCTTCTTTATAGCTTACAAAA
TTATATTTTTAAATGTCAAATGTCACAATTTCACTTTCCTTCTTCAAGAACTGATTAGACTTCTAGCTTCTCCTATGCTC
TCATCTTGCATAACGTTCATTTGCACGTGGTTAAAACTGATAGGCAATAAATAATAACCCATCTGTCTTTTCCTGACCCA
ACATAACTAAAACCTTCCTTTCAAGGCGAAAATGAACTATAATCTCACAGACAAGAGTAGCTATAGACAACTAATTCTTT
CTGGTCTCTCGTGGTCATGGGACTATTCACCACTGCCGTCCCTGCTGTCTATTTGGTGGACCAGCCAGTTTTACTGTGAC
CCATACCATGGCTCTTGAGAAGCATGAAGCTGCCGAAATCGCAGTTGTAGGGTTAATGGAAAACATGGTGAAGTATAGCT
TACATATATATACAATTTTGGCACAAAATGAAGCCTAAGAAATTGTACCGGGCTGGGTGTGGTGACTCATGCCTGTAATC
CCAGCACTTTGAGAGACCAAGGTGGGCGGATCACCTGAGGTCGGGAGTTCGAGACCAGACTGAGCAACATGGAGAAACCC

TCTCTACTTAAAATACAAAATTAGCCAGGCATGGTGGCGCATGCCTGTAACCCCAGCTACTCAGGAGGCTAAGGCAAGAG
AATCTCTTCCACCCTGGAGGCATAGGTTGTGGTGAGAGAAGATCACACCATTGCACTCCAGCTGGGGCAACAAGAGCGAA
GCTCCGTCTCAATAAATAAATAAATAAATAAATAAATAAATAAATAAATAAATAAATAAAAGAAAAAGAAATTGTACCAG
GAAGTGGATTGGGAATGCTCTATATATTCTCCCCCTCCAAGAAGTTTTTTTAATTTATTGTATTGTGAAAGATCTGCAGC
TATTTATTTTGTGTGGTACTCTTTTGCAATAAACTACTTCTGCATAAATGAACTCAAAAACTTTGATATTCATGAACCTT
CATAATGTTGATCTAGTACATCTTGCCCAACTAGCCCTTGGGCCATTCCTTCCATGCCACTGGTTGAAAAATATGCTCCC
CACCCCAGTAACATCAGCATCAGACCTCTGCAACCAGAAAGATTCCATAGGTAATGAAATCCAAATGCTGGCATTTGGAA
GTCAAACCAGTGTATTTTGAAATGGAAAGGCAAGGGGAAGTGCATGGAGCAAGTACAGCATGTGCTACAACTTCAGTTCA
TACCCTCCTTAAAATACTGTGCAACTAAACTGTCAATTGCCCATTGATAGAGATAGTATCTTTTTCAGCTTTACACCTTC
CGAATTCAACACACTGCTAGCCTCAGGACTAAGCGCTCAATAAATGTTTATCAAAAGATTGAGTAAATGAATCAGTGGTG
AAATAAAATGTACAAACACATACGCTTCATACACGTTTAGGAAGCATTTCTAGTGAGCTTTGTGGATATTTTATTTCTTA
AAGGTTCTTTTTCTTTCCATGTTCATGTTATTCTTTATTTTTTAAAATGTTGCTTCTGCTTATTTCTCTTTCTACGGCAG
CTATATTTACTTGCCTGAGAGCACAGCATATACTGATGTTAATCAAACTTTAGATGAAACACCTAAAATGATAGAAAAGA
ATCTGCTCTAATTAAATAATAAAGCAACCCCTACAGGTTTAGACATGTGCCTTCCGGTGTGGGAAAGAAAAAATTTAATG
AATGTAGTAGTTTTATGCCAAGAACATTTCCCTCGTGCACCTGTGTTTTAATAAGATAGAATATAAAATAGCAAAAGGGG
CCCGACTTTTGTGATGATATTTACTCATAAGGTAGTAAGTCAGATGTGATGACATTTTCTTTGACTTAGAGCTGCTATAC
TTGGGTCAGATTTCAGTTCAGTAAATTTGCAGTGAAGTTGTCTTTCTAACATGGTGTCATCCTGGAACTGCCCTGCTCCC
ACAGTAAGCTTCCACGGGGTTGGGGTTGCGCCTTTTCCAAGGCAGCCCTGGGTTTGCGCAGGGACGCGGCTGCTCTGGGC
GTGGTTCCGGGAAACGCAGCGGCGCCGACCCTGGGTCTCGCACATTCTTCACGTCCGTTCGCAGCGTCACCCGGATCTTC
GCCGCTACCCTTGTGGGCCCCCCGGCGACGCTTCCTGCTCCGCCCCTAAGTCGGGAAGGTTCCTTGCGGTTCGCGGCGTG
CCGGACGTGACAAACGGAAGCCGCACGTCTCACTAGTACCCTCGCAGACGGACAGCGCCAGGGAGCAATGGCAGCGCGCC
GACCGCGATGGGCTGTGGCCAATAGCGGCTGCTCAGCAGGGCGCGCCGAGAGCAGCGGCCGGGAAGGGGCGGTGCGGGAG
GCGGGGTGTGGGGCGGTAGTGTGGGCCCTGTTCCTGCCCGCGCGGTGTTCCGCATTCTGCAAGCCTCCGGAGCGCACGTC
GGCAGTCGGCTCCCTCGTTGACCGAATCACCGACCTCTCTCCCCACACGACTCTAGAGGATCCCCGGGTACCGGTCGCCA
CCATGGTGAGCAAGGGCGAGGAGCTGTTCACCGGGGTGGTGCCCATCCTGGTCGAGCTGGACGGCGACGTAAACGGCCAC
AAGTTCAGCGTGTCCGGCGAGGGCGAGGGCGATGCCACCTACGGCAAGCTGACCCTGAAGTTCATCTGCACCACCGGCAA
GCTGCCCGTGCCCTGGCCCACCCTCGTGACCACCCTGACCTACGGCGTGCAGTGCTTCAGCCGCTACCCCGACCACATGA
AGCAGCACGACTTCTTCAAGTCCGCCATGCCCGAAGGCTACGTCCAGGAGCGCACCATCTTCTTCAAGGACGACGGCAAC
TACAAGACCCGCGCCGAGGTGAAGTTCGAGGGCGACACCCTGGTGAACCGCATCGAGCTGAAGGGCATCGACTTCAAGGA
GGACGGCAACATCCTGGGGCACAAGCTGGAGTACAACTACAACAGCCACAACGTCTATATCATGGCCGACAAGCAGAAGA
ACGGCATCAAGGTGAACTTCAAGATCCGCCACAACATCGAGGACGGCAGCGTGCAGCTCGCCGACCACTACCAGCAGAAC
ACCCCCATCGGCGACGGCCCCGTGCTGCTGCCCGACAACCACTACCTGAGCACCCAGTCCGCCCTGAGCAAAGACCCCAA
CGAGAAGCGCGATCACATGGTCCTGCTGGAGTTCGTGACCGCCGCCGGGATCACTCTCGGCATGGACGAGCTGTACAAGT
AAAGCGGCCGCGACTCTAGAATTTTAAGCTGTGCCTTCTAGTTGCCAGCCATCTGTTGTTTGCCCCTCCCCCGTGCCTTC
CTTGACCCTGGAAGGTGCCACTCCCACTGTCCTTTCCTAATAAAATGAGGAAATTGCATCGCATTGTCTGAGTAGGTGTC
ATTCTATTCTGGGGGGTGGGGTGGGGCAGGACAGCAAGGGGGAGGATTGGGAAGACAATAGCAGGCATGCACGTGTACAG
GCTAGGGAGTGGGTAGGAGTGGGGGTGAAATCCTCTTAATGTTTATGGTGTCAGTAGATTCAAACTAAAATTAGCCTTAC
AGCCATACTCCTAATAAGGGGCCCCTGGCATATTTAATTGATTTAACAAATTTATCAAAAATAGATAAACTGAAATCTGC
CTTGAAATTAATTACTGTATCTCTATTTTTATAAGAAAATATTTTGGACCCGTTCCCTCTGCCTTATGGGTGCAACTCCC
GGCAGAATAGGTCATGTCTCCTGAAAATAGTTCTTATTTTCTTTACTACTTATGACCTCTTATAGCCTAGAAGTTTTTCC
TTGCTATTTCTAGTTGAAATGCTAATCTGGCATAATTTCTAGTTGAAATGCTAATCTGGCATAATTTCAGAACTAATTTT
CTGTTAATGCCACTTGGAACATCTAAATTCCTCCTTTTTCAATAATACTATATTTGTGTGTTGCAAACACACACAAATCA
TACTATATTTGTGTGTATATATAGTAAATTCCAACTTATAAAAGTAAAATTGGAATTTTTTTCTTTCTTTGGAAAATTAT
TTTATAATTGAGAATTATAAATGTCACTTTTTTAAATGCTGCAACCTTTGAGATTGGTTTCAATAAAGTAAAACTTAGTA
AACATTAAGAAAAATGATAGCTTGATATGTTCACTAATATGGTAAATGAAAACTTTTATGTGTGTATAGGCTCTTGTTAA
TTATAACTACCTTCACAGGAAAAACAGTCTTGTGGAAGGTAATGGTGCCAGTGAGAGAAAATAGAGAAAGTAGAATAGTA
GTAAGAGAGAAAATAGAATTTCCCGTTTAGAATAAAGCTAAGTAAAAAGTGAATTGTTTCAGTTTTATTTATGTATTTGT
AAGTGTGGTCTCATGCCATATATATTCAAAGATAGACAGAGATCTTAATCTTTAATTTTTTATGGCCAAAGAAAATGAGT
CCCATGTAAAAGGGACATTTTCGTAAGCTGATCTCAAGTGGTGTATTTTGATTTGCCGCAGCAGCCTATGGAAGAAATAT
CATTTTGGCTGCCTAAGAAGAAAAATGTCATACCTTTCTATTTTTTATTCATACTCCGCATATTTGGAATTCGTATTCTC
ACCATACTTTTGGGAAGATAACACGAAATTGCCTTACTTGCCTCGAAATCAAAATCTGCCATTCGTTTTAAAATAAAATG
GCTTTTTCATCTCATTTTCCTTTAGCAGAAAAAAGTAAGTTAAATTTTTTTGTAGCTGTGTTAAGTGTACTGTTCCTCCC
TCGTTGAGAACGAAACAAAGACATATGATTCCATTTACTGTAATTGTTTTGGTTTGCCCAATTACAATAGCAGTAAAATC
ATTTACACATAGTAAAATGTTTGAGCCTTAAAGGGCAAGAAGACTAAATTTAGCTGAAGATATACAACTTTCATGTGCCA
ATGAGGAAATGTATAAAATCAGTTATACATATTTTCCTCATTCATAGATATTGAAAATATTCATATATGTGTACATGTAT
TTGATAACTTAATATTTTCTAAATAACAGAAATTATATACTGTATATATATTTGGATATTATGATTATTGTTATTGCCAC
CATTGAGCACTTACTATGGGCTCAGAAAACTTCACTGTATTATTTTTAATTCTTATAACAACCCTGTGAAATATATATTA
TGCCTATTTCACAAATGAGAACTCTGAAATCAGGAAATTAAATCACTTGCTGAAGTTTACAAAGCACTGTTAGGCAATAA
AACAGAATTTCAAACTCACGTTTGCTTGTCTTCAAATTTTGTATGCTGTACAACCTGATTGTTTTACTACTTGTTCTTAA
TTTCAAAAAAAATCTTCATACTAAAAGATGATACTTTGGGAGTTCTAAAGAACATGTTTTTGGCCGGGCGCGGTGGCTCA
CGCCTGTAATCCCAGCACTTTGGGAGGCCGAGGCAGAAGGATCACGAGGTCAGGAGATCGAGACCGTCCTGGCCAACAAG
GTGAAACCCCGTCTCTACTAAAAAATACAAAAAATTAGCCGGGCGTGGTGGCGGGCGCCTGTAGTCCCAGCTACTCGGGA
AGCTGAGGCGGGAGAAAGGCGTGAACCTGGGAGGCAGAGCTTGTAGTGAGCCGAGATCGCGCCGCTGCACTCCAGCCTGG
TTGACAGAGCGAGACTCCGTCTCAAAAATAACAAAACAAAACAAAACAAAACAAAAAAAACCATGTTTCTTTTCAGAAGG
TATACTCATTTGAAGTGGATACCAATTATTTGTATTAAAATTACTTATGGATAAATTTGAATCTGCAAAAATTAAGTGCA
ACATTATTTTTTGGCACCTGTTATAGGAGGGTAACTCGAGGGTACCTCTTAATTAACTGGCCTCATGGGCCTTCCGCTCA
CTGCCCGCTTTCCAGTCGGGAAACCTGTCGTGCCAGCTGCATTAACATGGTCATAGCTGTTTCCTTGCGTATTGGGCGCT
CTCCGCTTCCTCGCTCACTGACTCGCTGCGCTCGGTCGTTCGGGTAAAGCCTGGGGTGCCTAATGAGCAAAAGGCCAGCA

```
AAAGGCCAGGAACCGTAAAAAGGCCGCGTTGCTGGCGTTTTTCCATAGGCTCCGCCCCCCTGACGAGCATCACAAAAATC
GACGCTCAAGTCAGAGGTGGCGAAACCCGACAGGACTATAAAGATACCAGGCGTTTCCCCCTGGAAGCTCCCTCGTGCGC
TCTCCTGTTCCGACCCTGCCGCTTACCGGATACCTGTCCGCCTTTCTCCCTTCGGGAAGCGTGGCGCTTTCTCATAGCTC
ACGCTGTAGGTATCTCAGTTCGGTGTAGGTCGTTCGCTCCAAGCTGGGCTGTGTGCACGAACCCCCCGTTCAGCCCGACC
GCTGCGCCTTATCCGGTAACTATCGTCTTGAGTCCAACCCGGTAAGACACGACTTATCGCCACTGGCAGCAGCCACTGGT
AACAGGATTAGCAGAGCGAGGTATGTAGGCGGTGCTACAGAGTTCTTGAAGTGGTGGCCTAACTACGGCTACACTAGAAG
AACAGTATTTGGTATCTGCGCTCTGCTGAAGCCAGTTACCTTCGGAAAAAGAGTTGGTAGCTCTTGATCCGGCAAACAAA
CCACCGCTGGTAGCGGTGGTTTTTTTGTTTGCAAGCAGCAGATTACGCGCAGAAAAAAAGGATCTCAAGAAGATCCTTTG
ATCTTTTCTACGGGGTCTGACGCTCAGTGGAACGAAAACTCACGTTAAGGGATTTTGGTCATGAGATTATCAAAAAGGAT
CTTCACCTAGATCCTTTTAAATTAAAAATGAAGTTTTAAATCAATCTAAAGTATATATGAGTAAACTTGGTCTGACAGTT
ACCAATGCTTAATCAGTGAGGCACCTATCTCAGCGATCTGTCTATTTCGTTCATCCATAGTTGCCTGACTCCCCGTCGTG
TAGATAACTACGATACGGGAGGGCTTACCATCTGGCCCCAGTGCTGCAATGATACCGCGAGAACCACGCTCACCGGCTCC
AGATTTATCAGCAATAAACCAGCCAGCCGGAAGGGCCGAGCGCAGAAGTGGTCCTGCAACTTTATCCGCCTCCATCCAGT
CTATTAATTGTTGCCGGGAAGCTAGAGTAAGTAGTTCGCCAGTTAATAGTTTGCGCAACGTTGTTGCCATTGCTACAGGC
ATCGTGGTGTCACGCTCGTCGTTTGGTATGGCTTCATTCAGCTCCGGTTCCCAACGATCAAGGCGAGTTACATGATCCCC
CATGTTGTGCAAAAAAGCGGTTAGCTCCTTCGGTCCTCCGATCGTTGTCAGAAGTAAGTTGGCCGCAGTGTTATCACTCA
TGGTTATGGCAGCACTGCATAATTCTCTTACTGTCATGCCATCCGTAAGATGCTTTTCTGTGACTGGTGAGTACTCAACC
AAGTCATTCTGAGAATAGTGTATGCGGCGACCGAGTTGCTCTTGCCCGGCGTCAATACGGGATAATACCGCGCCACATAG
CAGAACTTTAAAAGTGCTCATCATTGGAAAACGTTCTTCGGGGCGAAAACTCTCAAGGATCTTACCGCTGTTGAGATCCA
GTTCGATGTAACCCACTCGTGCACCCAACTGATCTTCAGCATCTTTTACTTTCACCAGCGTTTCTGGGTGAGCAAAAACA
GGAAGGCAAAATGCCGCAAAAAAGGGAATAAGGGCGACACGGAAATGTTGAATACTCATACTCTTCCTTTTTCAATATTA
TTGAAGCATTTATCAGGGTTATTGTCTCATGAGCGGATACATATTTGAATGTATTTAGAAAAATAAACAAATAGGGGTTC
CGCGCACATTTCCCCGAAAAGTGCCAC
```

Supplementary Figure 24. Map and nucleotide sequence of the unmodified donor construct targeting the human *DMD* locus. DNA sequences sharing identity to the human *DMD* gene are indicated in orange; Magenta arrow, gRNA^{DMD} target site (TS); hPGK1 promoter, human phosphoglycerate kinase 1 gene (*PGK1*) regulatory sequences; EGFP, enhanced green fluorescence protein gene (*EGFP*) open reading frame; bGH1 poly(A) signal, bovine growth hormone gene (*GH1*) polyadenylation signal; AmpR, *β-lactamase* ampicillin resistance gene; ori, high-copy number ColE1 prokaryotic origin of replication.

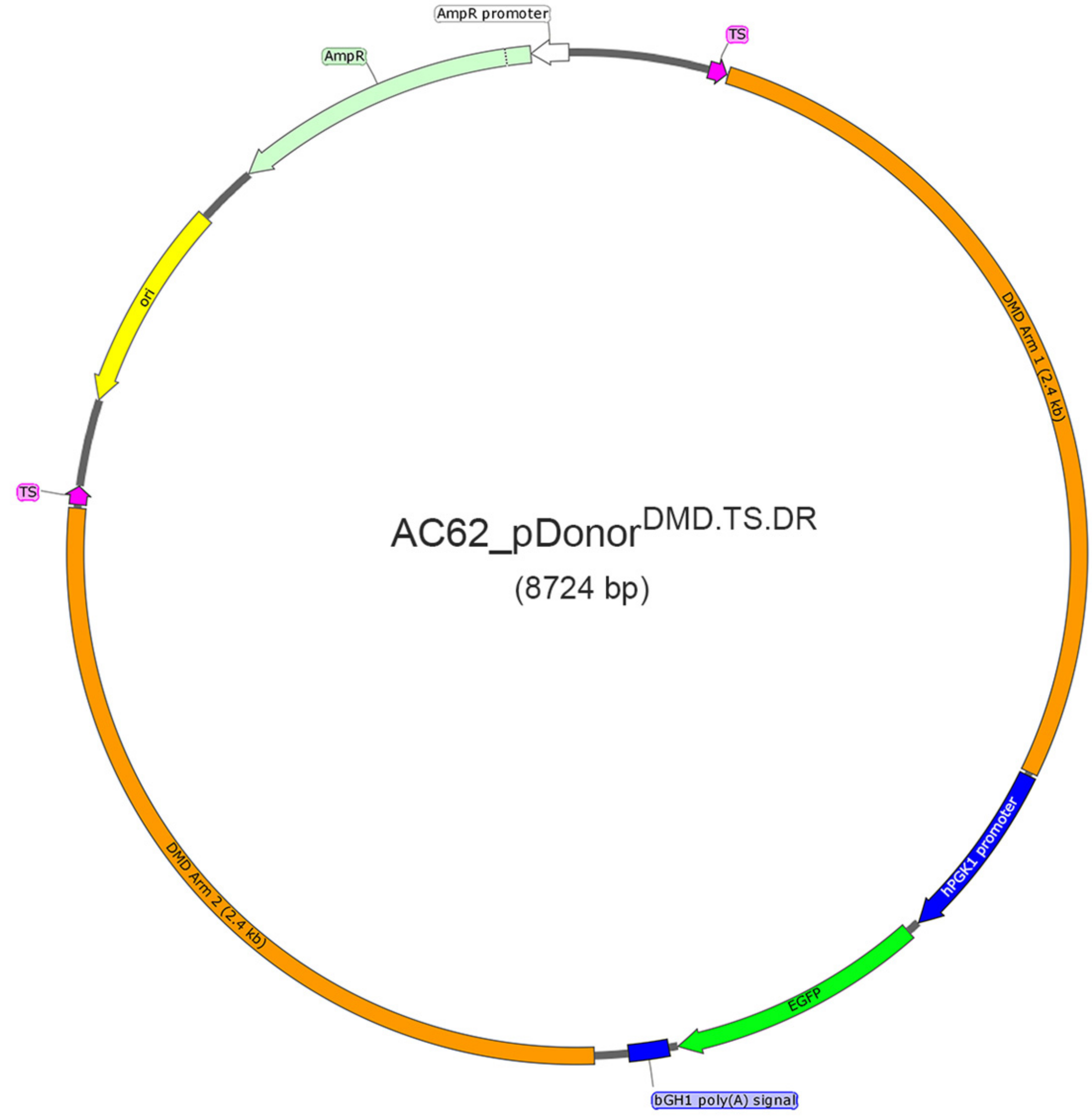


```
>AC62_pdonor.DMD.TS.DR  (8724 bp)
CTAAATTGTAAGCGTTAATATTTTGTTAAAATTCGCGTTAAATTTTTGTTAAATCAGCTCATTTTTTAACCAATAGGCCG
AAATCGGCAAAATCCCTTATAAATCAAAAGAATAGACCGAGATAGGGTTGAGTGGCCGCTACAGGGCGCTCCCATTCGCC
ATTCAGGCTGCGCAACTGTTGGGAAGGGCGTTTCGGTGCGGGCCTCTTCGCTATTACGCCAGCTGGCGAAAGGGGGATGT
GCTGCAAGGCGATTAAGTTGGGTAACGCCAGGGTTTTCCCAGTCACGACGTTGTAAAACGACGGCCAGTGAGCGCGACGT
AATACGACTCACTATAGGGCGAATTGGCGGAAGGCCGTCAAGGCCTAGGTGGAACTGCCCTGCTCCCACAGTTACATACA
GGCTAGGGAGTGGGTAGGAGCCTAGGCCATAAGGTAAAGAGGACACCAAATACAGTAAATAATAACATAATAATAAAGAA
ACCTTATGAATTCTATTCAGTGATATCCATATTGAGGTATTTAAGGTGAATGATACTGATGTCAGCAGTTTACCTGGATA
TGCATCAAAAATATGATTGATTGATGGATGGATCGGAGGAAGGAAAAGATATGTGATCAAAACGAGTATAGTAAAATGTT
CATGGTGGAATCTAGATATATAGATATCACTTGTAATTTTTAAAAATGTTATTGCATTTTTGAAATTTGTTATGTTAAAA
TATTGGGTGAAAATTCTGATGCCCTGGCCATGTCCCAAATCAGTTGAATCAGATCTCTGGTGTATTTTTTTTTTAAGATC
CTTGGATGACTCCATTGTACAGCAAGTTTGCAAACCACTATTCTAACTTCTCACCACCCCCTTCTTTATAGCTTACAAAA
TTATATTTTTAAATGTCAAATGTCACAATTTCACTTTCCTTCTTCAAGAACTGATTAGACTTCTAGCTTCTCCTATGCTC
TCATCTTGCATAACGTTCATTTGCACGTGGTTAAAACTGATAGGCAATAAATAATAACCCATCTGTCTTTTCCTGACCCA
ACATAACTAAAACCTTCCTTTCAAGGCGAAAATGAACTATAATCTCACAGACAAGAGTAGCTATAGACAACTAATTCTTT
CTGGTCTCTCGTGGTCATGGGACTATTCACCACTGCCGTCCCTGCTGTCTATTTGGTGGACCAGCCAGTTTTACTGTGAC
CCATACCATGGCTCTTGAGAAGCATGAAGCTGCCGAAATCGCAGTTGTAGGGTTAATGGAAAACATGGTGAAGTATAGCT
TACATATATATACAATTTTGGCACAAAATGAAGCCTAAGAAATTGTACCGGGCTGGGTGTGGTGACTCATGCCTGTAATC
```

CCAGCACTTTGAGAGACCAAGGTGGGCGGATCACCTGAGGTCGGGAGTTCGAGACCAGACTGAGCAACATGGAGAAACCC
TCTCTACTTAAAATACAAAATTAGCCAGGCATGGTGGCGCATGCCTGTAACCCCAGCTACTCAGGAGGCTAAGGCAAGAG
AATCTCTTCCACCCTGGAGGCATAGGTTGTGGTGAGAGAAGATCACACCATTGCACTCCAGCTGGGGCAACAAGAGCGAA
GCTCCGTCTCAATAAATAAATAAATAAATAAATAAATAAATAAATAAATAAATAAATAAAAGAAAAAGAAATTGTACCAG
GAAGTGGATTGGGAATGCTCTATATATTCTCCCCCTCCAAGAAGTTTTTTAATTTATTGTATTGTGAAAGATCTGCAGC
TATTTATTTTGTGTGGTACTCTTTTGCAATAAACTACTTCTGCATAAATGAACTCAAAAACTTTGATATTCATGAACCTT
CATAATGTTGATCTAGTACATCTTGCCCAACTAGCCCTTGGGCCATTCCTTCCATGCCACTGGTTGAAAAATATGCTCCC
CACCCCAGTAACATCAGCATCAGACCTCTGCAACCAGAAAGATTCCATAGGTAATGAAATCCAAATGCTGGCATTTGGAA
GTCAAACCAGTGTATTTTGAAATGGAAAGGCAAGGGGAAGTGCATGGAGCAAGTACAGCATGTGCTACAACTTCAGTTCA
TACCCTCCTTAAAATACTGTGCAACTAAACTGTCAATTGCCCATTGATAGAGATAGTATCTTTTTCAGCTTTACACCTTC
CGAATTCAACACACTGCTAGCCTCAGGACTAAGCGCTCAATAAATGTTTATCAAAAGATTGAGTAAATGAATCAGTGGTG
AAATAAAATGTACAAACACATACGCTTCATACACGTTTAGGAAGCATTTCTAGTGAGCTTTGTGGATATTTTATTTCTTA
AAGGTTCTTTTTCTTTCCATGTTCATGTTATTCTTTATTTTTTAAAATGTTGCTTCTGCTTATTTCTCTTTCTACGGCAG
CTATATTTACTTGCCTGAGAGCACAGCATATACTGATGTTAATCAAACTTTAGATGAAACACCTAAAATGATAGAAAAGA
ATCTGCTCTAATTAAATAATAAAGCAACCCCTACAGGTTTAGACATGTGCCTTCCGGTGTGGGAAAGAAAAAATTTAATG
AATGTAGTAGTTTTATGCCAAGAACATTTCCCTCGTGCACCTGTGTTTTAATAAGATAGAATATAAAATAGCAAAAGGGG
CCCGACTTTTGTGATGATATTTACTCATAAGGTAGTAAGTCAGATGTGATGACATTTTCTTTGACTTAGAGCTGCTATAC
TTGGGTCAGATTTCAGTTCAGTAAATTTGCAGTGAAGTTGTCTTTCTAACATGGTGTCATCCTGGAACTGCCCTGCTCCC
ACAGTAAGCTTCCACGGGGTTGGGGTTGCGCCTTTTCCAAGGCAGCCCTGGGTTTGCGCAGGGACGCGGCTGCTCTGGGC
GTGGTTCCGGGAAACGCAGCGGCGCCGACCCTGGGTCTCGCACATTCTTCACGTCCGTTCGCAGCGTCACCCGGATCTTC
GCCGCTACCCTTGTGGGCCCCCCGGCGACGCTTCCTGCTCCGCCCCTAAGTCGGGAAGGTTCCTTGCGGTTCGCGGCGTG
CCGGACGTGACAAACGGAAGCCGCACGTCTCACTAGTACCCTCGCAGACGGACAGCGCCAGGGAGCAATGGCAGCGCGCC
GACCGCGATGGGCTGTGGCCAATAGCGGCTGCTCAGCAGGGCGCGCCGAGAGCAGCGGCCGGGAAGGGGCGGTGCGGGAG
GCGGGGTGTGGGGCGGTAGTGTGGGCCCTGTTCCTGCCCGCGCGGTGTTCCGCATTCTGCAAGCCTCCGGAGCGCACGTC
GGCAGTCGGCTCCCTCGTTGACCGAATCACCGACCTCTCTCCCCACACGACTCTAGAGGATCCCCGGGTACCGGTCGCCA
CCATGGTGAGCAAGGGCGAGGAGCTGTTCACCGGGGTGGTGCCCATCCTGGTCGAGCTGGACGGCGACGTAAACGGCCAC
AAGTTCAGCGTGTCCGGCGAGGGCGAGGGCGATGCCACCTACGGCAAGCTGACCCTGAAGTTCATCTGCACCACCGGCAA
GCTGCCCGTGCCCTGGCCCACCCTCGTGACCACCCTGACCTACGGCGTGCAGTGCTTCAGCCGCTACCCCGACCACATGA
AGCAGCACGACTTCTTCAAGTCCGCCATGCCCGAAGGCTACGTCCAGGAGCGCACCATCTTCTTCAAGGACGACGGCAAC
TACAAGACCCGCGCCGAGGTGAAGTTCGAGGGCGACACCCTGGTGAACCGCATCGAGCTGAAGGGCATCGACTTCAAGGA
GGACGGCAACATCCTGGGGCACAAGCTGGAGTACAACTACAACAGCCACAACGTCTATATCATGGCCGACAAGCAGAAGA
ACGGCATCAAGGTGAACTTCAAGATCCGCCACAACATCGAGGACGGCAGCGTGCAGCTCGCCGACCACTACCAGCAGAAC
ACCCCCATCGGCGACGGCCCCGTGCTGCTGCCCGACAACCACTACCTGAGCACCCAGTCCGCCCTGAGCAAAGACCCCAA
CGAGAAGCGCGATCACATGGTCCTGCTGGAGTTCGTGACCGCCGCCGGGATCACTCTCGGCATGGACGAGCTGTACAAGT
AAAGCGGCCGCGACTCTAGAATTTTAAGCTGTGCCTTCTAGTTGCCAGCCATCTGTTGTTTGCCCCTCCCCCGTGCCTTC
CTTGACCCTGGAAGGTGCCACTCCCACTGTCCTTTCCTAATAAAATGAGGAAATTGCATCGCATTGTCTGAGTAGGTGTC
ATTCTATTCTGGGGGGTGGGGTGGGGCAGGACAGCAAGGGGGAGGATTGGGAAGACAATAGCAGGCATGCACGTGTACAG
GCTAGGGAGTGGGTAGGAGTGGGGGTGAAATCCTCTTAATGTTTATGGTGTCAGTAGATTCAAACTAAAATTAGCCTTAC
AGCCATACTCCTAATAAGGGGCCCCTGGCATATTTAATTGATTTAACAAATTTATCAAAAATAGATAAACTGAAATCTGC
CTTGAAATTAATTACTGTATCTCTATTTTTATAAGAAAATATTTTGGACCCGTTCCCTCTGCCTTATGGGTGCAACTCCC
GGCAGAATAGGTCATGTCTCCTGAAAATAGTTCTTATTTTCTTTACTACTTATGACCTCTTATAGCCTAGAAGTTTTTCC
TTGCTATTTCTAGTTGAAATGCTAATCTGGCATAATTTCTAGTTGAAATGCTAATCTGGCATAATTTCAGAACTAATTTT
CTGTTAATGCCACTTGGAACATCTAAATTCCTCCTTTTTCAATAATACTATATTTGTGTGTTGCAAACACACACAAATCA
TACTATATTTGTGTGTATATATAGTAAATTCCAACTTATAAAAGTAAAATTGGAATTTTTTTCTTTCTTTGGAAAATTAT
TTTATAATTGAGAATTATAAATGTCACTTTTTTAAATGCTGCAACCTTTGAGATTGGTTTCAATAAAGTAAAACTTAGTA
AACATTAAGAAAAATGATAGCTTGATATGTTCACTAATATGGTAAATGAAAACTTTTATGTGTGTATAGGCTCTTGTTAA
TTATAACTACCTTCACAGGAAAAACAGTCTTGTGGAAGGTAATGGTGCCAGTGAGAGAAAATAGAGAAAGTAGAATAGTA
GTAAGAGAGAAAATAGAATTTCCCGTTTAGAATAAAGCTAAGTAAAAAGTGAATTGTTTCAGTTTTATTTATGTATTTGT
AAGTGTGGTCTCATGCCATATATATTCAAAGATAGACAGAGATCTTAATCTTTAATTTTTTATGGCCAAAGAAAATGAGT
CCCATGTAAAAGGGACATTTTCGTAAGCTGATCTCAAGTGGTGTATTTTGATTTGCCGCAGCAGCCTATGGAAGAAATAT
CATTTTGGCTGCCTAAGAAGAAAAATGTCATACCTTTCTATTTTTTATTCATACTCCGCATATTTGGAATTCGTATTCTC
ACCATACTTTTGGGAAGATAACACGAAATTGCCTTACTTGCCTCGAAATCAAAATCTGCCATTCGTTTTAAAATAAAATG
GCTTTTTCATCTCATTTTCCTTTAGCAGAAAAAAGTAAGTTAAATTTTTTTGTAGCTGTGTTAAGTGTACTGTTCCTCCC
TCGTTGAGAACGAAACAAAGACATATGATTCCATTTACTGTAATTGTTTTGGTTTGCCCAATTACAATAGCAGTAAAATC
ATTTACACATAGTAAAATGTTTGAGCCTTAAAGGGCAAGAAGACTAAATTTAGCTGAAGATATACAACTTTCATGTGCCA
ATGAGGAAATGTATAAAATCAGTTATACATATTTTCCTCATTCATAGATATTGAAAATATTCATATATGTGTACATGTAT
TTGATAACTTAATATTTTCTAAATAACAGAAATTATATACTGTATATATATTTGGATATTATGATTATTGTTATTGCCAC
CATTGAGCACTTACTATGGGCTCAGAAAACTTCACTGTATTATTTTTAATTCTTATAACAACCCTGTGAAATATATATTA
TGCCTATTTCACAAATGAGAACTCTGAAATCAGGAAATTAAATCACTTGCTGAAGTTTACAAAGCACTGTTAGGCAATAA
AACAGAATTTCAAACTCACGTTTGCTTGTCTTCAAATTTTGTATGCTGTACAACCTGATTGTTTTACTACTTGTTCTTAA
TTTCAAAAAAAATCTTCATACTAAAAGATGATACTTTGGGAGTTCTAAAGAACATGTTTTTGGCCGGGCGCGGTGGCTCA
CGCCTGTAATCCCAGCACTTTGGGAGGCCGAGGCAGAAGGATCACGAGGTCAGGAGATCGAGACCGTCCTGGCCAACAAG
GTGAAACCCCGTCTCTACTAAAAAATACAAAAAATTAGCCGGGCGTGGTGGCGGGCGCCTGTAGTCCCAGCTACTCGGGA
AGCTGAGGCGGGAGAAAGGCGTGAACCTGGGAGGCAGAGCTTGTAGTGAGCCGAGATCGCGCCGCTGCACTCCAGCCTGG
TTGACAGAGCGAGACTCCGTCTCAAAAATAACAAAACAAAACAAAACAAAACAAAAAAAACCATGTTTCTTTTCAGAAGG
TATACTCATTTGAAGTGGATACCAATTATTTGTATTAAAATTACTTATGGATAAATTTGAATCTGCAAAAATTAAGTGCA
ACATTATTTTTTGGCACCTGTTATAGGAGGGTAACTCGAGTGGAACTGCCCTGCTCCCACAGTTACATACAGGCTAGGGA
GTGGGTAGGAGCTCGAGGGTACCTCTTAATTAACTGGCCTCATGGGCCTTCCGCTCACTGCCCGCTTTCCAGTCGGGAAA

CCTGTCGTGCCAGCTGCATTAACATGGTCATAGCTGTTTCCTTGCGTATTGGGCGCTCTCCGCTTCCTCGCTCACTGACT
CGCTGCGCTCGGTCGTTCGGGTAAAGCCTGGGGTGCCTAATGAGCAAAAGGCCAGCAAAAGGCCAGGAACCGTAAAAAGG
CCGCGTTGCTGGCGTTTTTCCATAGGCTCCGCCCCCCTGACGAGCATCACAAAAATCGACGCTCAAGTCAGAGGTGGCGA
AACCCGACAGGACTATAAAGATACCAGGCGTTTCCCCCTGGAAGCTCCCTCGTGCGCTCTCCTGTTCCGACCCTGCCGCT
TACCGGATACCTGTCCGCCTTTCTCCCTTCGGGAAGCGTGGCGCTTTCTCATAGCTCACGCTGTAGGTATCTCAGTTCGG
TGTAGGTCGTTCGCTCCAAGCTGGGCTGTGTGCACGAACCCCCCGTTCAGCCCGACCGCTGCGCCTTATCCGGTAACTAT
CGTCTTGAGTCCAACCCGGTAAGACACGACTTATCGCCACTGGCAGCAGCCACTGGTAACAGGATTAGCAGAGCGAGGTA
TGTAGGCGGTGCTACAGAGTTCTTGAAGTGGTGGCCTAACTACGGCTACACTAGAAGAACAGTATTTGGTATCTGCGCTC
TGCTGAAGCCAGTTACCTTCGGAAAAAGAGTTGGTAGCTCTTGATCCGGCAAACAAACCACCGCTGGTAGCGGTGGTTTT
TTTGTTTGCAAGCAGCAGATTACGCGCAGAAAAAAAGGATCTCAAGAAGATCCTTTGATCTTTTCTACGGGGTCTGACGC
TCAGTGGAACGAAAACTCACGTTAAGGGATTTTGGTCATGAGATTATCAAAAAGGATCTTCACCTAGATCCTTTTAAATT
AAAAATGAAGTTTTAAATCAATCTAAAGTATATATGAGTAAACTTGGTCTGACAGTTACCAATGCTTAATCAGTGAGGCA
CCTATCTCAGCGATCTGTCTATTTCGTTCATCCATAGTTGCCTGACTCCCCGTCGTGTAGATAACTACGATACGGGAGGG
CTTACCATCTGGCCCCAGTGCTGCAATGATACCGCGAGAACCACGCTCACCGGCTCCAGATTTATCAGCAATAAACCAGC
CAGCCGGAAGGGCCGAGCGCAGAAGTGGTCCTGCAACTTTATCCGCCTCCATCCAGTCTATTAATTGTTGCCGGGAAGCT
AGAGTAAGTAGTTCGCCAGTTAATAGTTTGCGCAACGTTGTTGCCATTGCTACAGGCATCGTGGTGTCACGCTCGTCGTT
TGGTATGGCTTCATTCAGCTCCGGTTCCCAACGATCAAGGCGAGTTACATGATCCCCCATGTTGTGCAAAAAAGCGGTTA
GCTCCTTCGGTCCTCCGATCGTTGTCAGAAGTAAGTTGGCCGCAGTGTTATCACTCATGGTTATGGCAGCACTGCATAAT
TCTCTTACTGTCATGCCATCCGTAAGATGCTTTTCTGTGACTGGTGAGTACTCAACCAAGTCATTCTGAGAATAGTGTAT
GCGGCGACCGAGTTGCTCTTGCCCGGCGTCAATACGGGATAATACCGCGCCACATAGCAGAACTTTAAAAGTGCTCATCA
TTGGAAAACGTTCTTCGGGGCGAAAACTCTCAAGGATCTTACCGCTGTTGAGATCCAGTTCGATGTAACCCACTCGTGCA
CCCAACTGATCTTCAGCATCTTTTACTTTCACCAGCGTTTCTGGGTGAGCAAAAACAGGAAGGCAAAATGCCGCAAAAAA
GGGAATAAGGGCGACACGGAAATGTTGAATACTCATACTCTTCCTTTTTCAATATTATTGAAGCATTTATCAGGGTTATT
GTCTCATGAGCGGATACATATTTGAATGTATTTAGAAAAATAAACAAATAGGGGTTCCGCGCACATTTCCCCGAAAAGTG
CCAC

Supplementary Figure 25. Map and nucleotide sequence of the unmodified donor construct targeting the human *DMD* locus. DNA sequences sharing identity to the human *DMD* gene are indicated in orange; Magenta arrows, gRNA^{DMD} target site (TS); hPGK1 promoter, human phosphoglycerate kinase 1 gene (*PGK1*) regulatory sequences; EGFP, enhanced green fluorescence protein gene (*EGFP*) open reading frame; bGH1 poly(A) signal, bovine growth hormone gene (*GH1*) polyadenylation signal; AmpR, *β-lactamase* ampicillin resistance gene; ori, high-copy number ColE1 prokaryotic origin of replication.

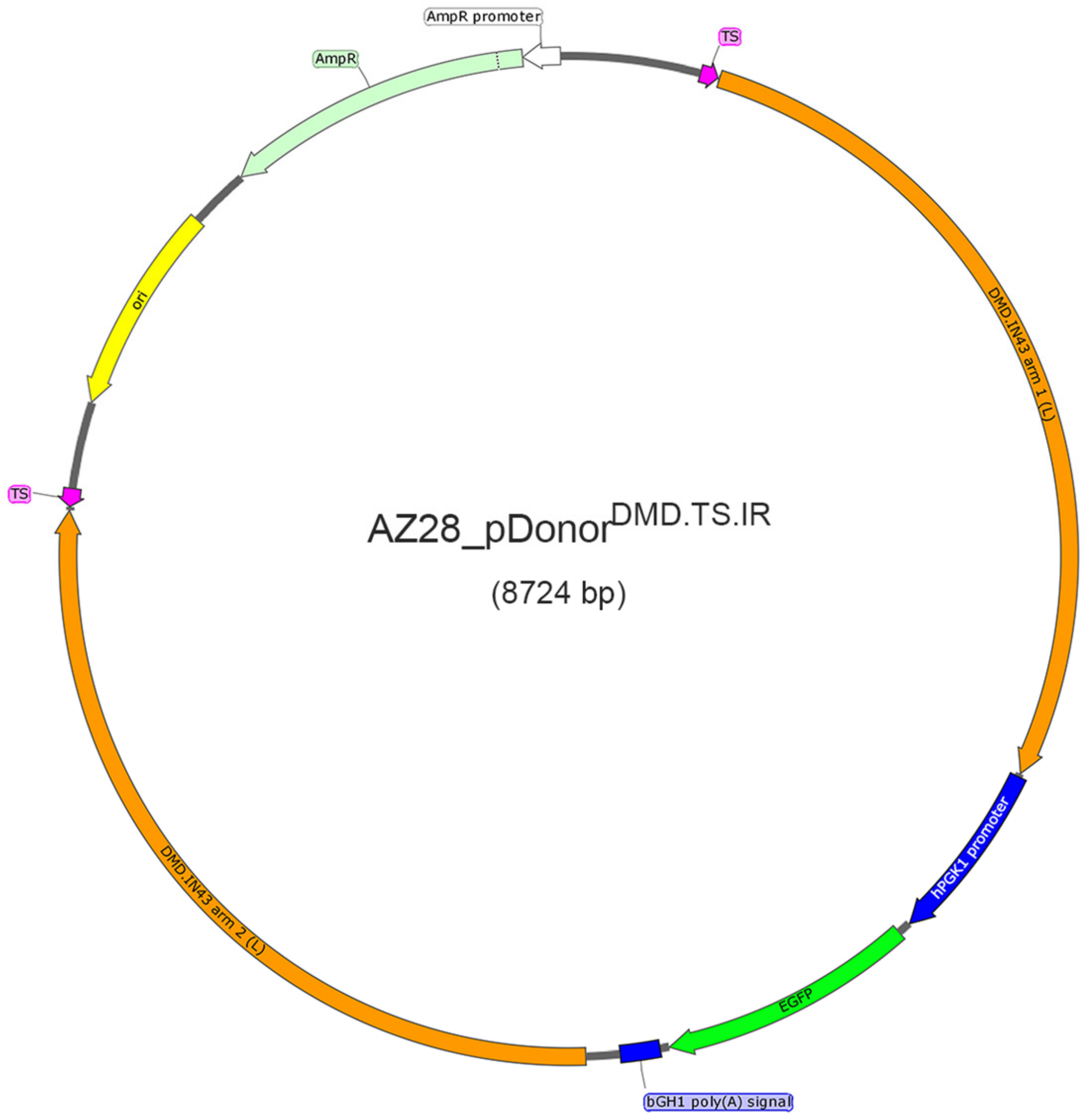


```
>AZ28_pdonor.DMD.TS.IR  (8724 bp)
CTAAATTGTAAGCGTTAATATTTTGTTAAAATTCGCGTTAAATTTTTGTTAAATCAGCTCATTTTTTAACCAATAGGCCG
AAATCGGCAAAATCCCTTATAAATCAAAAGAATAGACCGAGATAGGGTTGAGTGGCCGCTACAGGGCGCTCCCATTCGCC
ATTCAGGCTGCGCAACTGTTGGGAAGGGCGTTTCGGTGCGGGCCTCTTCGCTATTACGCCAGCTGGCGAAAGGGGGATGT
GCTGCAAGGCGATTAAGTTGGGTAACGCCAGGGTTTTCCCAGTCACGACGTTGTAAAACGACGGCCAGTGAGCGCGACGT
AATACGACTCACTATAGGGCGAATTGGCGGAAGGCCGTCAAGGCCTAGGTGGAACTGCCCTGCTCCCACAGTTACATACA
GGCTAGGGAGTGGGTAGGAGCCTAGGCCATAAGGTAAAGAGGACACCAAATACAGTAAATAATAACATAATAATAAAGAA
ACCTTATGAATTCTATTCAGTGATATCCATATTGAGGTATTTAAGGTGAATGATACTGATGTCAGCAGTTTACCTGGATA
TGCATCAAAAATATGATTGATTGATGGATGGATCGGAGGAAGGAAAAGATATGTGATCAAAACGAGTATAGTAAAATGTT
CATGGTGGAATCTAGATATATAGATATCACTTGTAATTTTTAAAAATGTTATTGCATTTTTGAAATTTGTTATGTTAAAA
TATTGGGTGAAAATTCTGATGCCCTGGCCATGTCCCAAATCAGTTGAATCAGATCTCTGGTGTATTTTTTTTTTAAGATC
CTTGGATGACTCCATTGTACAGCAAGTTTGCAAACCACTATTCTAACTTCTCACCACCCCCTTCTTTATAGCTTACAAAA
TTATATTTTTAAATGTCAAATGTCACAATTTCACTTTCCTTCTTCAAGAACTGATTAGACTTCTAGCTTCTCCTATGCTC
TCATCTTGCATAACGTTCATTTGCACGTGGTTAAAACTGATAGGCAATAAATAATAACCCATCTGTCTTTTCCTGACCCA
ACATAACTAAAACCTTCCTTTCAAGGCGAAAATGAACTATAATCTCACAGACAAGAGTAGCTATAGACAACTAATTCTTT
CTGGTCTCTCGTGGTCATGGGACTATTCACCACTGCCGTCCCTGCTGTCTATTTGGTGGACCAGCCAGTTTTACTGTGAC
CCATACCATGGCTCTTGAGAAGCATGAAGCTGCCGAAATCGCAGTTGTAGGGTTAATGGAAAACATGGTGAAGTATAGCT
TACATATATATACAATTTTGGCACAAAATGAAGCCTAAGAAATTGTACCGGGCTGGGTGTGGTGACTCATGCCTGTAATC
CCAGCACTTTGAGAGACCAAGGTGGGCGGATCACCTGAGGTCGGGAGTTCGAGACCAGACTGAGCAACATGGAGAAACCC
TCTCTACTTAAAATACAAAATTAGCCAGGCATGGTGGCGCATGCCTGTAACCCCAGCTACTCAGGAGGCTAAGGCAAGAG
AATCTCTTCCACCCTGGAGGCATAGGTTGTGGTGAGAGAAGATCACACCATTGCACTCCAGCTGGGGCAACAAGAGCGAA
```

GCTCCGTCTCAATAAATAAATAAATAAATAAATAAATAAATAAATAAATAAATAAATAAAAGAAAAAGAAATTGTACCAG
GAAGTGGATTGGGAATGCTCTATATATTCTCCCCCTCCAAGAAGTTTTTTTAATTTATTGTATTGTGAAAGATCTGCAGC
TATTTATTTTGTGTGGTACTCTTTTGCAATAAACTACTTCTGCATAAATGAACTCAAAAACTTTGATATTCATGAACCTT
CATAATGTTGATCTAGTACATCTTGCCCAACTAGCCCTTGGGCCATTCCTTCCATGCCACTGGTTGAAAAATATGCTCCC
CACCCCAGTAACATCAGCATCAGACCTCTGCAACCAGAAAGATTCCATAGGTAATGAAATCCAAATGCTGGCATTTGGAA
GTCAAACCAGTGTATTTTGAAATGGAAAGGCAAGGGGAAGTGCATGGAGCAAGTACAGCATGTGCTACAACTTCAGTTCA
TACCCTCCTTAAAATACTGTGCAACTAAACTGTCAATTGCCCATTGATAGAGATAGTATCTTTTTCAGCTTTACACCTTC
CGAATTCAACACACTGCTAGCCTCAGGACTAAGCGCTCAATAAATGTTTATCAAAAGATTGAGTAAATGAATCAGTGGTG
AAATAAAATGTACAAACACATACGCTTCATACACGTTTAGGAAGCATTTCTAGTGAGCTTTGTGGATATTTTATTTCTTA
AAGGTTCTTTTTCTTTCCATGTTCATGTTATTCTTTATTTTTTAAAATGTTGCTTCTGCTTATTTCTCTTTCTACGGCAG
CTATATTTACTTGCCTGAGAGCACAGCATATACTGATGTTAATCAAACTTTAGATGAAACACCTAAAATGATAGAAAAGA
ATCTGCTCTAATTAAATAATAAAGCAACCCCTACAGGTTTAGACATGTGCCTTCCGGTGTGGGAAAGAAAAAATTTAATG
AATGTAGTAGTTTTATGCCAAGAACATTTCCCTCGTGCACCTGTGTTTTAATAAGATAGAATATAAAATAGCAAAAGGGG
CCCGACTTTTGTGATGATATTTACTCATAAGGTAGTAAGTCAGATGTGATGACATTTTCTTTGACTTAGAGCTGCTATAC
TTGGGTCAGATTTCAGTTCAGTAAATTTGCAGTGAAGTTGTCTTTCTAACATGGTGTCATCCTGGAACTGCCCTGCTCCC
ACAGTAAGCTTCCACGGGGTTGGGGTTGCGCCTTTTCCAAGGCAGCCCTGGGTTTGCGCAGGGACGCGGCTGCTCTGGGC
GTGGTTCCGGGAAACGCAGCGGCGCCGACCCTGGGTCTCGCACATTCTTCACGTCCGTTCGCAGCGTCACCCGGATCTTC
GCCGCTACCCTTGTGGGCCCCCCGGCGACGCTTCCTGCTCCGCCCCTAAGTCGGGAAGGTTCCTTGCGGTTCGCGGCGTG
CCGGACGTGACAAACGGAAGCCGCACGTCTCACTAGTACCCTCGCAGACGGACAGCGCCAGGGAGCAATGGCAGCGCGCC
GACCGCGATGGGCTGTGGCCAATAGCGGCTGCTCAGCAGGGCGCGCCGAGAGCAGCGGCCGGGAAGGGGCGGTGCGGGAG
GCGGGGTGTGGGGCGGTAGTGTGGGCCCTGTTCCTGCCCGCGCGGTGTTCCGCATTCTGCAAGCCTCCGGAGCGCACGTC
GGCAGTCGGCTCCCTCGTTGACCGAATCACCGACCTCTCTCCCCACACGACTCTAGAGGATCCCCGGGTACCGGTCGCCA
CCATGGTGAGCAAGGGCGAGGAGCTGTTCACCGGGGTGGTGCCCATCCTGGTCGAGCTGGACGGCGACGTAAACGGCCAC
AAGTTCAGCGTGTCCGGCGAGGGCGAGGGCGATGCCACCTACGGCAAGCTGACCCTGAAGTTCATCTGCACCACCGGCAA
GCTGCCCGTGCCCTGGCCCACCCTCGTGACCACCCTGACCTACGGCGTGCAGTGCTTCAGCCGCTACCCCGACCACATGA
AGCAGCACGACTTCTTCAAGTCCGCCATGCCCGAAGGCTACGTCCAGGAGCGCACCATCTTCTTCAAGGACGACGGCAAC
TACAAGACCCGCGCCGAGGTGAAGTTCGAGGGCGACACCCTGGTGAACCGCATCGAGCTGAAGGGCATCGACTTCAAGGA
GGACGGCAACATCCTGGGGCACAAGCTGGAGTACAACTACAACAGCCACAACGTCTATATCATGGCCGACAAGCAGAAGA
ACGGCATCAAGGTGAACTTCAAGATCCGCCACAACATCGAGGACGGCAGCGTGCAGCTCGCCGACCACTACCAGCAGAAC
ACCCCCATCGGCGACGGCCCCGTGCTGCTGCCCGACAACCACTACCTGAGCACCCAGTCCGCCCTGAGCAAAGACCCCAA
CGAGAAGCGCGATCACATGGTCCTGCTGGAGTTCGTGACCGCCGCCGGGATCACTCTCGGCATGGACGAGCTGTACAAGT
AAAGCGGCCGCGACTCTAGAATTTTAAGCTGTGCCTTCTAGTTGCCAGCCATCTGTTGTTTGCCCCTCCCCCGTGCCTTC
CTTGACCCTGGAAGGTGCCACTCCCACTGTCCTTTCCTAATAAAATGAGGAAATTGCATCGCATTGTCTGAGTAGGTGTC
ATTCTATTCTGGGGGGTGGGGTGGGGCAGGACAGCAAGGGGGAGGATTGGGAAGACAATAGCAGGCATGCACGTGTACAG
GCTAGGGAGTGGGTAGGAGTGGGGGTGAAATCCTCTTAATGTTTATGGTGTCAGTAGATTCAAACTAAAATTAGCCTTAC
AGCCATACTCCTAATAAGGGGCCCCTGGCATATTTAATTGATTTAACAAATTTATCAAAAATAGATAAACTGAAATCTGC
CTTGAAATTAATTACTGTATCTCTATTTTTATAAGAAAATATTTTGGACCCGTTCCCTCTGCCTTATGGGTGCAACTCCC
GGCAGAATAGGTCATGTCTCCTGAAAATAGTTCTTATTTTCTTTACTACTTATGACCTCTTATAGCCTAGAAGTTTTTCC
TTGCTATTTCTAGTTGAAATGCTAATCTGGCATAATTTCTAGTTGAAATGCTAATCTGGCATAATTTCAGAACTAATTTT
CTGTTAATGCCACTTGGAACATCTAAATTCCTCCTTTTTCAATAATACTATATTTGTGTGTTGCAAACACACACAAATCA
TACTATATTTGTGTGTATATATAGTAAATTCCAACTTATAAAAGTAAAATTGGAATTTTTTTCTTTCTTTGGAAAATTAT
TTTATAATTGAGAATTATAAATGTCACTTTTTTAAATGCTGCAACCTTTGAGATTGGTTTCAATAAAGTAAAACTTAGTA
AACATTAAGAAAAATGATAGCTTGATATGTTCACTAATATGGTAAATGAAAACTTTTATGTGTGTATAGGCTCTTGTTAA
TTATAACTACCTTCACAGGAAAAACAGTCTTGTGGAAGGTAATGGTGCCAGTGAGAGAAAATAGAGAAAGTAGAATAGTA
GTAAGAGAGAAAATAGAATTTCCCGTTTAGAATAAAGCTAAGTAAAAAGTGAATTGTTTCAGTTTTATTTATGTATTTGT
AAGTGTGGTCTCATGCCATATATATTCAAAGATAGACAGAGATCTTAATCTTTAATTTTTTATGGCCAAAGAAAATGAGT
CCCATGTAAAAGGGACATTTTCGTAAGCTGATCTCAAGTGGTGTATTTTGATTTGCCGCAGCAGCCTATGGAAGAAATAT
CATTTTGGCTGCCTAAGAAGAAAAATGTCATACCTTTCTATTTTTTATTCATACTCCGCATATTTGGAATTCGTATTCTC
ACCATACTTTTGGGAAGATAACACGAAATTGCCTTACTTGCCTCGAAATCAAAATCTGCCATTCGTTTTAAAATAAAATG
GCTTTTTCATCTCATTTTCCTTTAGCAGAAAAAAGTAAGTTAAATTTTTTTGTAGCTGTGTTAAGTGTACTGTTCCTCCC
TCGTTGAGAACGAAACAAAGACATATGATTCCATTTACTGTAATTGTTTTGGTTTGCCCAATTACAATAGCAGTAAAATC
ATTTACACATAGTAAAATGTTTGAGCCTTAAAGGGCAAGAAGACTAAATTTAGCTGAAGATATACAACTTTCATGTGCCA
ATGAGGAAATGTATAAAATCAGTTATACATATTTTCCTCATTCATAGATATTGAAAATATTCATATATGTGTACATGTAT
TTGATAACTTAATATTTTCTAAATAACAGAAATTATATACTGTATATATATTTGGATATTATGATTATTGTTATTGCCAC
CATTGAGCACTTACTATGGGCTCAGAAAACTTCACTGTATTATTTTTAATTCTTATAACAACCCTGTGAAATATATATTA
TGCCTATTTCACAAATGAGAACTCTGAAATCAGGAAATTAAATCACTTGCTGAAGTTTACAAAGCACTGTTAGGCAATAA
AACAGAATTTCAAACTCACGTTTGCTTGTCTTCAAATTTTGTATGCTGTACAACCTGATTGTTTTACTACTTGTTCTTAA
TTTCAAAAAAAATCTTCATACTAAAAGATGATACTTTGGGAGTTCTAAAGAACATGTTTTTGGCCGGGCGCGGTGGCTCA
CGCCTGTAATCCCAGCACTTTGGGAGGCCGAGGCAGAAGGATCACGAGGTCAGGAGATCGAGACCGTCCTGGCCAACAAG
GTGAAACCCCGTCTCTACTAAAAAATACAAAAAATTAGCCGGGCGTGGTGGCGGGCGCCTGTAGTCCCAGCTACTCGGGA
AGCTGAGGCGGGAGAAAGGCGTGAACCTGGGAGGCAGAGCTTGTAGTGAGCCGAGATCGCGCCGCTGCACTCCAGCCTGG
TTGACAGAGCGAGACTCCGTCTCAAAAATAACAAAACAAAACAAAACAAAACAAAAAAAACCATGTTTCTTTTCAGAAGG
TATACTCATTTGAAGTGGATACCAATTATTTGTATTAAAATTACTTATGGATAAATTTGAATCTGCAAAAATTAAGTGCA
ACATTATTTTTTGGCACCTGTTATAGGAGGGTAACTCGAGCTCCTACCCACTCCCTAGCCTGTATGTAACTGTGGGAGCA
GGGCAGTTCCACTCGAGGGTACCTCTTAATTAACTGGCCTCATGGGCCTTCCGCTCACTGCCCGCTTTCCAGTCGGGAAA
CCTGTCGTGCCAGCTGCATTAACATGGTCATAGCTGTTTCCTTGCGTATTGGGCGCTCTCCGCTTCCTCGCTCACTGACT
CGCTGCGCTCGGTCGTTCGGGTAAAGCCTGGGGTGCCTAATGAGCAAAAGGCCAGCAAAAGGCCAGGAACCGTAAAAAGG
CCGCGTTGCTGGCGTTTTTCCATAGGCTCCGCCCCCCTGACGAGCATCACAAAAATCGACGCTCAAGTCAGAGGTGGCGA

```
AACCCGACAGGACTATAAAGATACCAGGCGTTTCCCCCTGGAAGCTCCCTCGTGCGCTCTCCTGTTCCGACCCTGCCGCT
TACCGGATACCTGTCCGCCTTTCTCCCTTCGGGAAGCGTGGCGCTTTCTCATAGCTCACGCTGTAGGTATCTCAGTTCGG
TGTAGGTCGTTCGCTCCAAGCTGGGCTGTGTGCACGAACCCCCCGTTCAGCCCGACCGCTGCGCCTTATCCGGTAACTAT
CGTCTTGAGTCCAACCCGGTAAGACACGACTTATCGCCACTGGCAGCAGCCACTGGTAACAGGATTAGCAGAGCGAGGTA
TGTAGGCGGTGCTACAGAGTTCTTGAAGTGGTGGCCTAACTACGGCTACACTAGAAGAACAGTATTTGGTATCTGCGCTC
TGCTGAAGCCAGTTACCTTCGGAAAAAGAGTTGGTAGCTCTTGATCCGGCAAACAAACCACCGCTGGTAGCGGTGGTTTT
TTTGTTTGCAAGCAGCAGATTACGCGCAGAAAAAAAGGATCTCAAGAAGATCCTTTGATCTTTTCTACGGGGTCTGACGC
TCAGTGGAACGAAAACTCACGTTAAGGGATTTTGGTCATGAGATTATCAAAAAGGATCTTCACCTAGATCCTTTTAAATT
AAAAATGAAGTTTTAAATCAATCTAAAGTATATATGAGTAAACTTGGTCTGACAGTTACCAATGCTTAATCAGTGAGGCA
CCTATCTCAGCGATCTGTCTATTTCGTTCATCCATAGTTGCCTGACTCCCCGTCGTGTAGATAACTACGATACGGGAGGG
CTTACCATCTGGCCCCAGTGCTGCAATGATACCGCGAGAACCACGCTCACCGGCTCCAGATTTATCAGCAATAAACCAGC
CAGCCGGAAGGGCCGAGCGCAGAAGTGGTCCTGCAACTTTATCCGCCTCCATCCAGTCTATTAATTGTTGCCGGGAAGCT
AGAGTAAGTAGTTCGCCAGTTAATAGTTTGCGCAACGTTGTTGCCATTGCTACAGGCATCGTGGTGTCACGCTCGTCGTT
TGGTATGGCTTCATTCAGCTCCGGTTCCCAACGATCAAGGCGAGTTACATGATCCCCCATGTTGTGCAAAAAAGCGGTTA
GCTCCTTCGGTCCTCCGATCGTTGTCAGAAGTAAGTTGGCCGCAGTGTTATCACTCATGGTTATGGCAGCACTGCATAAT
TCTCTTACTGTCATGCCATCCGTAAGATGCTTTTCTGTGACTGGTGAGTACTCAACCAAGTCATTCTGAGAATAGTGTAT
GCGGCGACCGAGTTGCTCTTGCCCGGCGTCAATACGGGATAATACCGCGCCACATAGCAGAACTTTAAAAGTGCTCATCA
TTGGAAAACGTTCTTCGGGGCGAAAACTCTCAAGGATCTTACCGCTGTTGAGATCCAGTTCGATGTAACCCACTCGTGCA
CCCAACTGATCTTCAGCATCTTTTACTTTCACCAGCGTTTCTGGGTGAGCAAAAACAGGAAGGCAAAATGCCGCAAAAAA
GGGAATAAGGGCGACACGGAAATGTTGAATACTCATACTCTTCCTTTTTCAATATTATTGAAGCATTTATCAGGGTTATT
GTCTCATGAGCGGATACATATTTGAATGTATTTAGAAAAATAAACAAATAGGGGTTCCGCGCACATTTCCCCGAAAAGTG
CCAC
```

Supplementary Figure 26. Map and nucleotide sequence of the unmodified donor construct targeting the human *DMD* locus. DNA sequences sharing identity to the human *DMD* gene are indicated in orange; Magenta arrows, gRNA^{DMD} target site (TS); hPGK1 promoter, human phosphoglycerate kinase 1 gene (*PGK1*) regulatory sequences; EGFP, enhanced green fluorescence protein gene (*EGFP*) open reading frame; bGH1 poly(A) signal, bovine growth hormone gene (*GH1*) polyadenylation signal; AmpR, *β-lactamase* ampicillin resistance gene; ori, high-copy number ColE1 prokaryotic origin of replication.

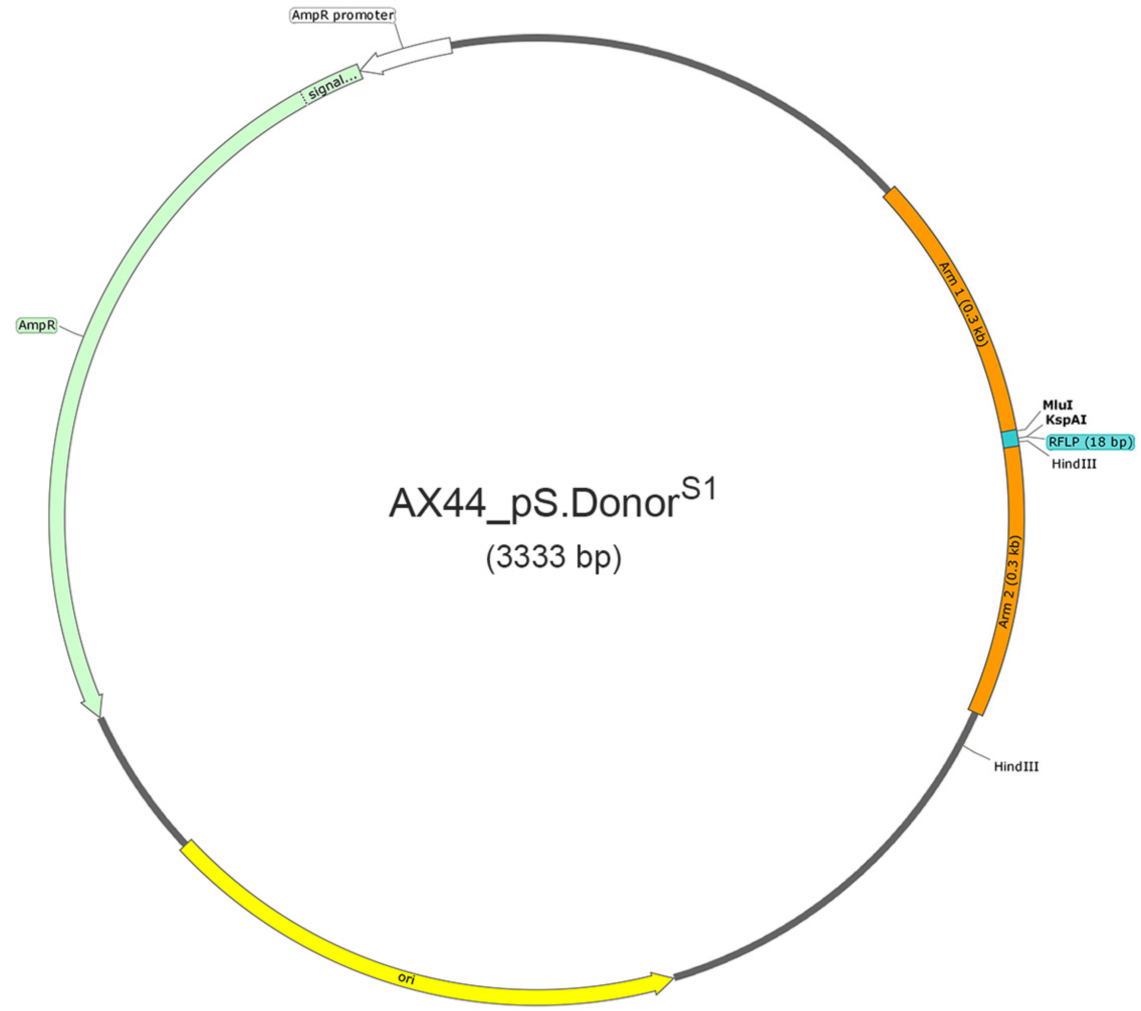

>AX44_pS.Donor.S1 (3333 bp)
TCGCGCGTTTCGGTGATGACGGTGAAAACCTCTGACACATGCAGCTCCCGGAGACGGTCACAGCTTGTCTGTAAGCGGAT
GCCGGGAGCAGACAAGCCCGTCAGGGCGCGTCAGCGGGTGTTGGCGGGTGTCGGGGCTGGCTTAACTATGCGGCATCAGA
GCAGATTGTACTGAGAGTGCACCATATGCGGTGTGAAATACCGCACAGATGCGTAAGGAGAAAATACCGCATCAGGCGCC
ATTCGCCATTCAGGCTGCGCAACTGTTGGGAAGGGCGATCGGTGCGGGCCTCTTCGCTATTACGCCAGCTGGCGAAAGGG
GGATGTGCTGCAAGGCGATTAAGTTGGGTAACGCCAGGGTTTTCCCAGTCACGACGTTGTAAAACGACGGCCAGTGAATT
CGAGCTCGGTACCTCGCGAATGCATCTAGATGGCCGCGGATTCGGGTCACCTCTCACTCCTTTCATTTGGGCAGCTCCCC
TACCCCCCTTACCTCTCTAGTCTGTGCTAGCTCTTCCAGCCCCCTGTCATGGCATCTTCCAGGGGTCCGAGAGCTCAGCT
AGTCTTCTTCCTCCAACCCGGGCCCCTATGTCCACTTCAGGACAGCATGTTTGCTGCCTCCAGGGATCCTGTGTCCCCGA
GCTGGGACCACCTTATATTCCCAGGGCCGGTTAATGTGGCTCTGGTTCTGGGTACTTTTATCTGTCCCCTCCACCCCACA
GTGGGGCCACTAGGGACACGCGTGTTAACAAGCTTAGGATTGGTGACAGAAAAGCCCCATCCTTAGGCCTCCTCCTTCCT
AGTCTCCTGATATTGGGTCTAACCCCCACCTCCTGTTAGGCAGATTCCTTATCTGGTGACACACCCCCATTTCCTGGAGC
CATCTCTCTCCTTGCCAGAACCTCTAAGGTTTGCTTACGATGGAGCCAGAGAGGATCCTGGGAGGGAGAGCTTGGCAGGG
GGTGGGAGGGAAGGGGGGGATGCGTGACCTGCCCGGTTCTCAGTGGCCACCCTGCGCTACCCTCTCCCAGAACCTGAGCT
GCTCTGACGCGGCCATCGGATCCCGGGCCCGTCGACTGCAGAGGCCTGCATGCAAGCTTGGCGTAATCATGGTCATAGCT
GTTTCCTGTGTGAAATTGTTATCCGCTCACAATTCCACACAACATACGAGCCGGAAGCATAAAGTGTAAAGCCTGGGGTG
CCTAATGAGTGAGCTAACTCACATTAATTGCGTTGCGCTCACTGCCCGCTTTCCAGTCGGGAAACCTGTCGTGCCAGCTG
CATTAATGAATCGGCCAACGCGCGGGGAGAGGCGGTTTGCGTATTGGGCGCTCTTCCGCTTCCTCGCTCACTGACTCGCT
GCGCTCGGTCGTTCGGCTGCGGCGAGCGGTATCAGCTCACTCAAAGGCGGTAATACGGTTATCCACAGAATCAGGGGATA
ACGCAGGAAAGAACATGTGAGCAAAAGGCCAGCAAAAGGCCAGGAACCGTAAAAAGGCCGCGTTGCTGGCGTTTTTCCAT
AGGCTCCGCCCCCCTGACGAGCATCACAAAAATCGACGCTCAAGTCAGAGGTGGCGAAACCCGACAGGACTATAAAGATA
CCAGGCGTTTCCCCCTGGAAGCTCCCTCGTGCGCTCTCCTGTTCCGACCCTGCCGCTTACCGGATACCTGTCCGCCTTTC
TCCCTTCGGGAAGCGTGGCGCTTTCTCATAGCTCACGCTGTAGGTATCTCAGTTCGGTGTAGGTCGTTCGCTCCAAGCTG
GGCTGTGTGCACGAACCCCCCGTTCAGCCCGACCGCTGCGCCTTATCCGGTAACTATCGTCTTGAGTCCAACCCGGTAAG
ACACGACTTATCGCCACTGGCAGCAGCCACTGGTAACAGGATTAGCAGAGCGAGGTATGTAGGCGGTGCTACAGAGTTCT

```
TGAAGTGGTGGCCTAACTACGGCTACACTAGAAGAACAGTATTTGGTATCTGCGCTCTGCTGAAGCCAGTTACCTTCGGA
AAAAGAGTTGGTAGCTCTTGATCCGGCAAACAAACCACCGCTGGTAGCGGTGGTTTTTTTGTTTGCAAGCAGCAGATTAC
GCGCAGAAAAAAAGGATCTCAAGAAGATCCTTTGATCTTTTCTACGGGGTCTGACGCTCAGTGGAACGAAAACTCACGTT
AAGGGATTTTGGTCATGAGATTATCAAAAAGGATCTTCACCTAGATCCTTTTAAATTAAAAATGAAGTTTTAAATCAATC
TAAAGTATATATGAGTAAACTTGGTCTGACAGTTACCAATGCTTAATCAGTGAGGCACCTATCTCAGCGATCTGTCTATT
TCGTTCATCCATAGTTGCCTGACTCCCCGTCGTGTAGATAACTACGATACGGGAGGGCTTACCATCTGGCCCCAGTGCTG
CAATGATACCGCGAGACCCACGCTCACCGGCTCCAGATTTATCAGCAATAAACCAGCCAGCCGGAAGGGCCGAGCGCAGA
AGTGGTCCTGCAACTTTATCCGCCTCCATCCAGTCTATTAATTGTTGCCGGGAAGCTAGAGTAAGTAGTTCGCCAGTTAA
TAGTTTGCGCAACGTTGTTGCCATTGCTACAGGCATCGTGGTGTCACGCTCGTCGTTTGGTATGGCTTCATTCAGCTCCG
GTTCCCAACGATCAAGGCGAGTTACATGATCCCCCATGTTGTGCAAAAAAGCGGTTAGCTCCTTCGGTCCTCCGATCGTT
GTCAGAAGTAAGTTGGCCGCAGTGTTATCACTCATGGTTATGGCAGCACTGCATAATTCTCTTACTGTCATGCCATCCGT
AAGATGCTTTTCTGTGACTGGTGAGTACTCAACCAAGTCATTCTGAGAATAGTGTATGCGGCGACCGAGTTGCTCTTGCC
CGGCGTCAATACGGGATAATACCGCGCCACATAGCAGAACTTTAAAAGTGCTCATCATTGGAAAACGTTCTTCGGGGCGA
AAACTCTCAAGGATCTTACCGCTGTTGAGATCCAGTTCGATGTAACCCACTCGTGCACCCAACTGATCTTCAGCATCTTT
TACTTTCACCAGCGTTTCTGGGTGAGCAAAAACAGGAAGGCAAAATGCCGCAAAAAAGGGAATAAGGGCGACACGGAAAT
GTTGAATACTCATACTCTTCCTTTTTCAATATTATTGAAGCATTTATCAGGGTTATTGTCTCATGAGCGGATACATATTT
GAATGTATTTAGAAAAATAAACAAATAGGGGTTCCGCGCACATTTCCCCGAAAAGTGCCACCTGACGTCTAAGAAACCAT
TATTATCATGACATTAACCTATAAAAATAGGCGTATCACGAGGCCCTTTCGTC
```

Supplementary Figure 27. Map and nucleotide sequence of *AAVS1*-targeting plasmid AX44_pS.Donor[S1]. Orange regions, sequences homologous to the human *AAVS1* "safe harbour" locus; RFLP, restriction fragment length polymorphism; AmpR, *β-lactamase* ampicillin resistance gene; high-copy number ColE1 origin of replication.

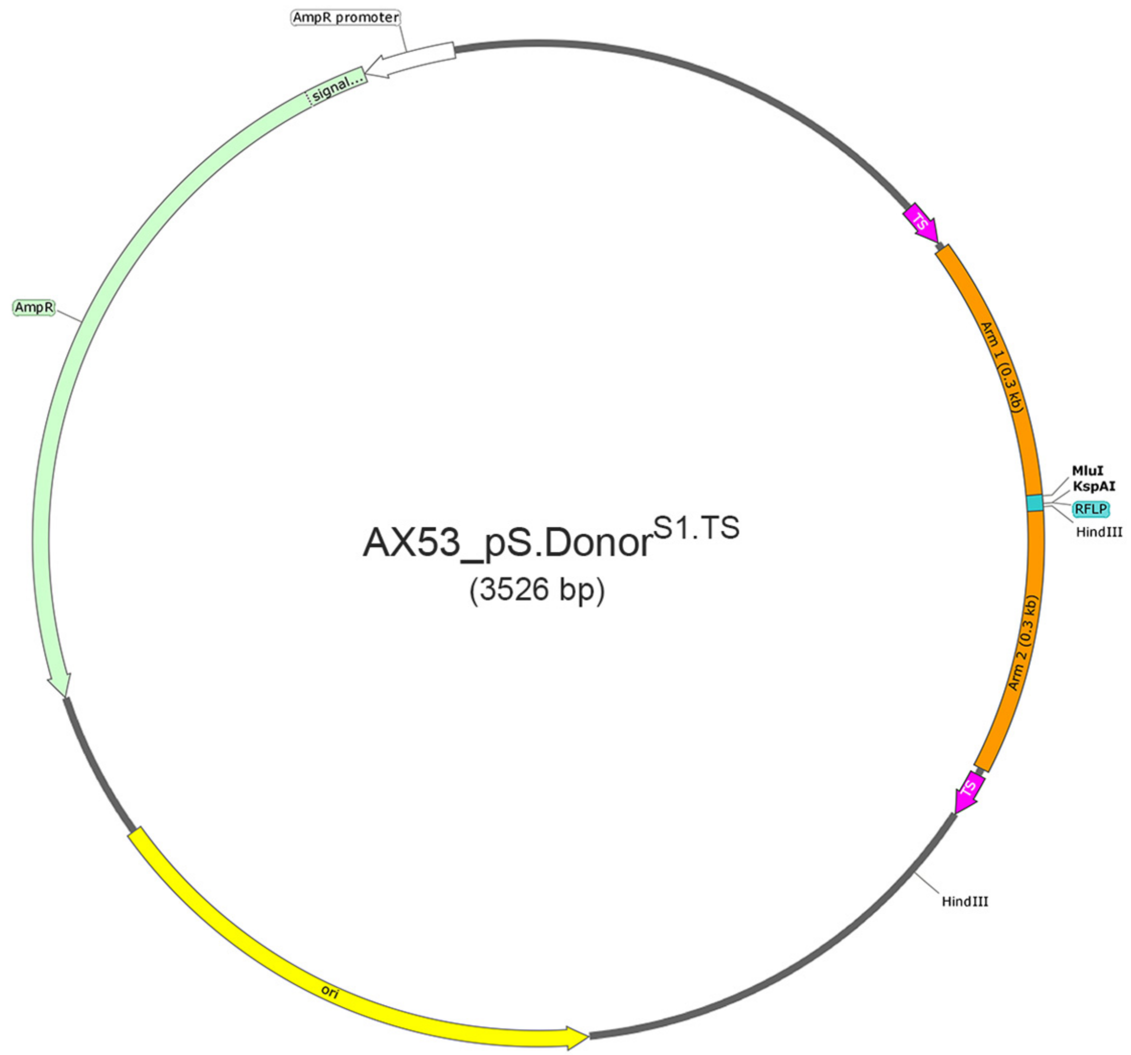

>AX53_pS.Donor.S1.TS (3526 bp)
TCGCGCGTTTCGGTGATGACGGTGAAAACCTCTGACACATGCAGCTCCCGGAGACGGTCACAGCTTGTCTGTAAGCGGAT
GCCGGGAGCAGACAAGCCCGTCAGGGCGCGTCAGCGGGTGTTGGCGGGTGTCGGGGCTGGCTTAACTATGCGGCATCAGA
GCAGATTGTACTGAGAGTGCACCATATGCGGTGTGAAATACCGCACAGATGCGTAAGGAGAAAATACCGCATCAGGCGCC
ATTCGCCATTCAGGCTGCGCAACTGTTGGGAAGGGCGATCGGTGCGGGCCTCTTCGCTATTACGCCAGCTGGCGAAAGGG
GGATGTGCTGCAAGGCGATTAAGTTGGGTAACGCCAGGGTTTTCCCAGTCACGACGTTGTAAAACGACGGCCAGTGAATT
CGAGCTCGGTACCTCGCGAATGCATCTAGATATCTAGGGATAACAGGGTAATGTCGAGGCCGGATTTAAATCTGTCCCCT
CCACCCCACAGTGGGGCCACTAGGGACAGGATTGGTGACAGAGCGGCCGCGGATTCGGGTCACCTCTCACTCCTTTCATT
TGGGCAGCTCCCCTACCCCCCTTACCTCTCTAGTCTGTGCTAGCTCTTCCAGCCCCCTGTCATGGCATCTTCCAGGGGTC
CGAGAGCTCAGCTAGTCTTCTTCCTCCAACCCGGGCCCCTATGTCCACTTCAGGACAGCATGTTTGCTGCCTCCAGGGAT
CCTGTGTCCCCGAGCTGGGACCACCTTATATTCCCAGGGCCGGTTAATGTGGCTCTGGTTCTGGGTACTTTTATCTGTCC
CCTCCACCCCACAGTGGGGCCACTAGGGACACGCGTGTTAACAAGCTTAGGATTGGTGACAGAAAAGCCCCATCCTTAGG
CCTCCTCCTTCCTAGTCTCCTGATATTGGGTCTAACCCCCACCTCCTGTTAGGCAGATTCCTTATCTGGTGACACACCCC
CATTTCCTGGAGCCATCTCTCTCCTTGCCAGAACCTCTAAGGTTTGCTTACGATGGAGCCAGAGAGGATCCTGGGAGGGA
GAGCTTGGCAGGGGGTGGAGGGAAGGGGGGGATGCGTGACCTGCCCGGTTCTCAGTGGCCACCCTGCGCTACCCTCTCC
CAGAACCTGAGCTGCTCTGACGCGGCCGCGCCGGCGCTGTCCCCTCCACCCCACAGTGGGGCCACTAGGGACAGGATTGG
TGACAGAGTTTAAACTAGGGATAACAGGGTAATGTCGAGGCCGGGATATCGGATCCCGGGCCCGTCGACTGCAGAGGCCT
GCATGCAAGCTTGGCGTAATCATGGTCATAGCTGTTTCCTGTGTGAAATTGTTATCCGCTCACAATTCCACACAACATAC
GAGCCGGAAGCATAAAGTGTAAAGCCTGGGGTGCCTAATGAGTGAGCTAACTCACATTAATTGCGTTGCGCTCACTGCCC
GCTTTCCAGTCGGGAAACCTGTCGTGCCAGCTGCATTAATGAATCGGCCAACGCGCGGGGAGAGGCGGTTTGCGTATTGG
GCGCTCTTCCGCTTCCTCGCTCACTGACTCGCTGCGCTCGGTCGTTCGGCTGCGGCGAGCGGTATCAGCTCACTCAAAGG
CGGTAATACGGTTATCCACAGAATCAGGGGATAACGCAGGAAAGAACATGTGAGCAAAAGGCCAGCAAAAGGCCAGGAAC
CGTAAAAAGGCCGCGTTGCTGGCGTTTTTCCATAGGCTCCGCCCCCCTGACGAGCATCACAAAAATCGACGCTCAAGTCA
GAGGTGGCGAAACCCGACAGGACTATAAAGATACCAGGCGTTTCCCCCTGGAAGCTCCCTCGTGCGCTCTCCTGTTCCGA
CCCTGCCGCTTACCGGATACCTGTCCGCCTTTCTCCCTTCGGGAAGCGTGGCGCTTTCTCATAGCTCACGCTGTAGGTAT

CTCAGTTCGGTGTAGGTCGTTCGCTCCAAGCTGGGCTGTGTGCACGAACCCCCCGTTCAGCCCGACCGCTGCGCCTTATC
CGGTAACTATCGTCTTGAGTCCAACCCGGTAAGACACGACTTATCGCCACTGGCAGCAGCCACTGGTAACAGGATTAGCA
GAGCGAGGTATGTAGGCGGTGCTACAGAGTTCTTGAAGTGGTGGCCTAACTACGGCTACACTAGAAGAACAGTATTTGGT
ATCTGCGCTCTGCTGAAGCCAGTTACCTTCGGAAAAAGAGTTGGTAGCTCTTGATCCGGCAAACAAACCACCGCTGGTAG
CGGTGGTTTTTTTGTTTGCAAGCAGCAGATTACGCGCAGAAAAAAAGGATCTCAAGAAGATCCTTTGATCTTTTCTACGG
GGTCTGACGCTCAGTGGAACGAAAACTCACGTTAAGGGATTTTGGTCATGAGATTATCAAAAAGGATCTTCACCTAGATC
CTTTTAAATTAAAAATGAAGTTTTAAATCAATCTAAAGTATATATGAGTAAACTTGGTCTGACAGTTACCAATGCTTAAT
CAGTGAGGCACCTATCTCAGCGATCTGTCTATTTCGTTCATCCATAGTTGCCTGACTCCCCGTCGTGTAGATAACTACGA
TACGGGAGGGCTTACCATCTGGCCCCAGTGCTGCAATGATACCGCGAGACCCACGCTCACCGGCTCCAGATTTATCAGCA
ATAAACCAGCCAGCCGGAAGGGCCGAGCGCAGAAGTGGTCCTGCAACTTTATCCGCCTCCATCCAGTCTATTAATTGTTG
CCGGGAAGCTAGAGTAAGTAGTTCGCCAGTTAATAGTTTGCGCAACGTTGTTGCCATTGCTACAGGCATCGTGGTGTCAC
GCTCGTCGTTTGGTATGGCTTCATTCAGCTCCGGTTCCCAACGATCAAGGCGAGTTACATGATCCCCCATGTTGTGCAAA
AAAGCGGTTAGCTCCTTCGGTCCTCCGATCGTTGTCAGAAGTAAGTTGGCCGCAGTGTTATCACTCATGGTTATGGCAGC
ACTGCATAATTCTCTTACTGTCATGCCATCCGTAAGATGCTTTTCTGTGACTGGTGAGTACTCAACCAAGTCATTCTGAG
AATAGTGTATGCGGCGACCGAGTTGCTCTTGCCCGGCGTCAATACGGGATAATACCGCGCCACATAGCAGAACTTTAAAA
GTGCTCATCATTGGAAAACGTTCTTCGGGGCGAAAACTCTCAAGGATCTTACCGCTGTTGAGATCCAGTTCGATGTAACC
CACTCGTGCACCCAACTGATCTTCAGCATCTTTTACTTTCACCAGCGTTTCTGGGTGAGCAAAAACAGGAAGGCAAAATG
CCGCAAAAAAGGGAATAAGGGCGACACGGAAATGTTGAATACTCATACTCTTCCTTTTTCAATATTATTGAAGCATTTAT
CAGGGTTATTGTCTCATGAGCGGATACATATTTGAATGTATTTAGAAAAATAAACAAATAGGGGTTCCGCGCACATTTCC
CCGAAAAGTGCCACCTGACGTCTAAGAAACCATTATTATCATGACATTAACCTATAAAAATAGGCGTATCACGAGGCCCT
TTCGTC

Supplementary Figure 28. Map and nucleotide sequence of *AAVS1*-targeting plasmid AX53_pS.Donor$^{S1.TS}$. Orange regions, sequences homologous to the human *AAVS1* “safe harbour” locus; RFLP, restriction fragment length polymorphism; Magenta arrows, gRNA^{S1} target site (TS); AmpR, *β-lactamase* ampicillin resistance gene; high-copy number ColE1 origin of replication.

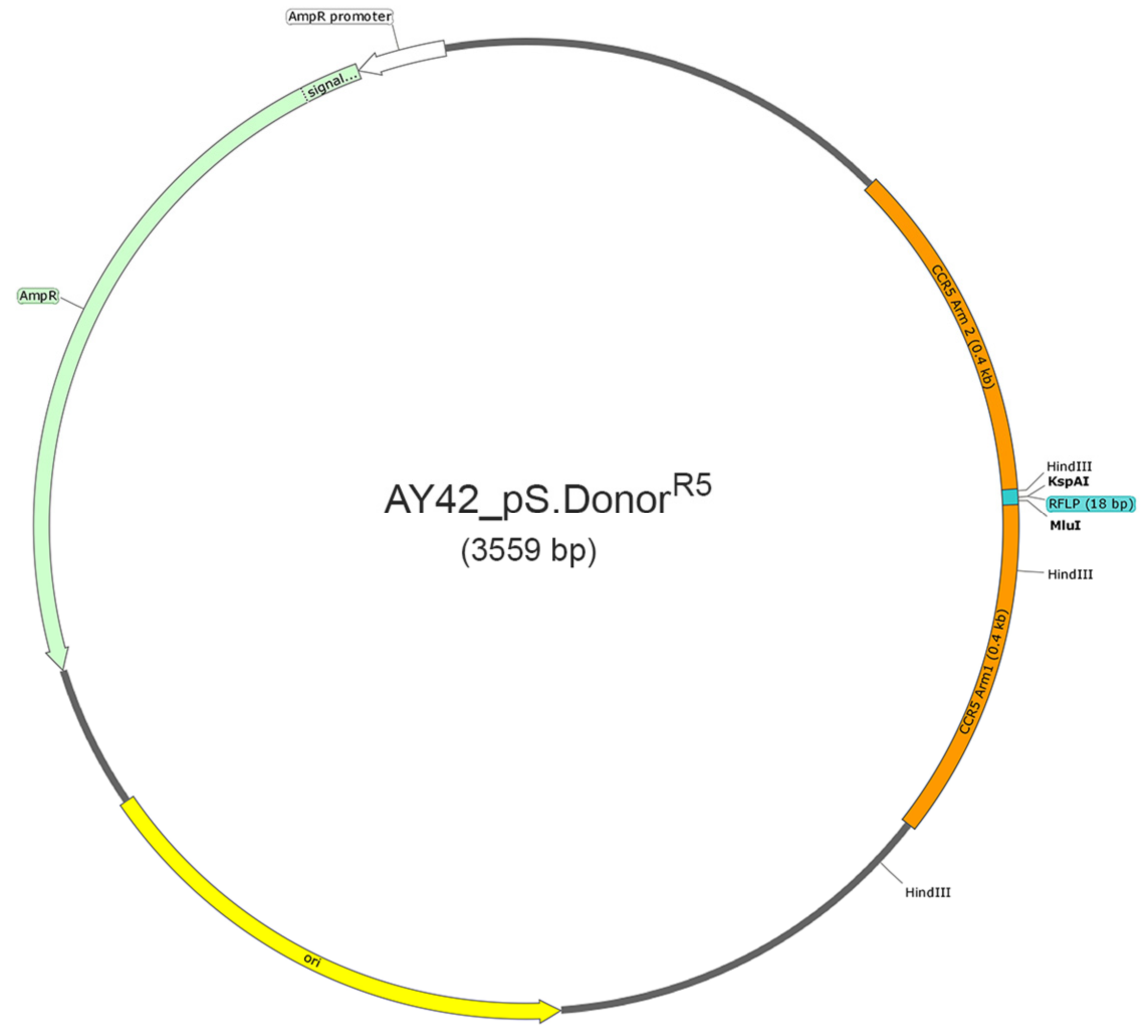


```
>AY42_pS.DonorR5  (3559 bp)
TCGCGCGTTTCGGTGATGACGGTGAAAACCTCTGACACATGCAGCTCCCGGAGACGGTCACAGCTTGTCTGTAAGCGGAT
GCCGGGAGCAGACAAGCCCGTCAGGGCGCGTCAGCGGGTGTTGGCGGGTGTCGGGGCTGGCTTAACTATGCGGCATCAGA
GCAGATTGTACTGAGAGTGCACCATATGCGGTGTGAAATACCGCACAGATGCGTAAGGAGAAAATACCGCATCAGGCGCC
ATTCGCCATTCAGGCTGCGCAACTGTTGGGAAGGGCGATCGGTGCGGGCCTCTTCGCTATTACGCCAGCTGGCGAAAGGG
GGATGTGCTGCAAGGCGATTAAGTTGGGTAACGCCAGGGTTTTCCCAGTCACGACGTTGTAAAACGACGGCCAGTGAATT
CGAGCTCGGTACCTCGCGAATGCATCTAGATGACGACCGCCGGAAACTTCAGCCTTTTGCAGTTTATCAGGATGAGGATG
ACCAGCATGTTGCCCACAAAACCAAAGATGAACACCAGTGAGTAGAGCGGAGGCAGGAGGCGGGCTGCGATTTGCTTCAC
ATTGATTTTTTGGCAGGGCTCCGATGTATAATAATTGATGTCATAGATTGGACTTGACACTTGATAATCCATCTTGTTCC
ACCCTGTGCATAAATAAAAAGTGATCTTTTATAAAGTCCTAGAATGTATTTAGTTGCCCTCCATGAATGCAAACTGTTTT
ATACATCAATAGGTTTTTAATTGCCTACATAGATGTCTACATTGAATTAACTCTCTTTTTGGCCAAGCAATGAAGTTTTG
TAGTGAAGGGAAGGTTTGCTGCTAGCTTCCCTGTCCACTAGATGGAGAAGCTTGTTAACACGCGTGCTCTGTTGGGGGAA
TTCATGAAAGCACCATCTCACCAAATAAAATCTTGTGCTCTATAGCACCATGGAGTGAATGAAGCTTTGACAACAATTTT
AAGAAACTGGGTCAAGCATGGTGCAGCTCAGCCTTTTTGTTGTTGTTGTTGTTGTGAGACAGGGTCTCACTCTGTCG
CCCAGGCTGGAGTGCAGTGGCACGATCATGGCTCACTGCAGCCTGGACCATCCCGGGCTCAAGCAATCCTCTCACCTCAC
CCCTCCAAGTGGCTGGGACTACAGGCGCCACCACACCAAGCTAATTTTGTTTATTTTTATAGATACAAGATCACACTAT
GTTGCCCAGGCTGGTCTCGAACTCCTGGGCTCAAGCGATCCTCCTGCCTCAGCCTCCTAGAATGCATTTGGCCGCGTCGT
ATCGGATCCCGGGCCCGTCGACTGCAGAGGCCTGCATGCAAGCTTGGCGTAATCATGGTCATAGCTGTTTCCTGTGTGAA
ATTGTTATCCGCTCACAATTCCACACAACATACGAGCCGGAAGCATAAAGTGTAAAGCCTGGGGTGCCTAATGAGTGAGC
TAACTCACATTAATTGCGTTGCGCTCACTGCCCGCTTTCCAGTCGGGAAACCTGTCGTGCCAGCTGCATTAATGAATCGG
CCAACGCGCGGGGAGAGGCGGTTTGCGTATTGGGCGCTCTTCCGCTTCCTCGCTCACTGACTCGCTGCGCTCGGTCGTTC
GGCTGCGGCGAGCGGTATCAGCTCACTCAAAGGCGGTAATACGGTTATCCACAGAATCAGGGGATAACGCAGGAAAGAAC
ATGTGAGCAAAAGGCCAGCAAAAGGCCAGGAACCGTAAAAAGGCCGCGTTGCTGGCGTTTTTCCATAGGCTCCGCCCCCC
TGACGAGCATCACAAAAATCGACGCTCAAGTCAGAGGTGGCGAAACCCGACAGGACTATAAAGATACCAGGCGTTTCCCC
CTGGAAGCTCCCTCGTGCGCTCTCCTGTTCCGACCCTGCCGCTTACCGGATACCTGTCCGCCTTTCTCCCTTCGGGAAGC
GTGGCGCTTTCTCATAGCTCACGCTGTAGGTATCTCAGTTCGGTGTAGGTCGTTCGCTCCAAGCTGGGCTGTGTGCACGA
```

```
ACCCCCCGTTCAGCCCGACCGCTGCGCCTTATCCGGTAACTATCGTCTTGAGTCCAACCCGGTAAGACACGACTTATCGC
CACTGGCAGCAGCCACTGGTAACAGGATTAGCAGAGCGAGGTATGTAGGCGGTGCTACAGAGTTCTTGAAGTGGTGGCCT
AACTACGGCTACACTAGAAGAACAGTATTTGGTATCTGCGCTCTGCTGAAGCCAGTTACCTTCGGAAAAAGAGTTGGTAG
CTCTTGATCCGGCAAACAAACCACCGCTGGTAGCGGTGGTTTTTTTGTTTGCAAGCAGCAGATTACGCGCAGAAAAAAAG
GATCTCAAGAAGATCCTTTGATCTTTTCTACGGGGTCTGACGCTCAGTGGAACGAAAACTCACGTTAAGGGATTTTGGTC
ATGAGATTATCAAAAAGGATCTTCACCTAGATCCTTTTAAATTAAAAATGAAGTTTTAAATCAATCTAAAGTATATATGA
GTAAACTTGGTCTGACAGTTACCAATGCTTAATCAGTGAGGCACCTATCTCAGCGATCTGTCTATTTCGTTCATCCATAG
TTGCCTGACTCCCCGTCGTGTAGATAACTACGATACGGGAGGGCTTACCATCTGGCCCCAGTGCTGCAATGATACCGCGA
GACCCACGCTCACCGGCTCCAGATTTATCAGCAATAAACCAGCCAGCCGGAAGGGCCGAGCGCAGAAGTGGTCCTGCAAC
TTTATCCGCCTCCATCCAGTCTATTAATTGTTGCCGGGAAGCTAGAGTAAGTAGTTCGCCAGTTAATAGTTTGCGCAACG
TTGTTGCCATTGCTACAGGCATCGTGGTGTCACGCTCGTCGTTTGGTATGGCTTCATTCAGCTCCGGTTCCCAACGATCA
AGGCGAGTTACATGATCCCCCATGTTGTGCAAAAAAGCGGTTAGCTCCTTCGGTCCTCCGATCGTTGTCAGAAGTAAGTT
GGCCGCAGTGTTATCACTCATGGTTATGGCAGCACTGCATAATTCTCTTACTGTCATGCCATCCGTAAGATGCTTTTCTG
TGACTGGTGAGTACTCAACCAAGTCATTCTGAGAATAGTGTATGCGGCGACCGAGTTGCTCTTGCCCGGCGTCAATACGG
GATAATACCGCGCCACATAGCAGAACTTTAAAAGTGCTCATCATTGGAAAACGTTCTTCGGGGCGAAAACTCTCAAGGAT
CTTACCGCTGTTGAGATCCAGTTCGATGTAACCCACTCGTGCACCCAACTGATCTTCAGCATCTTTTACTTTCACCAGCG
TTTCTGGGTGAGCAAAAACAGGAAGGCAAAATGCCGCAAAAAAGGGAATAAGGGCGACACGGAAATGTTGAATACTCATA
CTCTTCCTTTTTCAATATTATTGAAGCATTTATCAGGGTTATTGTCTCATGAGCGGATACATATTTGAATGTATTTAGAA
AAATAAACAAATAGGGGTTCCGCGCACATTTCCCCGAAAAGTGCCACCTGACGTCTAAGAAACCATTATTATCATGACAT
TAACCTATAAAAATAGGCGTATCACGAGGCCCTTTCGTC
```

Supplementary Figure 29. Map and nucleotide sequence of *CCR5*-targeting plasmid AY42_pS.Donor^{R5}. Orange regions, sequences homologous to the human *CCR5* "safe harbour" locus; RFLP, restriction fragment length polymorphism; AmpR, *β-lactamase* ampicillin resistance gene; high-copy number ColE1 origin of replication.

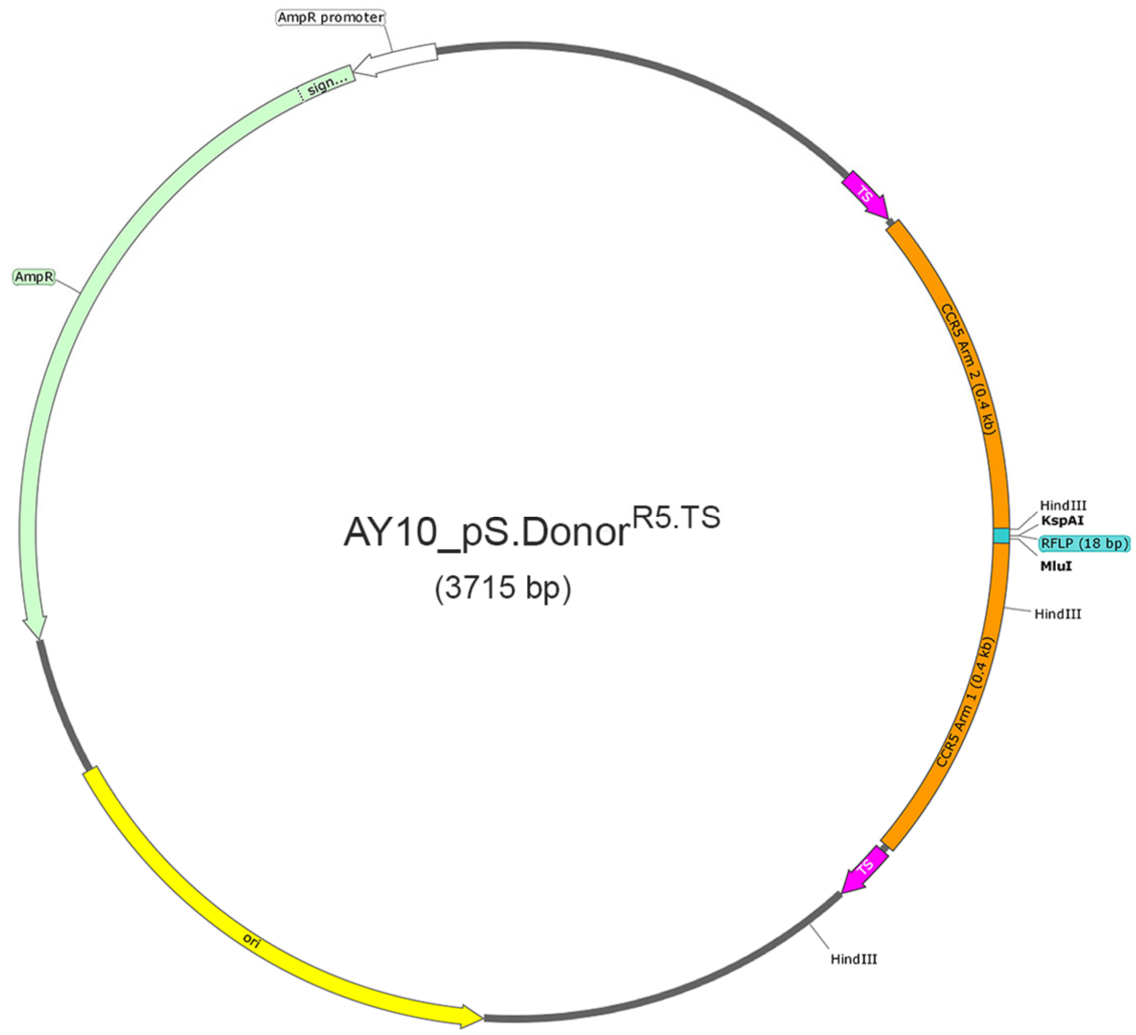

>AY10_pS.Donor.R5.TS (3715 bp)
TCGCGCGTTTCGGTGATGACGGTGAAAACCTCTGACACATGCAGCTCCCGGAGACGGTCACAGCTTGTCTGTAAGCGGAT
GCCGGGAGCAGACAAGCCCGTCAGGGCGCGTCAGCGGGTGTTGGCGGGTGTCGGGGCTGGCTTAACTATGCGGCATCAGA
GCAGATTGTACTGAGAGTGCACCATATGCGGTGTGAAATACCGCACAGATGCGTAAGGAGAAAATACCGCATCAGGCGCC
ATTCGCCATTCAGGCTGCGCAACTGTTGGGAAGGGCGATCGGTGCGGGCCTCTTCGCTATTACGCCAGCTGGCGAAAGGG
GGATGTGCTGCAAGGCGATTAAGTTGGGTAACGCCAGGGTTTTCCCAGTCACGACGTTGTAAAACGACGGCCAGTGAATT
CGAGCTCGGTACCTCGCGAATGCATCTAGATGACGACCGCCGGCGTTTGCTGCTAGCTTCCCTGTCCACTAGATGGAGAG
CTTGGCTCTGTTGGGGGAATTCATGAAAGCACCATCTGTTTAAACTTCAGCCTTTTGCAGTTTATCAGGATGAGGATGAC
CAGCATGTTGCCCACAAAACCAAAGATGAACACCAGTGAGTAGAGCGGAGGCAGGAGGCGGGCTGCGATTTGCTTCACAT
TGATTTTTTGGCAGGGCTCCGATGTATAATAATTGATGTCATAGATTGGACTTGACACTTGATAATCCATCTTGTTCCAC
CCTGTGCATAAATAAAAAGTGATCTTTTATAAAGTCCTAGAATGTATTTAGTTGCCCTCCATGAATGCAAACTGTTTTAT
ACATCAATAGGTTTTTAATTGCCTACATAGATGTCTACATTGAATTAACTCTCTTTTTGGCCAAGCAATGAAGTTTTGTA
GTGAAGGGAAGGTTTGCTGCTAGCTTCCCTGTCCACTAGATGGAGAAGCTTGTTAACACGCGTGCTCTGTTGGGGGAATT
CATGAAAGCACCATCTCACCAAATAAAATCTTGTGCTCTATAGCACCATGGAGTGAATGAAGCTTTGACAACAATTTTAA
GAAACTGGGTCAAGCATGGTGCAGCTCAGCCTTTTTGTTGTTGTTGTTGTTGTGAGACAGGGTCTCACTCTGTCGCC
CAGGCTGGAGTGCAGTGGCACGATCATGGCTCACTGCAGCCTGGACCATCCCGGGCTCAAGCAATCCTCTCACCTCACCC
CTCCAAGTGGCTGGGGACTACAGGCGCCACCACACCAAGCTAATTTTGTTTATTTTTATAGATACAAGATCACACTATGT
TGCCCAGGCTGGTCTCGAACTCCTGGGCTCAAGCGATCCTCCTGCCTCAGCCTCCTAGAATGCATTTAAATTTTGCTGCT
AGCTTCCCTGTCCACTAGATGGAGAGCTTGGCTCTGTTGGGGGAATTCATGAAAGCACCATCTGCGGCCGCGTCGTATCG
GATCCCGGGCCCGTCGACTGCAGAGGCCTGCATGCAAGCTTGGCGTAATCATGGTCATAGCTGTTTCCTGTGTGAAATTG
TTATCCGCTCACAATTCCACACAACATACGAGCCGGAAGCATAAAGTGTAAAGCCTGGGGTGCCTAATGAGTGAGCTAAC
TCACATTAATTGCGTTGCGCTCACTGCCCGCTTTCCAGTCGGGAAACCTGTCGTGCCAGCTGCATTAATGAATCGGCCAA
CGCGCGGGGAGAGGCGGTTTGCGTATTGGGCGCTCTTCCGCTTCCTCGCTCACTGACTCGCTGCGCTCGGTCGTTCGGCT
GCGGCGAGCGGTATCAGCTCACTCAAAGGCGGTAATACGGTTATCCACAGAATCAGGGGATAACGCAGGAAAGAACATGT
GAGCAAAAGGCCAGCAAAAGGCCAGGAACCGTAAAAAGGCCGCGTTGCTGGCGTTTTTCCATAGGCTCCGCCCCCCTGAC

```
GAGCATCACAAAAATCGACGCTCAAGTCAGAGGTGGCGAAACCCGACAGGACTATAAAGATACCAGGCGTTTCCCCCTGG
AAGCTCCCTCGTGCGCTCTCCTGTTCCGACCCTGCCGCTTACCGGATACCTGTCCGCCTTTCTCCCTTCGGGAAGCGTGG
CGCTTTCTCATAGCTCACGCTGTAGGTATCTCAGTTCGGTGTAGGTCGTTCGCTCCAAGCTGGGCTGTGTGCACGAACCC
CCCGTTCAGCCCGACCGCTGCGCCTTATCCGGTAACTATCGTCTTGAGTCCAACCCGGTAAGACACGACTTATCGCCACT
GGCAGCAGCCACTGGTAACAGGATTAGCAGAGCGAGGTATGTAGGCGGTGCTACAGAGTTCTTGAAGTGGTGGCCTAACT
ACGGCTACACTAGAAGAACAGTATTTGGTATCTGCGCTCTGCTGAAGCCAGTTACCTTCGGAAAAAGAGTTGGTAGCTCT
TGATCCGGCAAACAAACCACCGCTGGTAGCGGTGGTTTTTTTGTTTGCAAGCAGCAGATTACGCGCAGAAAAAAAGGATC
TCAAGAAGATCCTTTGATCTTTTCTACGGGGTCTGACGCTCAGTGGAACGAAAACTCACGTTAAGGGATTTTGGTCATGA
GATTATCAAAAAGGATCTTCACCTAGATCCTTTTAAATTAAAAATGAAGTTTTAAATCAATCTAAAGTATATATGAGTAA
ACTTGGTCTGACAGTTACCAATGCTTAATCAGTGAGGCACCTATCTCAGCGATCTGTCTATTTCGTTCATCCATAGTTGC
CTGACTCCCCGTCGTGTAGATAACTACGATACGGGAGGGCTTACCATCTGGCCCCAGTGCTGCAATGATACCGCGAGACC
CACGCTCACCGGCTCCAGATTTATCAGCAATAAACCAGCCAGCCGGAAGGGCCGAGCGCAGAAGTGGTCCTGCAACTTTA
TCCGCCTCCATCCAGTCTATTAATTGTTGCCGGGAAGCTAGAGTAAGTAGTTCGCCAGTTAATAGTTTGCGCAACGTTGT
TGCCATTGCTACAGGCATCGTGGTGTCACGCTCGTCGTTTGGTATGGCTTCATTCAGCTCCGGTTCCCAACGATCAAGGC
GAGTTACATGATCCCCCATGTTGTGCAAAAAAGCGGTTAGCTCCTTCGGTCCTCCGATCGTTGTCAGAAGTAAGTTGGCC
GCAGTGTTATCACTCATGGTTATGGCAGCACTGCATAATTCTCTTACTGTCATGCCATCCGTAAGATGCTTTTCTGTGAC
TGGTGAGTACTCAACCAAGTCATTCTGAGAATAGTGTATGCGGCGACCGAGTTGCTCTTGCCCGGCGTCAATACGGGATA
ATACCGCGCCACATAGCAGAACTTTAAAAGTGCTCATCATTGGAAAACGTTCTTCGGGGCGAAAACTCTCAAGGATCTTA
CCGCTGTTGAGATCCAGTTCGATGTAACCCACTCGTGCACCCAACTGATCTTCAGCATCTTTTACTTTCACCAGCGTTTC
TGGGTGAGCAAAAACAGGAAGGCAAAATGCCGCAAAAAAGGGAATAAGGGCGACACGGAAATGTTGAATACTCATACTCT
TCCTTTTTCAATATTATTGAAGCATTTATCAGGGTTATTGTCTCATGAGCGGATACATATTTGAATGTATTTAGAAAAAT
AAACAAATAGGGGTTCCGCGCACATTTCCCCGAAAAGTGCCACCTGACGTCTAAGAAACCATTATTATCATGACATTAAC
CTATAAAAATAGGCGTATCACGAGGCCCTTTCGTC
```

Supplementary Figure 30. Map and nucleotide sequence of *CCR5*-targeting plasmid AY10_pS.Donor$^{R5.TS}$. Orange regions, sequences homologous to the human *AAVS1* "safe harbour" locus; RFLP, restriction fragment length polymorphism; Magenta arrows, gRNA$^{R5.1}$ target site (TS); AmpR, *β-lactamase* ampicillin resistance gene; high-copy number ColE1 origin of replication.

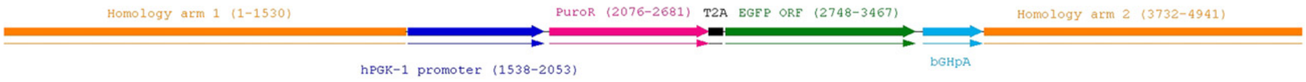

SEQUENCE

TCGACCCCGCCGCGCCGCCGCCCGCCCGCGCCGTGCTGGACTCCACCAACGCCGACGGTATCAGCGCCCTGCACCAGGTCAGC
GCCCCCCGCCCGGCGTCTCCCGGGGCCAGGTCCACCCTCTGCTGCGCCACCTGGGGCATCCTCCTTCCCCGTTGCCAGTCTCG
ATCCGCCCCGTCGTTCCTGGCCCTGGGCTTTGCCACCCTATGCTGACACCCCGTCCCAGTCCCCCTTACCATTCCCCTTCGAC
CACCCCACTTCCGAATTGGAGCCGCTTCAACTGGCCCTGGGCTTAGCCACTCTGTGCTGACCACTCTGCCCCAGGCCTCCTTA
CCATTCCCCTTCGACCTACTCTCTTCCGCATTGGAGTCGCTTTAACTGGCCCTGGCTTTGGCAGCCTGTGCTGACCCATGCAG
TCCTCCTTACCATCCCTCCCTCGACTTCCCCTCTTCCGATGTTGAGCCCCTCCAGCCGGTCCTGGACTTTGTCTCCTTCCCTG
CCCTGCCCTCTCCTGAACCTGAGCCAGCTCCCATAGCTCAGTCTGGTCTATCTGCCTGGCCCTGGCCATTGTCACTTTGCGCT
GCCCTCCTCTCGCCCCCGAGTGCCCTTGCTGTGCCGCCGGAACTCTGCCCTCTAACGCTGCCGTCTCTCTCCTGAGTCCGGAC
CACTTTGAGCTCTACTGGCTTCTGCGCCGCCTCTGGCCCACTGTTTCCCCTTCCCAGGCAGGTCCTGCTTTCTCTGACCTGCA
TTCTCTCCCCTGGGCCTGTGCCGCTTTCTGTCTGCAGCTTGTGGCCTGGGTCACCTCTACGGCTGGCCCAGATCCTTCCCTGC
CGCCTCCTTCAGGTTCCGTCTTCCTCCACTCCCTCTTCCCCTTGCTCTCTGCTGTGTTGCTGCCCAAGGATGCTCTTTCCGGA
GCACTTCCTTCTCGGCGCTGCACCACGTGATGTCCTCTGAGCGGATCCTCCCCGTGTCTGGGTCCTCTCCGGGCATCTCTCCT
CCCTCACCCAACCCCATGCCGTCTTCACTCGCTGGGTTCCCTTTTCCTTCTCCTTCTGGGGCCTGTGCCATCTCTCGTTTCTT
AGGATGGCCTTCTCCGACGGATGTCTCCCTTGCGTCCCGCCTCCCCTTCTTGTAGGCCTGCATCATCACCGTTTTTCTGGACA
ACCCCAAAGTACCCCGTCTCCCTGGCTTTAGCCACCTCTCCATCCTCTTGCTTTCTTTGCCTGGACACCCCGTTCTCCTGTGG
ATTCGGGTCACCTCTCACTCCTTTCATTTGGGCAGCTCCCCTACCCCCCTTACCTCTCTAGTCTGTGCTAGCTCTTCCAGCCC
CCTGTCATGGCATCTTCCAGGGGTCCGAGAGCTCAGCTAGTCTTCTTCCTCCAACCCGGGCCCCTATGTCCACTTCAGGACAG
CATGTTTGCTGCCTCCAGGGATCCTGTGTCCCCGAGCTGGGACCACCTTATATTCCCAGGGCCGGTTAATGTGGCTCTGGTTC
TGGGTACTTTTATCTGTCCCCTCCACCCCACAGTGGGGCAAGCTTCCACGGGGTTGGGGTTGCGCCTTTTCCAAGGCAGCCCT
GGGTTTGCGCAGGGACGCGGCTGCTCTGGGCGTGGTTCCGGGAAACGCAGCGGCGCCGACCCTGGGTCTCGCACATTCTTCAC
GTCCGTTCGCAGCGTCACCCGGATCTTCGCCGCTACCCTTGTGGGCCCCCCGGCGACGCTTCCTGCTCCGCCCCTAAGTCGGG
AAGGTTCCTTGCGGTTCGCGGCGTGCCGGACGTGACAAACGGAAGCCGCACGTCTCACTAGTACCCTCGCAGACGGACAGCGC
CAGGGAGCAATGGCAGCGCGCCGACCGCGATGGGCTGTGGCCAATAGCGGCTGCTCAGCAGGGCGCGCCGAGAGCAGCGGCCG
GGAAGGGGCGGTGCGGGAGGCGGGGTGTGGGGCGGTAGTGTGGGCCCTGTTCCTGCCCGCGCGGTGTTCCGCATTCTGCAAGC
CTCCGGAGCGCACGTCGGCAGTCGGCTCCCTCGTTGACCGAATCACCGACCTCTCTCCCCACCGGGTACGTCGCTAGCCCACC
ATGACCGAGTACAAGCCCACGGTGCGCCTCGCCACCCGCGACGACGTCCCCCGGGCCGTACGCACCCTCGCCGCCGCGTTCGC
CGACTACCCCGCCACGCGCCACACCGTCGACCCGGACCGCCACATCGAGCGGGTCACCGAGCTGCAAGAACTCTTCCTCACGC
GCGTCGGGCTCGACATCGGCAAGGTGTGGGTCGCGGACGACGGCGCCGCGGTGGCGGTCTGGACCACGCCGGAGAGCGTCGAA
GCGGGGGCGGTGTTCGCCGAGATCGGCCCGCGCATGGCCGAGTTGAGCGGTTCCCGGCTGGCCGCGCAGCAACAGATGGAAGG
CCTCCTGGCGCCGCACCGGCCCAAGGAGCCCGCGTGGTTCCTGGCCACCGTCGGCGTCTCGCCCGACCACCAGGGCAAGGGTC
TGGGCAGCGCCGTCGTGCTCCCCGGAGTGGAGGCGGCCGAGCGCGCCGGGGTGCCCGCCTTCCTGGAGACCTCCGCGCCCCGC
AACCTCCCCTTCTACGAGCGGCTCGGCTTCACCGTCACCGCCGACGTCGAGGTGCCCGAAGGACCGCGCACCTGGTGCATGAC
CCGCAAGCCCGGTGCCGGATCGGGAGAGGGCAGAGGAAGTCTGCTAACATGCGGTGACGTCGAGGAGAATCCTGGCCCACCGG
TCGCCACCATGGTGAGCAAGGGCGAGGAGCTGTTCACCGGGGTGGTGCCCATCCTGGTCGAGCTGGACGGCGACGTAAACGGC
CACAAGTTCAGCGTGTCCGGCGAGGGCGAGGGCGATGCCACCTACGGCAAGCTGACCCTGAAGTTCATCTGCACCACCGGCAA
GCTGCCCGTGCCCTGGCCCACCCTCGTGACCACCCTGACCTACGGCGTGCAGTGCTTCAGCCGCTACCCCGACCACATGAAGC
AGCACGACTTCTTCAAGTCCGCCATGCCCGAAGGCTACGTCCAGGAGCGCACCATCTTCTTCAAGGACGACGGCAACTACAAG
ACCCGCGCCGAGGTGAAGTTCGAGGGCGACACCCTGGTGAACCGCATCGAGCTGAAGGGCATCGACTTCAAGGAGGACGGCAA
CATCCTGGGGCACAAGCTGGAGTACAACTACAACAGCCACAACGTCTATATCATGGCCGACAAGCAGAAGAACGGCATCAAGG
TGAACTTCAAGATCCGCCACAACATCGAGGACGGCAGCGTGCAGCTCGCCGACCACTACCAGCAGAACACCCCCATCGGCGAC
GGCCCCGTGCTGCTGCCCGACAACCACTACCTGAGCACCCAGTCCGCCCTGAGCAAAGACCCCAACGAGAAGCGCGATCACAT
GGTCCTGCTGGAGTTCGTGACCGCCGCCGGGATCACTCTCGGCATGGACGAGCTGTACAAGTAAAGCGGCCGCGTCGAGTCTA
GGATCAGCCTCGACTGTGCCTTCTAGTTGCCAGCCATCTGTTGTTTGCCCCTCCCCCGTGCCTTCCTTGACCCTGGAAGGTGC
CACTCCCACTGTCCTTTCCTAATAAAATGAGGAAATTGCATCGCATTGTCTGAGTAGGTGTCATTCTATTCTGGGGGGTGGGG
TGGGGCAGGACAGCAAGGGGGAGGATTGGGAAGACAATAGCAGGCATGCTGGGGATGCGGTGGGCTCTATGGAAGCTTTACTA
GGGACAGGATTGGTGACAGAAAAGCCCCATCCTTAGGCCTCCTCCTTCCTAGTCTCCTGATATTGGGTCTAACCCCCACCTCC
TGTTAGGCAGATTCCTTATCTGGTGACACACCCCCATTTCCTGGAGCCATCTCTCTCCTTGCCAGAACCTCTAAGGTTTGCTT
ACGATGGAGCCAGAGAGGATCCTGGGAGGGAGAGCTTGGCAGGGGGTGGGAGGGAAGGGGGGGATGCGTGACCTGCCCGGTTC
TCAGTGGCCACCCTGCGCTACCCTCTCCCAGAACCTGAGCTGCTCTGACGCGGCTGTCTGGTGCGTTTCACTGATCCTGGTGC
TGCAGCTTCCTTACACTTCCCAAGAGGAGAAGCAGTTTGGAAAAACAAAATCAGAATAAGTTGGTCCTGAGTTCTAACTTTGG

CTCTTCACCTTTCTAGTCCCCAATTTATATTGTTCCTCCGTGCGTCAGTTTTACCTGTGAGATAAGGCCAGTAGCCAGCCCCG
TCCTGGCAGGGCTGTGGTGAGGAGGGGGGTGTCCGTGTGGAAAACTCCCTTTGTGAGAATGGTGCGTCCTAGGTGTTCACCAG
GTCGTGGCCGCCTCTACTCCCTTTCTCTTTCTCCATCCTTCTTTCCTTAAAGAGTCCCCAGTGCTATCTGGGACATATTCCTC
CGCCCAGAGCAGGGTCCCGCTTCCCTAAGGCCCTGCTCTGGGCTTCTGGGTTTGAGTCCTTGGCAAGCCCAGGAGAGGCGCTC
AGGCTTCCCTGTCCCCCTTCCTCGTCCACCATCTCATGCCCCTGGCTCTCCTGCCCCTTCCCTACAGGGGTTCCTGGCTCTGC
TCTTCAGACTGAGCCCCGTTCCCCTGCATCCCCGTTCCCCTGCATCCCCCTTCCCCTGCATCCCCCAGAGGCCCCAGGCCACC
TACTTGGCCTGGACCCCACGAGAGGCCACCCCAGCCCTGTCTACCAGGCTGCCTTTTGGGTGGATTCTCCTCCAACTGTGGGG
TGACTGCTTGGCAAACTCACTCTTCGGGGTATCCCAGGAGGCCTGGAGCATTGGGGTGGGCTGGGGTTCAGAGAGGAGGGATT
CCCTTCTCAGGTTACGTGGCCAAGAAGCAGGGGAGCTGGGTTTGGGTCAGGTCTGGGTGTGGGGTGACCAGCTTATGCTGTTT
GCCCAGGACAGCCTAGTTTTAGCACTGAAACCCTCAGTCCTAGG

Supplementary Figure 31. Map and nucleotide sequence of the targeting module in the Puro^{R}.T2A.EGFP-encoding plasmids pDonor.EP^{S1} and pDonor.EP$^{S1.TS}$. Construct pDonor.EP$^{S1.TS}$ differs from pDonor.EP^{S1} in that it has its *AAVS1*-targeting module bracketed by gRNA^{S1} target sites. The regions of homology in pDonor.EP$^{S1.TS}$ and pDonor.EP^{S1} are identical to those present in their EGFP-encoding counterparts. hPGK-1 promoter, human phosphoglycerate kinase 1 gene (*PGK1*) regulatory sequences; PuroR, *Streptomyces* puromycin N-acetyl-transferase (*pac*) gene; T2A, *Thoseaasigna* virus 2A “self-cleaving” peptide coding sequence; EGFP, enhanced green fluorescence protein gene (*EGFP*) open reading frame; bGHpA, bovine growth hormone gene (*GH1*) polyadenylation signal; DNA sequences sharing identity to the human *AAVS1* “safe harbour” locus are indicated in orange.

Supplementary Table 1. Oligonucleotide pairs to generate the gRNA expression constructs used in this study.

Plasmids	Oligonucleotide pairs
AL8_pgRNA^{DMD}	5′-ACCGTTACATACAGGCTAGGGAG-3′<br>5′-AAACCTGCCTAGCCTGTATGTAA-3′
AD19_pgRNA$^{S1.2}$	5′-ACCGTCCCTAGTGGCCCCACTGT-3′<br>5′-AAACACAGTGGGGCCACTAGGGA-3′
AD13_pgRNA$^{S1.3}$	5′-ACCGTCACCAATCCTGTCCCTAG-3′<br>5′-AAACCTAGGGACAGGATTGGTGA-3′
AY22_pgRNA$^{R5.1}$	5′-ACCGGAGAGCTTGGCTCTGTTGG-3′<br>5′-AAACCCAACAGAGCCAAGCTCTC-3′
AY23_pgRNA$^{R5.2}$	5′-ACCGAGCCAAGCTCTCCATCTAG-3′<br>5′-AAACCTAGATGGAGAGCTTGGCT-3′
L06_pgRNA$^{OUT.1}$	5′-ACCGCCCCGTCGTTCCTGGCCCT-3′<br>5′-AAACAGGGCCAGGAACGACGGGG-3′
AA44_pgRNA$^{OUT.2}$	5′-ACCGATTCCCTTCTCAGGTTACG-3′<br>5′-AAACCGTAACCTGAGAAGGGAAT-3′
X32_pgRNA$^{IN.1}$	5′-ACCGTTAATGTGGCTCTGGTTCT-3′<br>5′-AAACAGAACCAGAGCCACATTAA-3′
AA48_pgRNA$^{IN.2}$	5′-ACCGTTAGGCAGATTCCTTATC-3′<br>5′-AAACGATAAGGAATCTGCCTAA-3′

Supplementary Table 2. Transfection scheme for the experiments presented in **Supplementary Fig. 1a** assessing disruption of allelic *AAVS1* target sequences by Cas9 versus Cas9^{D10A}.

HEK 293T cells	24-well plate 1.0×10^{5} cells/well (1.65 µl PEI/well, medium replaced at 24 h p.t.)					DNA break type
	pCas9	pCas9^{D10A}	pgRNA^{GFP1}	pgRNA^{S1}	pcDNA3.1	
Size (bp)	9553	9553	3974	3973	5428	
DNA (ng/well)	250			250		DSB
		250		250		Nick
	250		250			Control
		250	250			Control
				250	250	Control

Post-transfection (p.t.)

Supplementary Table 3. Transfection scheme for the experiments shown in **Supplementary Fig. 1c** assessing target gene mutagenesis by Cas9 versus Cas9^{D10A}.

H27 cells	24-well plate 0.8×10^{5} cells/well (1.65 µl PEI/well, medium replaced at 24 h p.t.)				DNA break type
	pCas9	pCas9^{D10A}	pgRNA^{GFP2}	pgRNA^{NT}	
Size (bp)	9553	9553	3974	3045	
DNA (ng/well)	364		116		DSB
		364	116		Nick
	364			116	Control
		364		116	Control

Supplementary Table 4. Transfection scheme for the experiments presented in **Supplementary Figs. 1d** and **1e** probing for *DMD-AAVS1* translocations.

HeLa cells	24-well plate 0.6×10^{5} cells/well (1.65 µl PEI/well, medium replaced at 24 h p.t.)					DNA break type
	pCas9	pCas9^{D10A}	pgRNA^{S1}	pgRNA^{DMD}	pcDNA3.1	
Size (bp)	9553	9553	3973	3046	5428	
DNA (ng/well)	180		160	160		DSB
		180	160	160		Nick
	180					Control

Supplementary Table 5. Transfection scheme for the *DMD*-targeting experiments shown in **Figs. 1b** and **1c**.

HeLa cells	24-well plate 0.6×10^5 cells/well (2.19 µl PEI/well, medium replaced at 6 h p.t.)						Gene targeting strategy
	pCas9	pCas9^{D10A}	pgRNA^{Empty}	pgRNA^{DMD}	pDonor^{DMD}	pDonor$^{DMD.TS}$	
Size (bp)	9553	9553	3915	3046	8610	8667	
DNA (ng/well)	150			150	200		Standard
	150			150		200	DSB^2
		150		150	200		Nick
		150		150		200	Nick^2
	150		150		200		Control

Supplementary Table 6. Transfection scheme for the *DMD*-targeting experiments shown in **Supplementary Fig. 2**.

HeLA cells	24-well plate 0.4×10^5 cells/well (2.19 µl PEI/well, medium replaced at 6 h p.t.)								
	pCAG.Cas9	pCAG.Cas9^{D10A}	pgRNA^{DMD}	pgRNA^{Empty}	pDonor^{DMD}	pDonor^{DMD1TS}	pDonor^{DMD2TS}	pDMD$^{DonorIV.TS}$	Gene targeting strategy
Size (bp)	10636	10636	3047	3915	8610	8667	8724	8724	
DNA (ng/well)	286,26		82,01		231,73				Standard
		286,26	82,01			231,73			Nick^2
		286,26	82,01				231,73		Nick^2
		286,26	82,01					231,73	Nick^2
		286,26	82,01		231,73				Nick
	286,26			82,01	231,73				Ctrl
	286,26			82,01		231,73			Ctrl

Supplementary Table 7. Transfection scheme for the *AAVS1*-targeting experiments in 293T cells presented in **Fig. 2**.

HEK293T cells	24-well plate1.0×10^5 cells/well (1.65 µl PEI/well, medium replaced at 24 h p.t.)						Gene targeting strategy
	pCas9	pCas9^{D10A}	pgRNA^{Empty}	pgRNA^{S1}	pDonorE^{S1}	pDonorE$^{S1.TS}$	
Size (bp)	9553	9553	3915	3973	11375	11465	
DNA (ng/well)	150			150	200		Standard
	150			150		200	DSB^2
		150		150	200		Nick
		150		150		200	Nick^2
	150		150		200		Control
	150		150			200	Control
		150	150		200		Control
		150	150			200	Control

Supplementary Table 8. Transfection scheme for the *AAVS1*-targeting experiments in HeLa cells shown in **Fig. 2**.

HeLa cells	24-well plate0.6×10^5 cells/well (1.32 µl PEI/well, medium replaced at 24 h p.t.)						Gene targeting strategy
	pCas9	pCas9^{D10A}	pgRNA^{Empy}	pgRNA^{S1}	pDonorE^{S1}	pDonorE$^{S1.TS}$	
Size (bp)	9553	9553	3915	3973	11375	11465	
DNA (ng/well)	120			120	200		Standard
	120			120		200	DSB^2
		120		120	200		Nick
		120		120		200	Nick^2
	120		120		160		Control
	120		120			160	Control
		120	120		160		Control
		120	120			160	Control

Supplementary Table 9. Transfection scheme for the experiments shown in **Supplementary Fig. 4** comparing donors with no, one or two gRNA^{S1} target sites.

HeLa Cells	24-well plate0.6×10^5 cells/well (2.19 µl PEI/well, medium replaced at 6 h p.t.)							Gene targeting strategy
	pCas9	pCas9^{D10A}	pgRNA^{Empy}	pgRNA^{S1}	pDonor^{S1}	pDonor$^{S1.1×TS}$	pDonor$^{S1.TS}$	
Size (bp)	9553	9553	3915	3973	7199	7251	7359	
DNA (ng/well)	150			150	200			Standard
		150		150	200			Nick (0×TS)
		150		150		200		Nick^2 (1×TS)
		150		150			200	Nick^2 (2×TS)
	150		150		200			Control

Supplementary Table 10. Transfection scheme for the experiments presented in **Supplementary Fig. 5a** testing different *AAVS1*-targeting reagents.

HeLa Cells	24-well plate 0.6×10^5 cells/well (2.19 µl PEI/well, medium replaced at 6 h p.t.)							
	pCas9	pCas9^{D10A}	pgRNA^{Empy}	pgRNA^{S1}	pgRNA$^{S1.2}$	pDonor^{S1}	pDonor$^{S1.1×TS}$	Gene targeting strategy
Size(bp)	9553	9553	3915	3973	3046	5966	6078	
DNA (ng/well)	150			150		200	200	Standard
		150		150				Nick^2
	150				150		200	Standard
		150			150			Nick^2
	150		150			200		Control

Supplementary Table 11. Transfection scheme for the experiments shown in **Supplementary Fig. 5b** testing different *AAVS1*-targeting reagents.

HeLa Cells	24-well plate 0.6×10^5 cells/well (2.19 µl PEI/well, medium replaced at 6 h p.t.)								
	pCas9	pCas9^{D10A}	pgRNA^{Empy}	pgRNA^{S1}	pDonor^{S1}	pDonor$^{S1.1\times TS}$	pDonor.37^{S1}	pDonor.37$^{S1.1\times TS}$	
Size(bp)	9553	9553	3915	3973	7199	7251	5966	6078	
DNA (ng/well)	150			150	200				Standard
		150		150	200				Nick
		150		150		200			Nick^2
	150			150			200		Standard
		150		150			200		Nick
		150		150				200	Nick^2
	150		150		200				Control

Supplementary Table 12. Transfection scheme for the experiments shown in **Fig. 5c** assessing *AAVS1*-targeting induced by Cas9^{D10A} versus Cas9^{H840A}.

HeLA cells	24-well plate 0.4×10^5 cells/well (2.19 µl PEI/well, medium replaced at 6 h p.t.)							
	pCas9	pCas9^{D10A}	pCas9^{H840A}	pgRNA^{Empy}	pgRNA^{S1}	pDonorE^{S1}	pDonorE$^{S1.TS}$	Gene targeting strategy
Size (bp)	9553	9553	9553	3915	3973	11375	11465	
DNA (ng/well)	150				150	200		Standard
	150				150		200	DSB^2
		150			150	200		Nick
		150			150		200	Nick^2
			150		150	200		Nick
			150		150		200	Nick^2
	150			150		200		Control

Supplementary Table 13. Transfection scheme for the *AAVS1* editing experiments presented in **Fig. 2e**.

HeLA cells	24-well plate 0.4×10^5 cells/well (2.19 µl PEI/well, medium replaced at 6 h p.t.)					
	pCAG.Cas9	pCAG.Cas9^{D10A}	pgRNA^{S1}	pS.Donor^{S1}	pS.Donor$^{S1.TS}$	Gene targeting strategy
Size (bp)	10636	10636	3973	3333	3526	
DNA (ng/well)	371		106	123		Standard
		371	106		123	Nick^2
		371	106	123		Nick
	371		106		123	DSB^2

Supplementary Table 14. Transfection scheme for the experiments shown in **Supplementary Fig. 9** comparing DSB- versus SSB-dependent gene targeting at *AAVS1*.

HeLA cells	24-well plate<br>0.4×10^5 cells/well (2.19 µl PEI/well, medium replaced at 6 h p.t.)								
	pCAG.Cas9	pCAG.Cas9^{D10A}	pgRNA^{S1}	pgRNA$^{S1.2}$	pgRNA$^{S1.3}$	pDonor^{S1}	pDonor$^{S1.TS}$	pgRNA^{Empy}	Gene targeting strategy
Size (bp)	10636	10636	3973	3046	3046	7199	7359	3915	
DNA (ng/well)	306		114			207			Standard
	306		114				207		DSB[2]
	306		57	57		207			DSB
	306		57		57	207			DSB
		306	114			207			Nick
		306	114				207		Nick[2]
	306					207		114	Ctrl
	306						207	114	Ctrl

Supplementary Table 15. Transfection scheme for the donor competition experiments shown in **Fig. 4**.

HeLa Cells	24-well plate<br>0.8×10^5 cells/well (2.63 µl PEI/well, medium replaced at 6 h p.t.)					
	pCas9^{D10A}	pgRNA^{S1}	pDonor^{S1}	pDonor$^{S1.TS}$	pDonor.Turq^{S1}	pDonor.Turq$^{S1.TS}$
Size(bp)	9553	3973	7199	7359	7203	7359
DNA (ng/well)	164	68	124			124
	164	68		124	124	

Supplementary Table 16. Transfection scheme for the multiplexing gene targeting experiments presented in **Fig. 5**.

HeLa Cells	24-well plate<br>0.6×10^5 cells/well (2.19 µl PEI/well, medium replaced at 6 h p.t.)								Gene targeting strategy
	pCas9	pCas9^{D10A}	gRNA^{S1}	pDonor^{S1}	pDonor$^{S1.TS}$	pDonor.Turq^{S1}	pDonor.Turq$^{S1.TS}$	gRNA^{Empy}	pLacZ
Size (bp)	9553	9553	3973	7199	7359	7203	7359	3915		
DNA (ng/well)	164		68	124		124				Standard
		164	68		124		124			Nick[2]
	164		68	124					124	Standard
		164	68		124				124	Nick[2]
	164		68			124			124	Standard
		164	68				124		124	Nick[2]
	164			124				68	124	Ctrl
	164					124		68	124	Ctrl

Supplementary Table 17. Transfection scheme for the *CCR5* editing experiments presented in **Fig. 6c**.

HeLA cells	24-well plate<br>0.4×10^5 cells/well (2.19 µl PEI/well, medium replaced at 6 h p.t.)					
	pCAG.Cas9	pCAG.Cas9^{D10A}	pgRNA$^{R5.1}$	pDonor^{R5}	pDonor$^{R5.TS}$	Gene targeting strategy
Size (bp)	10636	10636	3046	3559	3715	
DNA (ng/well)	370		106	124		Standard
		370	106		124	Nick[2]
		370	106	124		Nick
	370		106		124	DSB[2]

Supplementary Table 18. Transfection scheme for the *CCR5* editing experiments presented in **Fig. 6d** (upper panel).

HeLA cells	24-well plate<br>0.4×10^5 cells/well (2.19 µl PEI/well, medium replaced at 6 h p.t.)							
	pCAG.Cas9	pCAG.Cas9^{D10A}	pgRNA$^{R5.1}$	pgRNA$^{R5.2}$	pDonor^{R5}	pDonor$^{R5.TS}$	pgRNA^{Empy}	Gene targeting strategy
Size (bp)	10636	10636	3046	3046	3559	3715	3915	
DNA (ng/well)	371		106		123			Standard
		371	106			123		Nick[2]
		371	106		123			Nick
	371		106			123		DSB[2]
	371		53	53	123			DSB
	371				123		106	Ctrl
	371					123	106	Ctrl

Supplementary Table 19. Transfection scheme for the *CCR5* mutagenesis experiments presented in **Fig. 6d** (lower panel).

HeLA cells	24-well plate<br>0.4×10^5 cells/well (2.19 µl PEI/well, medium replaced at 6 h p.t.)					
	pCAG.Cas9	pCAG.Cas9^{D10A}	pgRNA$^{R5.1}$	pgRNA$^{R5.2}$	pgRNA^{Empy}	pcDNA3.1	Gene targeting strategy
Size (bp)	10636		3046	3046	3915	5428	
DNA (ng/well)	371		106			123	DSB
	371			106		123	DSB
		371	106			123	Nick
		371	53	53		123	DSB
	371				106	123	Ctrl

Supplementary Table 20. Transfection scheme for the experiment investigating RGN-induced gene targeting by tandem paired nicking presented in **Supplementary Fig. 6**.

HeLA cells	24-well plate 0.6×10^5 cells/well (2.19 µl PEI/well, medium replaced at 6 h p.t.)										Gene targeting strategy
	pCas9	pCas9^{D10A}	pgRNA^{S1}	pgRNA$_{OUT.1}$	pgRNA$^{OUT.2}$	pgRNA$^{IN.1}$	pgRNA$^{IN.2}$	pDonorE^{S1}	pDonorE$^{S1.TS}$	pgRNA^{empty}	
Size (bp)	9553	9553	3973	3046	3046	3046	3045	11375	11465	3915	
DNA (ng/well)	150		75					200		75	Standard
		150	75						200	75	Nick^2
		150	75					200		75	Nick^2
		150		75	75			200			Tandem Nick^2
		150				75	75	200			Tandem Nick^2
	150							200		150	Control

Supplementary Table 21. Transfection scheme for the gene targeting experiments in human PSCs presented in **Fig. 3**.

LUMC0044iCtrl44.9 iPSCs (Condition A)	1.1×10^{6} cells per well (6-well plate) 5 µl Lipofectamine^{TM} 2000 per experimental condition						
	pCAG.Cas9	pCAG.Cas9^{D10A}	gRNA^{Empty}	gRNA^{S1}	pDonor^{S1}	pDonor$^{S1.TS}$	Gene targeting strategy
Size (bp)	10636	10636	3915	3973	7199	7359	
DNA (ng/well)	480			480	640		Standard
		480		480		640	Nick^{2}
		480	480		640		Nick
					640		Control

LUMC0044iCtrl44.9 iPSCs (Condition B)	0.5×10^{6} cells per well (6-well plate) 10 µl Lipofectamine^{TM} 2000 per experimental condition						
	pCAG.Cas9	pCAG.Cas9^{D10A}	gRNA^{Empty}	gRNA^{S1}	pDonor^{S1}	pDonor$^{S1.TS}$	Gene targeting strategy
Size (bp)	10636	10636	3915	3973	7199	7359	
DNA (ng/well)	1200			1200	1600		Standard
		1200		1200		1600	Nick^{2}
		1200	1200		1600		Nick
					1600		Control

H1 ESCs (Condition C)	0.5×10^{6} cells per well (6-well plate) 5 µl Lipofectamine^{TM} 2000 per experimental condition					
	pCas9.gRNA^{S1}	pCas9^{D10A}.gRNA^{S1}	pCas9.gRNA^{NT}	pDonor.EP^{S1}	pDonor.EP$^{S1.TS}$	Gene targeting strategy
Size (bp)	9450	9450	9447	7881	8041	
DNA(ng/well)	1000			1000		Standard
		1000			1000	Nick^{2}
		1000		1000		Nick
			1000	1000		Control

H1 ESCs (Condition D)	0.3×10^{6} cells per well (6-well plate) 1 µl Lipofectamine^{TM} 3000 + 2 µl P3000 per experimental condition					
	pCas9.gRNA^{S1}	pCas9^{D10A}.gRNA^{S1}	pCas9.gRNA^{NT}	pDonor.EP^{S1}	pDonor.EP$^{S1.TS}$	Gene targeting strategy
Size (bp)	9450	9450	9447	7881	8041	
DNA (ng/well)	500			500		Standard
		500			500	Nick^{2}
		500		500		Nick
			500	500		Control

LUMC0044iCtrl44 iPSCs (Condition E)	0.3×10^{6} cells per well (6-well plate) 1 µl Lipofectamine^{TM} 3000 + 2 µl P3000 per experimental condition					
	pCas9.gRNA^{S1}	pCas9^{D10A}.gRNA^{S1}	pCas9.gRNA^{NT}	pDonor.EP^{S1}	pDonor.EP$^{S1.TS}$	Gene targeting strategy
Size (bp)	9450	9450	9447	7881	8041	
DNA (ng/well)	500			500		Standard
		500			500	Nick^{2}
		500		500		Nick
			500	500		Control

Supplementary Table 22. Transfection scheme for the experiment investigating apoptosis in ESCs triggered by Cas9 versus Cas9^{D10A} presented in **supplementary Fig. 12**.

H1 ESCs	5×10^5 cells per well (6-well plate)<br>1 µl Lipofectamine^{TM} 3000 + 2 µl P3000 per experimental condition						
	pCas9.gRNA^{S1}	pCas9^{D10A}.gRNA^{S1}	pCas9.gRNA^{NT}	pDonor^{S1}	pDonor$^{S1.TS}$	pgRNA^{Empy}	Gene targeting strategy
Size (bp)	9450	9450	9447	7199	7359	3915	
DNA(ng/well)	500			500			Standard
		500			500		Nick^2
		500		500			Nick
	500				500		DSB^2
			500	500			Ctrl
			500			500	Ctrl

Supplementary Table 23. Primer pairs and composition of PCR mixtures used for the analyses of genome-modifying events (**Supplementary Fig. 1a** ,**1b** and **1d** , **Fig. 2c** and **2e**, **Fig. 4d**, **Fig 5d**, **Fig. 6c**, **6d** and **6e**).

Target	Primer code	Primers (5' → 3') / final concentrations (mM)	dNTPs (mM)	$MgCl_2$ (mM)	GoTaq Flexi Buffer	GoTaq (Units)
AAVS1 (T7EI assay)	#999	TTCGGGTCACCTCTCACTCC / 0.4	0.4	1	1×	2
	#1000	GGCTCCATCGTAAGCAAACC / 0.4				
jC-*AAVS1*	#1046	CGACAACCACTACCTGAGCA / 0.4	0.4	1	1×	2.5
	#1047	GACCTGCCTGGAGAAGGAT / 0.4				
jH-T concatemer	#1061	TGCTCTAAGGGCGAATTCG / 0.4	0.4	2	1×	2.5
	#1062	ATGGTCATAGCTGTTTCCTG / 0.4				
EGFP	#978	GAGCTGGACGGCGACGTAAACG / 0.4	0.4	1	1×	2.5
	#979	CGCTTCTCGTTGGGGTCTTTGCT / 0.4				
HPRT1	#1005	TCACTGTATTGCCCAGGTTGGTG / 0.2	0.4	3	1×	2
	#1006	GAAAGCAAGTATGGTTTGCAGAGAT / 0.2				
DMD (T7EI assay)	#994	AGACATGACCTATTCTGCCG / 0.2	0.4	1	1×	2
	#1052	GGTAGTAAGTCAGATGTGATG / 0.2				
t(X;19)(p21; q13)	#1000	GGCTCCATCGTAAGCAAACC / 0.2	0.4	1	1×	2.5
	#1052	GGTAGTAAGTCAGATGTGATG / 0.2				
j-mTurquoise2	#1251	ACTACCTGAGCACCCAGTCCAAG / 0.2	0.4	1	1×	2.5
	#1253	TCACCCAGAGACAGTGACCAACC / 0.2				
j-EGFP	#1252	ACCTGAGCACCCAGTCCGCC / 0.2	0.4	1	1×	2.5
	#1253	TCACCCAGAGACAGTGACCAACC / 0.2				
AAVS1 (RFLP analysis)	#1257	AGCCACCTCTCCATCCTCTT / 0.2	0.4	1	1×	2.5
	#1258	AGGGAGTTTTCCACACGGAC / 0.2				
CCR5 (RFLP analysis)	#1280	GTGCATGTTCTTTGTGGGCT / 0.2	0.4	1	1×	2.5
	#1281	GGTCAGAGATGGCCAGGTTG / 0.2				

Supplementary Table 24. PCR cycling parameters used for the analyses of genome-modifying events (**Supplementary Fig. 1a** ,**1b** and **1d** , **Fig. 2c** and **2e**, **Fig. 4d**, **Fig 5d**, **Fig. 6c**, **6d** and **6e**).

Target	Initial denaturation	Denaturation	Annealing	Elongation	Cycles	Final elongation
AAVS1 (T7EI assay)	95 °C	95 °C	61 °C	72 °C	40	72°C
	3 min	30 s	30 s	30 s		5 min
jC-*AAVS1*	95 °C	95 °C	61 °C	72 °C	40	72 °C
	5 min	30 s	30 s	120 s		5 min
jH-T concatemer	95 °C	95 °C	57 °C	72 °C	35	72 °C
	3 min	25 s	25 s	20 s		2 min
EGFP	95 °C	95 °C	62 °C	72 °C	40	72 °C
	3 min	30 s	30 s	40 s		3 min
HPRT1	95 °C	95 °C	63 °C (decrease of 0.5 °C per cycle)	72 °C	10	
	3 min	25 s	30 s	25 s		
		95 °C	58 °C	72 °C	25	72 °C
		25 s	25 s	25 s		3 min
DMD (T7EI assay)	95 °C	95 °C	66 °C (decrease of 0.5 °C per cycle)	72 °C	10	
	5 min	30 s	30 s	30 s		
		95 °C	61 °C	72 °C	25	72 °C
		30 s	30 s	30 s		3 min
t(X;19)(p21;q13)	95 °C	95 °C	60 °C	72 °C	40	72 °C
	5 min	30 s	30 s	35 s		5 min
j-mTurquoise2	95 °C	95 °C	68 °C	72 °C	40	72 °C
	5 min	30 s	30 s	100 s		5 min
j-EGFP	95 °C	95 °C	67.4 °C	72 °C	40	72 °C
	5 min	30 s	30 s	100 s		5 min
AAVS1 (RFLP analysis)	95 °C	95 °C	58.3 °C	72 °C	40	72 °C
	5 min	30 s	30 s	60 s		5 min
CCR5 (RFLP analysis)	95 °C	95 °C	58.7 °C	72 °C	40	72 °C
	5 min	30 s	30 s	55 s		5 min

Supplementary Table 25. Thermocycler program for generating T7EI heteroduplex substrates spanning nuclease target sites.

Temperature	Time
95 °C	10 min
95 °C to 85 °C	-2.0 °C/s
85 °C	1 min
85 °C to 75 °C	-0.3 °C/s
75 °C	1 min
75 °C to 65 °C	-0.3 °C/s
65 °C	1 min
65 °C to 55 °C	-0.3 °C/s
55 °C	1 min
55 °C to 45 °C	-0.3 °C/s
45 °C	1 min
45 °C to 35 °C	-0.3 °C/s
35 °C	1 min
35 °C to 25 °C	-0.3 °C/s
25 °C	1 min
16 °C	Hold

Source: SURVEYOR Mutation Detection Kit (Transgenomic)

Supplementary Table 26. Primer pairs and composition of PCR mixtures used for the analyses of individual gene-edited cells (**Fig. 1c**, **Fig. 2d** and **Fig. 3d** gathering the data from **Supplementary Figs. 3**, **7** and **13**, respectively, and **Supplementary Fig. 16**).

Target	Primer code	Primers (5' → 3')<br>@ 0.5 mM final concentration	Master mix (mM)	Primer (μM)	DMSO
jC-*AAVS1*	#1046	CGACAACCACTACCTGAGCA	0.4	0.5	-
	#1047	GACCTGCCTGGAGAAGGAT			
jT-*AAVS1*	#986	AACCCCAACCCCGTGGAAG	0.4	0.5	2%
	#1004	GCACCGTCCGCTTCGAG			
EGFP	#978	CTGCATTCTAGTTGTGGTTTG	0.4	0.5	-
	#979	CTAACATGCGGTGACGTGG			
jC-*DMD*	#1026	TGCCTTGGAAAAGGCGC	0.4	0.5	-
	#1170	AGGGACCACTGTTTTATGGGA			
jT-*DMD*	#217	CGCATTGTCTGAGTAGGTGTC	0.4	0.5	-
	#1172	ACACTCCTTTTGTCCTGCTT			
AAVS1	#999	TTCGGGTCACCTCTCACTCC	0.4	0.5	-
	#1000	GGCTCCATCGTAAGCAAACC			
mTurq.2/EGFP (RFLP analysis)	#47	ACCCCGACCACATGAAGCAGC	0.4	0.5	-
	#1253	TCACCCAGAGACAGTGACCAACC			

Supplementary Table 27. Cycling parameters used for the junction PCR analyses of individual gene-edited cells (**Fig. 1c**, **Fig. 2d** and **Fig. 3d** gathering the data from **Supplementary Figs. 3**, **7** and **13**, respectively, and **Supplementary Fig. 16**).

Target	Initial denaturation	Denaturation	Annealing	elongation	Cycles	Final elongation
jC-*AAVS1*	98 °C	98 °C	64.5 °C	72 °C	35	72 °C
	5 min	7 s	7 s	35 s		1 min
jT-*AAVS1*	98 °C	98 °C	72 °C		35	72 °C
	5 min	7 s	30 s			2 min
EGFP	98 °C	98 °C	72 °C		35	72 °C
	5 min	5 s	20 s			2 min
jC-*DMD*	98 °C	98 °C	70 °C	72 °C	40	72 °C
	5 min	7 s	7 s	50 s		2 min
jT-*DMD*	98 °C	98 °C	66 °C	72 °C	40	72 °C
	5 min	7 s	7 s	50 s		2 min
AAVS1	98 °C	98 °C	66 °C	72 °C	40	72 °C
	5 min	5 s	5 s	20 s		1 min
mTurq.2/EGFP (RFLP analysis)	98 °C	98 °C	72 °C		40	72 °C
	5 min	5 s	1 min			2 min

Supplementary Table 28. Restriction Fragment Length Polymorphism analysis presented in **Fig. 2e**, **Fig. 6c** and **6d** and **Supplementary Fig. 16**.

Target	PCR kit	Restriction Enzyme	Buffer	PCR product	Total volume	Incubation time
AAVS1	GoTaq G2 Flexi DNA polymerase (Promega)	3.3 U HindIII (ThermoFisher Scientific)	Buffer Red	20 µl	30 µl	4 h
CCR5	GoTaq G2 Flexi DNA polymerase (Promega)	3.3 U MluI (ThermoFisher Scientific)	Buffer Red	20 µl	30 µl	4 h
EGFP/mTurquoise2-AAVS1	Phire Tissue Direct PCR Master Mix (ThermoFisher Scientific)	3.3 U BlpI (New England BioLab)	Cutsmart	10 µl	20 µl	Overnight
