# Supplementary material for: In trans paired nicking triggers seamless genome editing without double-stranded DNA cutting
Source: Nat Commun. 2017 Sep 22;8:657. doi: 10.1038/s41467-017-00687-1 (PMC5610252; doi:10.1038/s41467-017-00687-1)
Supplement: Supplementary file 2 — Peer Review File [file 41467_2017_687_MOESM2_ESM.pdf]

Editorial Note: this manuscript has been previously reviewed at another journal that is not operating a transparent peer review scheme. This document only contains reviewer comments and rebuttal letters for versions considered at Nature Communications.
